# Unveiling the catalytic potential of silicomolybdic acid in crafting diverse biologically relevant organic compounds

**DOI:** 10.1039/d5ra03549j

**Published:** 2025-08-13

**Authors:** Neeraj K. Sah, Krishna Kumar, Subrato Bhattacharya, Tanay Pramanik, Tanmoy Roy, Somenath Garai

**Affiliations:** a Department of Chemistry, Institute of Science, Banaras Hindu University Varanasi 221 005 Uttar Pradesh India neerajkumarsah5@gmail.com gl.krishna91@gmail.com s_bhatt@bhu.ac.in somgor@gmail.com; b Department of Chemistry, Institute of Engineering and Management, University of Engineering and Management Kolkata University Area, Action area 3, Newtown Kolkata 700160 India tanay.pramanik@gmail.com; c Department of Chemistry, Lovely Professional University Jalandhar-Delhi, G.T. Road Phagwara Punjab 144411 India tanmoyroyk@gmail.com

## Abstract

Silicomolybdic acid (SMA) is a widely explored heteropoly acid with key advantages, including easy recovery, safer handling, and strong acid strength. In this study, we report the first comprehensive application of SMA, a Keggin-type heteropoly acid, as an efficient and reusable Lewis acid catalyst for the synthesis of a range of oxygen- and nitrogen-containing heterocycles. The catalytic performance of SMA was demonstrated in the synthesis of biologically relevant chromene, imidazopyrimidine, xanthene, and benzylidene malononitrile derivatives *via* one-pot multicomponent reactions. All reactions proceeded under mild conditions using low catalyst loadings and environmentally benign solvents, affording excellent yields within short reaction times. A key advantage of this protocol is that it eliminates the need for column chromatography, enabling simple work-up and product isolation. Additionally, gram-scale synthesis and catalyst recyclability were successfully demonstrated, highlighting the practical utility of the method. Compared to existing protocols, this approach offers multiple benefits, including operational simplicity, shorter reaction durations, room temperature conditions, and high atom economy. Notably, SMA retained its catalytic activity over multiple cycles with minimal loss in efficiency. These findings establish SMA as a green, practical, and versatile catalyst for the sustainable synthesis of pharmacologically significant heterocycles, with strong potential for future broader applications in synthetic and medicinal chemistry.

## Abstract 2

This study examines silicomolybdic acid's catalytic potential for synthesizing chromene, imidazopyrimidine, xanthene, and benzylidene malononitrile derivatives efficiently.

## Graphical abstract

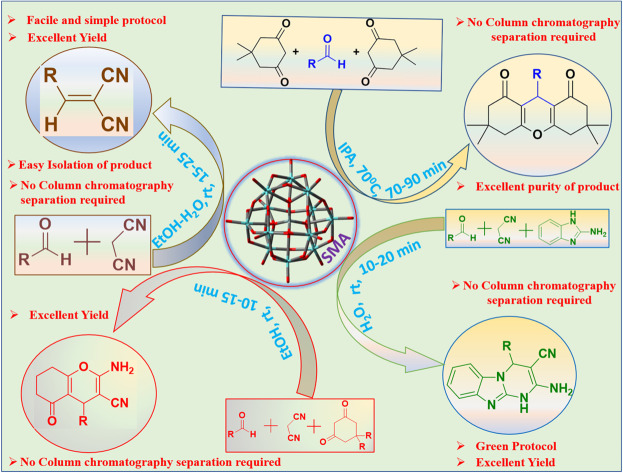

## 1. Introduction

**Fig. 1:**
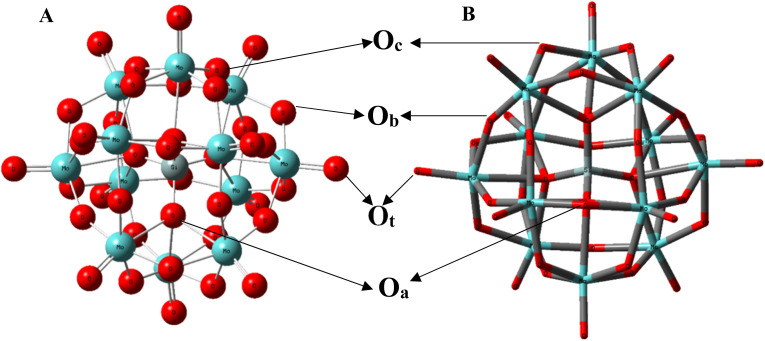
(A) Ball and bond representation of SMA (B) tube representation of SMA showing all four types of oxygen atoms where olive green balls correspond to silicon, sea green balls to molybdenum and red balls to oxygen atoms. While red balls connected *via* double bond represent terminal oxygen atoms (O_t_), red balls connected with silicon atom correspond to μ_4_-O_a_ type oxygen atoms and rest red balls correspond to μ_2_-O_b_ and μ_2_-O_c_ type oxygen atoms.

Lewis acid catalysts are often of major significance in the field of organocatalysis as they can facilitate chemical reactions by accepting electron pairs from other molecules or ions, thus promoting the formation of new bonds for a variety of organic transformations.^1^ Unlike traditional catalysts that primarily rely on providing a surface for reactants to interact, Lewis acids function by creating an electron-deficient environment, which enhances the reactivity of specific reaction partners. The catalyst itself remains unchanged at the end of the reaction, making it a highly efficient and versatile tool in various chemical transformations.^2^ This unique ability to activate otherwise inert molecules has profound implications in fields ranging from organic synthesis to industrial processes, making Lewis acid catalysts indispensable in modern chemistry.^3^ Common examples of Lewis acid catalysts include boranes, metal salts (such as metal chloride, perchlorate, triflate, *etc.*), *p*-toluene sulfonic acid and heteropoly acids.^1–4^ Heteropolyacid (HPAs) are well known Lewis acid catalysts owing to their distinctive physicochemical characteristics. In the literature, certain HPAs have been described as active catalysts.^5^ Silicomolybdic acid (SMA), with the empirical formula H_4_SiMo_12_O_40_·29H_2_O, is one of the most widely studied heteropoly acids from the Keggin family [(XM_12_O_40_)^*n*−^, where X = As, Si, P, Ge and M = V, Mo, Nb, W, Ta; *n* = 3, 4]. It has been extensively researched in fields such as analytical chemistry, biochemistry, materials science, and catalysis.^6,7^ The structure of this SMA exhibits Td symmetry, consisting of a central SiO_4_ tetrahedron surrounded by 12 interconnected MoO_6_ octahedra (Fig. 1). These octahedra form four triangular Mo_3_O_13_ units, also known as triads, where each triad consists of three edge-sharing MoO_6_ octahedra connected by μ_2_-oxo bridging Mo–O–Mo (μ_2_-O_c_). Additionally, the Mo_3_O_13_ triads are linked to one another through corner-sharing *via* μ_2_-oxo bridging Mo–O–Mo (μ_2_-O_b_), and each triad shares an oxygen atom (μ_4_-O_t_) with the central silicon atom through μ_4_-oxo bridging. Earlier, it has been utilized as homogeneous and supportive catalyst in a variety of electrophilic reactions (hydration, dehydration, isomerization, alkylation, and acylation) of different organic compounds rather frequently.^8–14^ In addition to their very strong acid strength,^15,16^ they have certain particularly advantageous technological aspects including simple recovery and handling that is safer for the environment.^8,11^ It is worth noting that the promotion of redox processes is also possible, most importantly, it is composed of the constituent atoms that have the ability to change their valence state while retaining the original structure of the acid.^16,17^ When compared to the acidity of conventional mineral acid catalysts, these solids have much more Brønsted acidic sites and are soluble in most organic solvents as well as water, as a result, both homogeneous and heterogeneous catalytic processes frequently utilize them as acid catalysts.^5^ For example, Török *et al.* have reported efficient catalytic activity of SMA towards the dehydration of butane-1,4-diol to tetrahydrofuran with excellent yield and high selectivity at 150 °C.^16^ During the dehydration process, the formation of blue heteropoly acid (reduced form of SMA) is observed which is confirmed by one-electron reduction in SMA from Mo(vi) → Mo(v) with the help of ESR spectroscopy. In another work, Dash and coworkers have intercalated SMA into graphene oxide and utilized it as a catalyst for the preparation of nitrogen-containing heterocycle isoxazole derivatives using microwave irradiation.^5^ They have performed this reaction in water with various aromatic aldehyde derivatives, hydroxyl amine hydrochloride and ethyl acetoacetate and have got excellent yield of isoxazole derivative within a short span of time. Similarly, Saher *et al.* have used different heteropoly acids as catalysts for the preparation of nitrogen-based heterocyclic compounds like 3,4-dihydropyrimidinones derivatives and they found better yield of products with SMA as compared to other heteropoly acids.^18^ Moreover, there have been some instances where many Lewis acid catalysts have been examined for a particular organic transformation and SMA has shown greater catalytic activity than others. For instance, Song and coworkers have investigated several Lewis acid catalysts for the degradation of polytetrahydrofuran to obtain tetrahydrofuran where SMA has revealed degradation efficiently with comparatively better yield and shorter time of degradation.^19^

Known for its potential, the Knoevenagel condensation is a frequently occurring reaction in synthetic organic chemistry that generates a C–C bond. This reaction involves condensation of carbonyl compound and active methylene group in an acidic or basic medium.^20^ The electrophilic olefins that result from this reaction are typically a highly helpful precursor for many chemical transformations including the Michel addition, Diels–Alder reaction and a variety of significant pharmaceutical items such as the popular and effective tyrosine kinase inhibitor 2-(3,4,5-trimethoxybenzylidene)malononitrile.^21,22^ Moreover, benzylidenemalononitrile derivatives, the most common Knoevenagel product obtained by the condensation of benzaldehyde derivatives and malononitrile, exhibit excellent biological activity like anticancer, antifungal and antibacterial activity.^23^ Over the past few decades, a broad variety of catalysts have been exploited to obtain these derivatives, each offering variable product yields. Under homogeneous conditions, weak bases typically catalyze the reaction; but, in the past decades, a rapid increase in heterogeneous catalysts, primarily made of inorganic substances such as clays and zeolites, has been seen.^24^ Some Lewis acid catalysts have also been exploited for this reaction. Similarly, xanthene core and its derivatives are a significant group of organic compounds, which are prevalent in natural products with a variety of biological functions.^25^ The most notable of them is xanthenedione, which is a structural component of many natural products and has a variety of pharmacological and therapeutic characteristics.^25,26^ Several functionalized, 1,8-dioxooctahydroxanthene derivatives have received considerable attention from many pharmaceutical and organic chemists owing to their anticancer, antiplasmodial, antiviral, antibacterial, and anti-inflammatory properties.^25,27^ Additionally, these heterocyclic compounds have found extensive utility as pH-sensitive fluorescent materials, photodynamic treatment sensitizers, luminous dyes, and laser technologies.^25,28^ As a result, many techniques have been established for the formation of xanthene derivatives, which are typically produced by the condensation of aldehydes with suitable active methylene compounds with the help of various catalysts. However, the majority of the published procedures need pricey ingredients, toxic organic solvents, prolonged reaction times, and time-consuming workup.^28,29^ SMA has been the focal point of our investigations owing to their variable Lewis acidity and redox properties.

The multicomponent reaction (MCR) offers a potent technique for the efficient production of a large variety of chemicals, comprising medicines, complex organic molecules, and biologically active substances.^30^ The MCR has been widely utilized in the preparation of natural products and other biologically active compounds ever since their discovery more than 170 years ago.^31^ MCRs have gained much consideration recently due to their beneficial qualities, including high efficiency, appealing conditions, simple completion, and environmental friendliness.^32^ The chromene ring system is regarded as one of nature's most important heterocycles as it serves as the parent ring for so many biologically significant derivatives. The present curiosity in 4H- and 2H-chromene derivatives stems from their potential use as anticancer, antitumor, anti-inflammatory, antimicrobial, antitubercular, anti-HIV, cytotoxic agent and antioxidant.^30–32^ Several techniques have been established for the production of 4H-chromene derivatives owing to their biological significance. In this scenario, the MCR of an aldehyde, an active methylene group and an enolizable C–H-activated acidic compound is one of the most adopted methods. This reaction has been carried out using a variety of homogeneous or heterogeneous catalysts so far. Similarly, the imidazopyrimidine derivatives, which include N-containing heterocycles of the pyrimidines and imidazole frameworks, are a significant class of nitrogen-containing heterocyclic compounds from the viewpoint of their pharmaceutical, agrochemical, and biological properties.^33^ It has numerous biological properties including anti-diabetes, anti-arrhythmia, anti-vascular hypertension, antimicrobial, anti-inflammatory, antiviral, anticonvulsant, anti-hepatitis B, and anticancer activities.^33–35^ Other therapeutic features of these compounds include anti-diabetic, DNA-gyrase inhibitors, antiulcer, platelet anti-aggregant, herbicidal, and molluscicided actions.^35,36^ After carefully examining the methods employed to make 2-amino-4-substituted-1,4-dihydrobenzolo[4,5]imidazo[1,2-*a*]pyrimidine-3-carbonitriles, we found that the majority of them involve one-pot, multi-component reaction involving active nitriles, aldehydes, and aminobenzimidazole.^37^ Even while these processes advance science, several of them have drawbacks, including lengthy reaction durations, difficult reaction environments, the utilization of hazardous reagents and solvents, the necessity to employ a lot of catalysts and the catalyst's non-recyclability. Therefore, it is still urgently necessary to introduce cheap, effective, and easily recoverable catalysts to quicken the aforementioned condensation reactions. SMA has been the focal point of our investigations owing to their variable Lewis acidity and redox properties.

In this study, we have explored SMA as a Lewis acid catalyst to prepare various biologically significant N-fused heterocyclic organic compounds. Herein, we have reported the synthesis of benzylidenemalononitrile derivatives with excellent yield using aromatic aldehydes, malononitriles and SMA in the solvent ethanol–water mixture at room temperature. Similarly, one-pot preparation of 2-amino-5-oxo-5,6,7,8-tetrahydro-4*H*-chromenes derivatives have been performed using SMA, dimedone or cyclohexane-1,3-dione, malononitrile and aromatic aldehydes in the solvent ethanol with an excellent yield at room temperature. Furthermore, several derivatives of 2-amino-4-substituted-1,4-dihydrobenzo[4,5]imidazolo[1,2-*a*]pyrimidine-3-carbonitriles have been prepared through the simple one-pot synthesis of 2-aminobenzimidazole, aromatic aldehydes and malononitrile in water using SMA at room temperature within a short span of time with an excellent yield. Moreover, the preparation of 1,8-dioxooctahydroxanthene derivatives have been reported using SMA, dimedone and aromatic aldehydes in the solvent isopropyl alcohol within a short span of time with an excellent yield. Although numerous reports on silicomolybdic acid (SMA) are available, this article presents the first instance of SMA catalysing these four multicomponent reactions, as they have not been previously reported with this catalyst. Although these four reactions have been reported in the literature with different catalysts, the potential of SMA remains unexplored. Utilizing SMA for these reactions offers several advantages over other reported catalysts. Notably, column chromatography is not required for product separation, as the product can be easily isolated through simple filtration, significantly reducing both the preparation time and overall cost. Additionally, the product conversion in all reactions is 100% with no by-products, ensuring high purity. Moreover, the reaction times are comparable to those achieved with other catalysts reported in the literature.

## 2. Experimental section

### 2.1. General information

All experiments were performed in the open air at ambient temperature and pressure. The solvents were purified by standard reported procedures and dried before use where necessary.^38^ Chemicals were purchased from Sigma-Aldrich and Avra Chemical Companies. The melting points of the complexes were determined in open capillaries using a Gallenkamp apparatus and are uncorrected. The NMR spectra were recorded in deuterated solvents [CDCl_3_ and DMSO-d_6_] on a JEOL ECZ 500 MHz FT NMR spectrometer. Chemical shifts are quoted in parts per million (ppm) downfield from the internal tetramethylsilane (TMS), and coupling constant (*J*) values are given in hertz (Hz). Thin-layer chromatography (TLC) was performed on a Merck 60 F254 silica gel, precoated on aluminium plates. Column chromatography was performed on silica gel 100–200 meshes (Merck). HR-MS analysis was carried out using a Sciex-X500R QTOF instrument. UV-visible (200–800 nm) spectra were obtained with Shimadzu UV-1700 Pharmaspec UV-visible spectrophotometer in aqueous solutions. Infrared spectra were recorded with a Varian-3100 FTIR spectrometer. The theoretical calculations were conducted using Gaussian-16 software and the semi-empirical parameterization method 6 (PM6) method was employed in our theoretical investigation.^39^ Note: Acetonitrile and malononitrile are considered a toxic and dangerous solvent due to its potential to cause cyanide poisoning when metabolized by the body, it should be handled with extreme caution and appropriate safety measures taken when working with it. Silicomolybdic acid is considered toxic, primarily causing irritation to skin and eyes, and can be harmful if ingested or absorbed through the skin; it is important to handle it with appropriate safety precautions like gloves and eye protection when working with it.

### 2.2. Synthesis of silicomolybdic acid

It was prepared by modified procedure in the literature^6,7^ 12 g of NaOH was dissolved in 180 mL water and the solution was heated up to boiling, then 35 g of molybdic acid was added to it in boiling condition, and the solution was completely dissolved then 50 mL of concentrated nitric acid was added to it and the solution was kept for stirring. Another solution was prepared, where 5 g of sodium metasilicate was dissolved in 125 mL of 2 N NaOH and the solution was boiled, then it was added to the former solution immediately. The mixture was stirred for 2–3 hours then it was kept in the separatory funnel then 15 mL concentrated HNO_3_ and 50 mL diethyl ether was added into the solution. The solution was shaken well for 5–10 minutes and kept to settle down for a while then the desired silicomolybdic acid was collected and left for crystallization.^7^ FTIR (cm^−1^): 1097 *ν*_s_ (Si–O), 958 *ν*_as_ (M=O), 909 *ν*_as_ (Mo–O–Mo), 780 *ν*_as_ (M–O–M).12Na_2_MoO_4(aq)_ + Na_2_SiO_3(aq)_ + 22HNO_3_ → Na_4_[SiMo_12_O_40_]_(aq)_ + 22NaNO_3_

### 2.3. General procedure for the preparation of compounds 1a–m

In a typical reaction, benzaldehyde (1.0 mmol), malononitrile (1.0 mmol), and SMA (1 mol%) were added to a 20 mL vial containing an ethanol–water mixture (2 : 2 mL). The reaction mixture was stirred vigorously at room temperature for 15–20 minutes. Upon achieving complete conversion of the precursor (monitored by TLC), stirring was stopped, and the reaction setup was left undisturbed overnight, allowing the product to precipitate. The solid product was then collected by simple filtration, washed 2–3 times with water, and the catalyst was recovered from the filtrate by evaporation of solvent. In most cases, a pure crystalline product was obtained directly. However, when necessary, recrystallization from hot ethanol was performed to ensure purity. The final product was obtained as a white solid, which was further characterized by NMR spectral analysis. The isolated yield, characterization data, and representative NMR spectra of the Knoevenagel condensation products are given in Table 2 and SI.

#### 2.3.1 2-Benzylidenemalononitrile (1a)

White powder. Yield: 146 mg (95%). ^1^H NMR (500 MHz, CDCl_3_): *δ* 7.91 (d, *J* = 7.6 Hz, 2H), 7.78 (s, 1H), 7.64 (t, *J* = 7.4 Hz, 1H), 7.55 (t, *J* = 7.4 Hz, 2H) ppm. ^13^C {^1^H} NMR (125 MHz, CDCl_3_): *δ* 160.03, 134.72, 131.02, 130.82, 129.72, 113.78, 112.62, 82.97 ppm.

#### 2.3.2 2-(4-Fluorobenzylidene) malononitrile (1b)

White powder. Yield: 143 mg (83%). ^1^H NMR (500 MHz, CDCl_3_): *δ* 8.00 (m, 2H), 7.78 (s, 1H), 7.33–7.23 (m, 2H) ppm. ^13^C {^1^H} NMR (125 MHz, CDCl_3_): *δ* 158.35, 133.53, 133.46, 117.38, 117.21, 113.62, 112.55, 82.55 ppm.

#### 2.3.3 2-(4-Chlorobenzylidene) malononitrile (1c)

White powder. Yield: 168 mg (89%). ^1^H NMR (500 MHz, CDCl_3_): *δ* 7.86 (d, *J* = 8.6 Hz, 2H), 7.73 (s, 1H), 7.52 (d, *J* = 9.2 Hz, 1H) ppm. ^13^C {^1^H} NMR (125 MHz, CDCl_3_): *δ* 158.32, 141.25, 131.90, 130.16, 129.35, 113.49, 112.39, 83.48 ppm.

#### 2.3.4 2-(Furan-2-ylmethylene) malononitrile (1d)

Pale yellow powder. Yield: 133 mg (92%). ^1^H NMR (500 MHz, CDCl_3_): *δ* 7.80 (s, 1H), 7.51 (s, 1H), 7.36 (d, *J* = 3.2 Hz, 1H), 6.72 (d, *J* = 4.5 Hz, 1H) ppm. ^13^C {^1^H} NMR (125 MHz, CDCl_3_): *δ* 149.59, 148.17, 143.12, 123.45, 114.51, 113.84, 112.64 ppm.

#### 2.3.5 2-(4-Methylbenzylidene) malononitrile (1e)

White powder. Yield: 160 mg (95%). ^1^H NMR (500 MHz, CDCl_3_): *δ* 7.81 (d, *J* = 7.9 Hz, 2H), 7.72 (s, 1H), 7.34 (d, *J* = 8.0 Hz, 2H) ppm. ^13^C {^1^H} NMR (125 MHz, CDCl_3_): *δ* 159.85, 146.46, 130.99, 130.46, 128.56, 114.09, 112.94, 81.29, 22.08 ppm.

#### 2.3.6 2-(2,5-Dimethoxybenzylidene)malononitrile (1f)

Yellow powder. Yield: 182 mg (85%). ^1^H NMR (500 MHz, CDCl_3_): *δ* 8.27 (s, 1H), 7.71 (d, *J* = 3 Hz, 1H), 7.17–7.15 (m, 1H), 6.92 (d, *J* = 9.5 Hz, 1H), 3.88 (s, 3H), 3.80 (s, 3H) ppm. ^13^C {^1^H} NMR (125 MHz, CDCl_3_): *δ* 154.16, 153.88, 153.57, 124.30, 120.25, 114.42, 113.28, 112.96, 111.36, 81.05, 56.43, 55.95 ppm.

#### 2.3.7 2-(3-Methylbenzylidene) malononitrile (1g)

White powder. Yield: 152 mg (90%). ^1^H NMR (500 MHz, CDCl_3_): *δ* 7.80 (d, *J* = 8 Hz, 2H), 7.72 (s, 1H), 7.33 (d, *J* = 8 Hz, 2H), 2.48 (s, 3H) ppm. ^13^C {^1^H} NMR (125 MHz, CDCl_3_): *δ* 159.86, 146.47, 131.00, 130.46, 128.57, 114.10, 112.95, 81.27, 22.08 ppm.

#### 2.3.8 2-(4-Bromobenzylidene) malononitrile (1h)

Yellow powder. Yield: 210 mg (90%). ^1^H NMR (500 MHz, CDCl_3_): *δ* 7.85 (d, *J* = 8 Hz, 2H), 7.73 (s, 1H), 7.51 (d, *J* = 9 Hz, 2H) ppm. ^13^C {^1^H} NMR (125 MHz, CDCl_3_): *δ* 158.38, 141.22, 131.92, 130.15, 129.38, 113.52, 112.42, 83.45, ppm.

#### 2.3.9 2-(4-Methoxybenzylidene)malononitrile (1i)

Yellowish green powder. Yield: 170 mg (92%). ^1^H NMR (500 MHz, CDCl_3_): *δ* 7.90 (d, *J* = 8.5 Hz, 2H), 7.64 (s, 1H), 7.00 (d, *J* = 8.5 Hz, 1H), 3.91 (s, 3H) ppm. ^13^C {^1^H} NMR (125 MHz, CDCl_3_): *δ* 164.93, 158.94, 133.53, 131, 128.92, 124.12, 115.23, 114.51, 113.43, 55.88 ppm.

#### 2.3.10 2-(4-Nitrobenzylidene) malononitrile (1j)

Faint yellow powder. Yield: 171 mg (86%). ^1^H NMR (500 MHz, CDCl_3_): *δ* 8.38 (s, 2H), 8.07 (s, 2H), 7.89 (s, 1H), ppm. ^13^C {^1^H} NMR (125 MHz, CDCl_3_): *δ* 156.95, 150.44, 131.39, 124.71, 111.67, 87.63, ppm.

#### 2.3.11 2-(2,4,5-Trimethoxybenzylidene)malononitrile (1k)

Yellow powder. Yield: 215 mg (88%). ^1^H NMR (500 MHz, CDCl_3_): *δ* 7.65 (s, 1H), 7.18 (s, 2H), 3.96 (s, 3H), 3.89 (s, 6H) ppm. ^13^C {^1^H} NMR (125 MHz, CDCl_3_): *δ* 159.54, 153.44, 144.11, 126.03, 114.10, 113.30, 108.44, 80.56, 61.29, 56.43, ppm.

#### 2.3.12 2-(Naphthalen-1-ylmethylene)malononitrile (1l)

Yellow powder. Yield: 168 mg (82%). ^1^H NMR (500 MHz, CDCl_3_): *δ* 8.64 (s, 1H), 8.27 (d, *J* = 7 Hz, 1H), 8.10 (d, *J* = 8.5 Hz, 1H), 7.95 (d, *J* = 8 Hz, 2H), 7.70–7.59 (m, 3H) ppm. ^13^C {^1^H} NMR (125 MHz, CDCl_3_): *δ* 157, 135, 133.62, 131.15, 129.51, 128.65, 128.58, 127.38, 125.46, 122.38, 113.80, 112.59, 85.23, ppm.

#### 2.3.13 2-(4-Cyanobenzylidene)malononitrile (1m)

Faint yellow powder. Yield: 169 mg (94%). ^1^H NMR (500 MHz, CDCl_3_): *δ* 7.99 (d, *J* = 8 Hz, 1H), 7.82 (d, *J* = 9 Hz, 3H), ppm. ^13^C {^1^H} NMR (125 MHz, CDCl_3_): *δ* 157.53, 134.36, 133.22, 130.79, 117.38, 117.31, 112.76, 111.79, 86.93, ppm.

### 2.4. General procedure for the synthesis of compounds 2a–j

In a typical procedure, benzaldehyde (1.0 mmol), malononitrile (1.0 mmol), cyclohexane-1,3-dione or dimedone (1.0 mmol), and SMA (1 mol%) were combined in a 20 mL vial containing 2–4 mL of ethanol. The reaction mixture was stirred vigorously at room temperature for 5–10 minutes. Upon achieving complete conversion of the precursor (monitored by TLC), stirring was stopped, and the reaction setup was left undisturbed overnight, allowing the product to precipitate. The solid product was then collected by simple filtration, washed 2–3 times with water, and the catalyst was recovered from the filtrate by evaporation of solvent. In most cases, a pure crystalline product was obtained directly. However, when necessary, recrystallization from hot ethanol was performed to ensure purity. The final product was obtained as a white solid, which was further characterized by NMR spectral analysis. The isolated yield, characterization data, and representative NMR spectra of the chromene derivatives are given in Table 5 and SI.

#### 2.4.1 2-Amino-4-(4-chlorophenyl)-5-oxo-5,6,7,8-tetrahydro-4*H*-chromene-3-carbonitrile (2a)

White powder. Yield: 256 mg (85%). ^1^H NMR (500 MHz, DMSO-d_6_): *δ* 7.32 (d, *J* = 7.9 Hz, 2H), 7.16 (d, *J* = 7.9 Hz, 2H), 6.99 (s, 2H), 4.18 (s, 1H), 2.59 (m, 2H), 2.26 (m, 2H), 1.90 (m, 2H) ppm. ^13^C {^1^H} NMR (125 MHz, DMSO-d_6_): *δ* 196.78, 165.38, 158.97, 144.20, 131.67, 129.56, 128.80, 120.15, 113.78, 58.34, 36.78, 35.47, 26.95, 20.23 ppm.

#### 2.4.2 2-Amino-4-(4-nitrophenyl)-5-oxo-5,6,7,8-tetrahydro-4*H*-chromene-3-carbonitrile (2b)

White powder. Yield: 268 mg (86%). ^1^H NMR (500 MHz, DMSO-d_6_): *δ* 8.14 (d, *J* = 9.0 Hz, 2H), 7.44 (d, *J* = 9.0 Hz, 2H), 7.11 (s, 1H), 4.34 (s, 1H), 2.63 (m, 2H), 2.27 (m, 2H), 1.93 (m, 2H) ppm. ^13^C {^1^H} NMR (125 MHz, DMSO-d_6_): *δ* 196.69, 165.82, 159.05, 152.78, 146.75, 129.05, 124.15, 119.90, 113.16, 57.52, 36.71, 36.06, 27.00, 20.21 ppm.

#### 2.4.3 2-Amino-5-oxo-4-phenyl-5,6,7,8-tetrahydro-4*H*-chromene-3-carbonitrile (2c)

White powder. Yield: 245 mg (92%). ^1^H NMR (500 MHz, DMSO-d_6_): *δ* 7.24 (m, 6H), 4.53 (s, 1H), 4.42 (s, 1H), 2.59 (m, 2H), 2.34 (m, 2H), 2.03 (m, 2H) ppm. ^13^C {^1^H} NMR (125 MHz, DMSO-d_6_): *δ* 196.38, 165.00, 159.01, 145.31, 128.85, 127.64, 127.05, 120.29, 114.33, 58.78, 36.85, 35.97, 26.99, 20.33 ppm.

#### 2.4.4 2-Amino-5-oxo-4-(*m*-tolyl)-5,6,7,8-tetrahydro-4*H*-chromene-3-carbonitrile (2d)

White powder. Yield: 250 mg (89%). ^1^H NMR (500 MHz, DMSO-d_6_): *δ* 7.16 (t, *J* = 8.0 Hz, 1H), 7.00–6.93 (m, 5H), 4.14 (s, 1H), 2.67–2.59 (m, 2H), 2.30–2.23 (m, 5H), 1.91–1.86 (m, 2H) ppm. ^13^C {^1^H} NMR (125 MHz, DMSO-d_6_): *δ* 196.28, 164.85, 159.04, 145.25, 137.83, 128.73, 128.19, 127.75, 124.80, 120.22, 114.47, 59.02, 36.89, 35.93, 27, 21.60, 20.34 ppm. HRMS (ESI^+^) *m*/*z*: [M + H]^+^ calcd. For C_17_H_17_N_2_O_2_, 281.1290; found, 281.1296.

#### 2.4.5 2-Amino-5-oxo-4-(4-methoxylphenyl)-5,6,7,8-tetrahydro-4*H*-chromene-3-carbonitrile (2e)

White powder. Yield: 263 mg (89%). ^1^H NMR (500 MHz, DMSO-d_6_): *δ* 7.07 (d, *J* = 9.0 Hz, 2H), 6.94 (s, 2H), 6.84 (d, *J* = 8.0 Hz, 2H), 4.15 (s, 1H), 3.71 (s, 3H), 2.63–2.57 (m, 2H), 2.31–2.21 (m, 2H), 1.97–1.85 (m, 2H) ppm. ^13^C {^1^H} NMR (125 MHz, DMSO-d_6_): *δ* 196.39, 164.64, 158.94, 158.45, 137.44, 128.73, 120.37, 114.20, 59.01, 55.53, 36.88, 35.15, 26.97, 20.34 ppm. HRMS (ESI^+^) *m*/*z*: [M + H]^+^ calcd. For C_17_H_17_N_2_O_3_, 297.1239; found, 297.1241.

#### 2.4.6 2-Amino-4-(4-hydroxyphenyl)-7,7-dimethyl-5-oxo-5,6,7,8-tetrahydro-4*H*-chromene-3-carbonitrile (2f)

White powder. Yield: 273 mg (88%). ^1^H NMR (500 MHz, DMSO-d_6_): *δ* 9.26 (s, 1H), 6.94 (t, *J* = 8 Hz, 4H), 6.67 (d, *J* = 8.5 Hz, 2H), 4.09 (s, 1H), 2.52 (d, *J* = 2 Hz, 2H), 2.25 (d, *J* = 16 Hz, 1H), 2.11 (d, *J* = 16 Hz, 1H), 1.05 (s, 3H), 0.97 (s, 3H) ppm. ^13^C {^1^H} NMR (125 MHz, DMSO-d_6_): *δ* 196.17, 162.51, 158.93, 156.51, 135.69, 134.41, 128.67, 120.40, 117.18, 115.52, 113.73, 59.31, 50.58, 35.23, 32.30, 28.94, 27.30 ppm.

#### 2.4.7 2-Amino-7,7-dimethyl-5-oxo-4-phenyl-5,6,7,8-tetrahydro-4*H*-chromene-3-carbonitrile (2g)

White powder. Yield: 272 mg (93%). ^1^H NMR (500 MHz, DMSO-d_6_): *δ* 7.28 (t, *J* = 7.5 Hz, 2H), 7.20–7.13 (m, 3H), 6.99 (s, 2H), 4.17 (s, 1H), 2.52 (d, *J* = 3.5 Hz, 2H), 2.25 (d, *J* = 16.0 Hz, 1H), 2.10 (d, *J* = 16.0 Hz, 1H), 1.04 (s, 3H), 0.96 (s, 3H) ppm. ^13^C {^1^H} NMR (125 MHz, DMSO-d_6_): *δ* 196.20, 163.03, 159.02, 145.26, 128.85, 127.67, 127.09, 120.25, 113.26, 58.84, 50.50, 36.10, 32.33, 28.91, 28.17, 27.32 ppm. HRMS (ESI^+^) *m*/*z*: [M + H]^+^ calcd. For C_18_H_19_N_2_O_2_, 295.1447; found, 295.1439.

#### 2.4.8 2-Amino-4-(4-chlorophenyl)-7,7-dimethyl-5-oxo-5,6,7,8-tetrahydro-4*H*-chromene-3-carbonitrile (2h)

White powder. Yield: 276 mg (84%). ^1^H NMR (500 MHz, DMSO-d_6_): *δ* 7.35 (d, *J* = 6.5 Hz, 2H), 7.17 (d, *J* = 6.5 Hz, 2H), 7.06 (s, 2H), 4.20 (s, 1H), 2.55 (d, *J* = 5.5 Hz, 2H), 2.20 (d, *J* = 16 Hz, 1H), 2.04 (d, *J* = 16.0 Hz, 1H), 1.11 (t, *J* = 7.0 Hz, 1H), 1.03 (s, 3H), 0.95 (s, 3H) ppm. ^13^C {^1^H} NMR (125 MHz, DMSO-d_6_): *δ* 196.20, 163.03, 159.02, 145.26, 128.85, 127.67, 127.09, 120.25, 113.26, 58.84, 50.50, 36.10, 32.33, 28.91, 28.17, 27.32 ppm.

#### 2.4.9 2-Amino-7,7-dimethyl-4-(4-nitrophenyl)-5-oxo-5,6,7,8-tetrahydro-4*H*-chromene-3-carbonitrile (2i)

Off white powder. Yield: 295 mg (87%). ^1^H NMR (500 MHz, DMSO-d_6_): *δ* 8.16 (d, *J* = 8.5 Hz, 2H), 7.43 (d, *J* = 11.5 Hz, 2H), 7.13 (s, 2H), 4.35 (s, 1H), 2.51 (d, *J* = 10.5 Hz, 2H), 2.25 (d, *J* = 16.0 Hz, 1H), 2.10 (d, *J* = 16.0 Hz, 1H), 1.02 (s, 3H), 0.94 (s, 3H) ppm. ^13^C {^1^H} NMR (125 MHz, DMSO-d_6_): *δ* 196.5, 163.8, 159.1, 152.8, 146.8, 129.1, 124.2, 119.9, 112.2, 57.6, 50.4, 36.2, 32.3, 28.7, 27.4 ppm.

#### 2.4.10 2-Amino-4-(4-cyanophenyl)-7,7-dimethyl-5-oxo-5,6,7,8-tetrahydro-4*H*-chromene-3-carbonitrile (2j)

White powder. Yield: 281 mg (88%). ^1^H NMR (500 MHz, DMSO-d_6_): *δ* 7.75 (d, *J* = 8.0 Hz, 2H), 7.34 (d, *J* = 8.5 Hz, 2H), 7.09 (s, 2H), 4.27 (s, 1H), 2.51 (s, 2H), 2.24 (d, *J* = 16.5 Hz, 1H), 2.10 (d, *J* = 16.5 Hz, 1H), 1.02 (s, 3H), 0.94 (s, 3H) ppm. ^13^C {^1^H} NMR (125 MHz, DMSO-d_6_): *δ* 196.5, 163.7, 159.1, 150.7, 132.9, 128.8, 119.9, 119.2, 112.2, 109.9, 57.7, 50.3, 36.3, 32.3, 28.7, 27.43 ppm.

### 2.5. General procedure for the synthesis of compounds 3a–j

In a typical reaction, benzaldehyde (1.0 mmol), malononitrile (1 mmol), 2-aminobenzimidazole (1.0 mmol), and SMA (1 mol%) were added to a 20 mL vial containing 2–4 mL water and the reaction mixture was stirred for 5–10 minutes at room temperature. The precipitate obtained was filtered and washed with 2 M sodium bicarbonate solution twice or thrice to remove catalyst. The product obtained was further recrystallized by ethanol if necessary. The final product was obtained as a white solid, which was further characterized by NMR spectral analysis. The isolated yield, characterization data, and representative NMR spectra of the imidazopyrimidine derivatives are given in Table 8 and SI.

#### 2.5.1 2-Amino-4-phenyl-1,4-dihydrobenzo[4,5]imidazo[1,2-*a*]pyrimidine-3-carbonitrile (3a)

White powder. Yield: 259 mg (90%). ^1^H NMR (500 MHz, DMSO-d_6_): *δ* 8.60 (s, 1H), 7.59 (d, *J* = 7.5 Hz, 1H), 7.30 (d, *J* = 7.0 Hz, 2H), 7.24 (d, *J* = 7 Hz, 3H), 7.17 (d, *J* = 7.5 Hz, 1H), 7.07 (t, *J* = 7.5 Hz, 1H), 6.96 (t, *J* = 7.5 Hz, 1H), 6.79 (s, 2H), 5.17 (s, 1H) ppm. ^13^C {^1^H} NMR (125 MHz, DMSO-d_6_): *δ* 152.3, 149.6, 144.1, 143.5, 129.8, 129.2, 128.4, 126.4, 123.8, 120.4, 119.7, 116.6, 112.9, 62.5, 53.7 ppm.

#### 2.5.2 2-Amino-4-(4-chlorophenyl)-1,4-dihydrobenzo[4,5]imidazo[1,2-*a*]pyrimidine-3-carbonitrile (3b)

White powder. Yield: 297 mg (94%). ^1^H NMR (500 MHz, DMSO-d_6_): *δ* 8.55 (s, 1H), 7.59 (d, *J* = 8.0 Hz, 1H), 7.38 (d, *J* = 8.5 Hz, 2H), 7.26 (d, *J* = 8.0 Hz, 2H), 7.19 (d, *J* = 7.5 Hz, 1H), 7.07 (t, *J* = 7.5 Hz, 1H), 6.96 (t, *J* = 7.5 Hz, 1H), 6.81 (s, 2H), 5.21 (s, 1H) ppm. ^13^C {^1^H} NMR (125 MHz, DMSO-d_6_): *δ* 152.04, 149.70, 144.01, 142.28, 132.88, 129.71, 129.16, 128.39, 123.85, 120.41, 119.51, 116.59, 112.92, 61.91, 53.02 ppm. HRMS (ESI^+^) *m*/*z*: [M + H]^+^ calcd for C_17_H_12_N_5_Cl 322.0859; found, 322.0833.

#### 2.5.3 2-Amino-4-(4-hydroxyphenyl)-1,4-dihydrobenzo[4,5]imidazo[1,2-*a*]pyrimidine-3-carbonitrile (3c)

White powder. Yield: 252 mg (83%). ^1^H NMR (500 MHz, DMSO-d_6_): *δ* 8.62 (s, 1H), 7.60 (d, *J* = 8.0 Hz, 1H), 7.33–7.24 (m, 5H), 7.19 (d, *J* = 8 Hz, 1H), 7.08 (d, *J* = 7.5 Hz, 2H), 6.97 (t, *J* = 7.5 Hz, 1H), 6.78 (s, 2H), 5.21 (s, 1H) ppm. ^13^C {^1^H} NMR (125 MHz, DMSO-d_6_): *δ* 152.29, 149.67, 144.02, 143.37, 129.77, 129.27, 128.46, 126.45, 123.96, 120.52, 119.72, 116.67, 112.88, 62.49, 53.75 ppm.

#### 2.5.4 2-Amino-4-(4-fluorophenyl)-1,4-dihydrobenzo[4,5]imidazo[1,2-*a*]pyrimidine-3-carbonitrile (3d)

White powder. Yield: 272 mg (89%). ^1^H NMR (500 MHz, DMSO-d_6_): *δ* 8.63 (s, 1H), 7.69 (d, *J* = 8.0 Hz, 1H), 7.39 (dd, *J* = 8, 5.0 Hz, 2H), 7.29 (d, *J* = 8 Hz, 1H), 7.25 (td, *J* = 9, 2 Hz, 2H), 7.18 (t, *J* = 7.5 Hz, 1H), 7.06 (t, *J* = 8 Hz, 1H), 6.90 (s, 2H), 5.31 (s, 1H) ppm. ^13^C {^1^H} NMR (125 MHz, DMSO-d_6_): *δ* 162.93, 161.32, 152.07, 149.64, 144.03, 139.52, 129.72, 128.55, 123.83, 120.38, 119.54, 116.57, 116.02, 115.88, 112.91, 62.26, 53.02 ppm. HRMS (ESI^+^) *m*/*z*: [M + H]^+^ calcd for C_17_H_12_N_5_F 306.1155; found, 306.1127.

#### 2.5.5 2-Amino-4-(4-bromophenyl)-1,4-dihydrobenzo[4,5]imidazo[1,2-*a*]pyrimidine-3-carbonitrile (3e)

White powder. Yield: 337 mg (92%). ^1^H NMR (500 MHz, DMSO-d_6_): *δ* 8.64 (s, 1H), 7.64 (d, *J* = 8.0 Hz, 1H), 7.49 (d, *J* = 6.5 Hz, 2H), 7.32 (d, *J* = 8 Hz, 1H), 7.29 (d, *J* = 8 Hz, 1H), 7.24 (d, *J* = 7.5 Hz, 1H), 7.12 (t, *J* = 7.5 Hz, 1H), 7.00 (t, *J* = 8 Hz, 1H), 6.90 (s, 2H), 5.29 (s, 1H) ppm. ^13^C {^1^H} NMR (125 MHz, DMSO-d_6_): *δ* 152.04, 149.89, 146.10, 144.05, 131.56, 131.25, 129.76, 129.48, 125.55, 123.96, 122.37, 120.52, 119.57, 116.69, 113.01, 61.63, 53.04 ppm. HRMS (ESI^+^) *m*/*z*: [M + H]^+^ calcd for C_17_H_12_N_5_Br, 366.0354; found, 366.0296.

#### 2.5.6 2-Amino-4-(3-methylphenyl)-1,4-dihydrobenzo[4,5]imidazo[1,2-*a*]pyrimidine-3-carbonitrile (3f)

White powder. Yield: 241 g (80%). ^1^H NMR (500 MHz, DMSO-d_6_): *δ* 8.56 (s, 1H), 7.63 (d, *J* = 8.0 Hz, 1H), 7.22 (d, *J* = 7.7 Hz, 2H), 7.11 (td, *J* = 7.0, 3 Hz, 3H), 7.06 (d, *J* = 7.5 Hz, 1H), 7.00 (t, *J* = 7.5 Hz, 1H), 6.80 (s, 2H), 5.17 (s, 1H), 2.27 (s, 3H) ppm. ^13^C {^1^H} NMR (125 MHz, DMSO-d_6_): *δ* 152.21, 149.58, 144.10, 143.36, 138.22, 129.76, 129.09, 128.96, 127.05, 123.77, 123.49, 120.28, 119.69, 116.50, 112.87, 62.39, 53.71, 21.59 ppm. HRMS (ESI^+^) *m*/*z*: [M − H]^+^ calcd for C_18_H_14_N_5_, 300.1249; found, 300.1238.

#### 2.5.7 2-Amino-4-(2,5-dimethoxyphenyl)-1,4-dihydrobenzo[4,5]imidazo[1,2-*a*]pyrimidine-3-carbonitrile (3g)

White powder. Yield: 285 mg, (82%). ^1^H NMR (500 MHz, DMSO-d_6_): *δ* 8.24 (s, 1H), 7.62 (d, *J* = 7.5 Hz, 1H), 7.23 (d, *J* = 7.5 Hz, 1H), 7.11 (t, *J* = 7.5 Hz, 1H), 7.00 (t, *J* = 7.5 Hz, 1H), 6.93 (d, *J* = 9 Hz, 1H), 6.82 (d, *J* = 9.5 Hz, 1H), 6.73 (s, 2H), 6.65 (s, 1H), 5.32 (s, 1H), 3.64 (s, 3H), 3.60 (s, 3H) ppm. ^13^C {^1^H} NMR (125 MHz, DMSO-d_6_): *δ* 153.90, 152.73, 151.40, 149.95, 144.37, 132.07, 130.08, 123.70, 120.31, 116.57, 114.23, 113.75, 113.44, 112.59, 62.46, 56.72, 56.04, 49.85 ppm. HRMS (ESI^+^) *m*/*z*: [M − H]^+^ calcd for C_19_H_17_N_5_O_2_, 346.1304; found, 346.1291.

#### 2.5.8 2-Amino-4-(3,4,5-trimethoxyphenyl)-1,4-dihydrobenzo[4,5]imidazo[1,2-*a*]pyrimidine-3-carbonitrile (3h)

White powder. Yield: 306 mg (81%). ^1^H NMR (500 MHz, DMSO-d_6_): *δ* 8.47 (s, 1H), 7.65 (d, *J* = 7.5 Hz, 1H), 7.22 (d, *J* = 7.5 Hz, 1H), 7.10 (t, *J* = 7.5 Hz, 1H), 7.00 (t, *J* = 7.5 Hz, 1H), 6.79 (d, *J* = 7.5 Hz, 2H), 6.63 (s, 2H), 5.18 (s, 1H), 3.69 (s, 6H), 3.63 (s, 3H) ppm. ^13^C {^1^H} NMR (125 MHz, DMSO-d_6_): *δ* 153.42, 144.09, 138.70, 129.81, 123.82, 120.40, 116.58, 112.87, 104.19, 60.50, 56.38, 53.86 ppm. HRMS (ESI^+^) *m*/*z*: [M − H]^+^ calcd for C_20_H_18_N_5_O_3_, 376.1410; found, 376.1404.

#### 2.5.9 2-Amino-4-(2-chlorophenyl)-1,4-dihydrobenzo[4,5]imidazo[1,2-*a*]pyrimidine-3-carbonitrile (3i)

White powder. Yield: 293 mg (91%). ^1^H NMR (500 MHz, DMSO-d_6_): *δ* 8.49 (s, 1H), 7.66 (d, *J* = 8.0 Hz, 1H), 7.48 (m, 1H), 7.34 (d, *J* = 2.5 Hz, 3H), 7.24 (d, *J* = 7.5 Hz, 1H), 7.12 (t, *J* = 7.5 Hz, 1H), 7.02 (t, *J* = 7.5 Hz, 1H), 6.88 (s, 2H), 5.64 (s, 1H) ppm. ^13^C {^1^H} NMR (125 MHz, DMSO-d_6_): *δ* 152.24, 150.07, 144.12, 139.86, 131.91, 130.30, 129.82, 128.96, 128.45, 123.92, 120.51, 119.04, 116.64, 112.99, 61.35, 51.38 ppm.

#### 2.5.10 2-Amino-4-(3-bromophenyl)-1,4-dihydrobenzo[4,5]imidazo[1,2-*a*]pyrimidine-3-carbonitrile (3j)

White powder. Yield: 330 mg (90%). ^1^H NMR (500 MHz, DMSO-d_6_): *δ* 8.60 (s, 1H), 7.62 (d, *J* = 8.5 Hz, 1H), 7.55 (d, *J* = 7.5 Hz, 2H), 7.24 (t, *J* = 8 Hz, 3H), 7.12 (t, *J* = 7.5 Hz, 1H), 7.00 (t, *J* = 8 Hz, 1H), 6.85 (s, 2H), 5.24 (s, 1H) ppm. ^13^C {^1^H} NMR (125 MHz, DMSO-d_6_): *δ* 152.04, 149.89, 146.10, 144.05, 131.56, 131.25, 129.76, 129.48, 125.55, 123.96, 122.37, 120.52, 119.57, 116.69, 113.01, 61.63, 53.04 ppm.

### 2.6. General procedure for the synthesis of 1,8-dioxooctahydroxanthenes derivatives 4a–j

In a typical reaction, a mixture of 5,5-dimethyl-1,3-cyclohexanedione (2 mmol), aldehyde (1 mmol) and SMA (1 mol%) were added to a 20 mL vial containing 2–4 mL Isopropyl alcohol (IPA). The mixture was stirred at 70 °C and completion of the reaction was monitored by TLC. Once the precipitate start forming then the reaction is cooled to room temperature for one hour. Then the precipitate was filtered and the final product was obtained as a white crystalline solid, which was further characterized by NMR spectral analysis. The isolated yield, characterization data, and representative NMR spectra of the xanthene derivatives are given in Table 11 and SI.

#### 2.6.1 3,3,6,6-Tetramethyl-9-phenyl-3,4,5,6,7,9-hexahydro-1*H*-xanthene-1,8(2*H*)-dione (4a)

Slaty color powder. Yield: 343 mg (98%). ^1^H NMR (500 MHz, CDCl_3_): *δ* 7.26 (d, *J* = 8 Hz, 2H), 7.18 (t, *J* = 7.5 Hz, 2H), 7.06 (t, *J* = 7.5 Hz, 1H), 4.72 (s, 1H), 2.44 (s, 4H), 2.22–2.12 (m, 4H), 1.07 (s, 6H), 0.96 (s, 6H) ppm. ^13^C {^1^H} NMR (125 MHz, CDCl_3_): *δ* 196.31, 162.39, 144.18, 128.56, 128.09, 126.40, 115.71, 50.81, 40.92, 32.25, 31.89, 29.33, 27.37 ppm. HRMS (ESI^+^) *m*/*z*: [M + H]^+^ calcd for C_23_H_27_O_3_, 351.1960; found, 351.1937.

#### 2.6.2 9-(4-Chlorophenyl)-3,3,6,6-tetramethyl-3,4,5,6,7,9-hexahydro-1*H*-xanthene-1,8(2*H*)-dione (4b)

White crystalline powder. Yield: 361 mg (94%). ^1^H NMR (500 MHz, CDCl_3_): *δ* 7.23 (d, *J* = 6.5 Hz, 2H), 7.18 (d, *J* = 6 Hz, 2H), 4.71 (s, 1H), 2.46 (s, 4H), 2.25–2.15 (m, 4H), 1.10 (s, 6H), 0.99 (s, 6H) ppm. ^13^C {^1^H} NMR (125 MHz, CDCl_3_): *δ* 196.33, 162.42, 142.70, 132, 129.76, 128.19, 115.24, 50.68, 40.83, 32.20, 31.46, 29.27, 27.28 ppm. HRMS (ESI^+^) *m*/*z*: [M + H]^+^ calcd for C_23_H_25_ClO_3_, 385.1570; found, 385.1555.

#### 2.6.3 4-(3,3,6,6-Tetramethyl-1,8-dioxo-2,3,4,5,6,7,8,9-octahydro-1*H*-xanthen-9-yl)benzonitrile (4c)

Yield: Slaty color powder. 338 mg (90%). ^1^H NMR (500 MHz, CDCl_3_): *δ* 7.52 (d, *J* = 6.5 Hz, 2H), 7.42 (d, *J* = 6.5 Hz, 2H), 4.77 (s, 1H), 2.48 (s, 4H), 2.26–2.15 (m, 4H), 1.11 (s, 6H), 0.99 (s, 6H) ppm. ^13^C {^1^H} NMR (125 MHz, CDCl_3_): *δ* 196.26, 162.86, 149.44, 131.98, 129.27, 119.03, 114.61, 110.20, 50.68, 40.83, 32.47, 32.23, 29.24, 27.28 ppm. HRMS (ESI^+^) *m*/*z*: [M + H]^+^ calcd for C_24_H_26_NO_3_, 376.1913; found, 376.1882.

#### 2.6.4 9-(4-Fluorophenyl)-3,3,6,6-tetramethyl-3,4,5,6,7,9-hexahydro-1*H*-xanthene-1,8(2*H*)-dione (4d)

Slaty color powder. Yield: 350 mg (95%). ^1^H NMR (500 MHz, CDCl_3_): *δ* 7.25 (d, *J* = 5 Hz, 2H), 6.90 (t, *J* = 6.5 Hz, 2H), 4.73 (s, 1H), 2.46 (s, 4H), 2.25–2.16 (m, 4H), 1.10 (s, 6H), 0.99 (s, 6H) ppm. ^13^C {^1^H} NMR (125 MHz, CDCl_3_): *δ* 196.40, 162.31, 160.56, 139.94, 129.85, 129.80, 115.51, 114.91, 50.72, 40.85, 32.21, 31.21, 29.27, 27.29 ppm. HRMS (ESI^+^) *m*/*z*: [M + H]^+^ calcd for C_23_H_26_FO_3_, 369.1866; found, 369.1837.

#### 2.6.5 9-(4-Hydroxyphenyl)-3,3,6,6-tetramethyl-3,4,5,6,7,9-hexahydro-1*H*-xanthene-1,8(2*H*)-dione (4e)

Slaty color powder. Yield: 327 mg, (89%). ^1^H NMR (500 MHz, CDCl_3_): *δ* 7.32 (s, 1H), 7.03 (d, *J* = 8 Hz, 2H), 6.52 (d, *J* = 8.5 Hz, 2H), 4.64 (s, 1H), 2.45 (s, 4H), 2.26–2.15 (m, 4H), 1.07 (s, 6H), 0.97 (s, 6H) ppm. ^13^C {^1^H} NMR (125 MHz, CDCl_3_): *δ* 197.51, 162.65, 154.97, 135.42, 129.37, 115.96, 115.36, 50.81, 40.90, 32.33, 31.00, 29.21, 27.46 ppm.

#### 2.6.6 3,3,6,6-Tetramethyl-9-(4-nitrophenyl)-3,4,5,6,7,9-hexahydro-1*H*-xanthene-1,8(2*H*)-dione (4f)

White flex powder. Yield: 356 mg (90%). ^1^H NMR (500 MHz, CDCl_3_): *δ* 8.09 (d, *J* = 6.5 Hz, 2H), 7.48 (d, *J* = 6.5 Hz, 2H), 4.83 (s, 1H), 2.50 (s, 4H), 2.27–2.15 (m, 4H), 1.12 (s, 6H), 0.99 (s, 6H) ppm. ^13^C {^1^H} NMR (125 MHz, CDCl_3_): *δ* 197.35, 162.52, 154.75, 135.51, 129.34, 115.85, 115.25, 50.75, 40.85, 32.28, 30.96, 29.17, 27.27 ppm. HRMS (ESI^+^) *m*/*z*: [M + H]^+^ calcd for C_23_H_26_NO_5_, 396.1811; found, 396.1784.

#### 2.6.7 9-(4-Bromophenyl)-3,3,6,6-tetramethyl-3,4,5,6,7,9-hexahydro-1*H*-xanthene-1,8(2*H*)-dione (4g)

White crystalline powder. Yield: 399 mg, (93%). ^1^H NMR (500 MHz, CDCl_3_): *δ* 7.33 (d, *J* = 6.5 Hz, 2H), 7.17 (d, *J* = 6 Hz, 2H), 4.70 (s, 1H), 2.46 (s, 4H), 2.25–2.15 (m, 4H), 1.10 (s, 6H), 0.99 (s, 6H) ppm. ^13^C {^1^H} NMR (125 MHz, CDCl_3_): *δ* 196.35, 162.45, 143.22, 131.15, 130.18, 120.23, 115.18, 50.69, 40.84, 32.21, 31.56, 29.28, 27.30 ppm. HRMS (ESI^+^) *m*/*z*: [M + H]^+^ calcd for C_23_H_26_BrO_3_, 429.1065; found, 429.1046.

#### 2.6.8 9-(4-Methoxyphenyl)-3,3,6,6-tetramethyl-3,4,5,6,7,9-hexahydro-1*H*-xanthene-1,8(2*H*)-dione (4h)

White crystalline powder. Yield: 314 mg (82%). ^1^H NMR (500 MHz, CDCl_3_): *δ* 7.20 (d, *J* = 6.5 Hz, 2H), 6.75 (d, *J* = 7 Hz, 2H), 4.69 (s, 1H), 3.72 (s, 3H), 2.46 (s, 4H), 2.24–2.15 (m, 4H), 1.09 (s, 6H), 0.99 (s, 6H) ppm. ^13^C {^1^H} NMR (125 MHz, CDCl_3_): *δ* 196.50, 162.09, 157.93, 136.49, 129.30, 115.77, 113.46, 55.10, 50.77, 40.85, 32.20, 30.96, 29.28, 27.34 ppm. HRMS (ESI^+^) *m*/*z*: [M + H]^+^ calcd for C_24_H_29_O_4_, 381.2066; found, 381.2087.

#### 2.6.9 3,3,6,6-Tetramethyl-9-(3,4,5-trimethoxyphenyl)-3,4,5,6,7,9-hexahydro-1*H*-xanthene-1,8(2*H*)-dione (4i)

White powder. Yield: 345 mg (78%). ^1^H NMR (500 MHz, CDCl_3_): *δ* 6.52 (s, 2H), 4.71 (s, 1H), 3.81 (s, 6H), 3.77 (s, 3H), 2.51–2.44 (m, 4H), 2.27–2.20 (m, 4H), 1.11 (s, 6H), 1.03 (s, 6H) ppm. ^13^C {^1^H} NMR (125 MHz, CDCl_3_): *δ* 196.51, 162.37, 152.76, 139.73, 136.52, 115.54, 105.68, 60.68, 56.08, 50.73, 40.89, 32.18, 31.81, 29.37, 27.17 ppm. HRMS (ESI^+^) *m*/*z*: [M + H]^+^ calcd for C_26_H_33_O_6_, 441.2277; found, 411.2278.

#### 2.6.10 9-(2,5-Dimethoxyphenyl)-3,3,6,6-tetramethyl-3,4,5,6,7,9-hexahydro-1*H*-xanthene-1,8(2*H*)-dione (4j)

White powder. Yield: 330 mg (80%). ^1^H NMR (500 MHz, CDCl_3_): *δ* 6.95 (d, *J* = 2.5 Hz, 1H), 6.69–6.63 (m, 4H), 4.83 (s, 1H), 3.75 (d, *J* = 2.5 Hz, 6H), 3.77 (s, 3H), 2.46–2.35 (m, 4H), 2.22–2.12 (m, 4H), 1.09 (s, 6H), 0.96 (s, 6H) ppm. ^13^C {^1^H} NMR (125 MHz, CDCl_3_): *δ* 196.50, 162.80, 152.20, 131.97, 117.49, 113.92, 112.85, 111.75, 55.92, 55.66, 50.88, 41.06, 32.15, 29.44, 26.98 ppm. HRMS (ESI^+^) *m*/*z*: [M + H]^+^ calcd for C_25_H_31_O_5_, 411.2171; found, 411.2151.

## 3. Result and discussion

### 3.1. Synthesis of silicomolybdic acid

Silicomolybdic acid was synthesized by acidifying an aqueous mixture of silicate and molybdate anions, following a modified procedure reported in the literature.^6,7^ The compound was characterized using UV-visible and FT-IR spectroscopy. Its solubility was evaluated in various polar and non-polar solvents, revealing good solubility in all polar solvents, including water.

#### 3.1.1 UV-visible spectral analysis

**Fig. 2:**
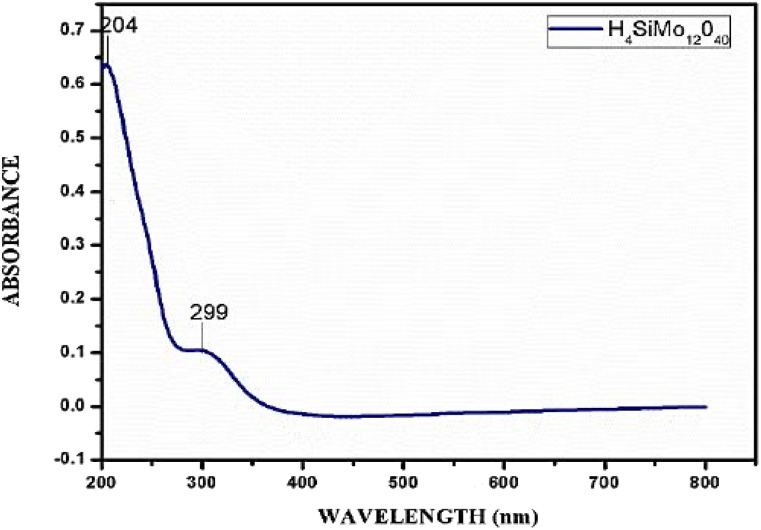
UV-visible spectrum of aqueous solution of SMA.

The UV-visible spectrum of the SMA was recorded in water as depicted in Fig. 2. The acid's electronic spectra revealed a prominent absorption band in the 203–208 nm range that was associated with the π → π* transition. Additionally, an absorption band was also observed at 299 nm, which is associated with the n → π* transition.

#### 3.1.2 FT-IR spectral analysis

**Fig. 3:**
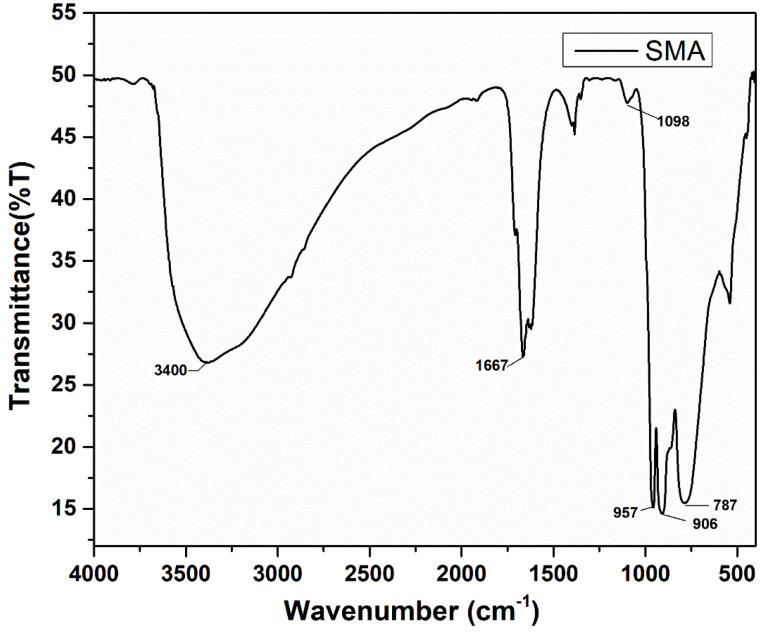
FT-IR spectrum of SMA.

Polyoxometalate compounds are typically characterized using infrared (IR) spectroscopy, which provides valuable information about their structural features. In particular, the IR spectra of polyoxometalates exhibit characteristic absorption bands corresponding to the vibrations of metal–oxygen bonds, such as M=O terminal bonds, M–O–M corner-sharing bonds, and M–O–M edge-sharing bonds. These bands help confirm the presence of the polyoxometalate framework and provide insights into its symmetry and composition. FT-IR spectrum of SMA is given in the Fig. 3. The spectrum shows a strong adsorption band at 1615 cm^−1^ which corresponds to the water peak since it contains a lot of water molecule. A strong band was observed in 958–952 cm^−1^ which is equivalent to the Mo–O terminal bond's asymmetric stretching frequency. A strong band was also observed at 906 cm^−1^. Which is equivalent to the Si–O bond's asymmetric stretching frequency and the band observed at 770–780 cm^−1^ which is equivalent to the asymmetric stretching frequency of Mo–O–Mo bridges.

### 3.2. Knoevenagel condensation reactions

The optimization of the titled compound has been done by tuning the solvent system, temperature, reaction time and catalyst amount and results are summarised in Table 1. The model reaction to yield a 2-benzylidenemalononitrile (1a) product has been chosen as the reaction between 1 mmol of benzaldehyde and 1 mmol of malononitrile in the presence of 1 mol% SMA. To maximize product production, the reaction is carried out in several solvents such as, it is first examined at room temperature in methanol for 5 minutes where we have observed low yield (Table 1 entry 1). Moreover, the utilization of other solvents such as tetrahydrofuran (THF), dichloromethane, ethanol and water also result in the formation of the product with low yield at room temperature for the same reaction time (Table 1 entries 2, 4, 6, 7).

**Table 1:** Selected optimization condition for Knoevenagel condensation^a^

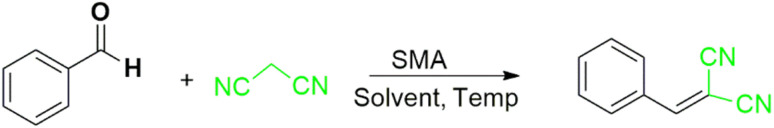					
Entry	Catalyst (mol%)	Solvent	Temp. (°C)	Time^b^ (min)	Yield^c^ (%)
1	1	MeOH	25	5	25
2	1	Water	25	5	15
3	1	ACN	25	5	35
4	1	Ethanol	25	5	25
5	1	Ethanol–water	25	5	40
6	1	THF	25	5	20
7	1	CH_2_Cl_2_	25	5	15
8	1	Ethanol	25	15	50
9	1	ACN	25	15	85
**10**	**1**	**Ethanol–water**	**25**	**15**	**95**
11	1	THF	25	15	50
12	1	Ethanol–water	25	30	95
13	1	Ethanol–water	50	30	95
14	0.5	Ethanol–water	25	30	90
15	2	Ethanol–water	25	30	90
16	—	Ethanol–water	25	60	15
17	—	Ethanol–water	25	180	30
18	—	Ethanol–water	50	240	30

a Reaction condition: benzaldehyde (1 mmol), malononitrile (1 mmol).

b Reaction progress monitored by TLC.

c Isolated yield.

However, we have found product formation with a slightly higher yield in acetonitrile for the same reaction condition (Table 1 entry 3). Furthermore, we have also utilized a combination of two solvents ethanol and water in a 1 : 1 ratio in the same reaction condition and this enhanced the formation of the product (Table 1 entry 5). Upon increasing the reaction time up to 15 minutes nominal increment in the yield has been observed in case of solvents like tetrahydrofuran (THF), dichloromethane, ethanol and water but significant enhancement in the yield has been observed in acetonitrile (Table 1 entry 9) and ethanol–water (1 : 1) system (Table 1 entry 10). Further increasing the reaction period has no improvement in the yield (Table 1 entry 12). Moreover, by increasing the reaction temperature (up to 50 °C) and time the increment in the yield of the product has not been observed (Table 1 entry 13). So far, we have observed reaction proceeds well in acetonitrile as well as ethanol–water system (1 : 1) however, we have neglected acetonitrile considering its potential toxicity and hazardous nature, and hence ethanol–water (1 : 1) system has been considered suitable for this reaction. We have also tried ethanol water mixture in different ratio like 1 : 2, 1 : 3, 2 : 1 respectively, but 1 : 1 ratio is found suitable considering yield of the product. Furthermore, the loading of catalyst in different mol% has also affected the product formation (Table 1 entries 13–15). The reaction is also examined without a catalyst for 3–4 hours at room temperature to 50 °C but we have got only nominal product formation (Table 1 entries 16–18). This finding reveals that the best-optimized condition for the synthesis of product 1a is in the ethanol–water in 1 : 1 at room temperature within 15 minutes with 1 mol% loading of SMA (Table 1 entry 10).

Under the optimized reaction conditions, a series of benzylidene malononitrile derivatives were synthesized using various benzaldehyde derivatives bearing both electron-donating and electron-withdrawing substituents, including 1-naphthaldehyde and 2,4,6-trimethoxybenzaldehyde. The results are summarized in Table 2. In general, good to excellent yields were obtained for most cases. However, reactions involving 1-naphthaldehyde and 2,4,6-trimethoxybenzaldehyde required comparatively longer reaction times. Notably, complete (100%) conversion of the precursors was observed in all cases, with no side products detected, indicating clean and efficient reactions.

**Table 2:** Scope of different aryl aldehyde and malononitrile for the synthesis of benzylidene derivatives^a^

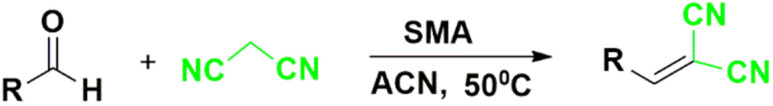						
Entry	R	Time^b^ (min)	Product	Yield^c^ (%)	M.P. (°C) (observed)	M.P. (°C) (reported)
1	–C_6_H_5_	15	1a	95	83–85	81–83 (ref. 40)
2	4-FC_6_H_4_	20	1b	83	124–126	130 (ref. 41)
3	4-ClC_6_H_4_	20	1c	89	158–160	160–162 (ref. 40)
4	Furfural	25	1d	92	72–75	70 (ref. 42)
5	4-MeC_6_H_4_	20	1e	95	134–136	132–134 (ref. 40)
6	2,5-(OMe)_2_C_6_H_3_	25	1f	85	109–111	110–112 (ref. 43)
7	3-MeC_6_H_4_	25	1g	90	75–77	74–75 (ref. 44)
8	4-BrC_6_H_4_	20	1h	90	152–154	153–154 (ref. 40)
9	4-OMeC_6_H_4_	20	1i	92	121–123	122 (ref. 41)
10	4-NO_2_C_6_H_4_	20	1j	86	159–161	158–160 (ref. 43)
11	2,4,5-(OMe)_3_C_6_H_2_	30	1k	88	134–136	136 (ref. 41)
12	1-Naphthayl	50	1l	82	173–175	174–176 (ref. 44)
13	4-CNC_6_H_4_	10	1m	94	152–154	151–153 (ref. 44)

a Reaction condition: aldehyde (1 mmol), malononitrile (1 mmol), SMA (1 mol%).

b Reaction progress monitored by TLC.

c Isolated yield.

**Scheme 1:**
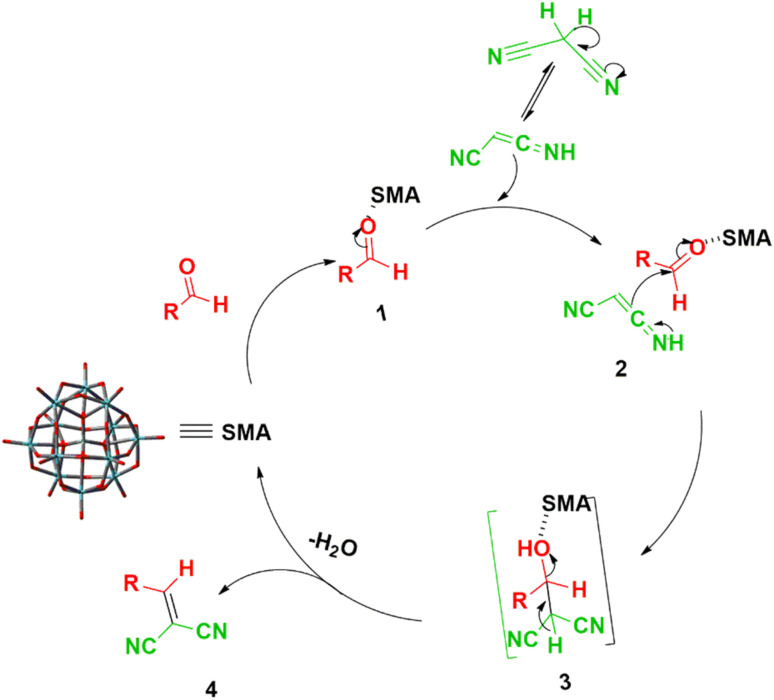
Schematic mechanism^45^ for the catalytic activity of SMA in the synthesis of title compounds (1a–m).

In accordance with the findings, Scheme 1 presents a probable mechanism to illustrate the formation of the title compounds (1a–m) catalyzed by the SMA. It is believed that the reaction happens in steps. Originally, we thought that SMA reduced the electron density around the carbonyl carbon by partially binding the carbonyl oxygen. Then, we thought that the active methylene group in malononitrile was added to the carbonyl carbon to create an intermediate, which, when a water molecule was removed, produced the desired product.^45^

**Fig. 4:**
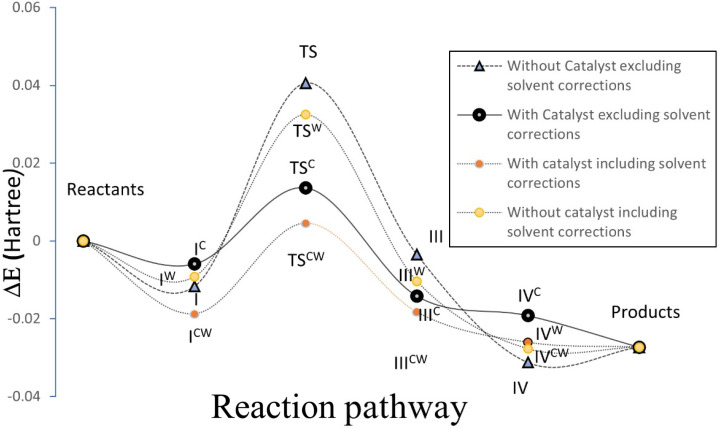
The energy profile diagram of the reaction showing relative energies (in Hartree) at different stage of reaction with (I^C^) and without catalyst (I) and also including the effect of solvent (indicated by superscript W) during the course of reaction.

**Fig. 5:**
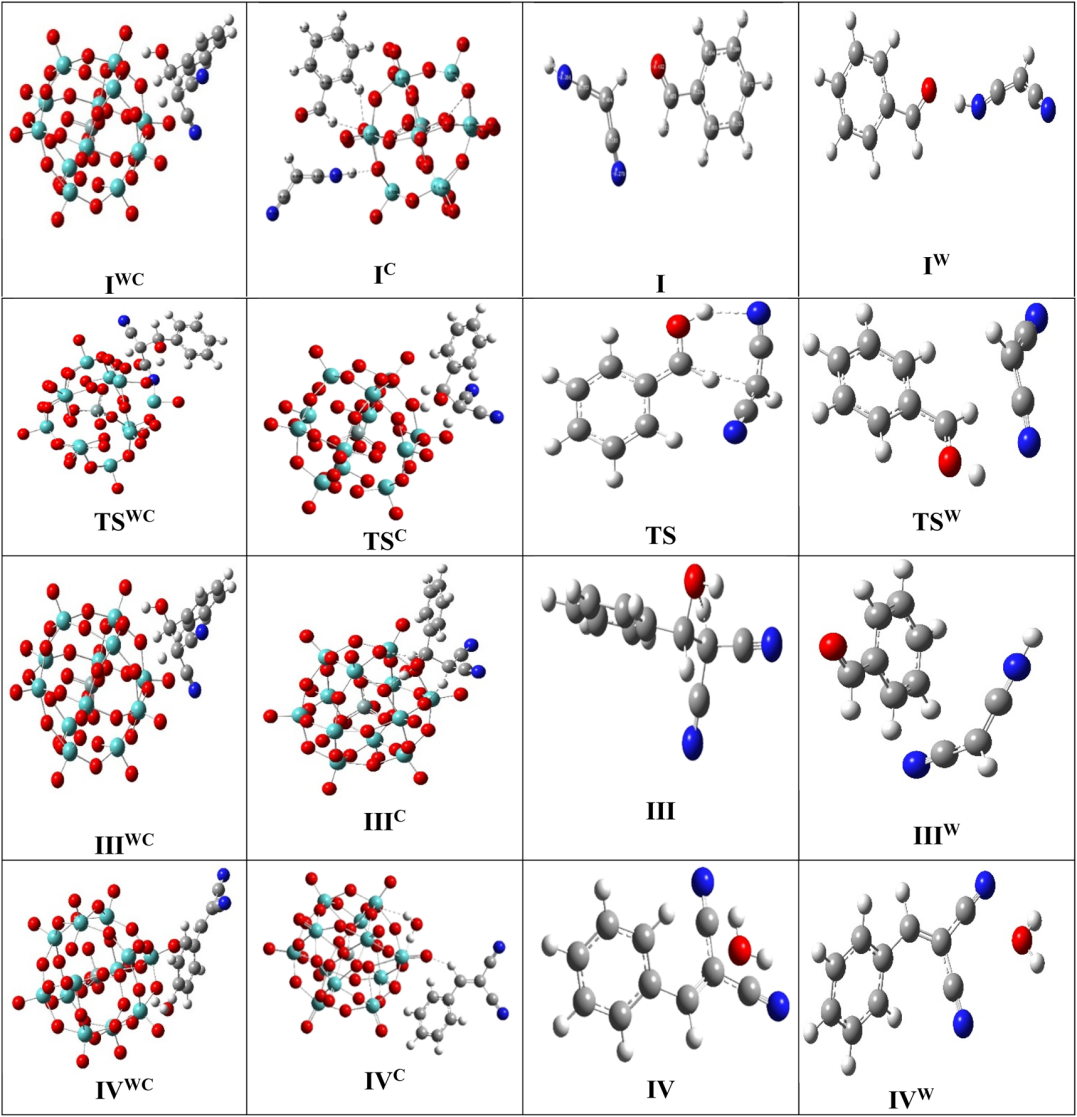
The optimised structure of various intermediate states with catalyst (I^C^) and without catalyst (I) and with and without considering solvent effect (without and with superscript W) during the course of reaction as presented in Fig. 4.

For a comprehensive examination of the reaction mechanism, a series of theoretical calculations were conducted using Gaussian-16 software.^39^ The semi-empirical parameterization method 6 (PM6) method was employed in our theoretical investigation to study the reactions with and without the catalyst. The calculations were also carried out separately in gas phase and also considering the effect of solvent (water). The solvent effect was investigated using the self-consistent reaction field (SCRF) method with polarizable continuum method (PCM) using water as the solvent. Vibrational frequency calculations at this semi-imperial level PM6 were also carried out to identify the transition states with one negative frequency and stable minimum without any negative frequency. The computational energy profile diagram is plotted in Fig. 4. It was observed that the system stabilized when the reactants mixed. The pre-reaction mixture is denoted as I and I^C^ (representing structures without and with the catalyst, respectively, excluding solvent effects), and as I_S_ and I_S_^C^ (without and with the catalyst, respectively, considering solvent effects) in Fig. 4. These structures are illustrated in Fig. 5, and the Cartesian coordinates for all optimized structures shown in Fig. 5 are provided in SI Table S1. In the next step of the reaction, the formation of a transition state was identified. The transition state is characterized by the presence of a single negative frequency. The transition states depicted as TS, TS^C^, TS^W^ and TS^WC^, respectively, are also presented in Fig. 4. Here it could be identified that the height of the reaction barrier (*e.g.* transition state) is reduced by ∼0.027 hartree (TS and TS^C^) in presence of catalyst when the effect of solvent was not considered. It was also observed that even in absence of catalyst, the solvent water reduces the transition state by ∼0.0081 hartree and thereby can influence the reaction process as observed in ref. 45. On including the solvent effect by the hight of the transition state for the catalytic reaction is found to decrease by ∼0.031 hartree.

The reduction in energy barrier elucidates that the catalyst, when binding both reactants, facilitates the reactions. The reason behind the reductions in the energy barrier can be rationalized by analyzing the pre-reaction mixture complex depicted in Fig. 4. Additionally, it was noted that the catalyst enhances the formation of C–C bonds by redistributing electronic clouds. In the presence of the catalyst, the C centre of benzaldehyde exhibits increased positivity, while the C centre in malononitrile becomes more electron-dense.

Assessing the viability of financial sustainability and promoting environmentally sustainable development are key aspects of our study, with a primary focus on evaluating the reuse and recycling potential of the catalyst. As mentioned in experimental procedure, after completion of reaction, product precipitates in the solution which was collected upon simple filtration and the catalyst remain in the filtrate which was recovered by evaporation and used for the next cycle. The reusability of the SMA was verified by the optimized reaction condition of 1a, after the completion of the reaction, the recovered catalyst was utilized for the subsequent reaction. This process was repeated for five cycles and the excellent yield of product (1a) was obtained in each cycle.

To showcase the practical synthetic utility of SMA, a gram-scale experiment was carried out for the synthesis of 1a (Table 2, entry 1). In this reaction, benzaldehyde (0.816 mL, 8 mmol), malononitrile (0.448 mL, 8 mmol) and SMA (1 mol%) were combined in ethanol–water (5 : 5 mL), yielding 1.07 g of product 1a with an isolated yield of 87%.

Furthermore, the catalytic performance of SMA for the synthesis of compound 1a by choosing benzaldehyde and malononitrile as model substrates has been studied comparatively with other HPA catalyst reported in literature, as summarized in Table 3. While many of these reported methods offer good to excellent yields under specific conditions, they often exhibit certain drawbacks. Common limitations include the requirement for high catalyst loading, prolonged reaction times, and the need for elevated temperatures, all of which can reduce the overall efficiency and practicality of the process. As evident from Table 3, SMA demonstrates superior catalytic efficiency in the synthesis of 1a when compared with other catalysts. Notably, the reaction proceeds efficiently at room temperature with a significantly shorter reaction time and lower catalyst loading. Additional benefits of SMA include its ease of separation from the reaction mixture, operation in a nontoxic solvent system. For instance, catalyst such as H_3_PMo_12_O_40_,^46^ H_3_PW_12_O_40_,^46^ H_4_PMo_11_VO_40_ (ref. 47) and H_5_PMo_10_V_2_O_40_,^47^ require elevated temperatures and slightly higher catalyst loadings compared to SMA. Despite these conditions, SMA provides a higher yield of compound 1a. Although these HPAs are effective under solvent-free conditions, their overall product yields are lower than those achieved with SMA. Similarly, the use of K_11_H[P_2_W_18_O_68_(HOSn^IV^OH)_3_]^48^ involves longer reaction times and results in lower yields, further highlighting the superior catalytic performance of SMA in the synthesis of compound 1a.

**Table 3:** Comparison of SMA's catalytic efficiency for the synthesis of 1a with that of other catalytic systems

Catalyst	Amount of catalyst	Solvent	Yield (%)	Time (min)	Temperature (°C)
H_3_PMo_12_O_40_ (ref. 46)	2 mol%	—	65	30	70
H_3_PW_12_O_40_ (ref. 46)	2 mol%	—	55	30	70
H_4_PMo_11_VO_40_ (ref. 47)	2 mol%	—	86	45	70
H_5_PMo_10_V_2_O_40_ (ref. 47)	2 mol%	—	88	45	70
K_11_H[P_2_W_18_O_68_(HOSn^IV^OH)_3_]^48^	0.01 g	Water	90	60	rt
**SMA (this work)**	**1 mol%**	**Ethanol:water**	**95**	**15**	**rt**

### 3.3. Chromene derivatives

The optimization of the titled compound has been done by tuning the solvent system, temperature, reaction time and catalyst amount and outcomes are summarised in Table 4. The model reaction to yield 2-amino-5-oxo-4-phenyl-5,6,7,8-tetrahydro-4*H*-chromene-3-carbonitrile (2c) product has been chosen as the reaction between 1 mmol of benzaldehyde, 1 mmol of malononitrile and 1 mmol cyclohexane-1,3-dione in the presence of 1 mol% SMA. Several solvents are used during the reaction to optimize the result yield such as, the reaction is first carried out in methanol for 5 minutes at room temperature, during which we have observed a low yield (Table 4 entry 1). Moreover, the utilization of other solvents such as tetrahydrofuran (THF), dichloromethane and water also result in the formation of the product with low yield at room temperature for 5 minutes (Table 4 entries 2, 6, 7). However, we have found product formation with a significantly high yield in the presence of acetonitrile and ethanol for the same reaction condition (Table 4 entries 3, 4). We have also utilized a combination of two solvents ethanol and water in a 1 : 1 ratio in the above reaction condition (Table 4 entry 5) but this does not enhance the formation of the product. Further, the period of the reaction has been increased by 10 minutes and no significant increment in the yield has been observed for any solvent used here (Table 4 entries 8–13). So far, we have observed that reaction proceeds well in acetonitrile as well as ethanol however, we have neglected the solvent acetonitrile considering its potential toxicity and hazardous nature, and hence ethanol has been considered suitable for this reaction. Moreover, the temperature of the reaction is raised to 50 °C and duration of the reaction is also increased in order to check its effect on the yield, but no enhancement in the yield is observed (Table 4 entry 14). Furthermore, loading of catalyst in different mol% has also affected the product formation (Table 4 entries 15, 16). The reaction is also examined without a catalyst for 3–4 hours at room temperature to 50 °C but we have got only nominal product formation (Table 4 entries 17, 18). This finding reveals that the best-optimized condition for the synthesis of product 2c is in the solvent ethanol at room temperature within 5 minutes with 1 mol% loading of SMA (Table 4 entry 4).

**Table 4:** Selected optimization conditions for the synthesis of chromene derivatives^a^

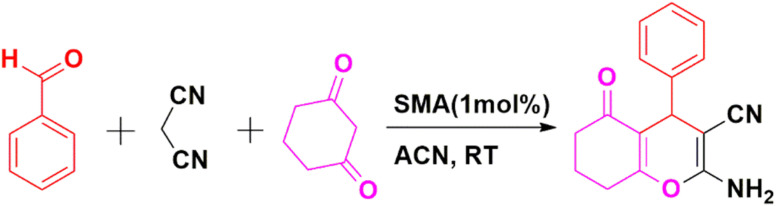					
Entry	Catalyst (mol%)	Solvent	Temp. (°C)	Time^b^ (min)	Yield^c^ (%)
1	1	MeOH	25	5	40
2	1	Water	25	5	25
3	1	ACN	25	5	90
**4**	**1**	**EtOH**	**25**	**5**	**92**
5	1	Ethanol–water	25	5	60
6	1	THF	25	5	45
7	1	CH_2_Cl_2_	25	5	30
8	1	MeOH	25	10	40
9	1	ACN	25	10	80
10	1	EtOH	25	10	92
11	1	Ethanol–water	25	10	45
12	1	THF	25	10	50
13	1	CH_2_Cl_2_	25	10	40
14	1	EtOH	50	30	92
15	0.5	EtOH	25	30	85
16	2	EtOH	25	30	80
17	—	EtOH	25	180	30
18	—	EtOH	50	240	30

a Reaction condition: benzaldehyde (1 mmol), malononitrile (1 mmol).

b Reaction progress monitored by TLC.

c Isolated yield.

In the next step, the activity of SMA towards various aromatic aldehydes with different functionalities such as Cl, Br, NO_2_, CH_3_, OH, OMe, *etc.* (Table 5 entries 2–10) to prepare 2-amino-5-oxo-4-substituted-5,6,7,8-tetrahydro-4*H*-chromene-3-carbonitrile derivatives (2a–j) has further been investigated using optimized condition and all have been proven to be suitable under the reaction condition. In fact, several derivatives of Chromene have been obtained in high to excellent yields using one pot MCR of dimedone or cyclohexane-1,3-dione, aromatic aldehydes, and malononitrile under optimal reaction conditions. As indicated by the outcomes in Table 5, a range of aromatic and heteroaromatic aldehydes, encompassing electron-donating or electron-withdrawing groups have been employed under optimized circumstances for the reaction to yield the intended outcomes (2a–j) in good to exceptional yields. In each examined instance, the reaction progressed uninterruptedly and the anticipated products were acquired without the need for an intermediate after the specified reaction time as outlined in Table 5. The identification of the obtained products has been accomplished through referring to their melting points and spectroscopic information along with the published data for the validated samples.

**Table 5:** SMA catalysed multicomponent synthesis of 2a–j^a^

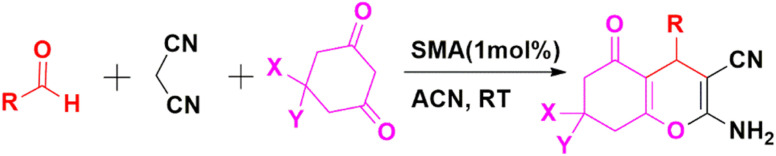							
Entry	R	X, Y	Time^b^ (min)	Product	Yield^c^ (%)	M.P. (°C) (observed)	M.P. (°C) (reported)
1	4-ClC_6_H_4_	H, H	10	2a	85	221–225	222–224 (ref. 49)
2	4-NO_2_C_6_H_4_	H, H	15	2b	86	234–236	236–238 (ref. 49)
3	–C_6_H_5_	H, H	10	2c	92	238–241	240–242 (ref. 49)
4	3-MeC_6_H_4_	H, H	14	2d	89	225–227	223–225 (ref. 49)
5	4-OMeC_6_H_4_	H, H	15	2e	89	191–193	190–192 (ref. 49)
6	4-OHC_6_H_4_	CH_3_, CH_3_	10	2f	88	214–218	215–217 (ref. 49)
7	–C_6_H_5_	CH_3_, CH_3_	15	2g	93	235–237	234–236 (ref. 49)
8	4-ClC_6_H_4_	CH_3_, CH_3_	20	2h	84	237–240	236–238 (ref. 49)
9	4-NO_2_C_6_H_4_	CH_3_, CH_3_	15	2i	87	180–183	182–184 (ref. 49)
10	4-CNC_6_H_4_	CH_3_, CH_3_	15	2j	88	228–230	230–232 (ref. 50)

a Reaction condition: aldehyde (1 mmol), malononitrile (1 mmol), dimedone (1 mmol) and SMA (1 mol%).

b Reaction progress monitored by TLC.

c Isolated yield.

**Scheme 2:**
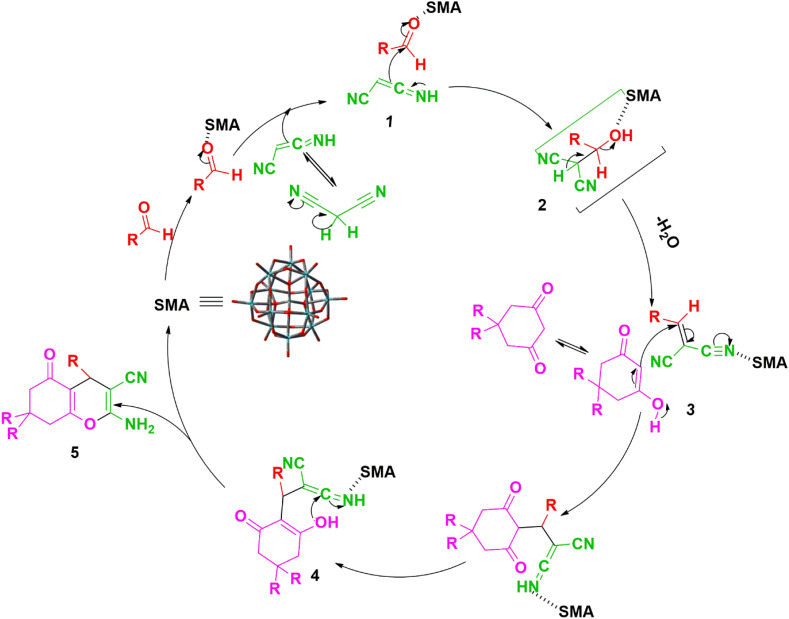
Schematic mechanism^29^ for the catalytic activity of SMA in the synthesis of title compounds (2a–j).

In accordance with the findings, Scheme 2 presents a probable mechanism to illustrate the formation of the title compounds (2a–j) catalyzed by the SMA, we believe that the reaction happens in a stepwise manner. Initially, SMA enhanced the electrophilicity on the carbonyl carbon atom of the aromatic aldehyde, then the nucleophilic attack of the active methylene group of malononitrile 1 to the electron-deficient carbon of activated aldehyde takes place. Which upon condensation forms intermediate 3 by releasing a water molecule *via* Knoevenagel condensation. Again, the nitrile group of intermediate 3 is activated by SMA, then activated methylene of dimedone reacts with intermediate 3*via* conjugate Michael addition to results intermediate 4, which produces the intended product 2-amino-4*H*-chromene-3-carbonitriles 5*via* intramolecular cyclization.^29^

A key benefit of the catalyst, which enhances its value for commercial use, is its reusability, with a primary focus on evaluating its potential for reuse and recycling. As mentioned in experimental procedure, after completion of reaction, product precipitates in the solution which has was collected upon simple filtration and the catalyst remain in the filtrate which was recovered by evaporation and used for the next cycle. The reusability of the SMA was verified by the optimized reaction condition of 1a, after the completion of the reaction, the recovered catalyst was utilized for the subsequent reaction. This process was repeated for five cycles and the excellent yield of product (2c) was obtained in each cycle.

To showcase the practical synthetic utility of SMA, a gram-scale experiment was carried out for the synthesis of 2c (Table 5, entry 3). In this reaction benzaldehyde (0.510 mL, 5 mmol), malononitrile (0.280 mL, 5 mmol), cyclohexane-1,3-dione (0.560 g, 5 mmol), and SMA (1 mol%) were reacted in ethanol (10 mL), producing 1.05 g of product 2c with an isolated yield of 78%.

Several methods have been reported for the synthesis of compound 2g, which typically involve a one-pot, three-component condensation reaction of aromatic aldehydes, malononitrile and dimedone in the presence of various catalysts. A comparative analysis of the catalytic performance of the proposed SMA with some recently reported catalysts is presented in Table 6. While many of these reported methods offer good to excellent yields under specific conditions, they often exhibit certain drawbacks. Common limitations include the requirement for high catalyst loading, prolonged reaction times, and the need for elevated temperatures, all of which can reduce the overall efficiency and practicality of the process. As evident from Table 6, SMA demonstrates superior catalytic efficiency in the synthesis of 2g when compared with other catalysts. Notably, the reaction proceeds efficiently at room temperature with a significantly shorter reaction time and lower catalyst loading. Additional benefits of SMA include its ease of separation from the reaction mixture, operation in a nontoxic solvent system, and excellent reusability over multiple cycles without significant loss in activity. Moreover, SMA is a stable, solid acid catalyst that is not only straightforward to synthesize in the laboratory but also commercially available, making it an attractive option for both academic research and industrial applications.

**Table 6:** Comparison of SMA's catalytic efficiency for the synthesis of 2g with that of other catalytic systems

Catalyst	Amount of catalyst	Yield (%)	Solvent	Time (min)	Temperature (°C)
Yb(PFO)_3_ (ref. 51)	5 mol%	90	EtOH	300	60
TBAF^52^	10 mol%	97	Water	30	Reflux
Re(PFO)_3_ (ref. 53)	5 mol%	90	EtOH	300	60
Yb(OTf)_3_ (ref. 54)	5 mol%	78	EtOH	300	60
Nano-TiO_2_–SO_3_H^29^	0.2 mol%	90	—	30	80
**SMA (this work)**	**1 mol%**	**93**	**EtOH**	**15**	**rt**

### 3.4. Imidazopyrimidine derivatives

The optimization of the titled compound has been done by tuning the solvent system, temperature, reaction time and catalyst amount and outcomes are summarised in Table 7. The model reaction to yield 2-amino-4-phenyl-1,4-dihydrobenzo[4,5]imidazo[1,2-*a*]pyrimidine-3-carbonitrile (3a) product has been chosen as the reaction between 1 mmol of benzaldehyde, 1 mmol of malononitrile and 1 mmol 2-aminobenzimidazole in the presence of 1 mol% SMA. Several solvents are used during the reaction to optimize the product yield such as, the reaction is first carried out in methanol for 10 minutes at room temperature, during which we have observed a low yield (Table 7 entry 1). Moreover, the utilization of other solvents such as tetrahydrofuran (THF), dichloromethane and acetonitrile also results in the formation of the product with low yield at room temperature for 10 minutes (Table 7 entries 3, 5, 6). Even the combination of two solvent acetonitrile and toluene has not worked well for the same reaction condition as no increment in the yield has been observed (Table 7 entry 4). However, utilizing water as a solvent has significantly enhanced product yield (Table 7 entry 2). Now, duration of the reaction has been extended from 10 minutes to 45 minutes and the temperature of the reaction has been raised to 75 °C as well, again the yield has been examined in various solvent but we have observed only marginal improvement in the yield (Table 7 entries 7–12). So far, water has worked well as solvent for this reaction using SMA, hence catalyst amount has been optimized in water at room temperature varying catalyst amount and we have observed 1 mol% loading of SMA provide best yield for this reaction (Table 7 entries 12–14). Control experiments has been conducted in the absence of catalyst for 3–4 hours at room temperature and 75 °C yielded only trace amounts of product (Table 7, entries 15 and 16). These results demonstrate that the optimal conditions for synthesizing compound 3a involve using water as the solvent at room temperature for 10 minutes with 1 mol% SMA as the catalyst (Table 7, entry 2).

**Table 7:** Selected optimization conditions for imidazopyrimidine derivative reaction^a^

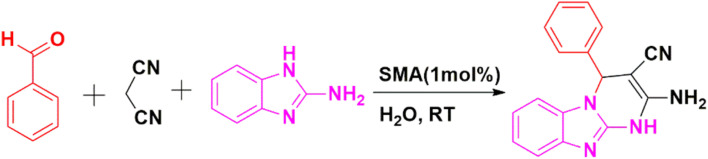					
Entry	Catalyst (mol%)	Solvent	Temp. (°C)	Time^b^ (min)	Yield^c^ (%)
1	1	MeOH	25	10	30
**2**	**1**	**Water**	**25**	**10**	**90**
3	1	ACN	25	10	30
4	1	ACN + toluene	25	10	25
5	1	THF	25	10	35
6	1	CH_2_Cl_2_	25	10	25
7	1	MeOH	75	45	35
8	1	ACN	75	45	50
9	1	ACN + toluene	75	45	35
10	1	THF	75	45	40
11	1	CH_2_Cl_2_	75	45	38
12	1	Water	75	45	90
13	0.5	Water	25	10	55
14	2	Water	25	10	60
15	—	Water	25	180	14
16	—	Water	75	240	20

a Reaction condition: benzaldehyde (1 mmol), malononitrile (1 mmol), 2-aminobenzimidazole (1 mmol).

b Reaction progress monitored by TLC.

c Isolated yield.

In the next step, the activity of SMA towards various aromatic aldehydes with different functionalities such as Cl, Br, NO_2_, CH_3_, OH, OMe, *etc.* (Table 8 entries 2–10) in the preparation of 2-amino-4-substituted-1,4-dihydrobenzo[4,5]imidazolo[1,2-*a*]pyrimidine-3-carbonitrile derivatives (3a–j) has further been investigated using optimized condition and all have been proven to be suitable under the reaction condition. In fact, several derivatives of imidazopyrimidine have been obtained in high to excellent yields with one pot MCR of 2-aminobenzimidazole, aromatic aldehydes, and malononitrile using optimal reaction conditions. As indicated by the outcomes in Table 8, a range of aromatic and heteroaromatic aldehydes, encompassing electron-donating or electron-withdrawing groups have been employed under optimized circumstances for the reaction to yield the intended outcomes (3a–j) in good to exceptional yields. In each examined instance, the reaction progressed uninterruptedly and the anticipated products have been acquired without the need for an intermediate after the specified reaction time as outlined in Table 8. The identification of the obtained products has been accomplished through referring their melting points and spectroscopic information along with the published data for the validated samples.

**Table 8:** SMA catalysed multicomponent synthesis of 3a–j^a^

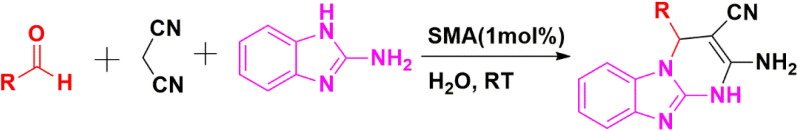						
Entry	R	Time^b^ (min)	Product	Yield^c^ (%)	M.P. (°C) (observed)	M.P. (°C) (reported)
1	–C_6_H_5_	10	3a	90	235–237	236–238 (ref. 35)
2	4-ClC_6_H_4_	15	3b	94	234–236	234–236 (ref. 35)
3	4-OHC_6_H_4_	16	3c	83	210–213	210–213 (ref. 36)
4	4-FC_6_H_4_	14	3d	89	253–255	254–256 (ref. 35)
5	4-BrC_6_H_4_	15	3e	92	244–246	243–245 (ref. 35)
6	4-MeC_6_H_4_	10	3f	80	224–226	240–242 (ref. 35)
7	2-OMe, 5-OMeC_6_H_3_	15	3g	82	221–223	223 (ref. 37)
8	3-OMe, 4-OMe, 5-OMeC_6_H_2_	20	3h	81	229–231	230–232 (ref. 34)
9	2-ClC_6_H_4_	12	3i	91	237–239	237–239 (ref. 35)
10	3-BrC_6_H_4_	14	3j	90	241–243	242–244 (ref. 35)

a Reaction condition: aldehyde (1 mmol), malononitrile (1 mmol), 2-aminobenzimidazole (1 mmol) and SMA (1 mol%).

b Reaction progress monitored by TLC.

c Isolated yield.

**Scheme 3:**
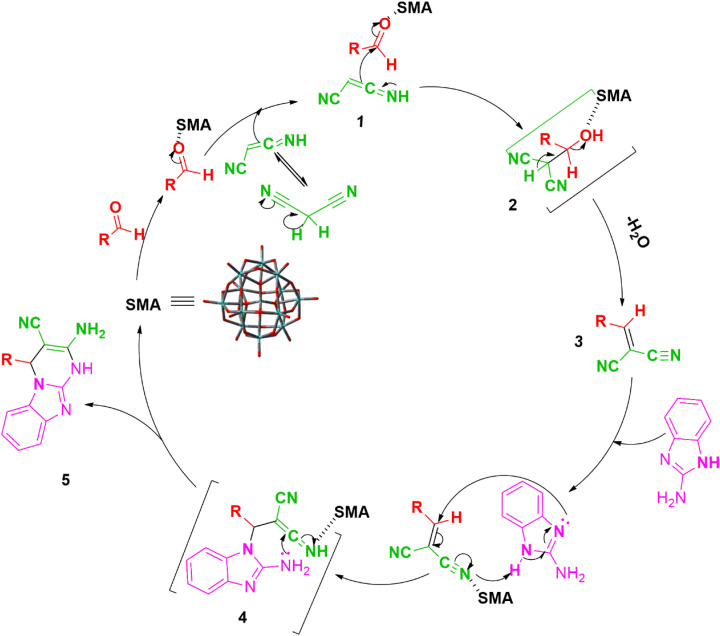
Schematic mechanism^34^ for the catalytic activity of SMA in the synthesis of title compounds (3a–j).

In accordance with the findings, Scheme 3 presents a probable mechanism to illustrate the stepwise formation of the title compounds (3a–j) catalyzed by the SMA, we believe that the reaction happens in a stepwise manner. First of all, we assume that the SMA enhances the electrophilicity of the carbon atom of aldehyde 2 which undergoes rapid nucleophilic attack by the tautomeric form of malononitrile 1 to results the intermediate 3(arylidinemalononitrile) by releasing a water molecule through Knoevenagel condensation method. Further, it experiences an instant nucleophilic attack of 2-aminobenzimidazole on the conjugated C=C bond of 3, resulting in the formation of intermediate 4 by Michael addition. Ultimately, the intramolecular concerted cyclization followed by tautomerization of intermediate 4 results in the formation of the title compounds (3a–j).^34^

To showcase the practical synthetic utility of SMA, a gram-scale experiment was carried out for the synthesis of 3a (Table 8, entry 1). In this reaction, benzaldehyde (0.510 mL, 5 mmol), malononitrile (0.280 mL, 5 mmol), 2-aminobenzimidazole (0.667 g, 5 mmol), and SMA (1 mol%) were combined in water (10 mL), yielding 1.03 g of product 3a with an isolated yield of 72%.

Various synthetic strategies have been reported for the preparation of compound 3b, most of which involve a one-pot, three-component condensation reaction between aromatic aldehydes, malononitrile, and 2-aminobenzimidazole in the presence of different catalysts. A comparative evaluation of the catalytic efficiency of the proposed SMA with other recently reported catalysts is summarized in Table 9. Although several of these methods yield good to excellent product outcomes under optimized conditions, many suffer from significant drawbacks such as high catalyst loading, toxic solvent, extended reaction durations, and the requirement for elevated temperatures, thereby limiting their scalability and operational simplicity. In contrast, the use of SMA offers several notable advantages. As shown in Table 9, SMA exhibits superior catalytic activity, enabling the synthesis of 3b under mild conditions specifically, at room temperature with a markedly shorter reaction time and lower catalyst dosage. Furthermore, the reaction proceeds in an environmentally benign solvent system, namely water, enhancing the green chemistry profile of the process. These attributes, combined with the ease of catalyst handling, potential for reusability, and commercial availability, position SMA as an efficient and sustainable alternative for the synthesis of 3b.

**Table 9:** Comparison of SMA's catalytic efficiency for the synthesis of 3b with that of other catalytic systems

Catalyst	Amount of catalyst	Yield (%)	Solvent	Time (min)	Temperature (°C)
[C_4_(DABCO)_2_](CuCl_4_)^54^	40 mg	95	—	7	110
*p*-TSA^34^	10 mol%	93	—	30	80
MgO^55^	5 mol%	75	MeCN	45	—
Fe_3_O_4_@IM^56^	30 mg	95	EtOH	15	Reflux
ZnFe_2_O_4_ (ref. 36)	10 mol%	96	MeOH	90	70
**SMA (this work)**	**1 mol%**	**94**	**Water**	**15**	**rt**

### 3.5. Xanthene derivatives

The optimization of the titled compound has been done by tuning the solvent system, temperature, reaction time and catalyst amount and the results are summarised in Table 10. The model reaction to yield 3,3,6,6-tetramethyl-9-phenyl-3,4,5,6,7,9-hexahydro-1*H*-xanthene-1,8(2*H*)-dione (4a) product has been chosen as the reaction between 1 mmol of benzaldehyde and 2 mmol of dimedone with 1 mol% of SMA. To maximize product production, the reaction is carried out in several solvents. The reaction is first carried out at 70 °C in methanol for one hour where we have observed low yield (Table 10 entry 1).

**Table 10:** Selected optimization condition for xanthene derivatives^a^

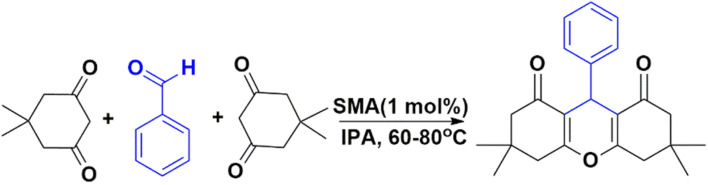					
Entry	Catalyst (mol%)	Solvent	Temp. (°C)	Time^b^ (min)	Yield^c^ (%)
1	1	MeOH	70	60	35
2	1	Water	70	60	20
3	1	ACN	70	60	50
**4**	**1**	**IPA**	**70**	**60**	**98**
5	1	THF	70	60	40
6	1	CH_2_Cl_2_	70	60	30
7	1	MeOH	80	80	40
8	1	ACN	80	80	60
9	1	IPA	80	80	98
10	1	THF	80	80	50
11	1	CH_2_Cl_2_	80	80	40
12	1	IPA	25	90	—
13	0.5	IPA	70	60	60
14	2	IPA	70	60	85
15	—	IPA	70	180	10

a Reaction condition: benzaldehyde (1 mmol), dimedone (2 mmol).

b Reaction progress monitored by TLC.

c Isolated yield.

Moreover, the utilization of other solvents such as water, acetonitrile, tetrahydrofuran (THF) and dichloromethane also resulted in the formation of the product with low yield at 70 °C for one hour (Table 10 entries 2, 3, 5, 6). However, we have found product formation with a significantly high yield in the presence of isopropyl alcohol (IPA) for the same reaction condition (Table 10 entry 4). Further, the temperature of the reaction has been increased to 80 °C and the duration of the reaction has also been increased from 60 to 80 minutes however, the nominal increment in the yield of the product has been observed in almost all solvents used above (Table 10 entries 7, 8, 10, 11) and in case of IPA, no change in the yield has been observed (Table 10 entry 9). This reaction has also been examined at room temperature in IPA but no formation of product has been observed (Table 10 entry 12). Furthermore, the loading of catalyst in different mol% has also affected the product formation (Table 10 entries 13, 14). Besides, the reaction is also examined without catalyst for 3 hours at 70 °C but we have got only nominal product formation (Table 10 entry 15). So far, we have found from the above findings that the best optimized condition for the synthesis of product 4a is in the solvent IPA at 70 °C within 10 min with 1 mol% loading of SMA (Table 10 entry 4).

In the next step, the activity of SMA towards various aromatic aldehydes with different functionalities such as Cl, Br, NO_2_, CH_3_, OH, OMe, *etc.* (Table 11 entries 2–9) in the preparation of several derivatives of 1,8-dioxo-octahydroxanthene (4a–i) has further been investigated using optimized condition and all have been proven to be suitable under the reaction condition. In fact, several derivatives of xanthene have been obtained in high to excellent yields using dimedone and aromatic aldehydes under optimal reaction conditions. As indicated by the outcomes in Table 11, a range of aromatic and heteroaromatic aldehydes, encompassing electron-donating or electron-withdrawing groups have been employed under optimized circumstances for the reaction to yield the intended outcomes (4a–i) in good to exceptional yields. In each examined instance, the reaction progressed uninterruptedly and the anticipated products have been acquired without the need for an intermediate after the specified reaction time as outlined in Table 11. The identification of the obtained products has been accomplished through referring to their melting points and spectroscopic information along with the published data for the validated samples.

**Table 11:** SMA catalysed synthesis of 4a–i^a^

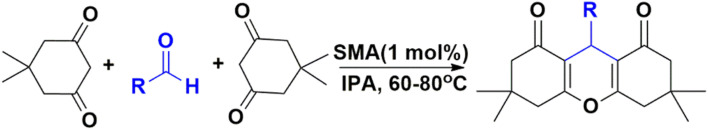						
Entry	R	Time^b^ (min)	Product	Yield^c^ (%)	M.P (°C) (observed)	M.P (°C) (reported)
1	–C_6_H_5_	80	4a	98	203–205	203–204 (ref. 26)
2	4-ClC_6_H_4_	85	4b	94	233–235	230–232 (ref. 27)
3	4-CNC_6_H_4_	90	4c	89	215–217	217–218 (ref. 27)
4	4-FC_6_H_4_	80	4d	95	226–228	227–228 (ref. 27)
5	4-OHC_6_H_4_	120	4e	89	246–248	245–246 (ref. 26)
6	4-NO_2_C_6_H_4_	85	4f	90	224–226	224–226 (ref. 26)
7	4-BrC_6_H_4_	90	4g	93	241–243	240–241 (ref. 27)
8	4-OMeC_6_H_4_	180	4h	82	243–245	241–243 (ref. 27)
9	3-OMe, 4-OMe, 5-OMeC_6_H_2_	240	4i	78	185–187	186–188 (ref. 57)
10	2-OMe, 5-OMeC_6_H_2_	80	4j	80	173–175	172–174 (ref. 58)

a Reaction condition: aldehyde (1 mmol), dimedone (2 mmol) at 70 °C.

b Reaction progress monitored by TLC.

c Isolated yield.

**Scheme 4:**
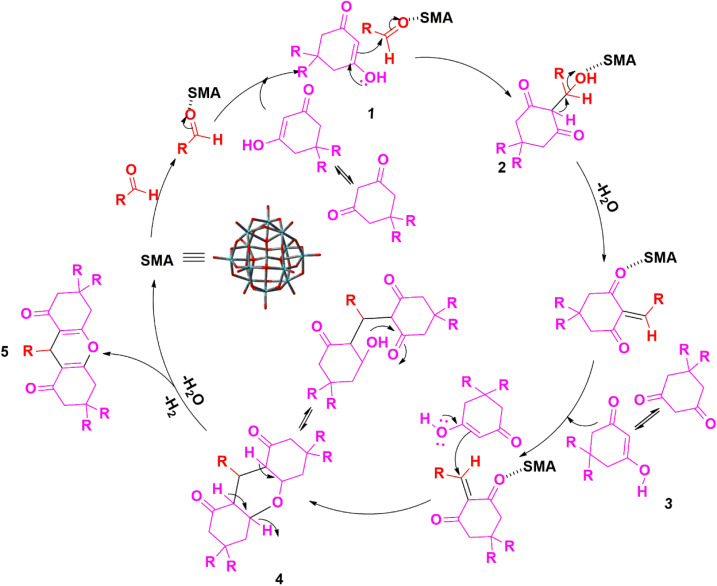
Schematic mechanism^29^ for the catalytic activity of SMA in the synthesis of title compounds (4a–i).

In accordance with the findings, Scheme 4 presents a most probable mechanism to illustrate the stepwise formation of the title compounds (4a–i) catalyzed by the SMA, we believe that the reaction happens in a stepwise manner. Initially, the carbonyl group of aromatic aldehyde 2 is activated by SMA, and then the nucleophilic attack of the active methylene group of one molecule of dimedone 1 to the electron-deficient carbon of activated aldehyde takes place. Which upon condensation forms intermediate 4 by releasing a water molecule *via* Knoevenagel condensation. Again, the carbonyl group of intermediate 4 is activated by SMA, then activated methylene of the second molecule of dimedone reacts with intermediate 4*via* conjugate Michael addition to results intermediate 6, which undergoes intramolecular cyclodehydration to produce the desired product 1,8-dioxo-octahydroxanthene 7.^29^

**Scheme 5:**
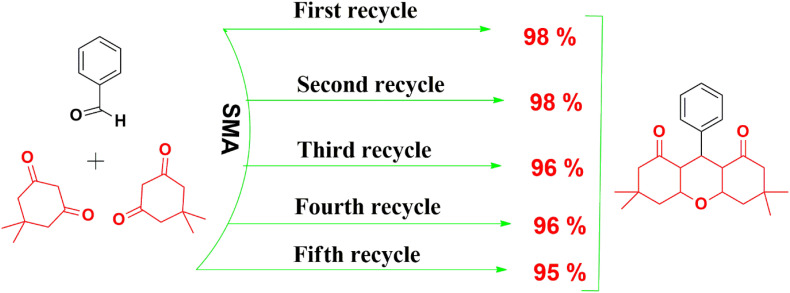
Reusability of SMA.

The catalyst's ability to be recycled and reused is a crucial aspect from the perspective of green chemistry, as it minimizes waste generation and reduces overall production cost, with a primary focus on evaluating the reuse and recycling potential of the catalyst. The reusability of the SMA was verified by the optimized reaction condition of 4a. As mentioned in the experimental section, after the completion of the reaction, the product obtained as precipitate and catalyst remains in solvent. The product was filtered and the filtrate containing catalyst was utilized for the subsequent reaction. This process was repeated for five cycles and the excellent yield of product (4a) was obtained in each cycle as mentioned in Scheme 5. Following the recyclability experiment, we have characterised our catalyst by infrared spectroscopy as it is typical characterisation technique in case of polyoxometalate compound. The IR spectra of polyoxometalates exhibit characteristic absorption bands corresponding to the vibrations of metal–oxygen bonds, such as M=O terminal bonds, M–O–M corner-sharing bonds, and M–O–M edge-sharing bonds. We have found there is almost no change in the spectra with respect to the previous one even after long term catalysis. These results imply that the catalyst's activity is only slightly altered and mostly remained as same as in the run.

To showcase the practical synthetic utility of SMA, a gram-scale experiment was carried out for the synthesis of 4a (Table 11, entry 1), where benzaldehyde (0.408 mL, 4 mmol), dimedone (1.12 g, 8 mmol), and SMA (1 mol%) were treated in IPA (8 mL) following the same optimized protocol, yielding 1.08 g of product 4a with a good isolated yield of 77%.

A comparative assessment of the catalytic performance of the proposed SMA against several recently reported catalysts is provided in Table 12. While many of these methods afford good to excellent yields under optimized conditions, they often suffer from notable limitations, including the need for high catalyst loading, prolonged reaction times, elevated reaction temperatures, and the use of toxic or hazardous solvents. These factors collectively hinder the practicality and sustainability of such methods for large-scale or routine applications. In contrast, the catalytic use of SMA presents significant advantages. As depicted in Table 12, SMA facilitates the synthesis of compound 4a under considerably milder conditions namely, at low temperature, with reduced reaction time and minimal catalyst loading. Moreover, the reaction is carried out in an environmentally friendly solvent system, specifically isopropyl alcohol (IPA), further enhancing the green credentials of the process. In addition to its excellent catalytic activity, SMA is a stable, reusable, and commercially accessible solid acid catalyst that can be easily handled and recovered. These combined features underscore its potential as a highly efficient, practical, and sustainable catalyst for the synthesis of 4a.

**Table 12:** Comparison of SMA's catalytic efficiency for the synthesis of 4a with that of other catalytic systems

Catalyst	Amount of catalyst	Yield (%)	Solvent	Time (min)	Temperature (°C)
SmCl_3_ (ref. 57)	20 mol%	98	—	540	120
CAN^59^	5 mol%	96	PEG 400	240	50
SbCl_3_/SiO_2_ (ref. 60)	10 mol%	93	—	50	120
Sr(OTf)_2_ (ref. 61)	10 mol%	85	DCM	300	80
H_14_[NaP_5_W_30_O_110_]^62^	0.4 mol%	88	—	120	120
**SMA (this work)**	**1 mol%**	**98**	**IPA**	**80**	**70**

## 4. Conclusion

In summary, we have successfully explored SMA as a Lewis acid catalyst for the preparation of various biologically significant O/N-fused heterocyclic organic molecules. Finally, the synthesis of a benzylidene derivative under benign conditions has been fruitfully accomplished using a novel, reusable, and efficient green catalyst called SMA. Similarly, our technique uses SMA, a readily available catalyst, for the synthesis of 2-amino-5-oxo-5,6,7,8-tetrahydro-4*H*-chromenes derivatives in a one-pot, three-component process at room temperature with an excellent yield. Furthermore, We have provided a straightforward, practical, and effective technique for the synthesis of many 2-amino-4-substituted-1,4-dihydrobenzo[4,5]imidazolo[1,2-*a*]pyrimidine-3-carbonitriles derivatives in water at room temperature within a short span of time with an excellent yield. In fact, it may be the preferred approach for obtaining different functionalized imidazopyrimidine derivatives owing to its affordability, efficacy, gentle reaction conditions, excellent product yields, simple work-up approach, and use of a very minimal quantity of SMA. Eventually, the procedure reported here for the synthesis of 1,8-dioxooctahydroxanthene derivatives has offered a more realistic improvement compared to conventional methods. The key advantage of using SMA in this reaction is that it eliminates the need for column chromatography for product purification, as commonly reported in the literature, thereby significantly reducing overall production costs. Moreover, the reaction conditions are remarkably simple most reactions proceed at room temperature in a greener solvent. Additionally, the catalyst can be easily separated, the product is obtained in a pure form, and the catalyst remains recyclable, enhancing the sustainability of the process. All the reaction is scalable for gram scale.

## Author contributions

Neeraj K. Sah: writing – original draft, investigation, formal analysis, data curation. Krishna Kumar: methodology, investigation, data curation. Subrato Bhattacharya: resources, methodology, investigation, formal analysis, data curation. Tanay Pramanik: writing – review & editing, supervision, methodology. Tanmoy Roy: writing – review & editing, software, resources, computational investigation, supervision. Somenath Garai: writing – review & editing, writing – original draft, supervision, methodology, resources, funding acquisition, conceptualization.

## Conflicts of interest

The authors declare that they have no known competing financial interests or personal relationships that could have appeared to influence the work reported in this paper.

## Supplementary Material

RA-015-D5RA03549J-s001

RA-015-D5RA03549J-s002

## Data Availability

Crystallographic data for compound 2d and 4j has been deposited at the CCDC under with CCDC number 2386630 and 2386631 respectively. CCDC 2386630 and 2386631 contain the supplementary crystallographic data for this paper.^63,64^ The data supporting this article have been included as part of the SI. See DOI: https://doi.org/10.1039/d5ra03549j.
