# Supplementary material for: Unveiling the catalytic potential of silicomolybdic acid in crafting diverse biologically relevant organic compounds
Source: RSC Adv. 2025 Aug 13;15(35):28783–804. doi: 10.1039/d5ra03549j (PMC12377150; doi:10.1039/d5ra03549j)
Supplement: RA-015-D5RA03549J-s001 [file RA-015-D5RA03549J-s001.pdf]

Supplementary Information (SI) for RSC Advances.
This journal is © The Royal Society of Chemistry 2025

Supporting information

Unveiling the Catalytic Potential of Silicomolybdic Acid in Crafting Diverse Biologically Relevant Organic Compounds

Neeraj K. Sah,[a] Krishna Kumar,[a] Subrato Bhattacharya,[a] Tanay Pramanik,[b]* Tanmoy Roy,[c]* Somenath Garai[a]*

[a] Department of Chemistry, Institute of Science, Banaras Hindu University, Varanasi 221 005, Uttar Pradesh, India;

[b] Department of Chemistry, Institute of Engineering and Management, University of Engineering and Management Kolkata, University Area, Action area 3, Newtown, Kolkata 700160;

[c] Department of Chemistry, Lovely Professional University, Jalandhar-Delhi, G.T. Road, Phagwara, Punjab (INDIA) -144411;

Table of Contents

Contents	Page
Structure of Compounds **1a-m, 2a-j, 3a-j, 4a-j**.	S2-S3
^{1}H-NMR and ^{13}C {^{1}H} NMR spectra of Compounds **1a-m.**	S4-S16
^{1}H-NMR and ^{13}C {^{1}H} NMR spectra of Compounds **2a-j.**	S17-S26
^{1}H-NMR and ^{13}C {^{1}H} NMR spectra of Compounds **3a-j.**	S27-S36
^{1}H-NMR and ^{13}C {^{1}H} NMR spectra of Compounds **4a-j**.	S37-S46
HRMS spectra of Compounds **2d, 2e, 2g, 3b-3h**	S47-S51
HRMS spectra of Compounds **4a-j**	S51-S55
Single Crystal for compound **2d** and **4j**	S56-S58
Cartesian coordinates for the optimized structures shown in **Fig. 5**	S59-S69

Structure of Compounds **1a-m**.

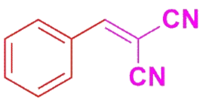

1a

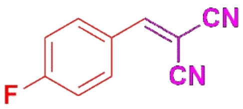

1b

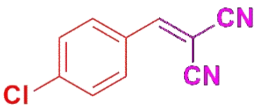

1c

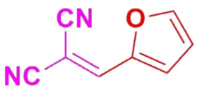

1d

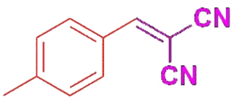

1e

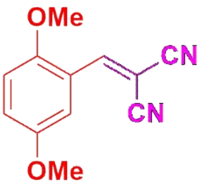

1f

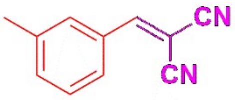

1g

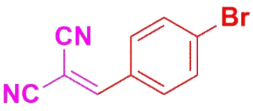

1h

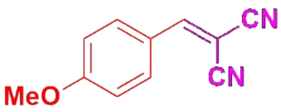

1i

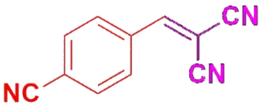

1j

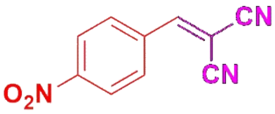

1k

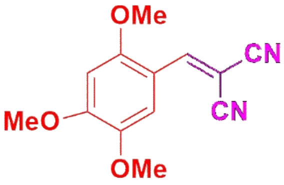

1l

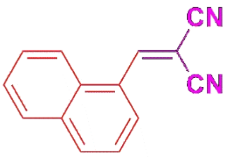

1m

Structure of Compounds **2a-j**.

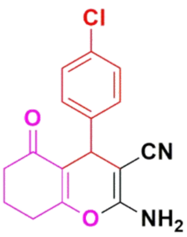

2a

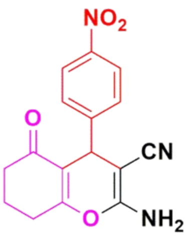

2b

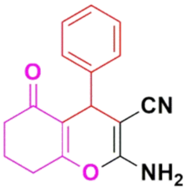

2c

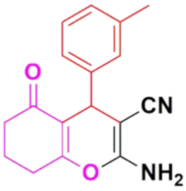

2d

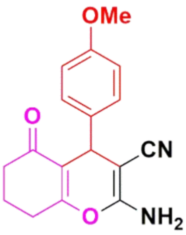

2e

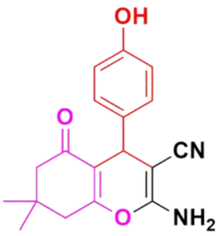

2f

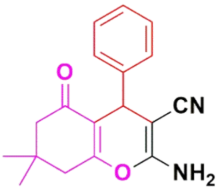

2g

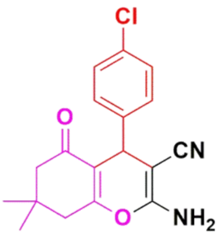

2h

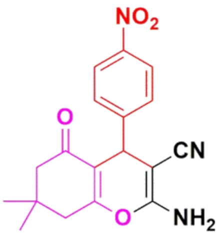

2i

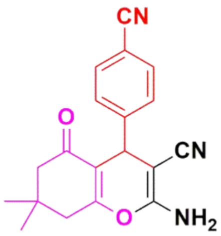

2j

Structure of Compounds **3a-j**.

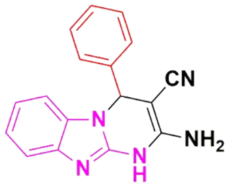

3a

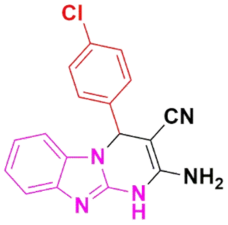

3b

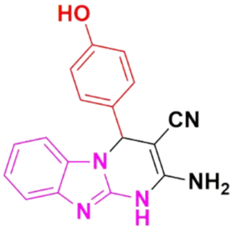

3c

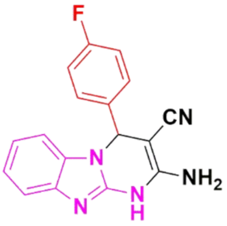

3d

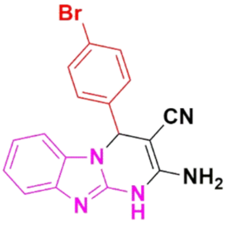

3e

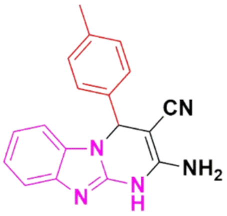

3f

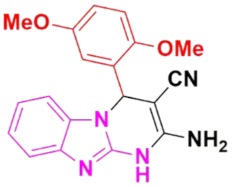

3g

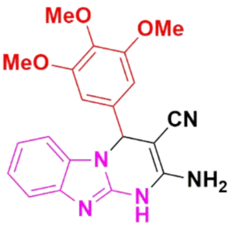

3h

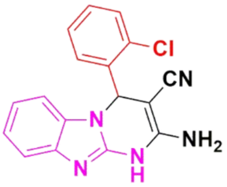

3i

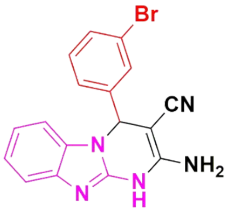

3j

Structure of Compounds **4a-j**.

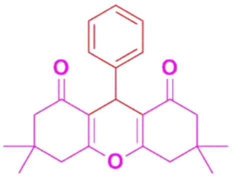

4a

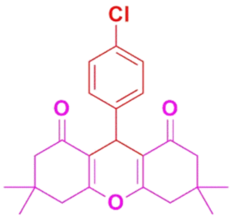

4b

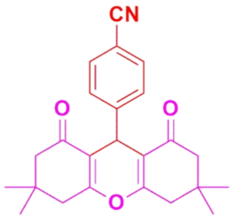

4c

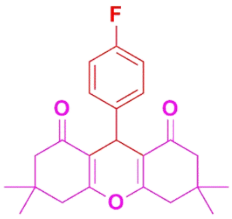

4d

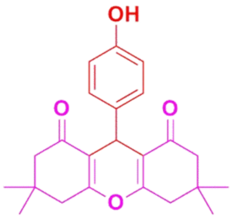

4e

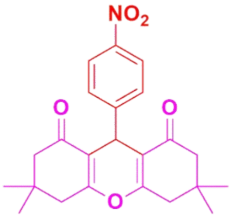

4f

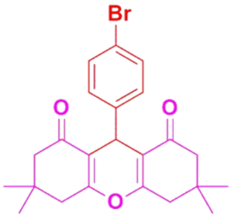

4g

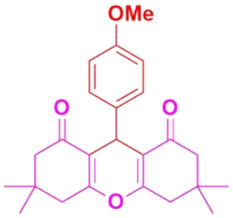

4h

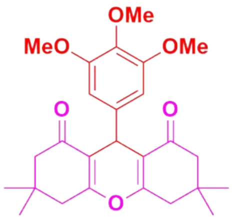

4i

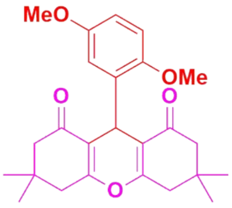

4j

^{1}H-NMR of 1a in $CDCl_3$

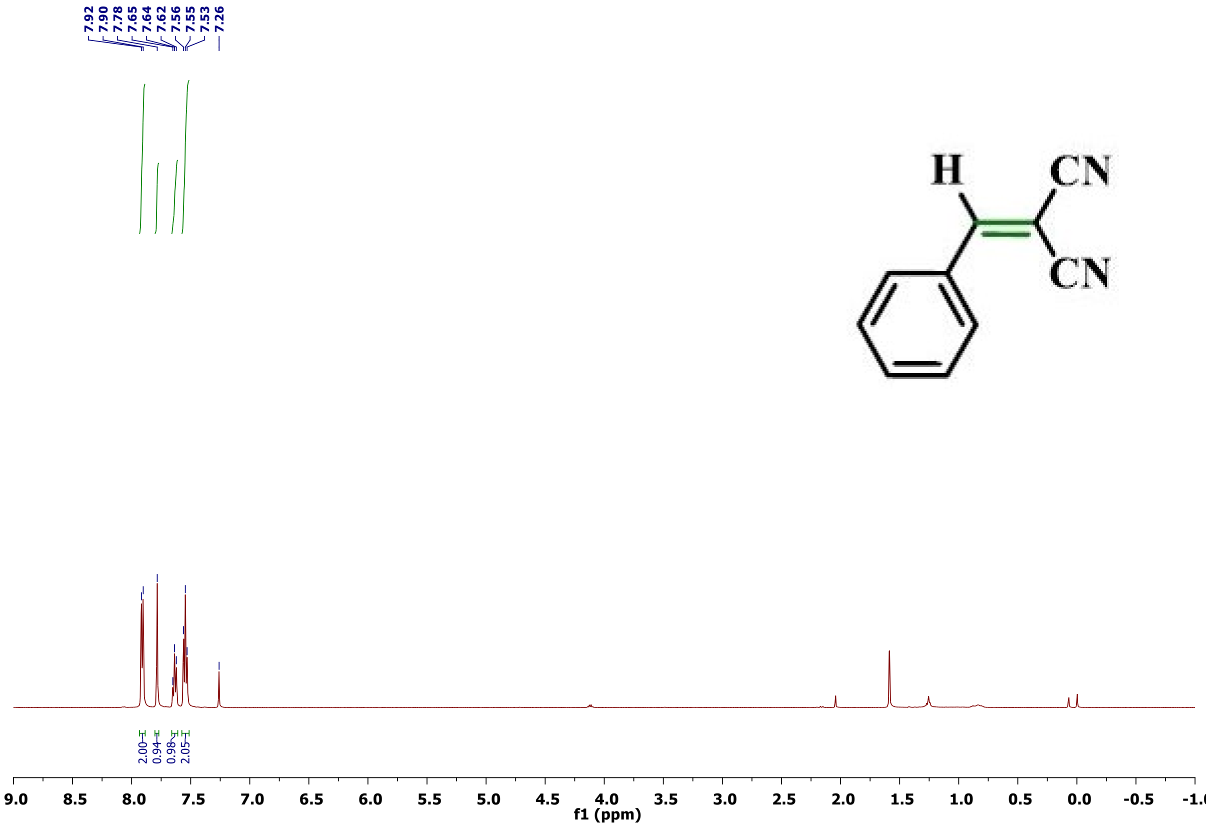

^{13}C {^{1}H} NMR of 1a in $CDCl_3$

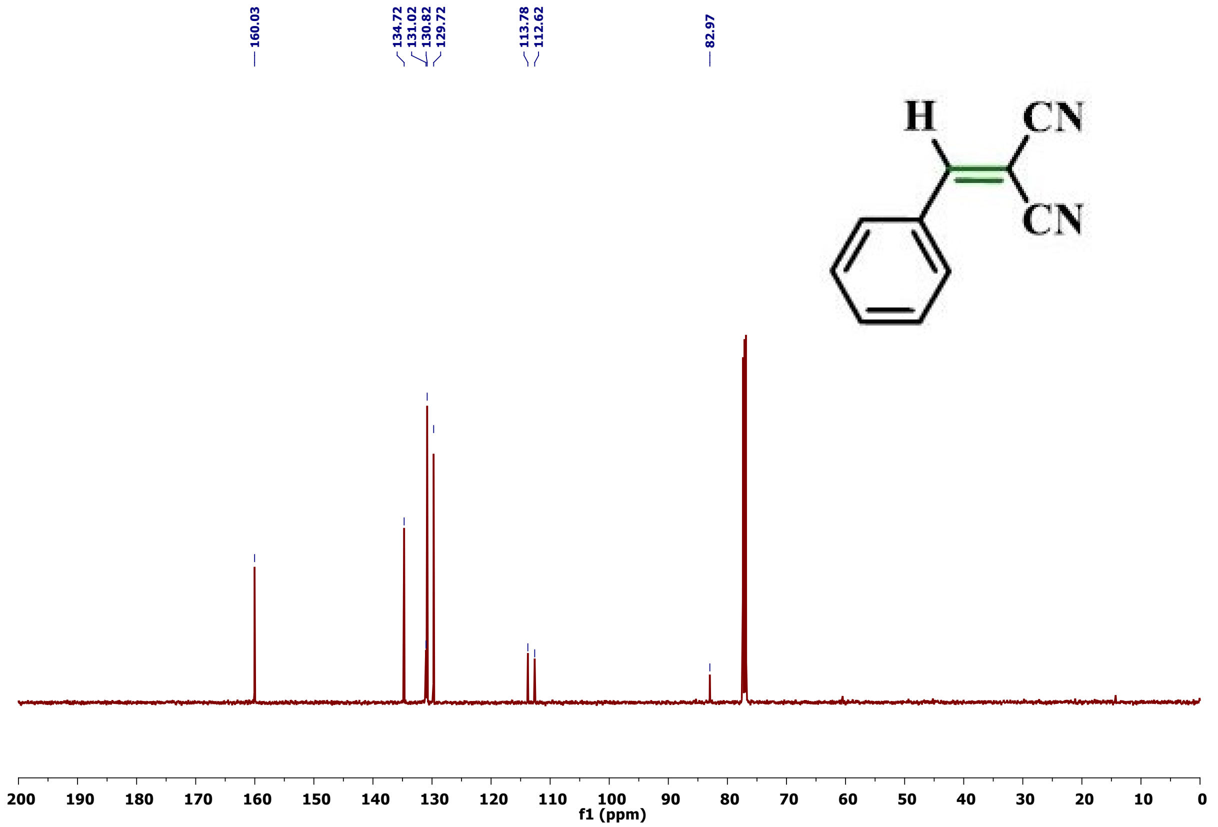

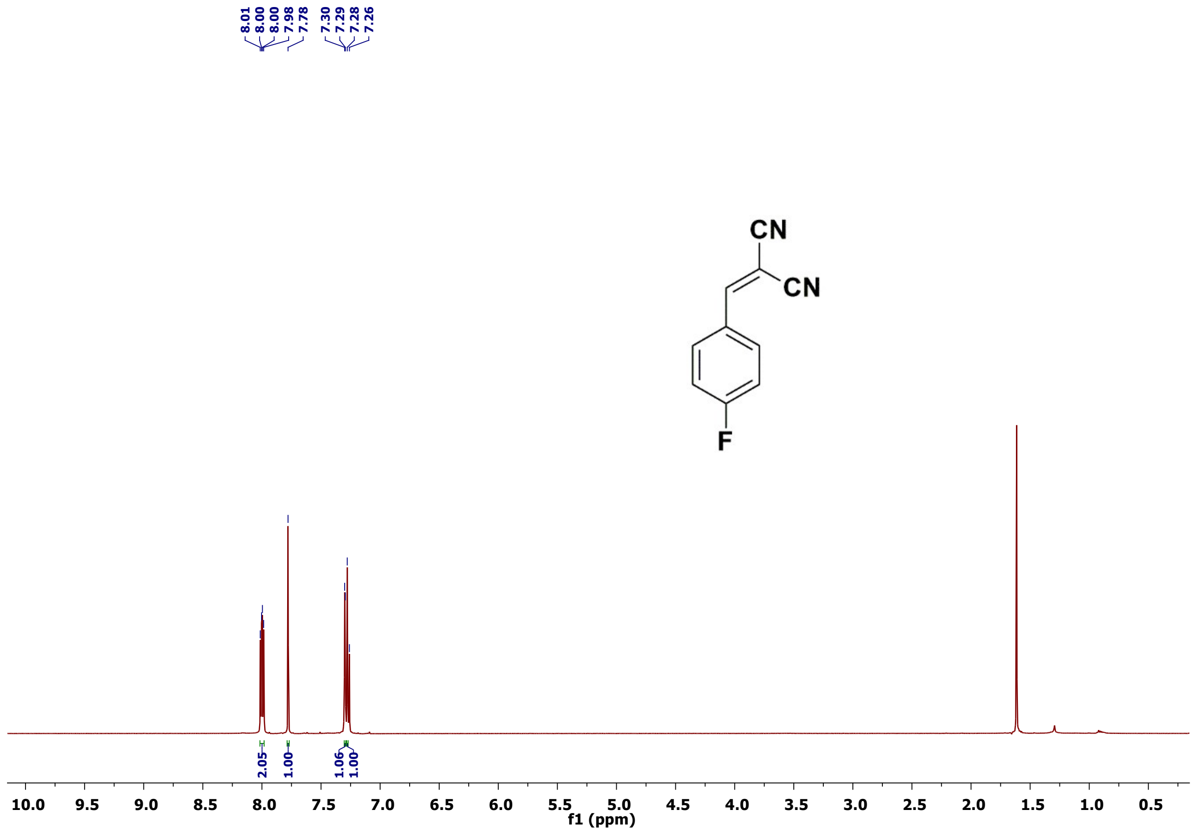

^{1}H-NMR of 1b in $CDCl_3$

^{13}C {^{1}H} NMR of 1b in $CDCl_3$

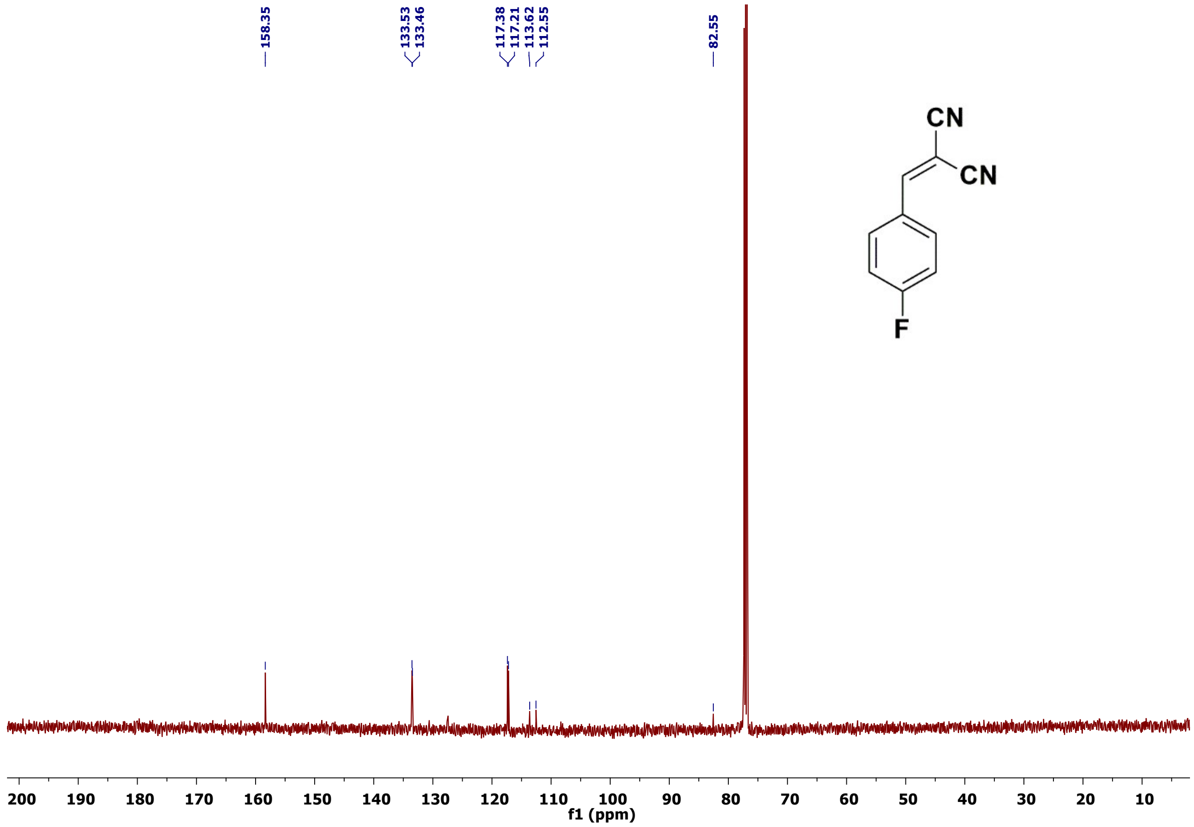

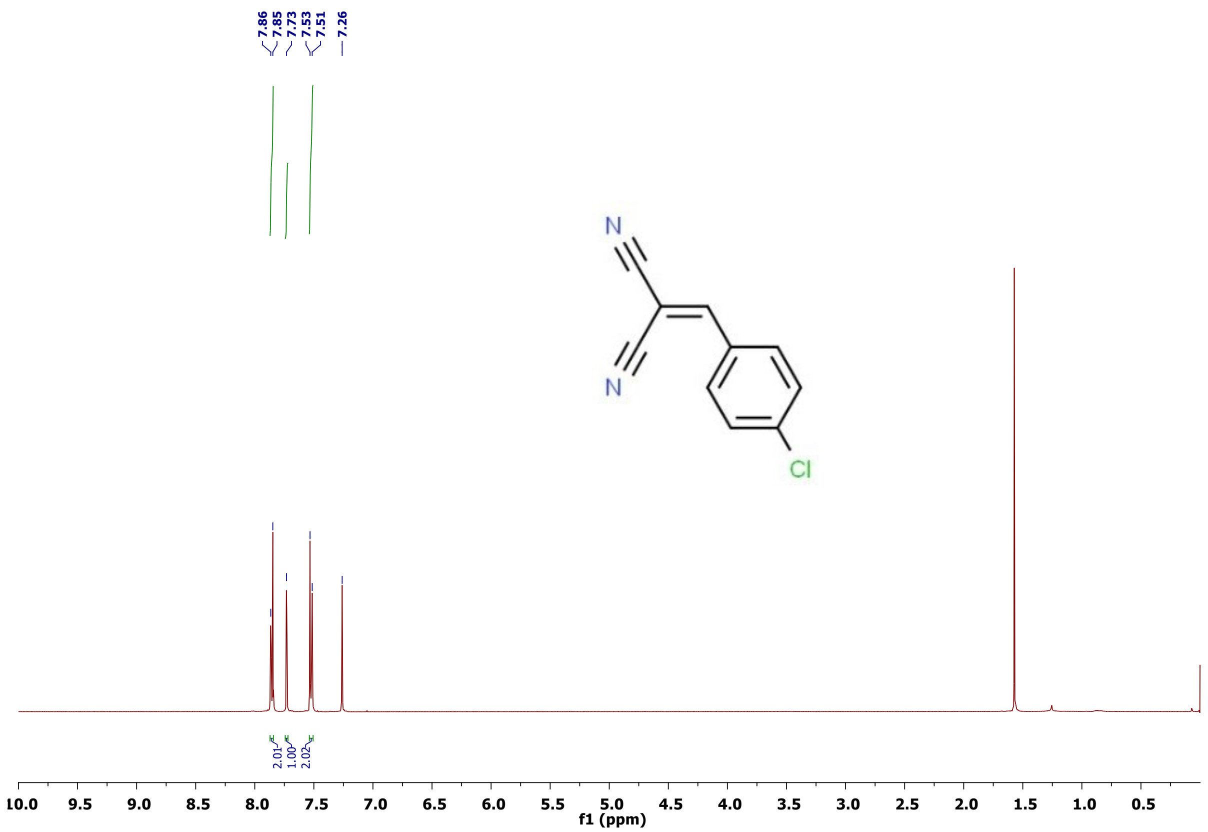

^{1}H-NMR of 1c in $CDCl_3$

^{13}C {^{1}H} NMR of 1c in $CDCl_3$

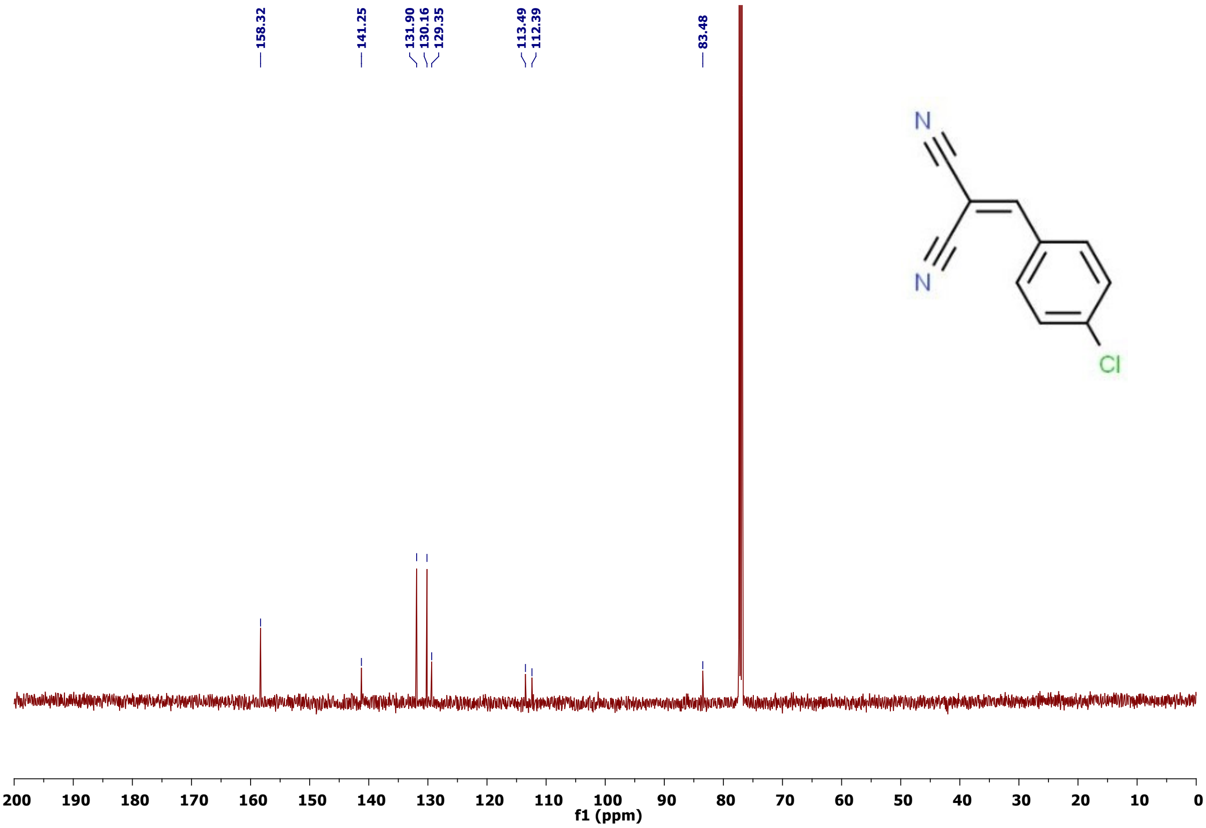

^{1}H-NMR of 1d in $CDCl_3$

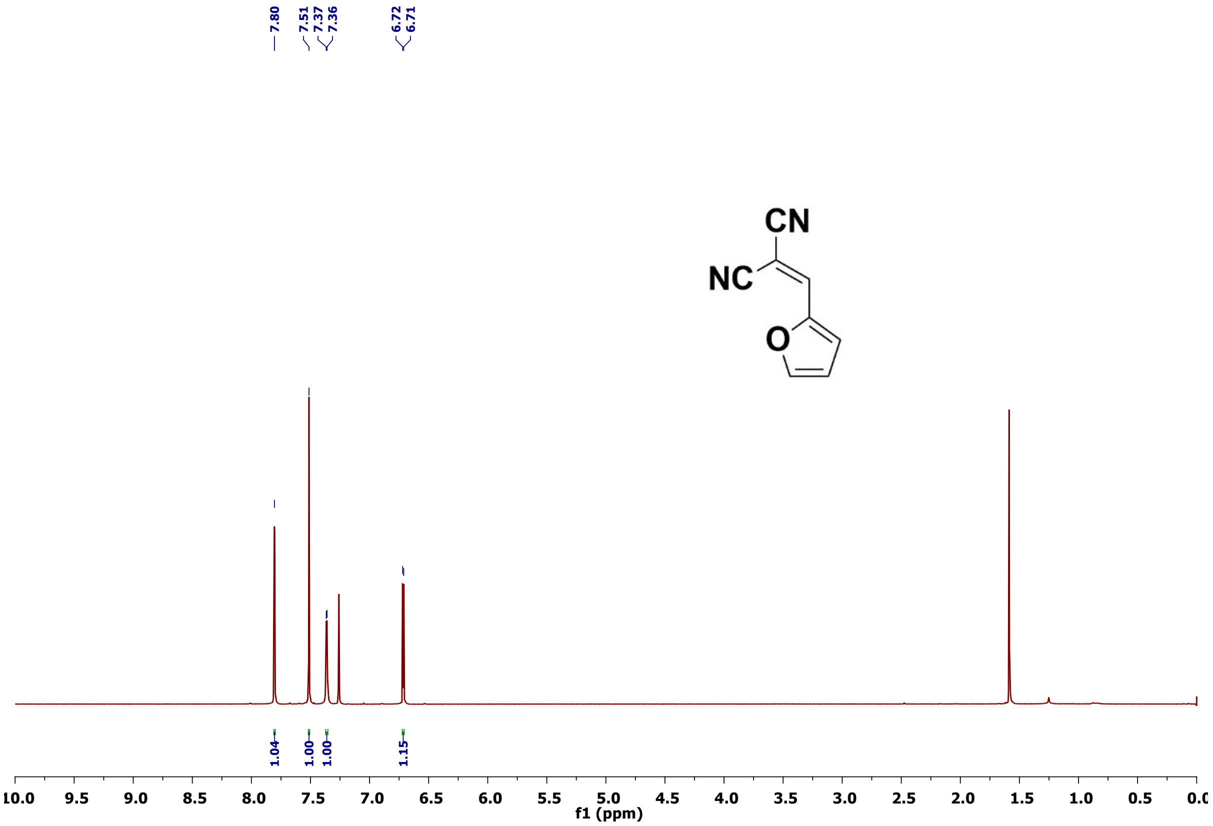

^{13}C {^{1}H} NMR of 1d in $CDCl_3$

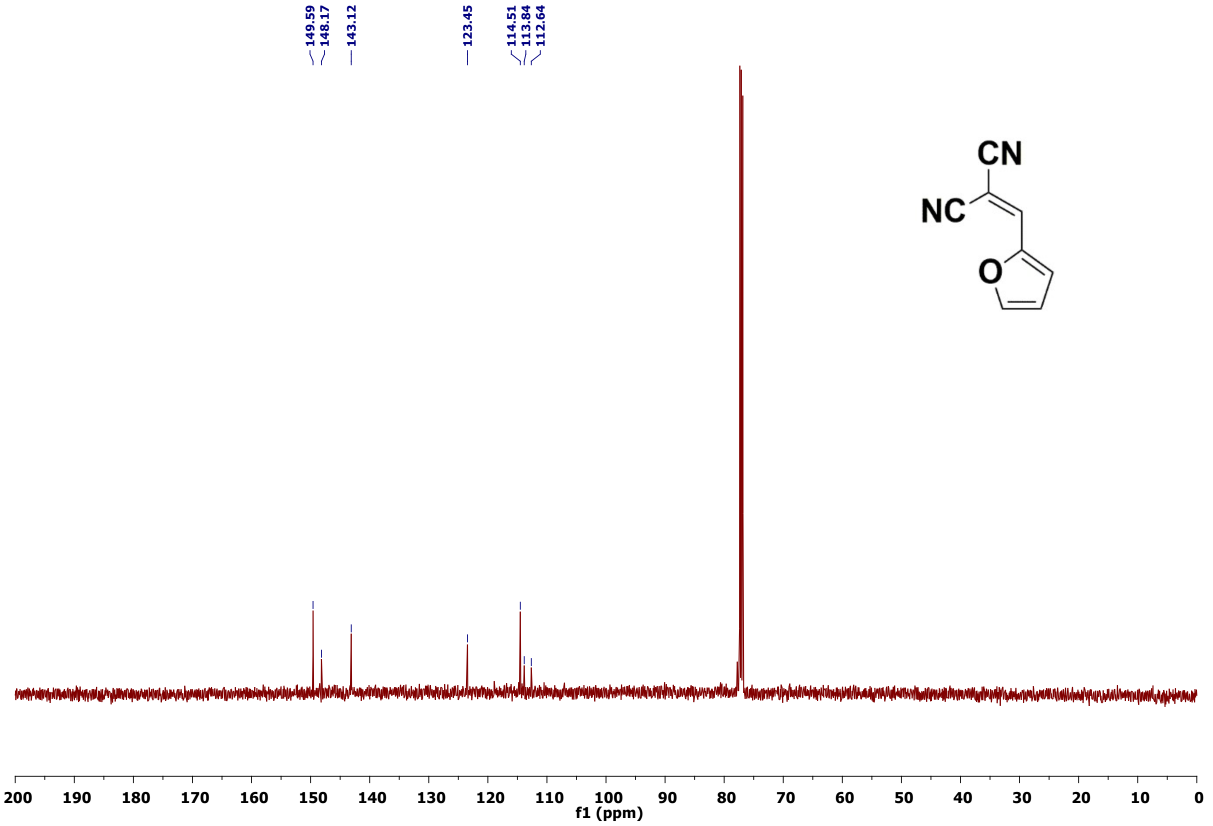

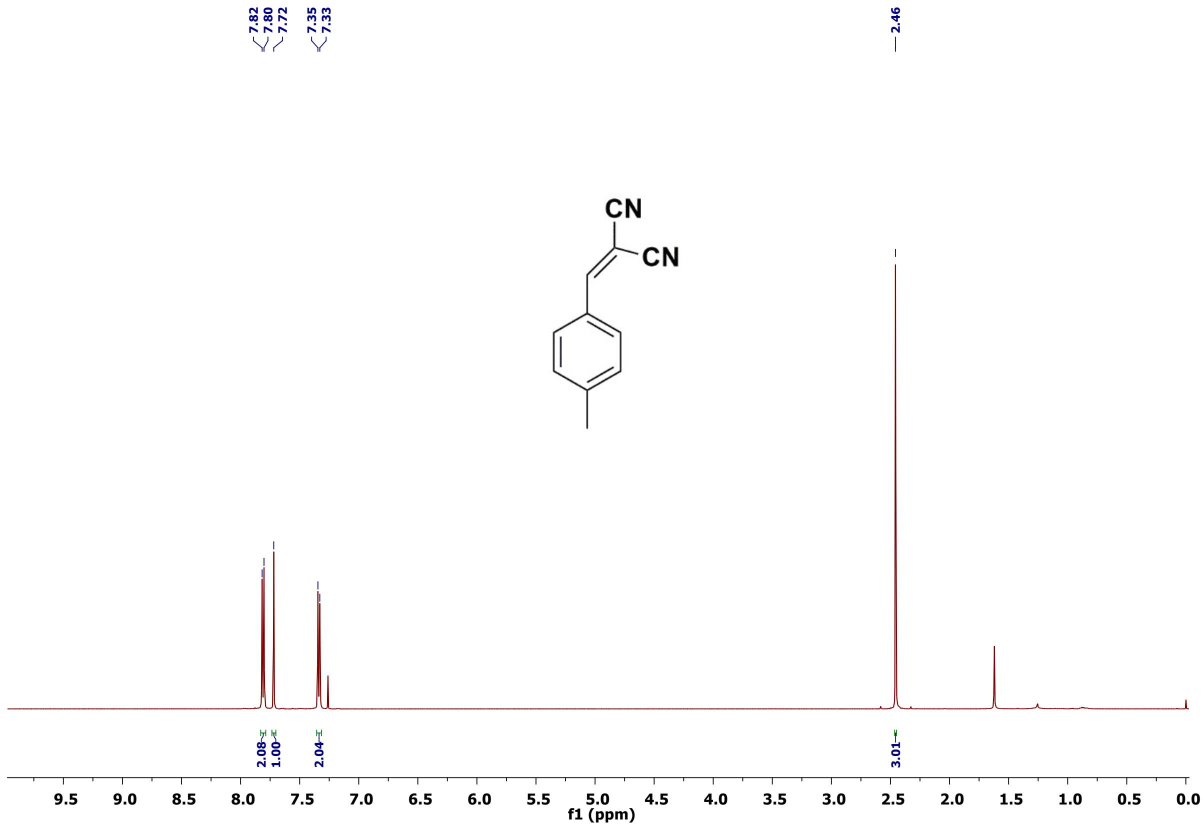

^{1}H-NMR of 1e in $CDCl_3$

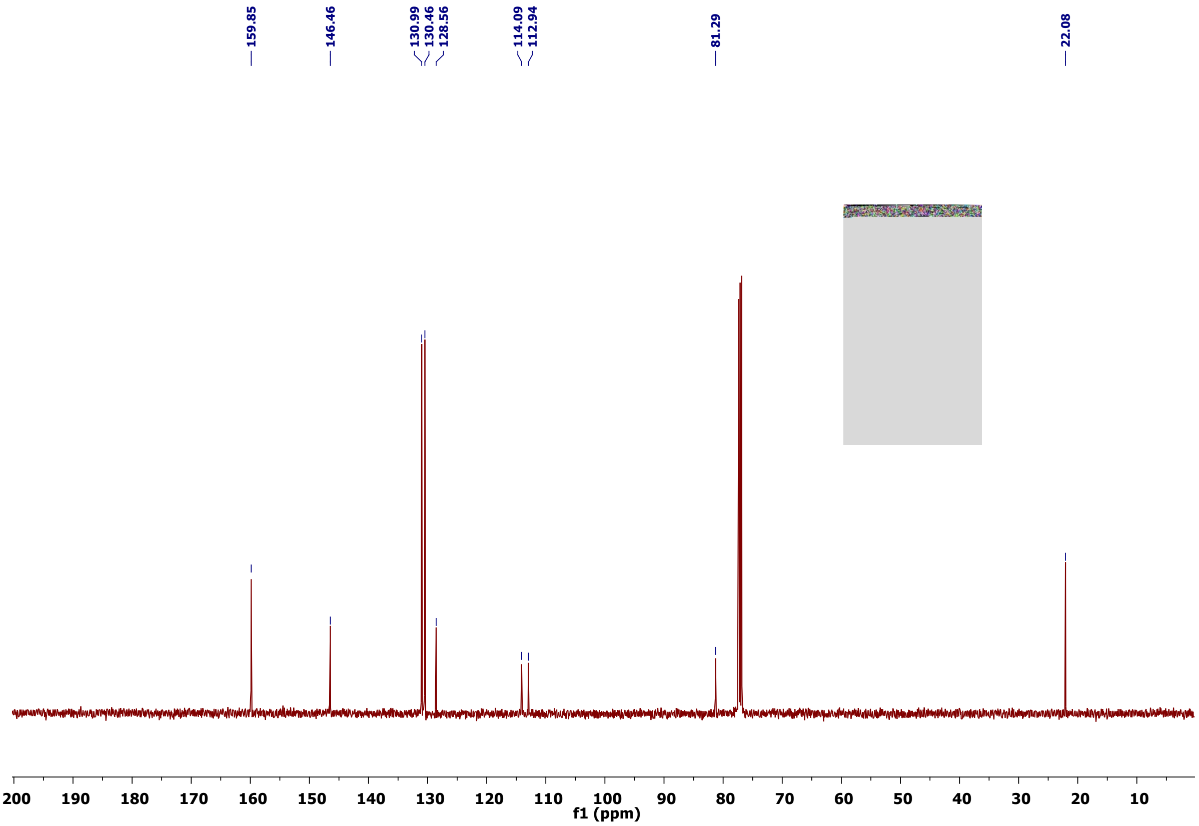

^{13}C {^{1}H} NMR of 1e in $CDCl_3$

^{1}H-NMR of 1f in $CDCl_3$

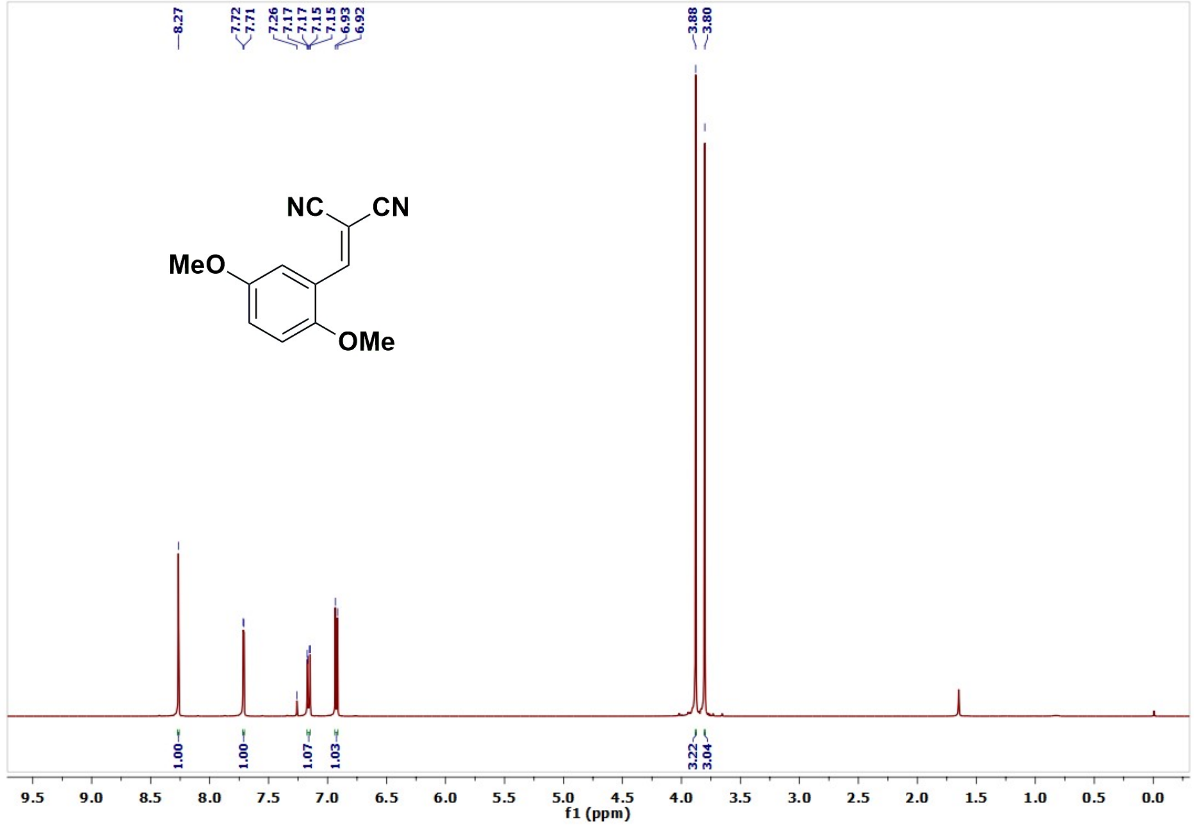

$^{13}C\ \{^1H\}$ NMR of 1f in $CDCl_3$

^1H-NMR of 1g in $CDCl_3$

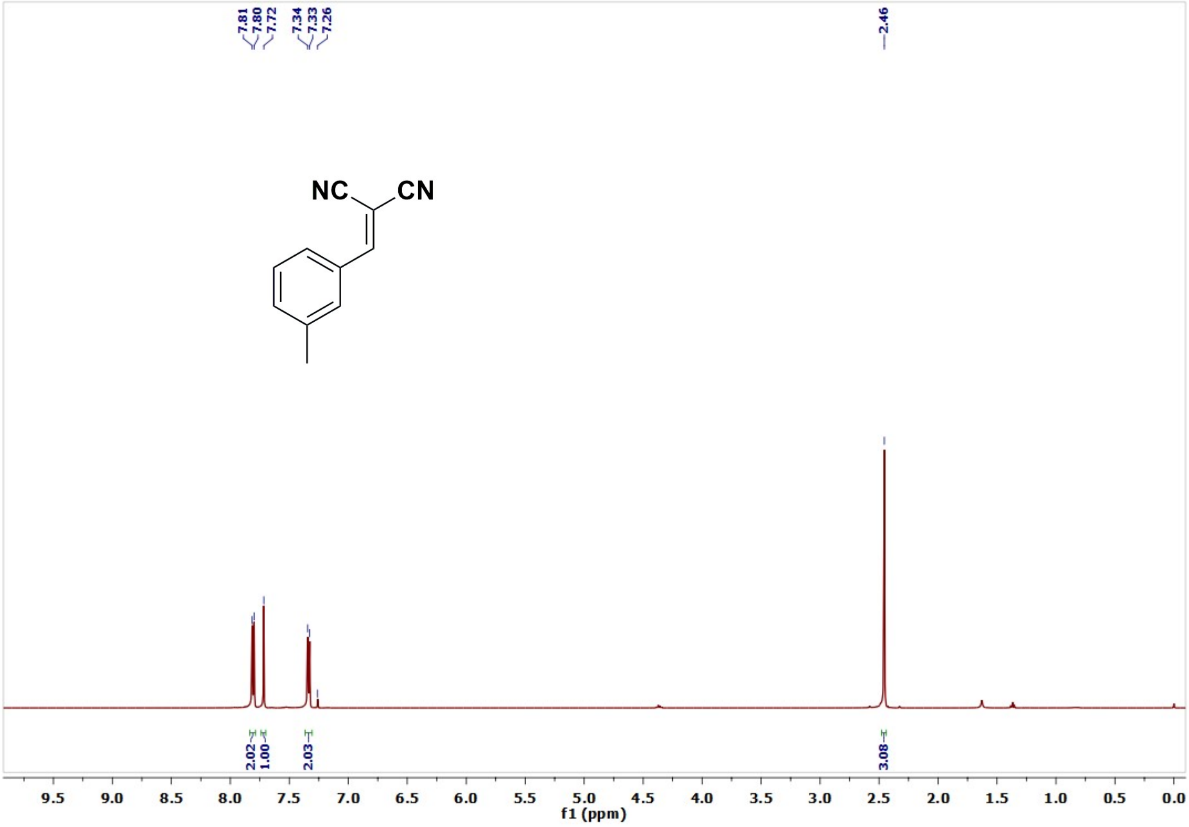

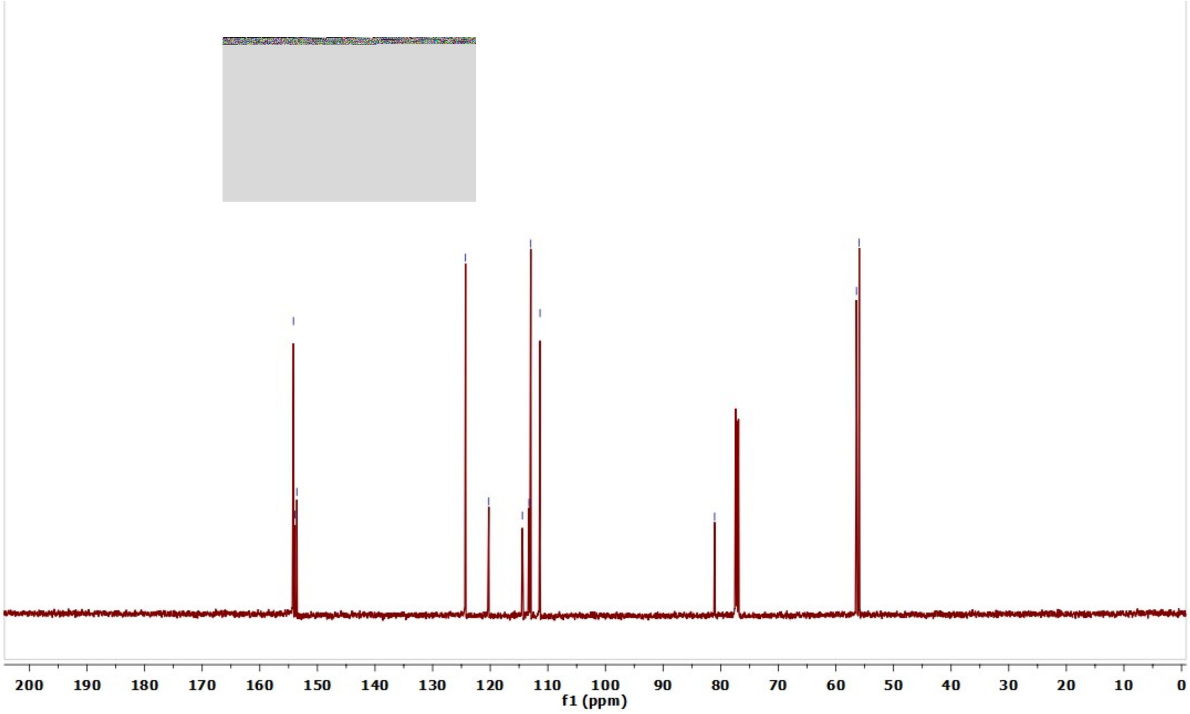

^{13}C {^{1}H} NMR of 1g in $CDCl_3$

^{1}H-NMR of 1h in $CDCl_3$

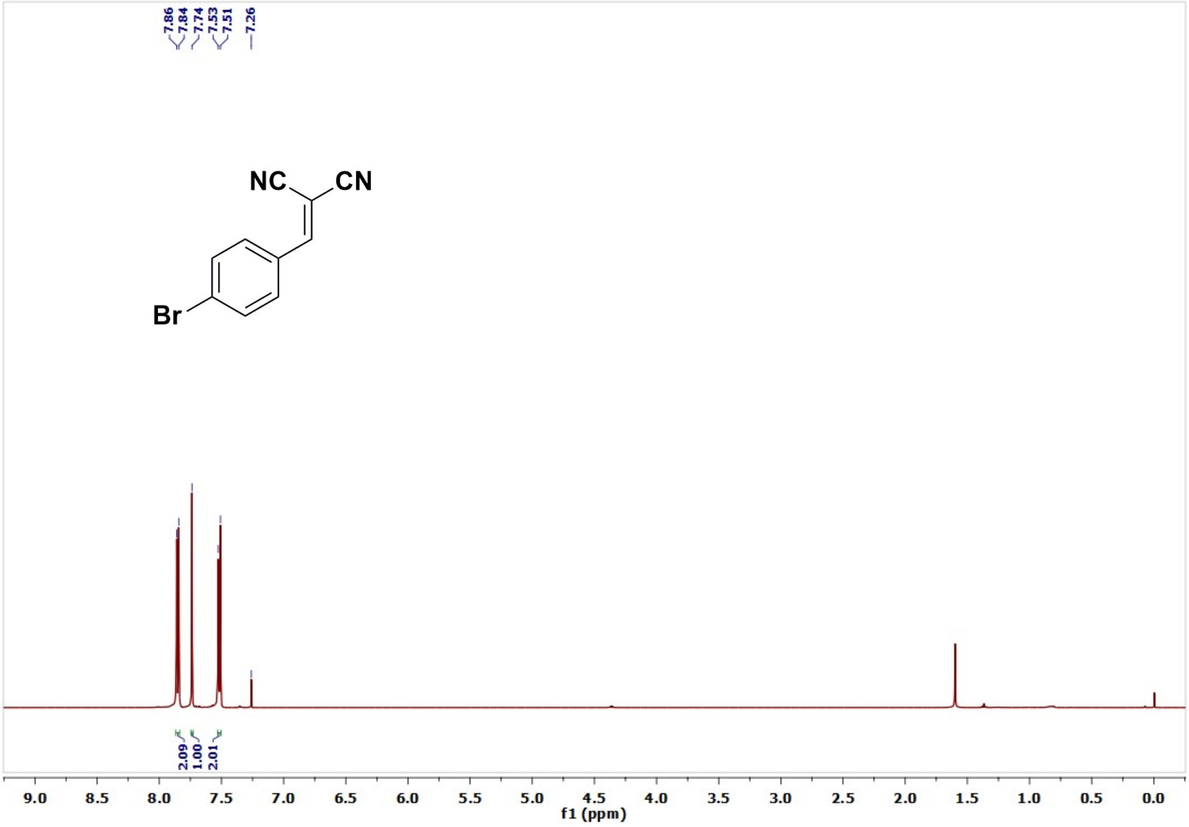

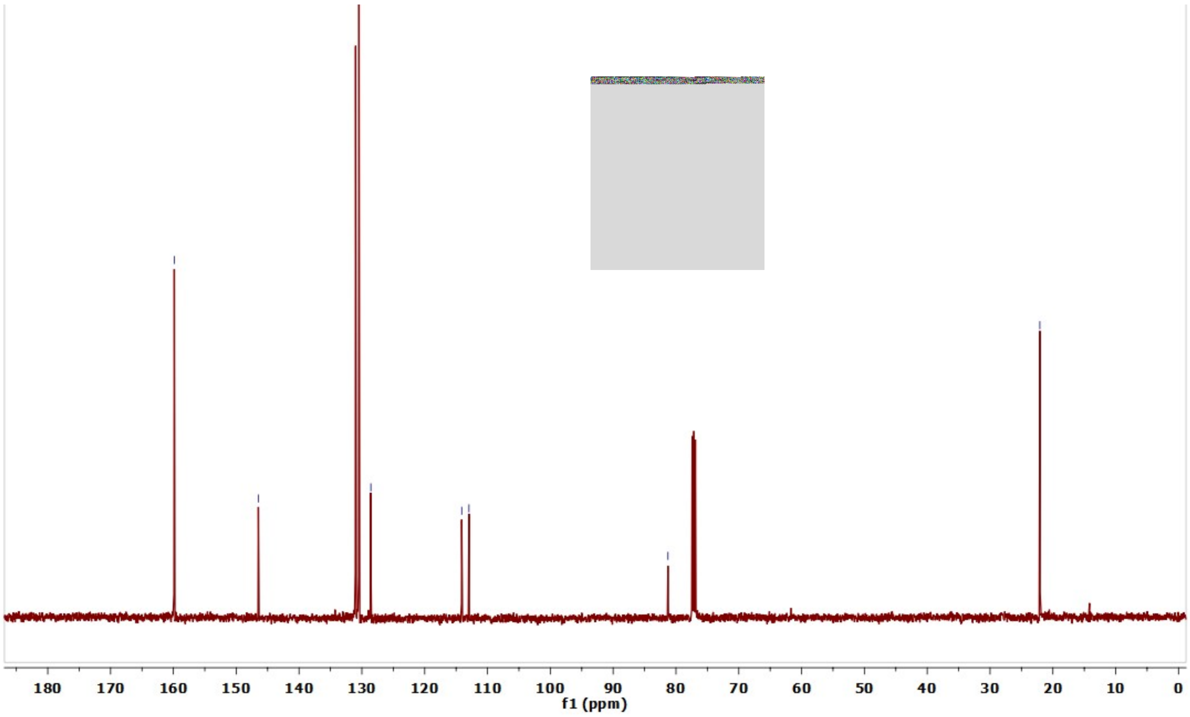

$^{13}C\ \{^{1}H\}$ NMR of 1h in $CDCl_3$

^{1}H-NMR of 1i in $CDCl_3$

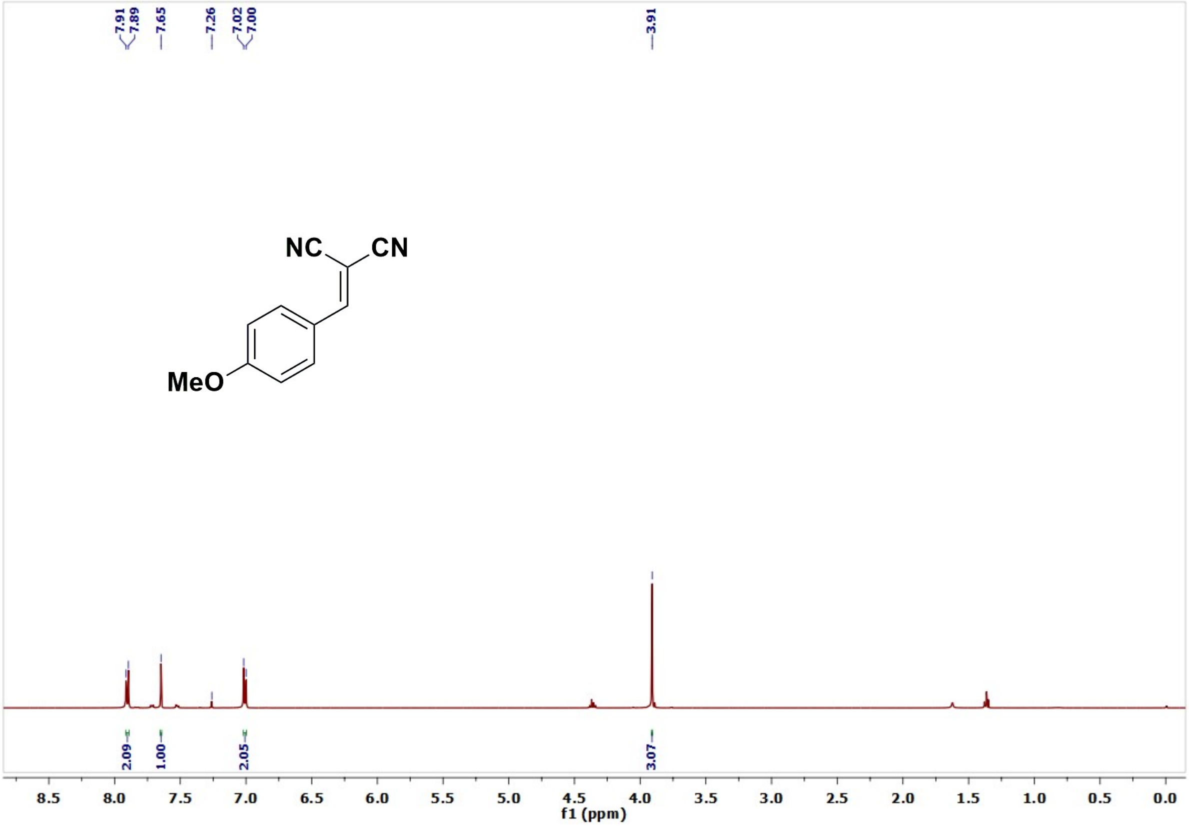

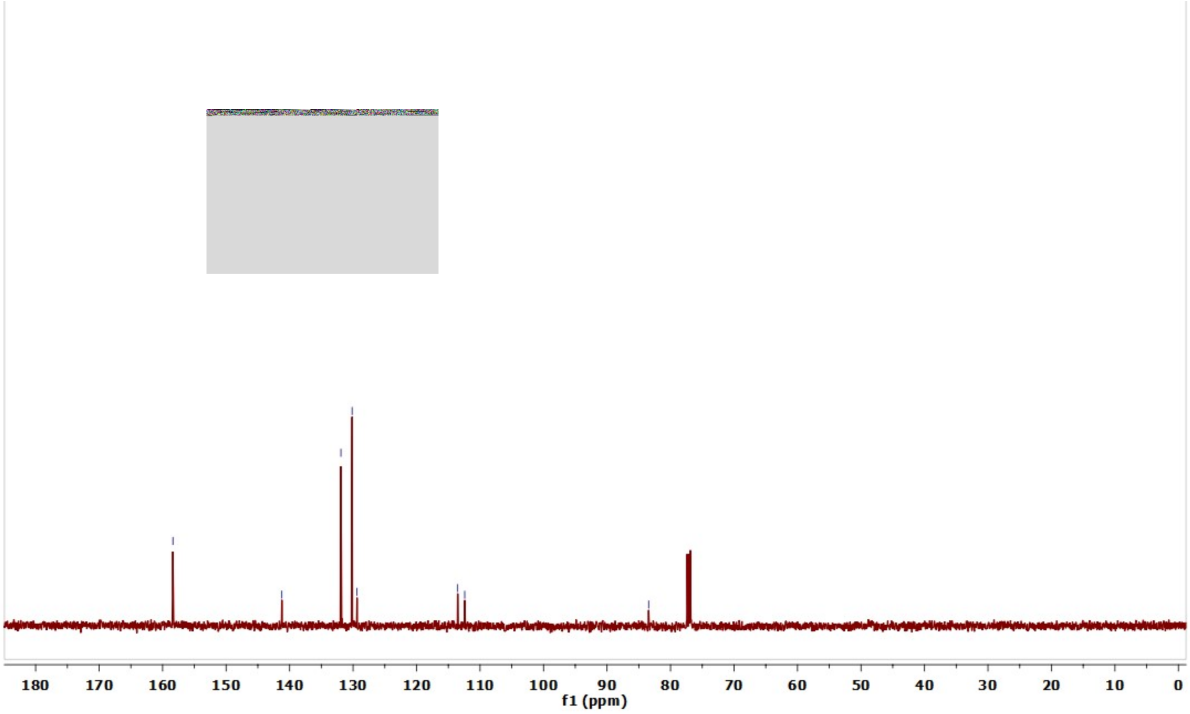

$^{13}C\{^{1}H\}$ NMR of 1i in $CDCl_3$

^{1}H-NMR of 1j in $CDCl_3$

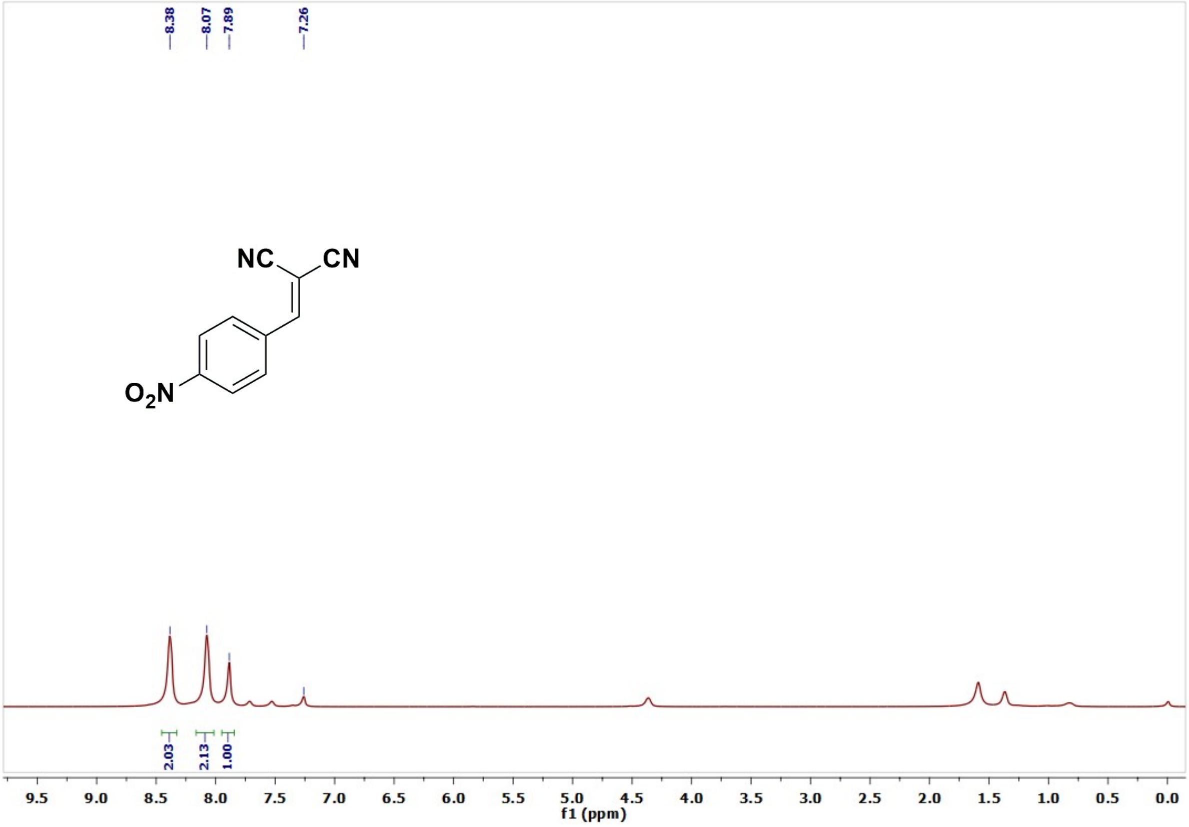

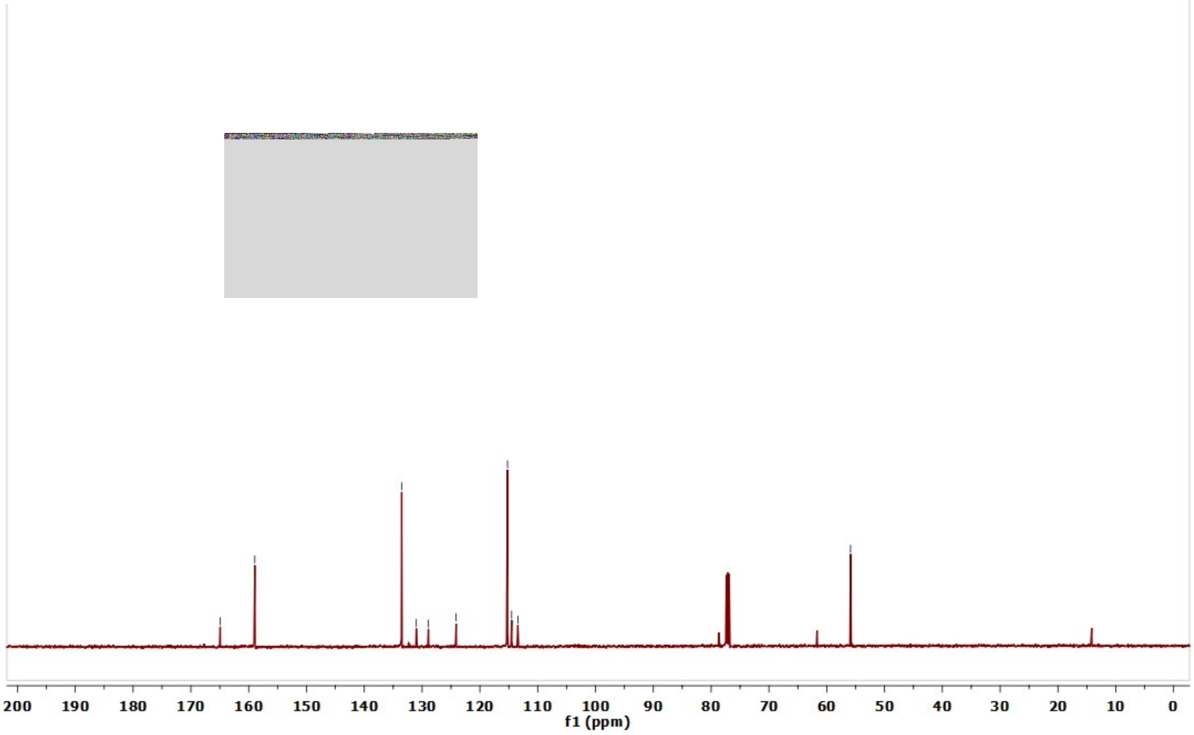

^{13}C {^{1}H} NMR of 1j in $CDCl_3$

^{1}H-NMR of 1k in $CDCl_3$

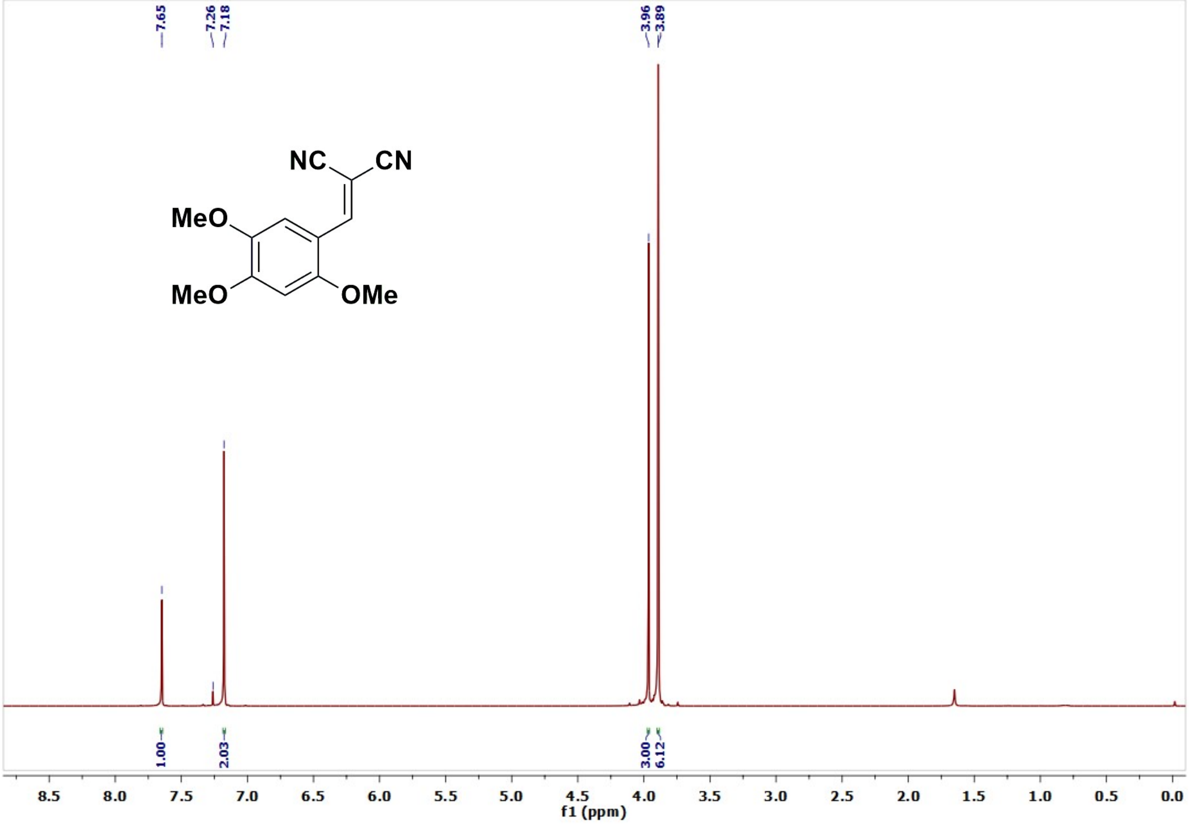

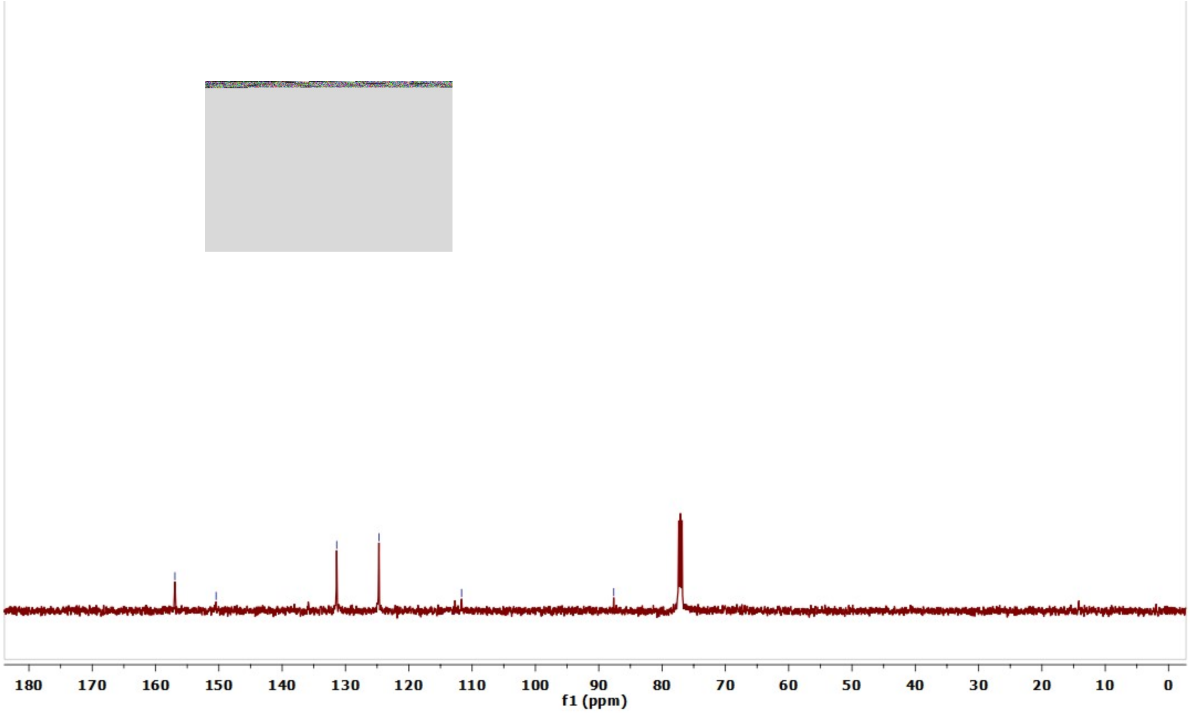

^{13}C {^{1}H} NMR of 1k in $CDCl_3$

^{1}H-NMR of 1l in $CDCl_3$

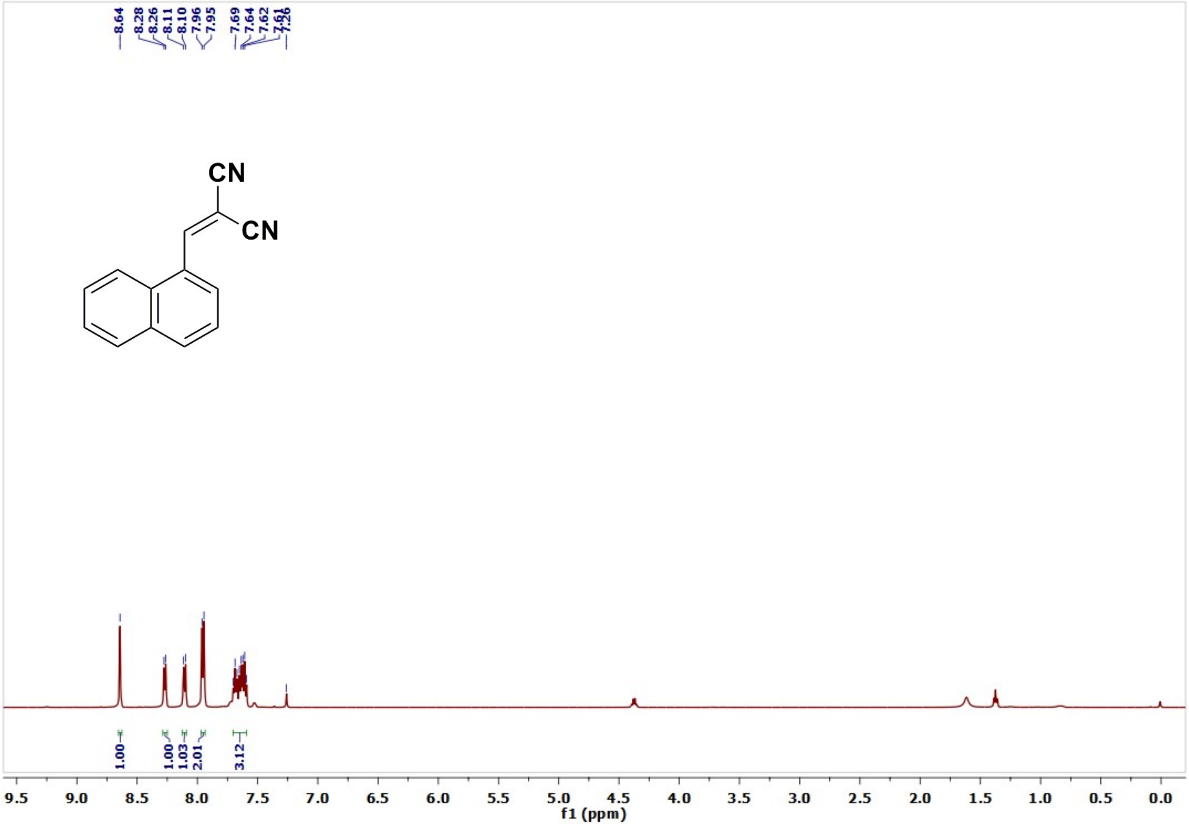

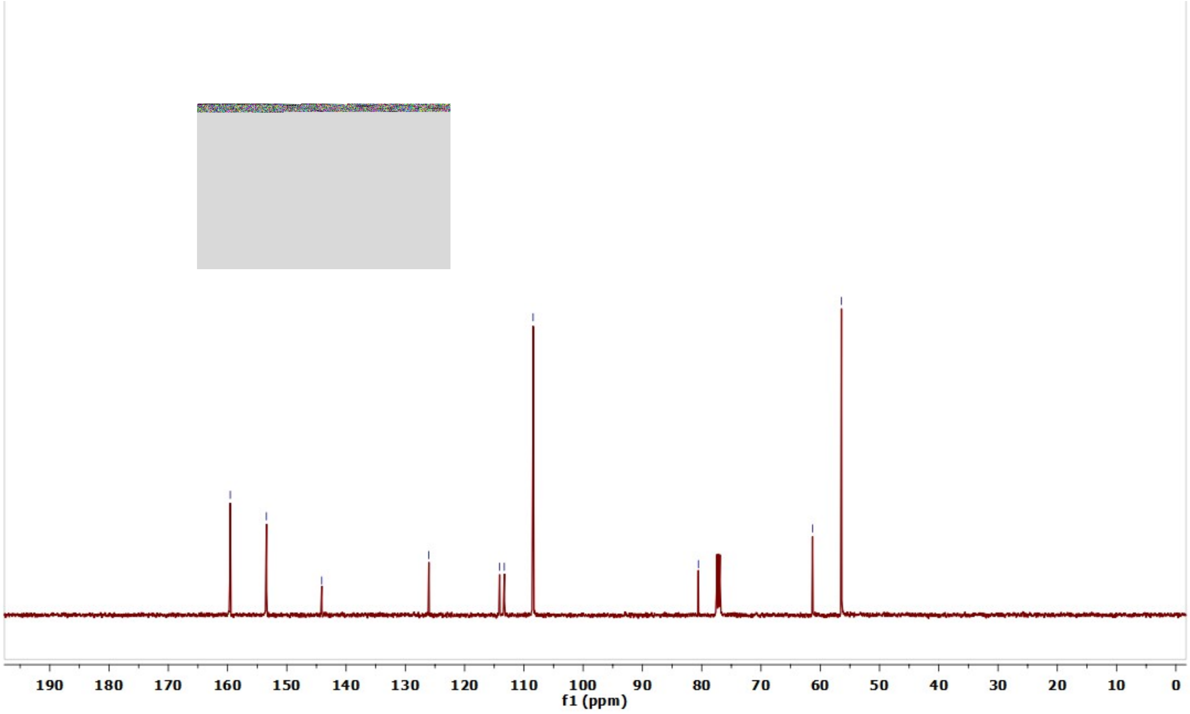

^{13}C {^{1}H} NMR of 1l in $CDCl_3$

^{1}H-NMR of 1m in $CDCl_3$

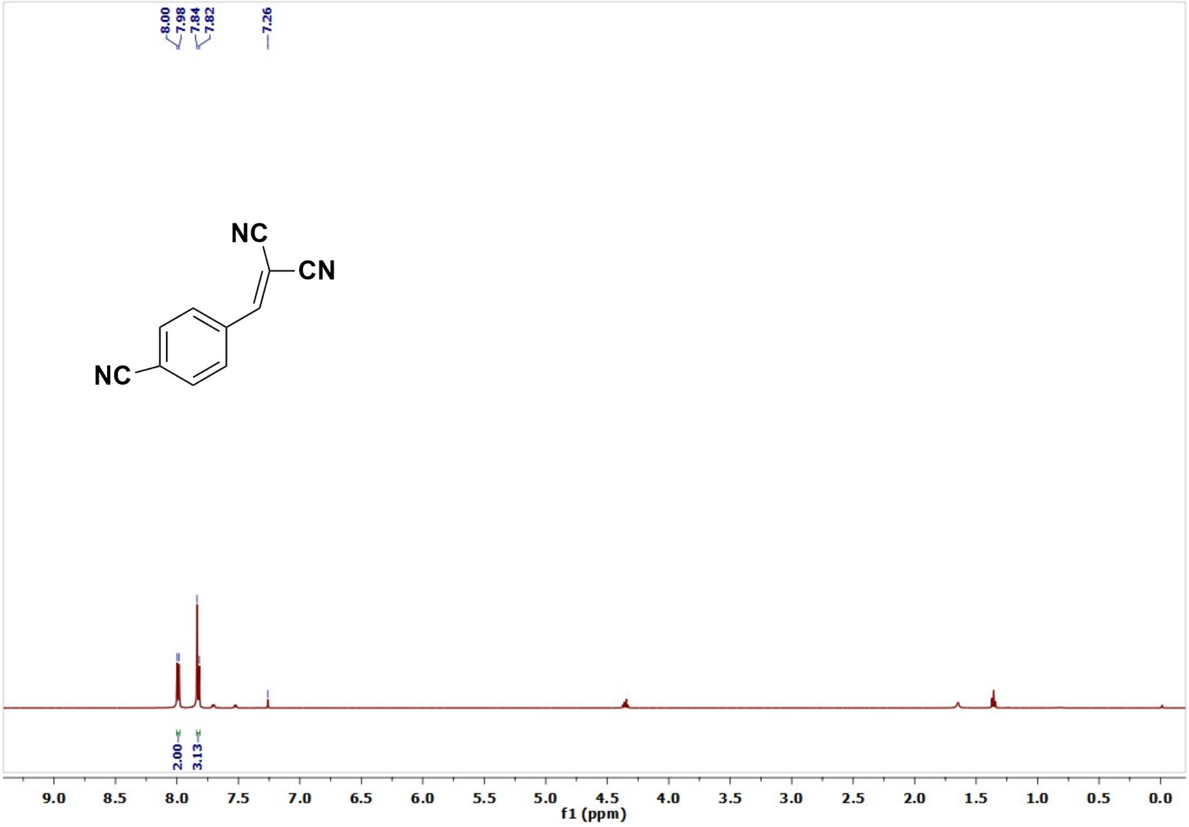

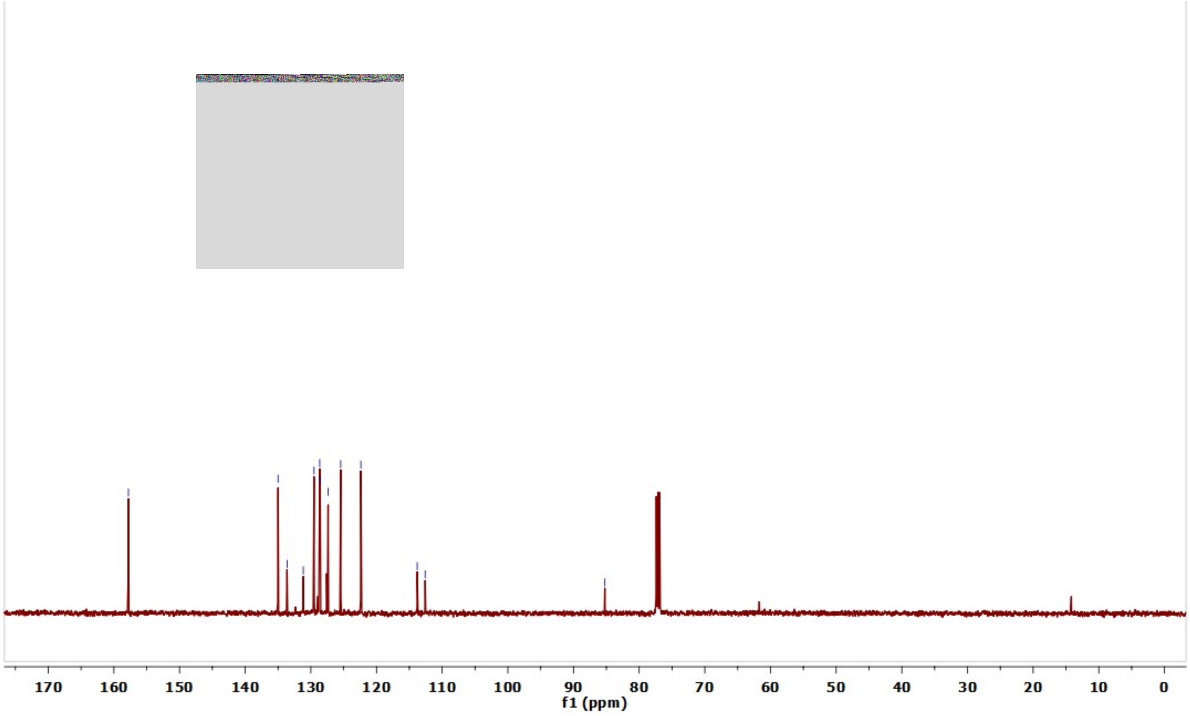

^{13}C {^{1}H} NMR of 1m in $CDCl_3$

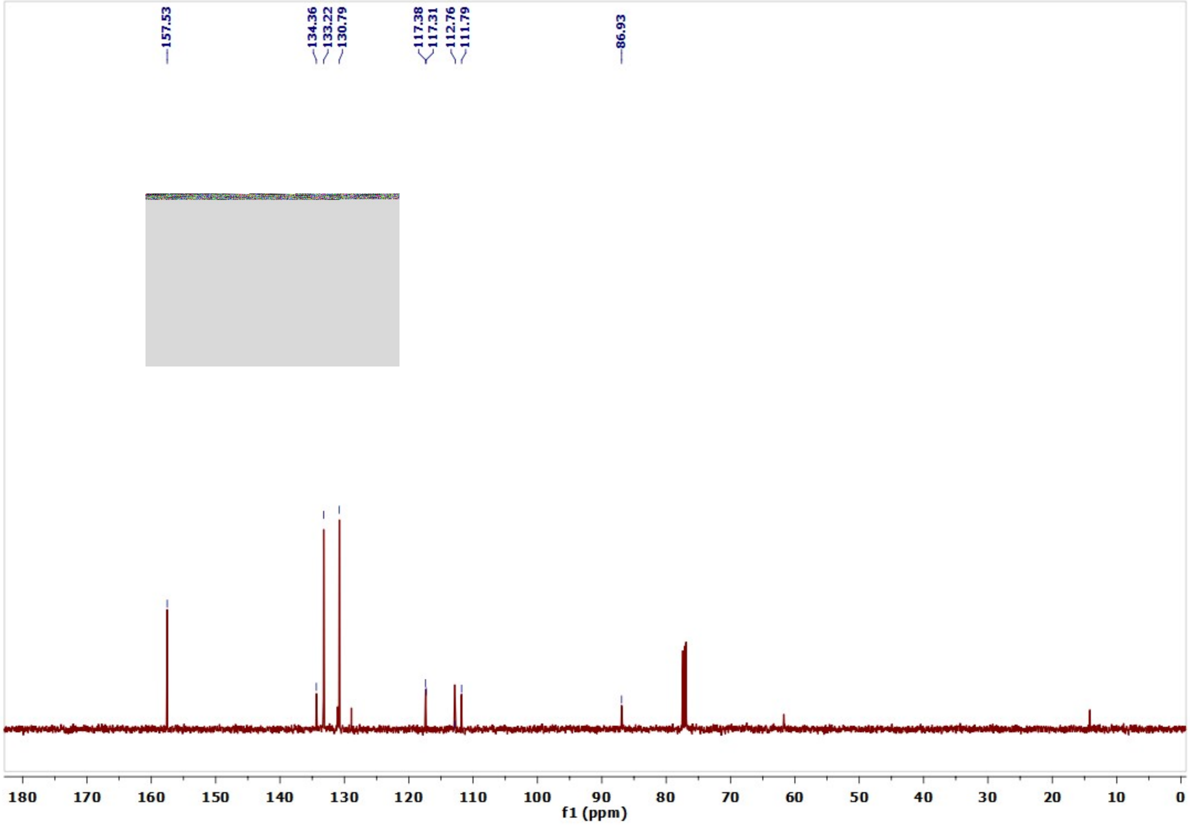

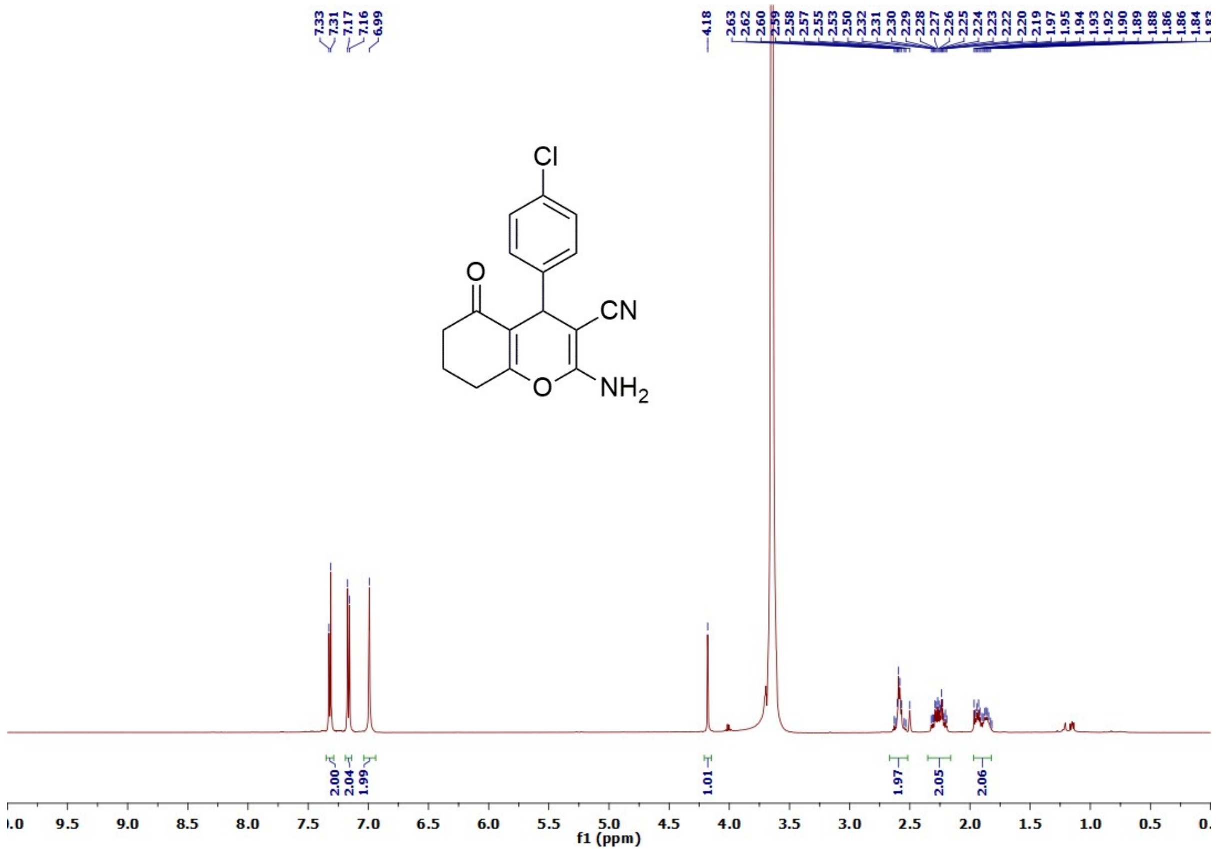

^{1}H-NMR of 2a in DMSO-d_6

^{13}C {^{1}H} NMR of 2a in DMSO-d_6

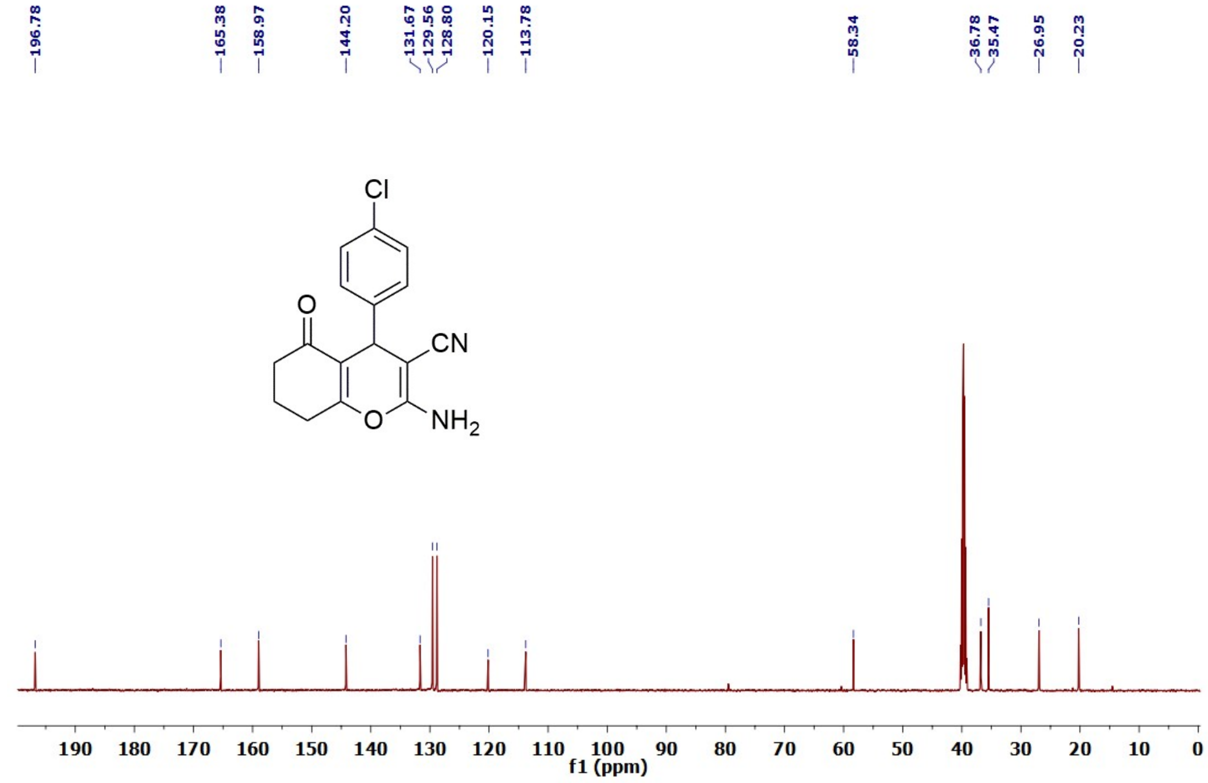

^{1}H-NMR of 2b in DMSO-d_6

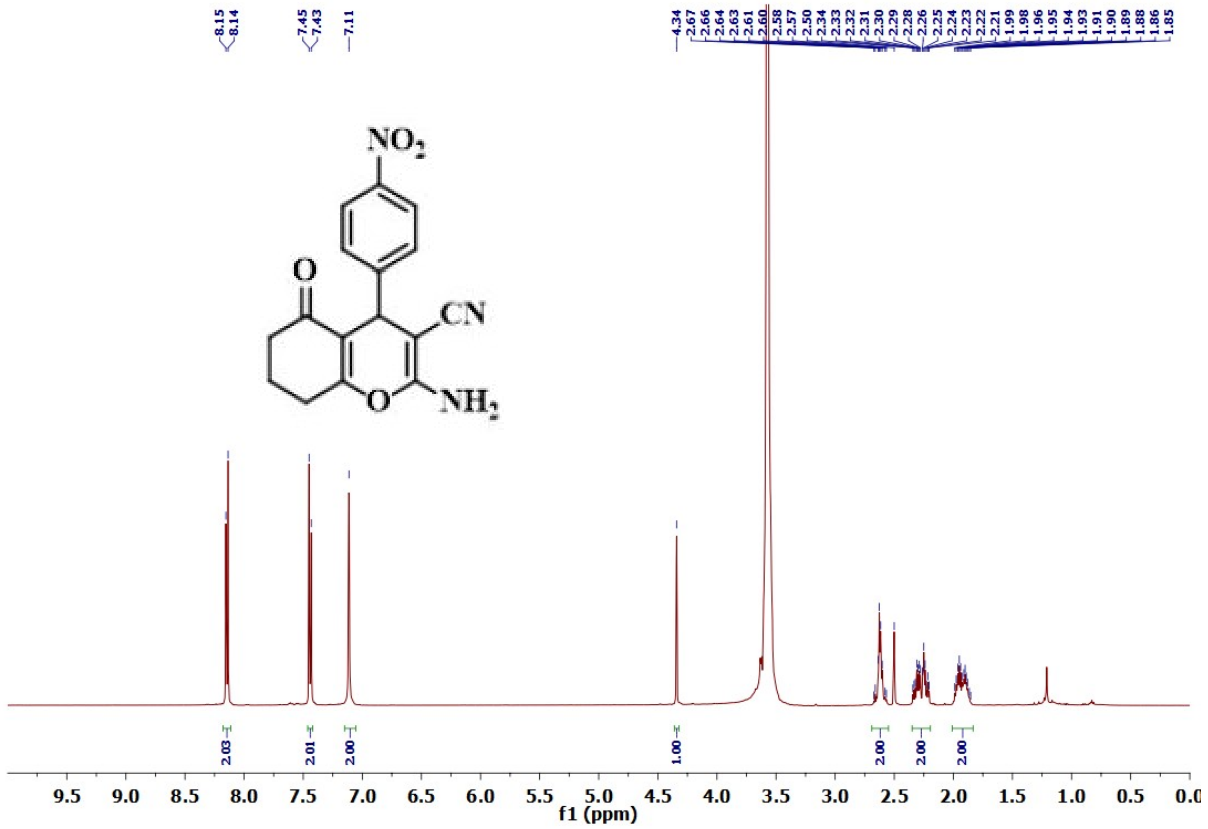

^{13}C {^{1}H} NMR of 2b in DMSO-d_6

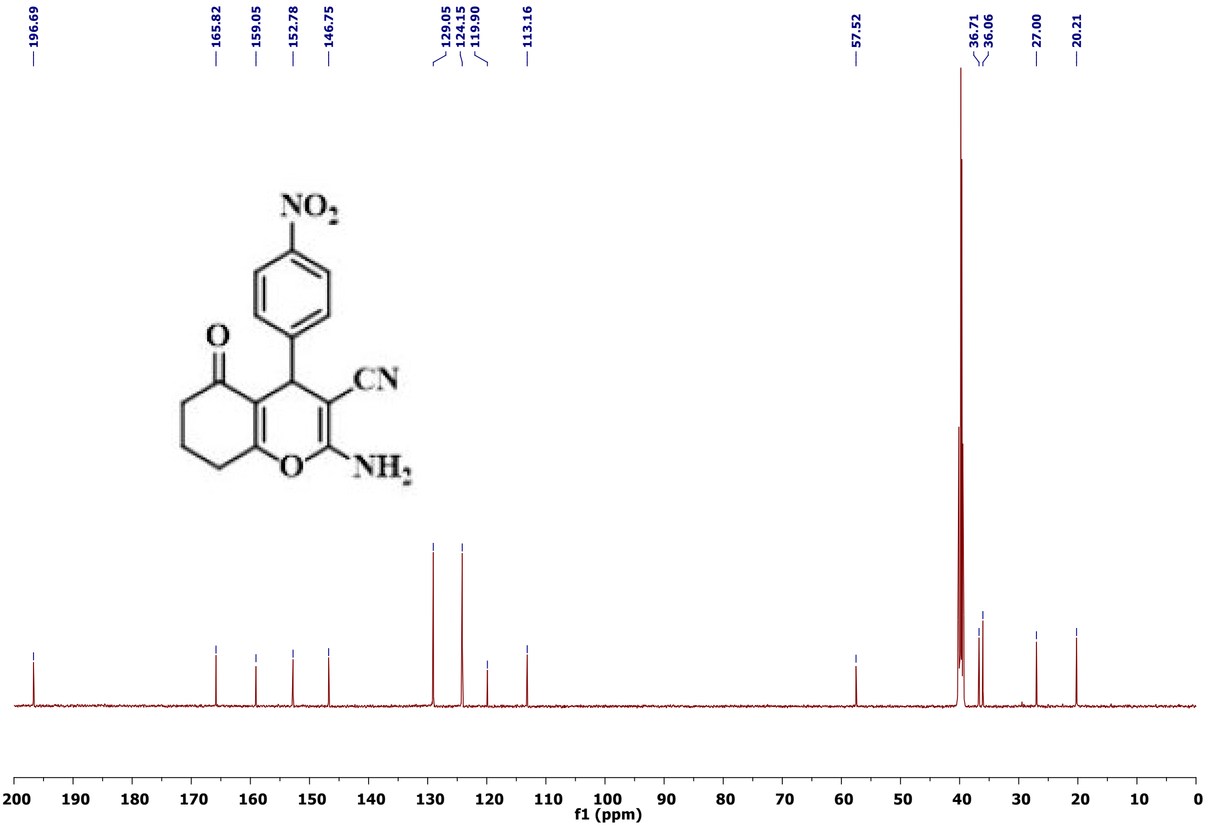

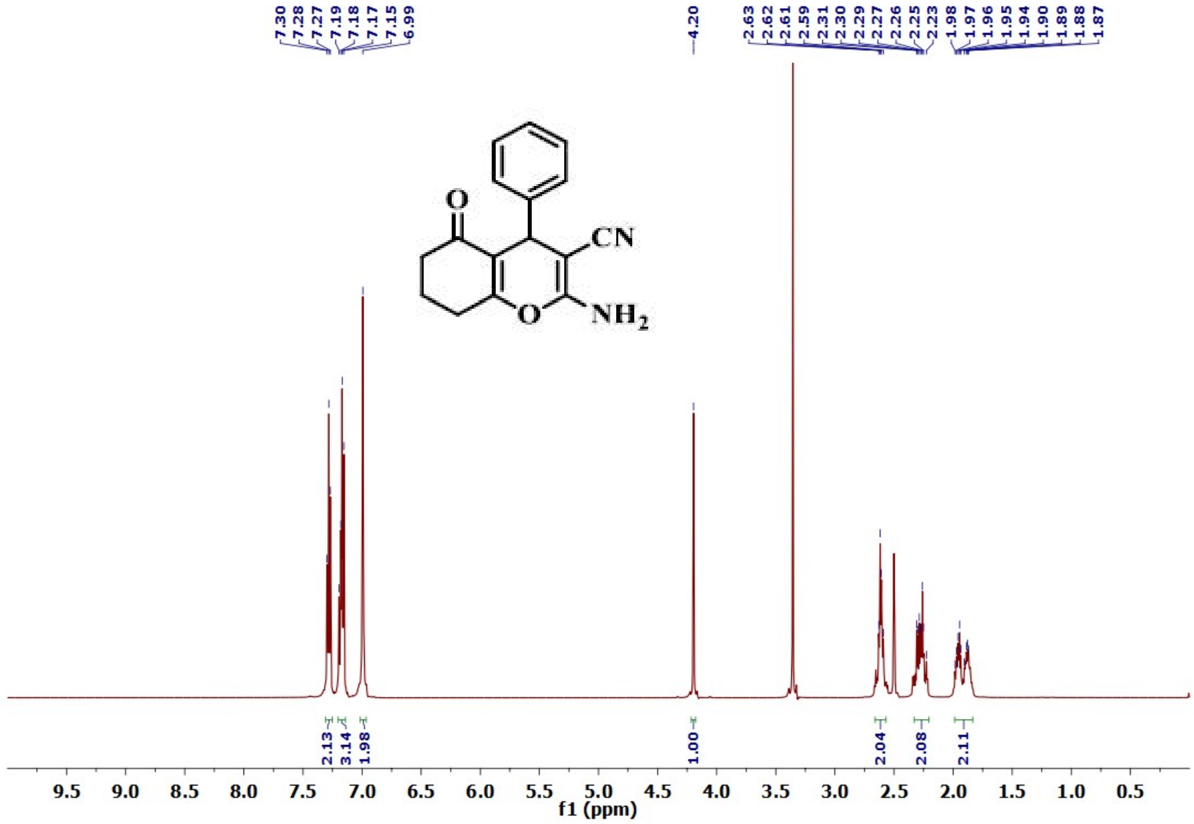

^{1}H-NMR of 2c in DMSO-d_6

^{13}C {^{1}H} NMR of 2c in DMSO-d_6

^{1}H-NMR of 2d in DMSO-d_6

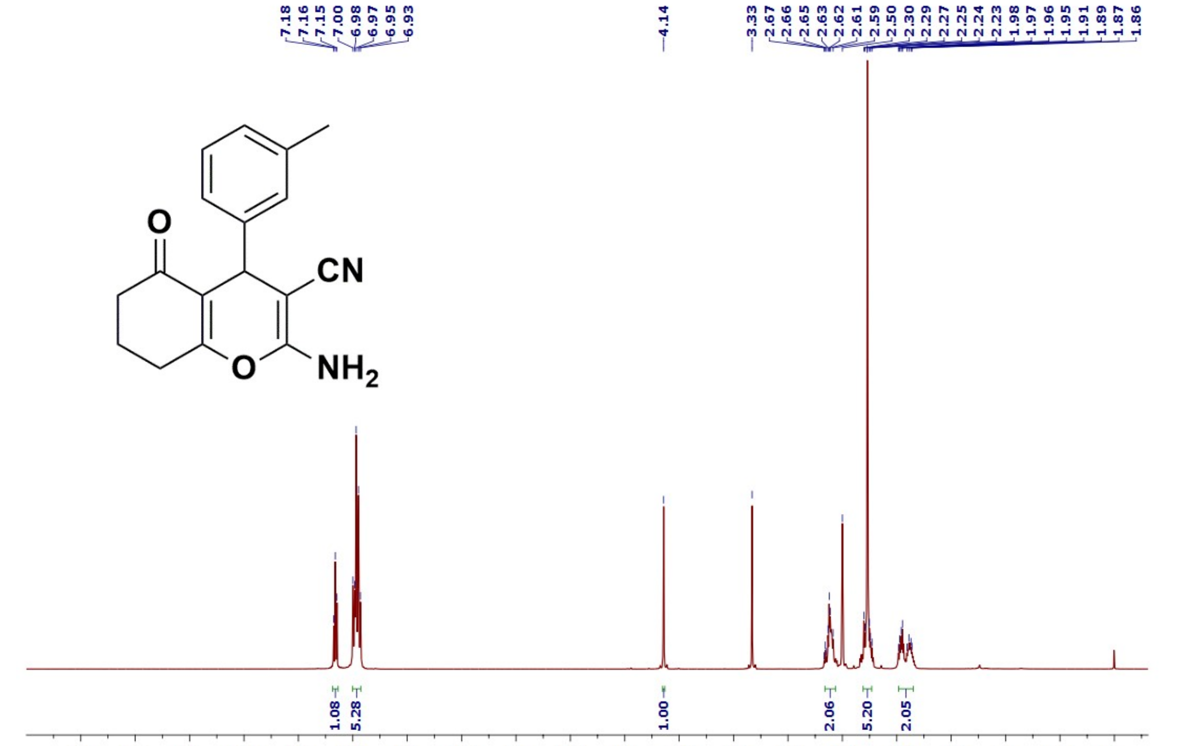

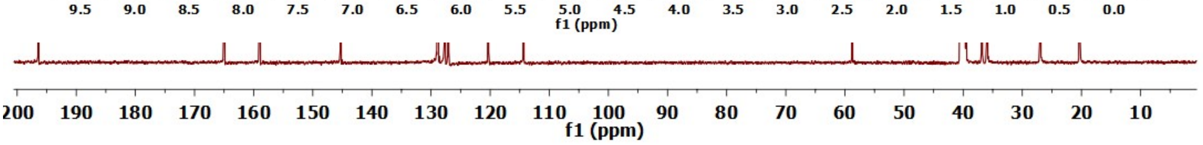

^{13}C {^{1}H} NMR of 2d in DMSO-d_6

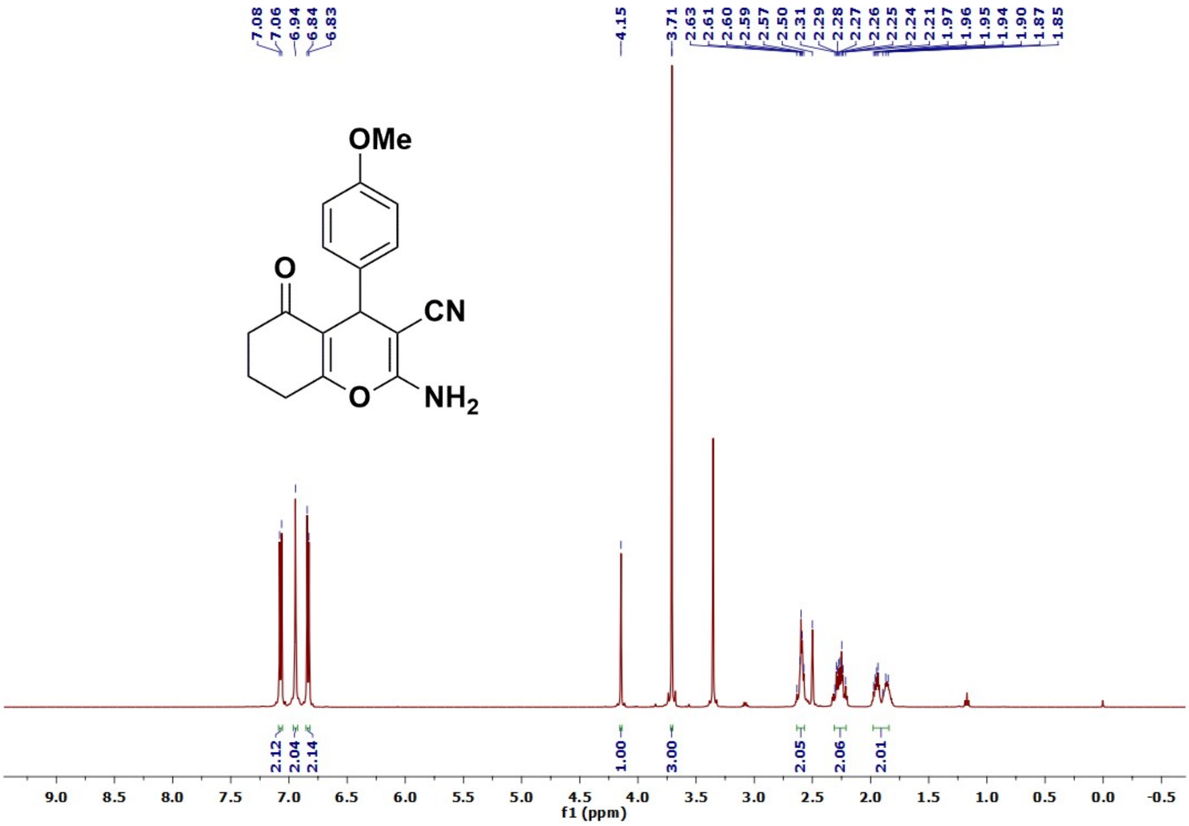

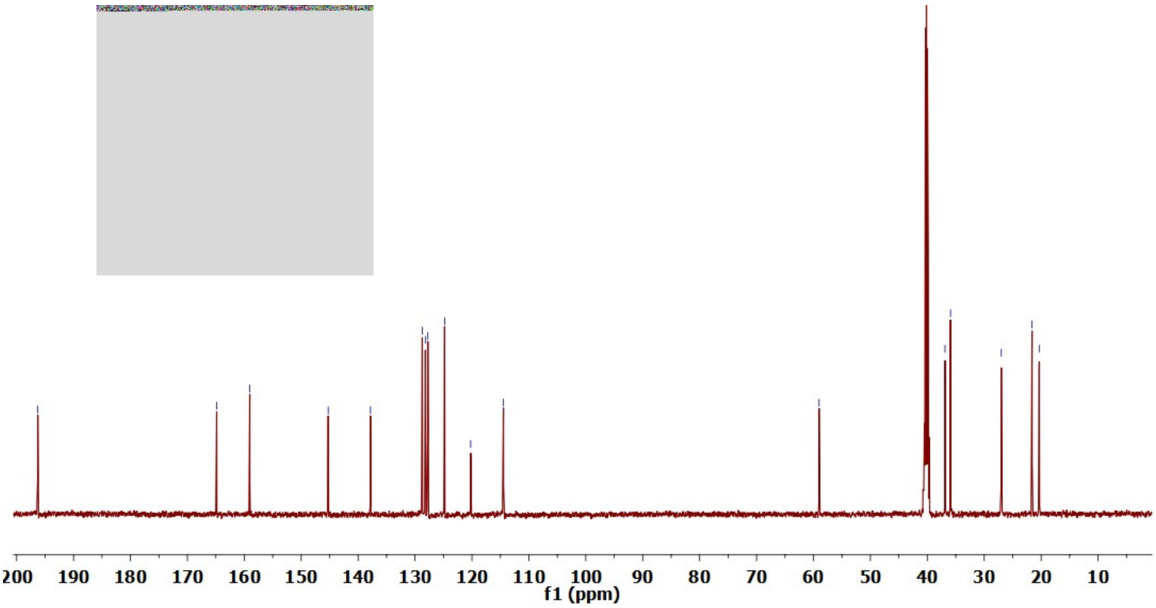

^{1}H-NMR of 2e in DMSO-d_6

$^{13}C\{^1H\}$ NMR of 2e in DMSO-d_6

^1H-NMR of 2f in DMSO-d_6

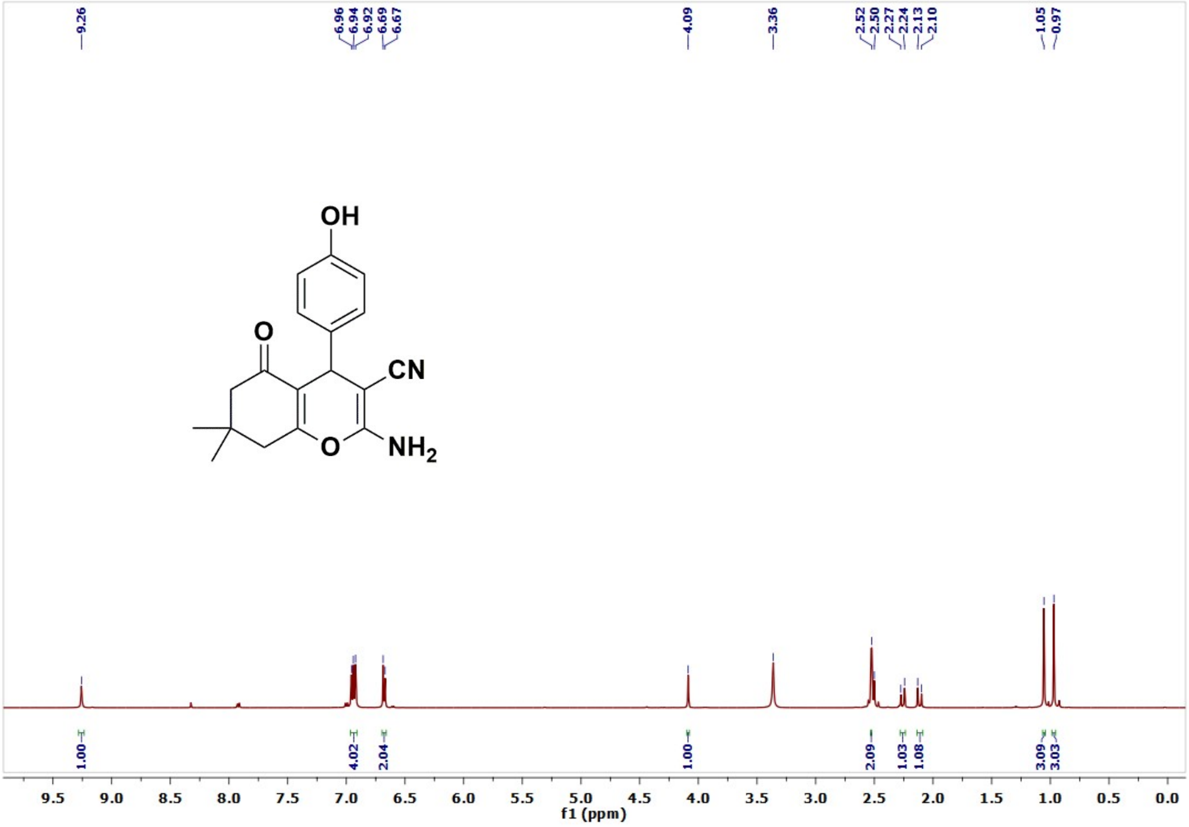

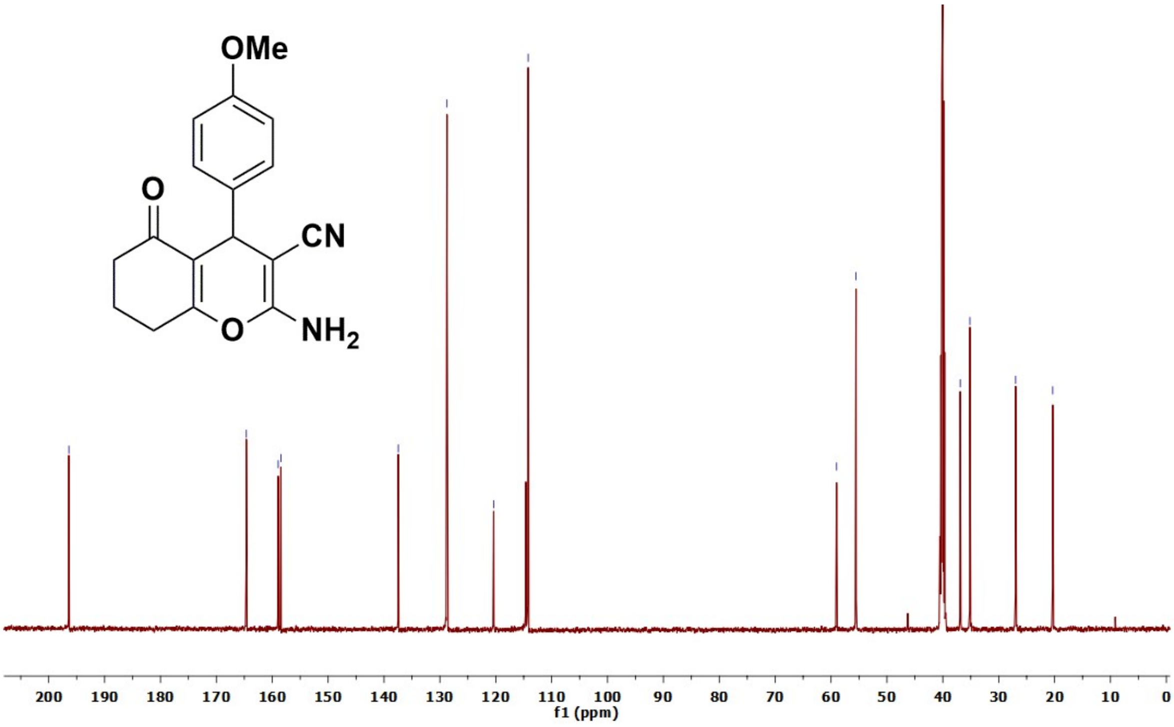

^{13}C {^{1}H} NMR of 2f in DMSO-*d*$_6$

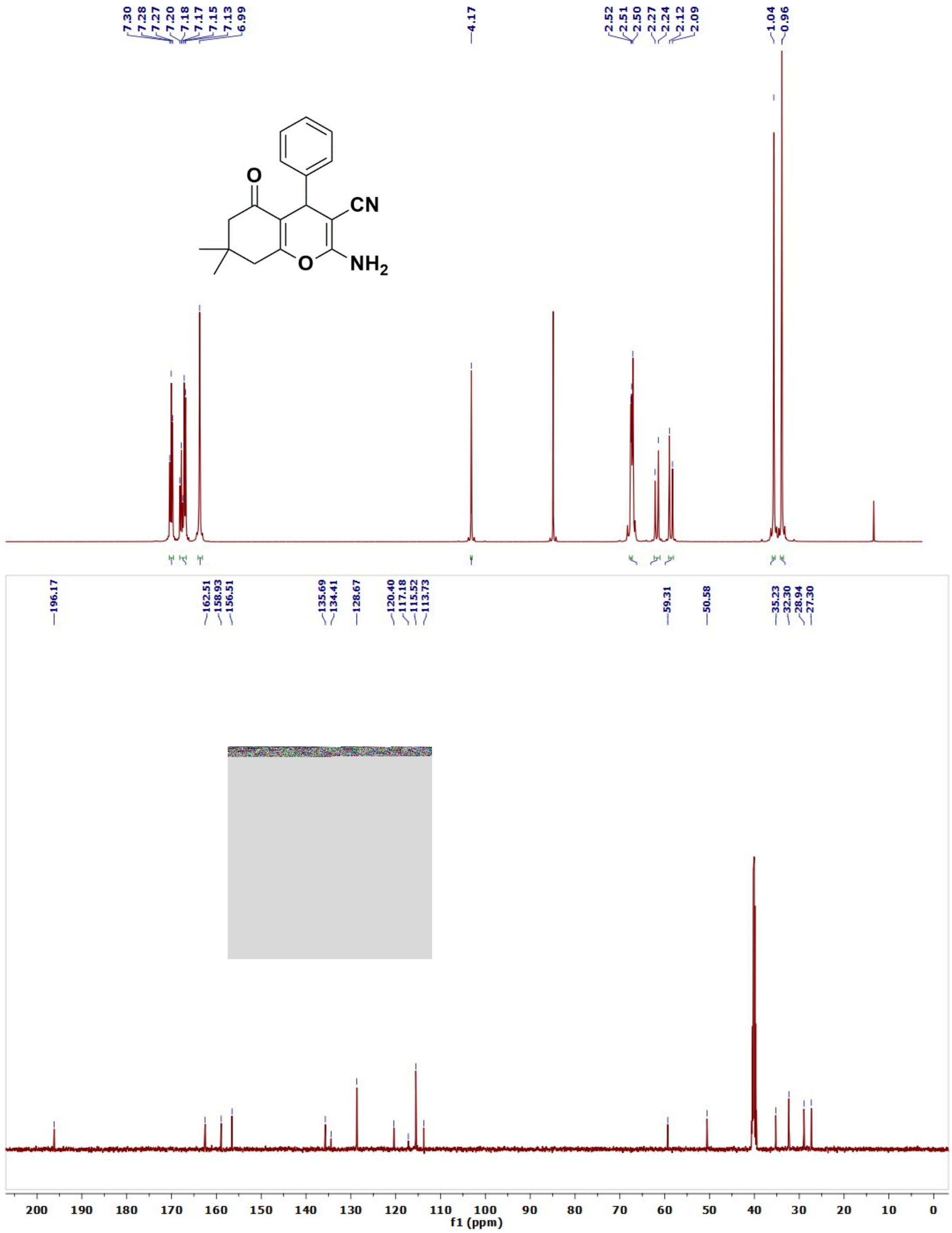

^{1}H-NMR of 2g in DMSO-*d*$_6$

^{13}C {^{1}H} NMR of 2g in DMSO-d_6

^{1}H-NMR of 2h in DMSO-d_6

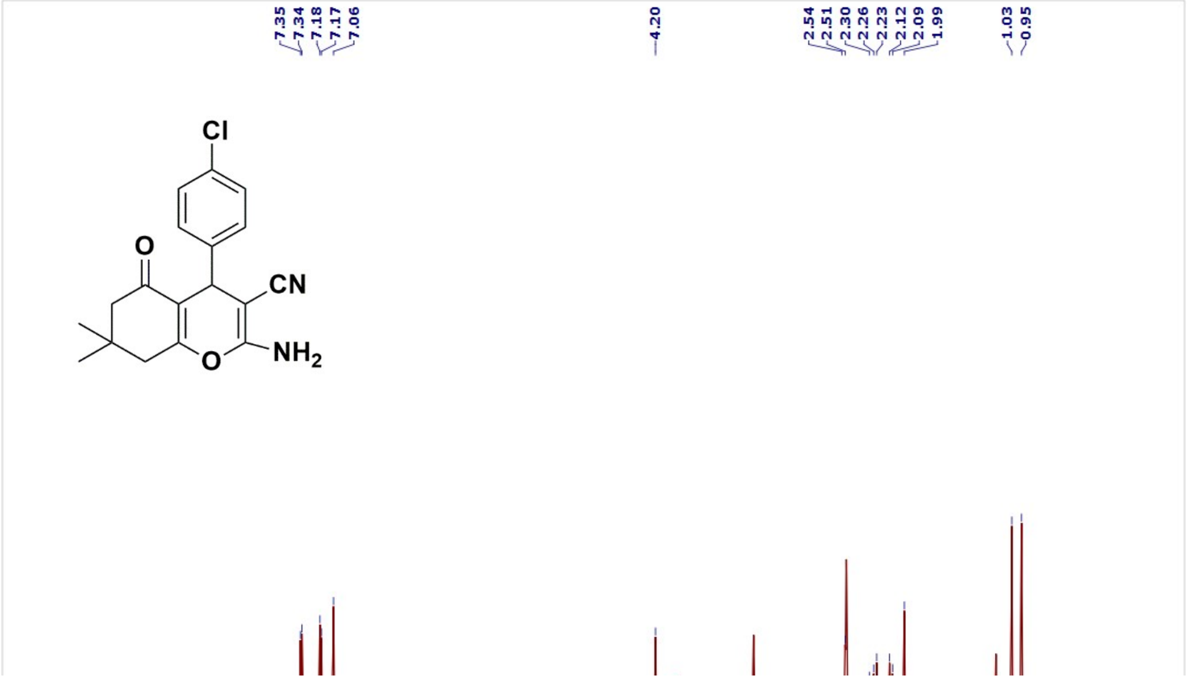

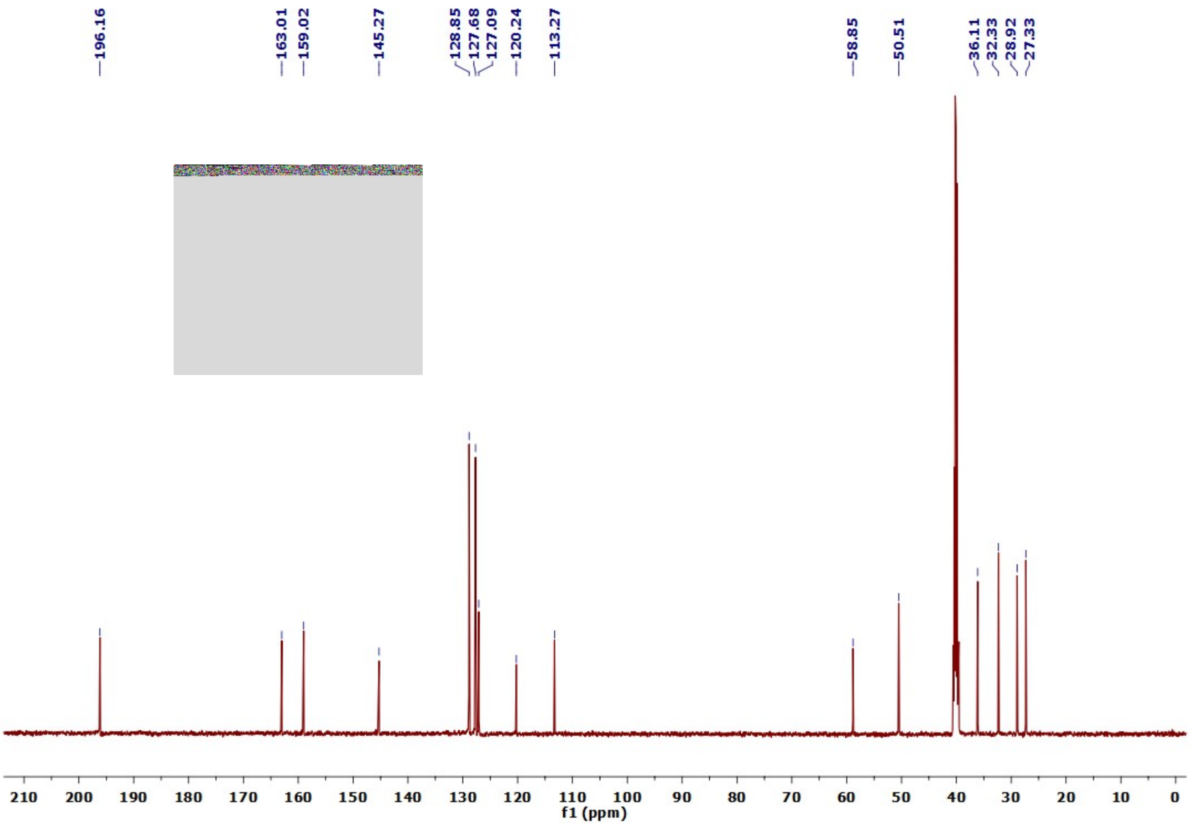

^{13}C {^{1}H} NMR of 2h in DMSO-d_6

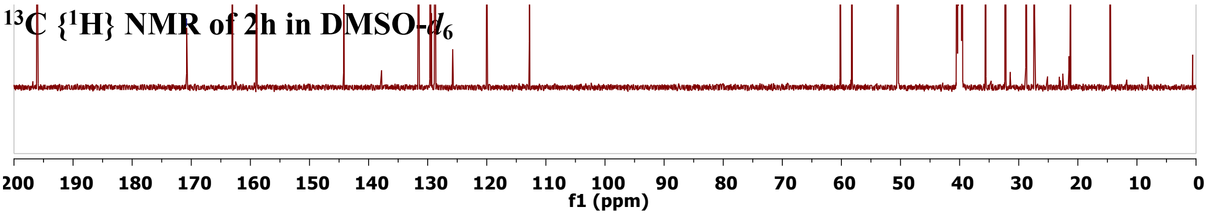

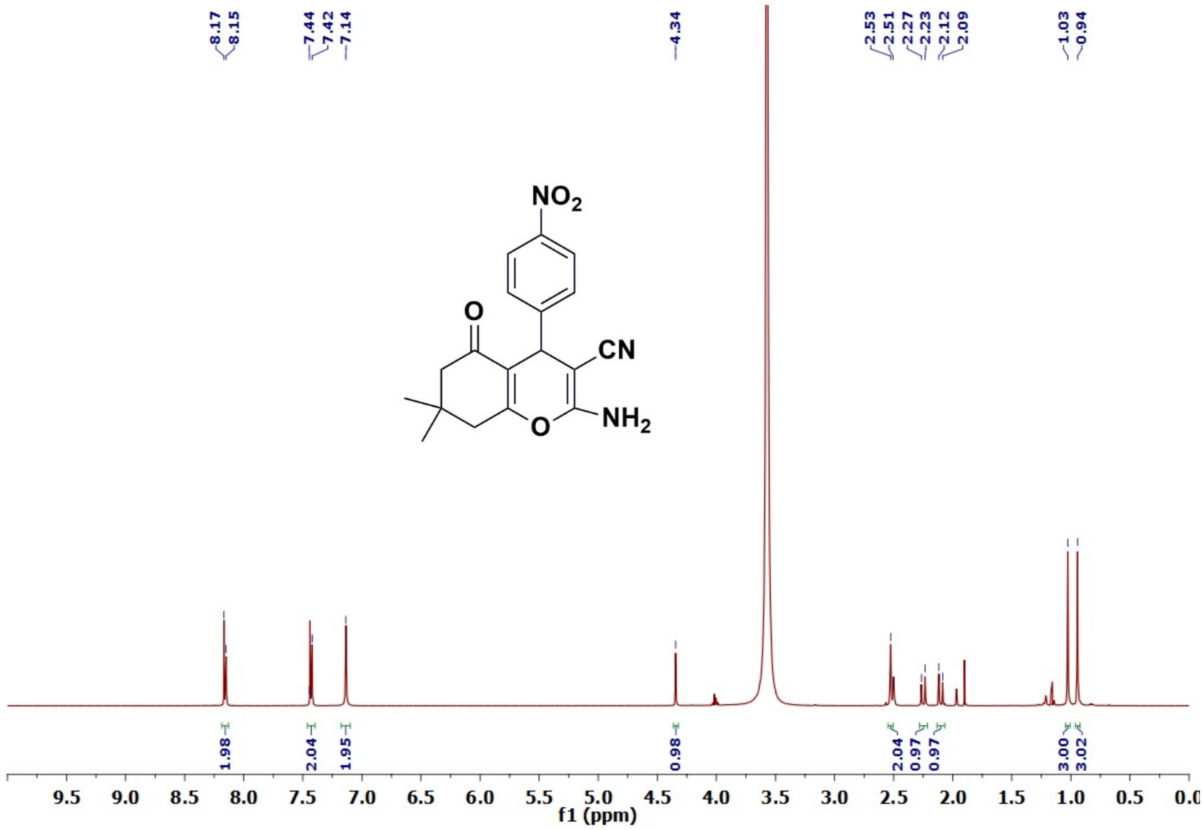

^{1}H-NMR of 2i in DMSO-d_6

^{13}C {^{1}H} NMR of 2i in DMSO-d_6

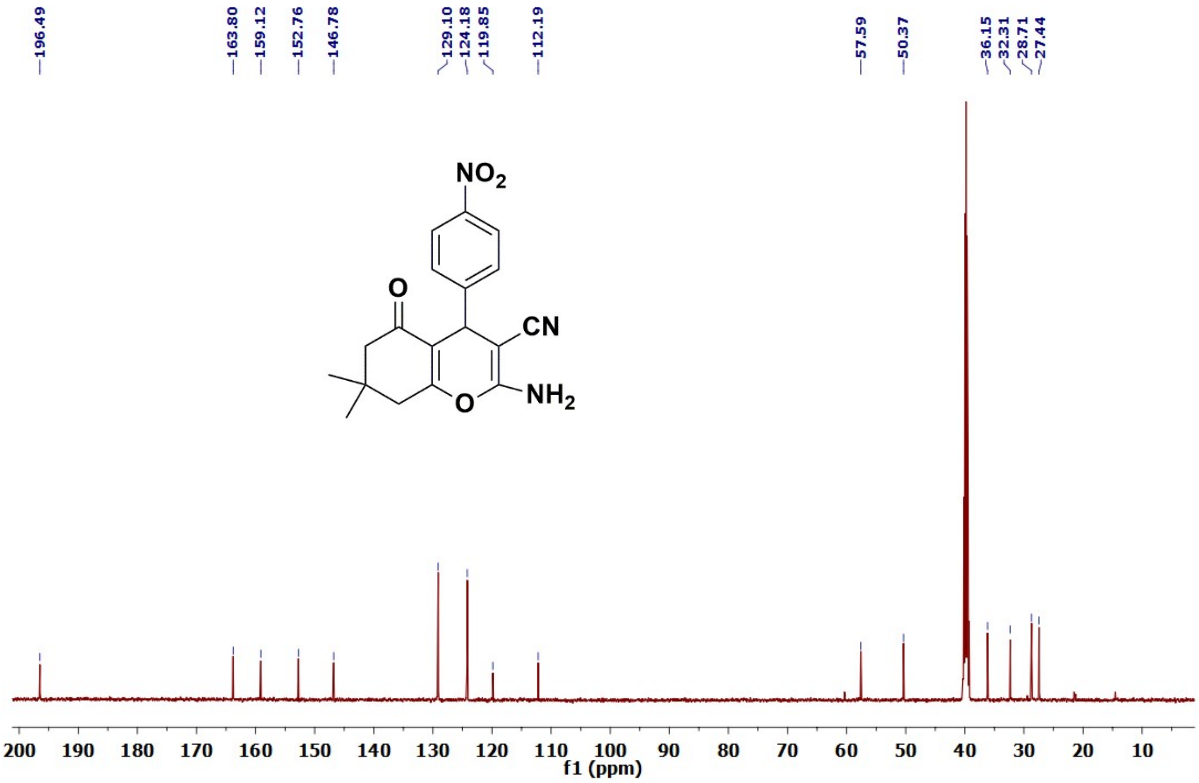

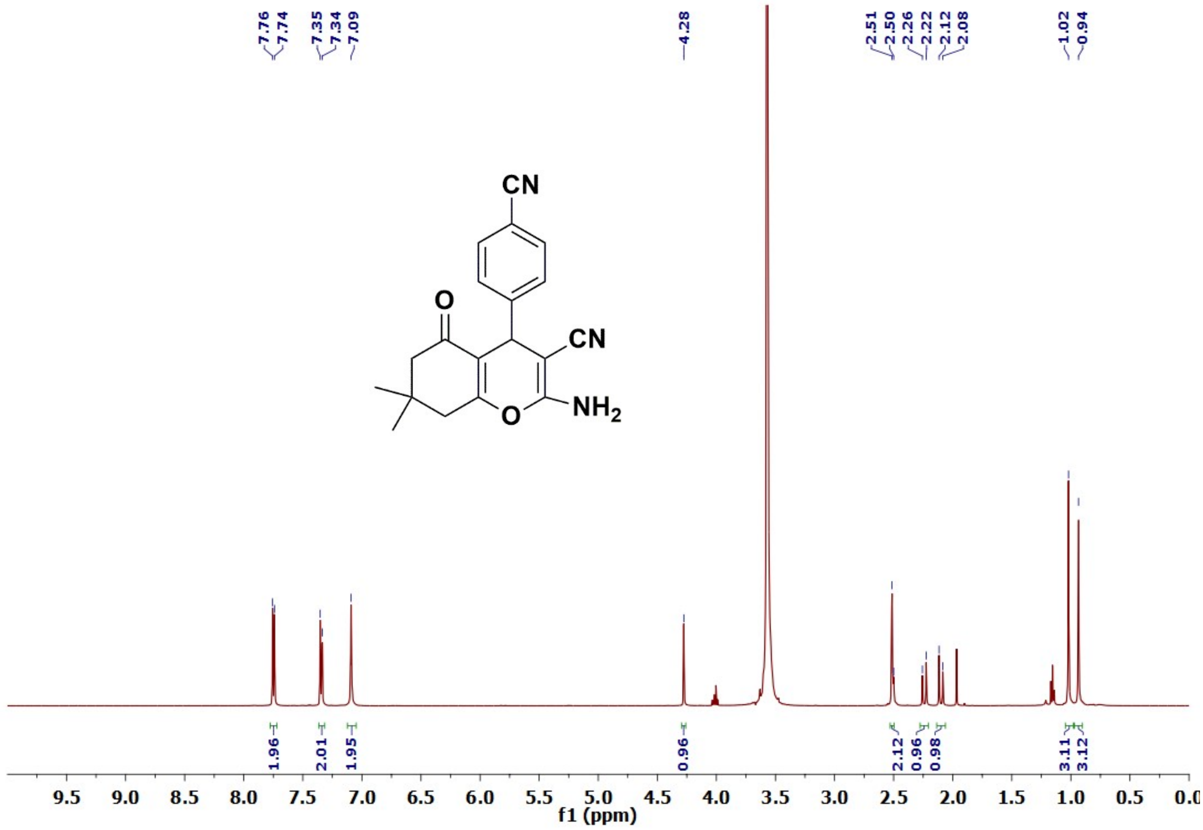

^{13}C {^{1}H} NMR of 2j in DMSO-*d*$_6$

^{13}C {^{1}H} NMR of 2j in DMSO-*d*$_6$

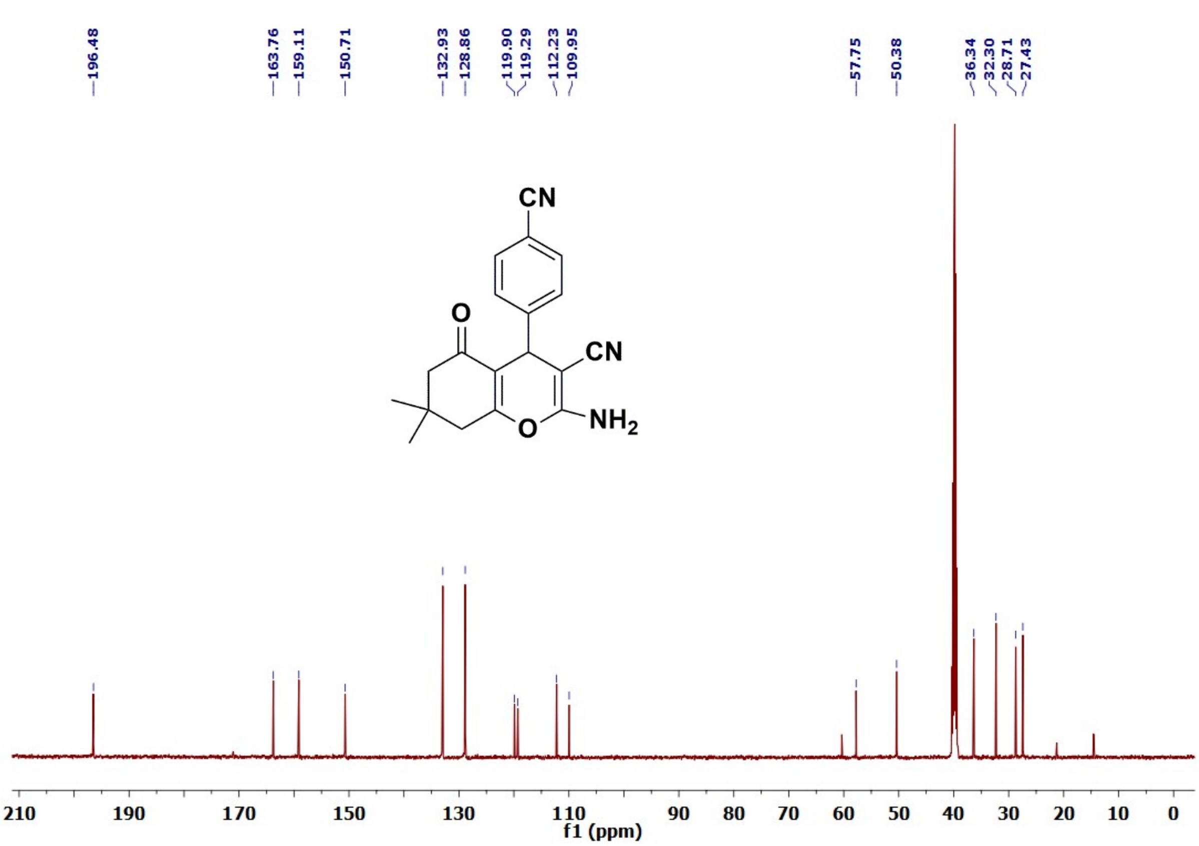

^{1}H-NMR of 3a in DMSO-d_6

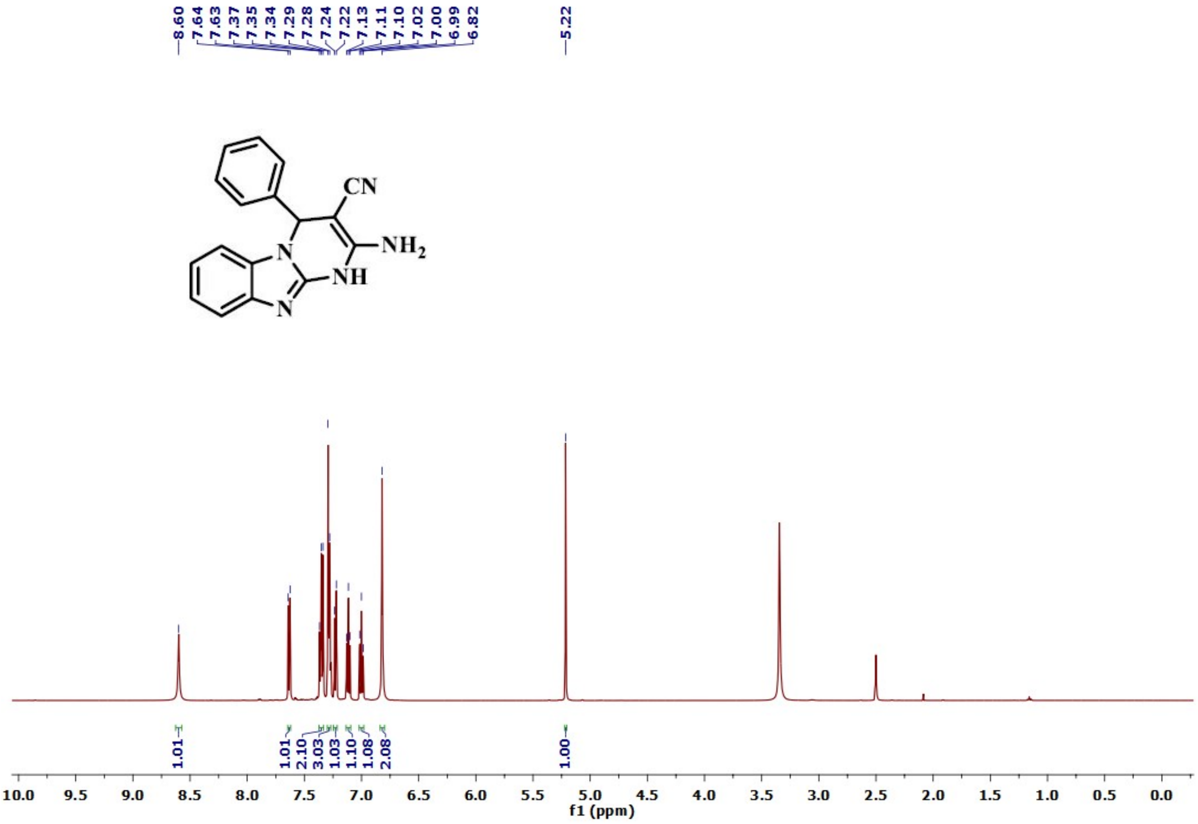

^{13}C {^{1}H} NMR of 3a in DMSO-d_6

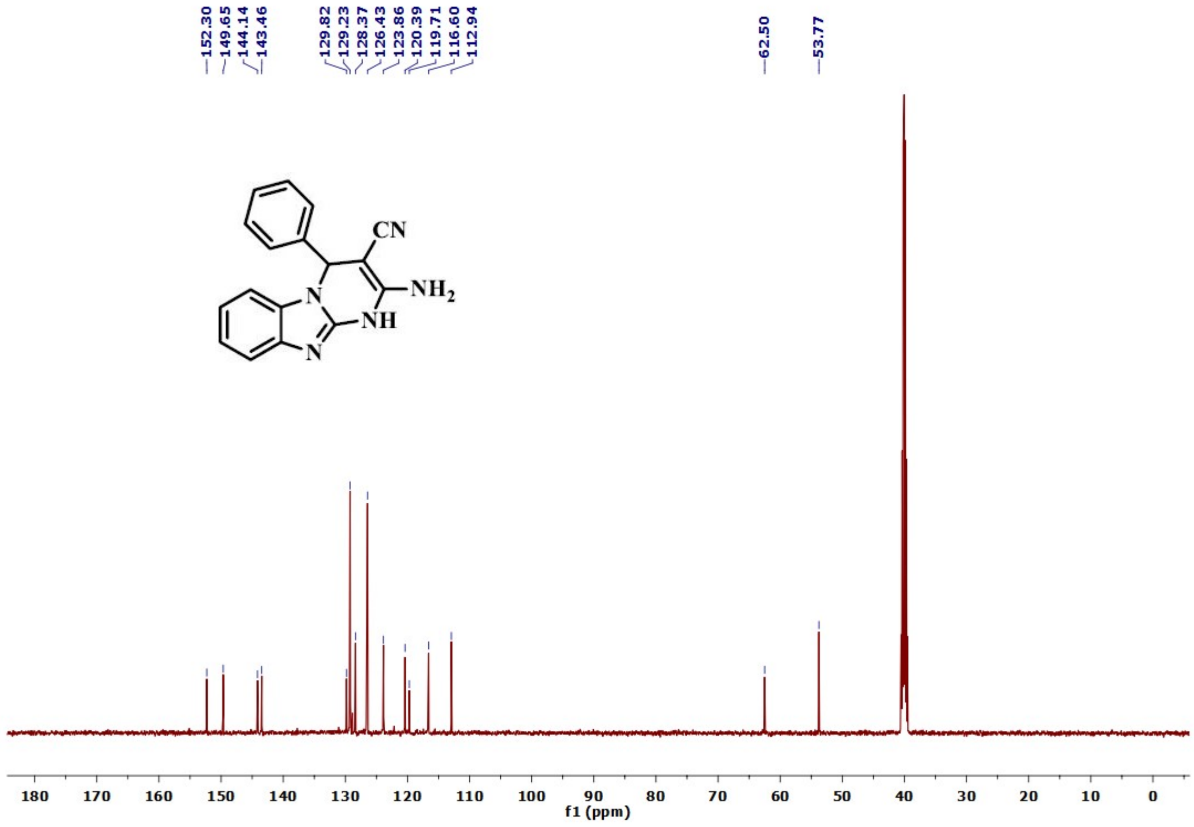

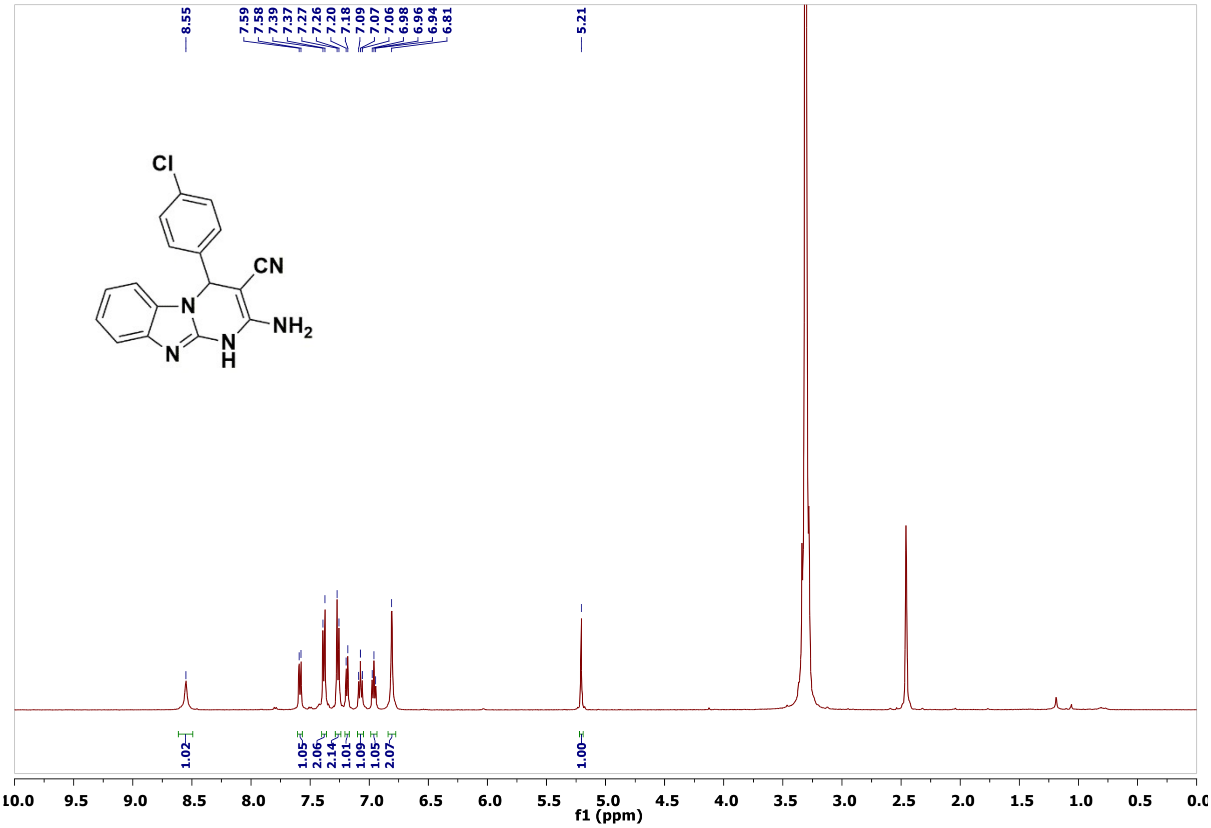

^{1}H-NMR of 3b in DMSO-d_6

^{13}C {^{1}H} NMR of 3b in DMSO-d_6

^{1}H-NMR of 3c in DMSO-d_6

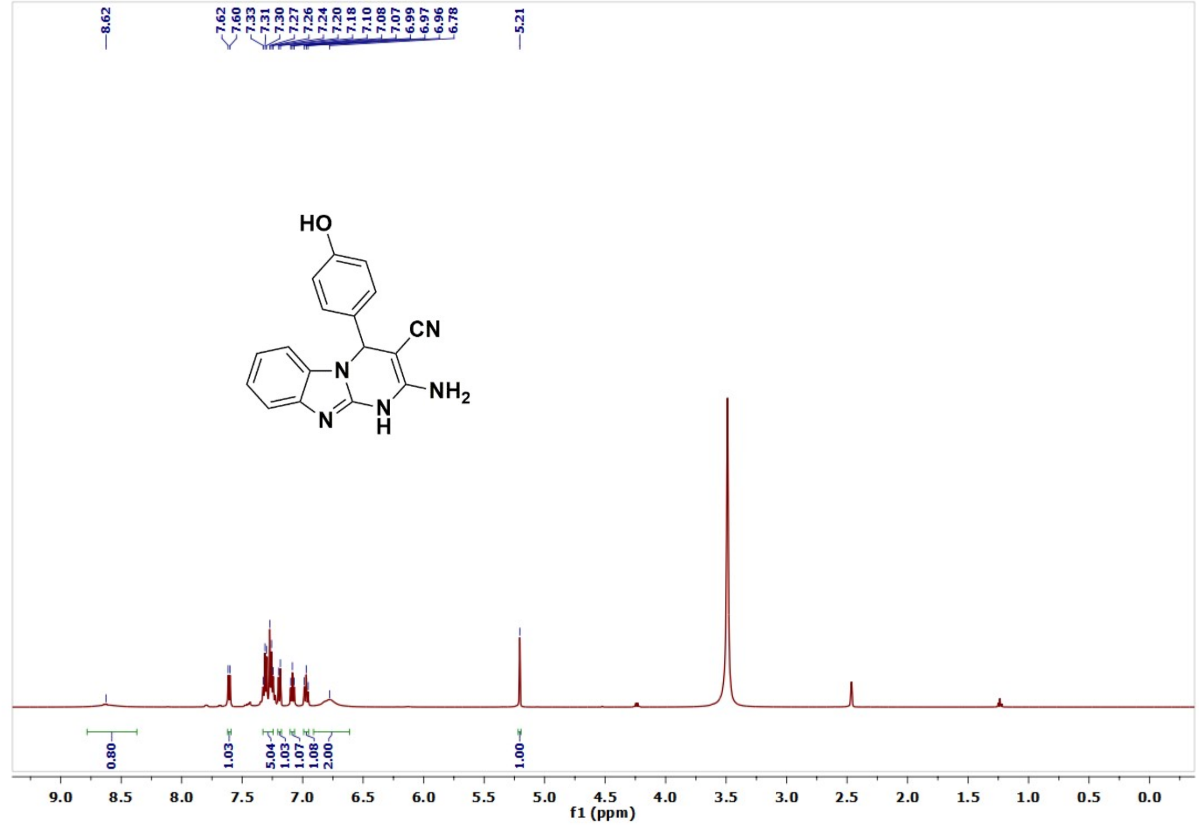

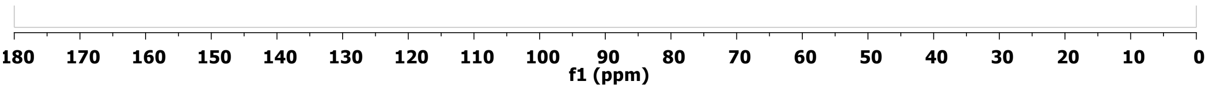

^{13}C {^{1}H} NMR of 3c in DMSO-d_6

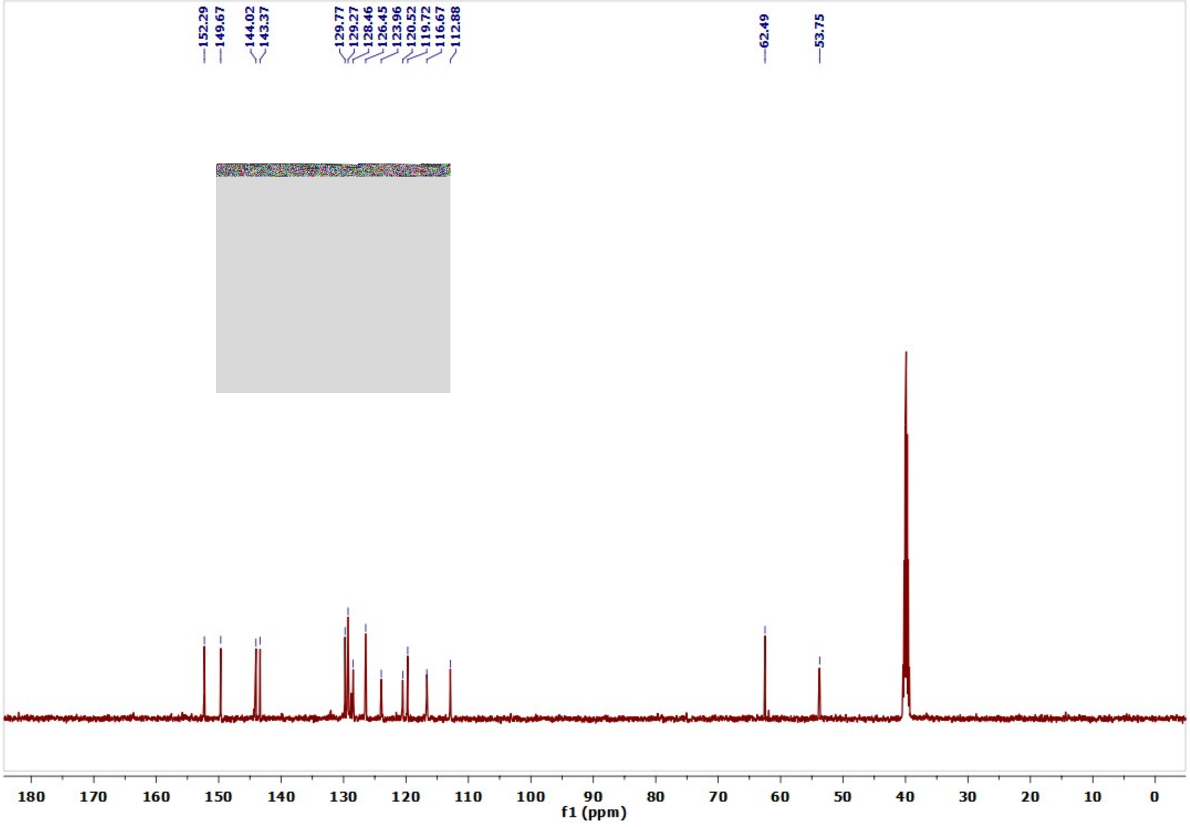

^{1}H-NMR of 3d in DMSO-*d*$_6$

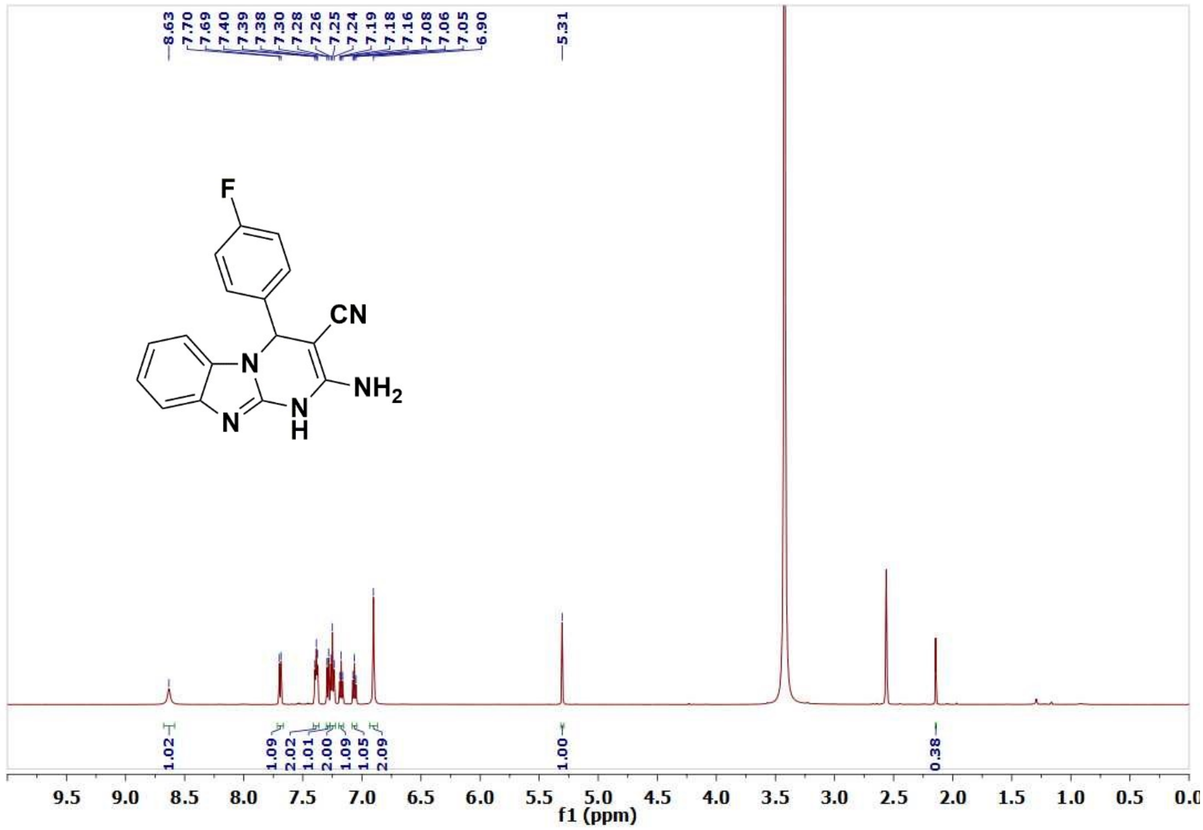

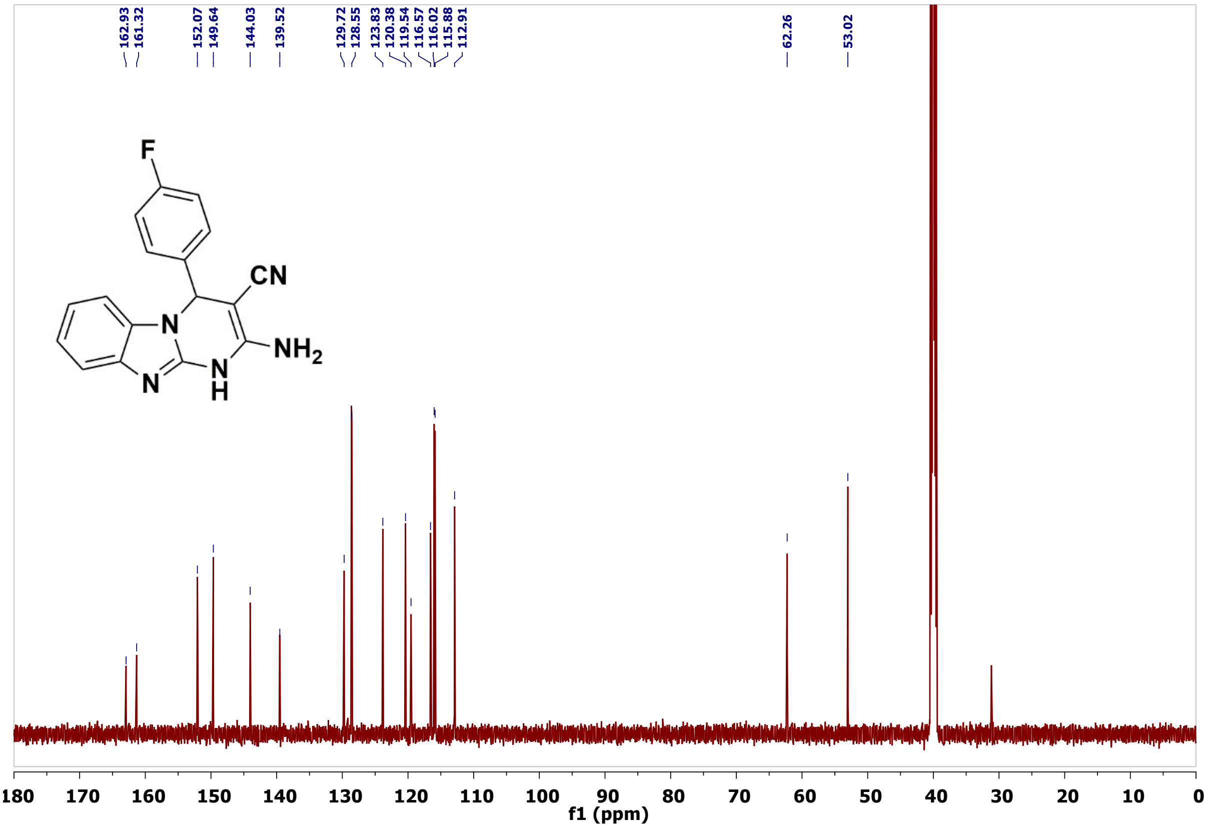

^{13}C {^{1}H} NMR of 3d in DMSO-*d*$_6$

^{1}H-NMR of 3e in DMSO-d_6

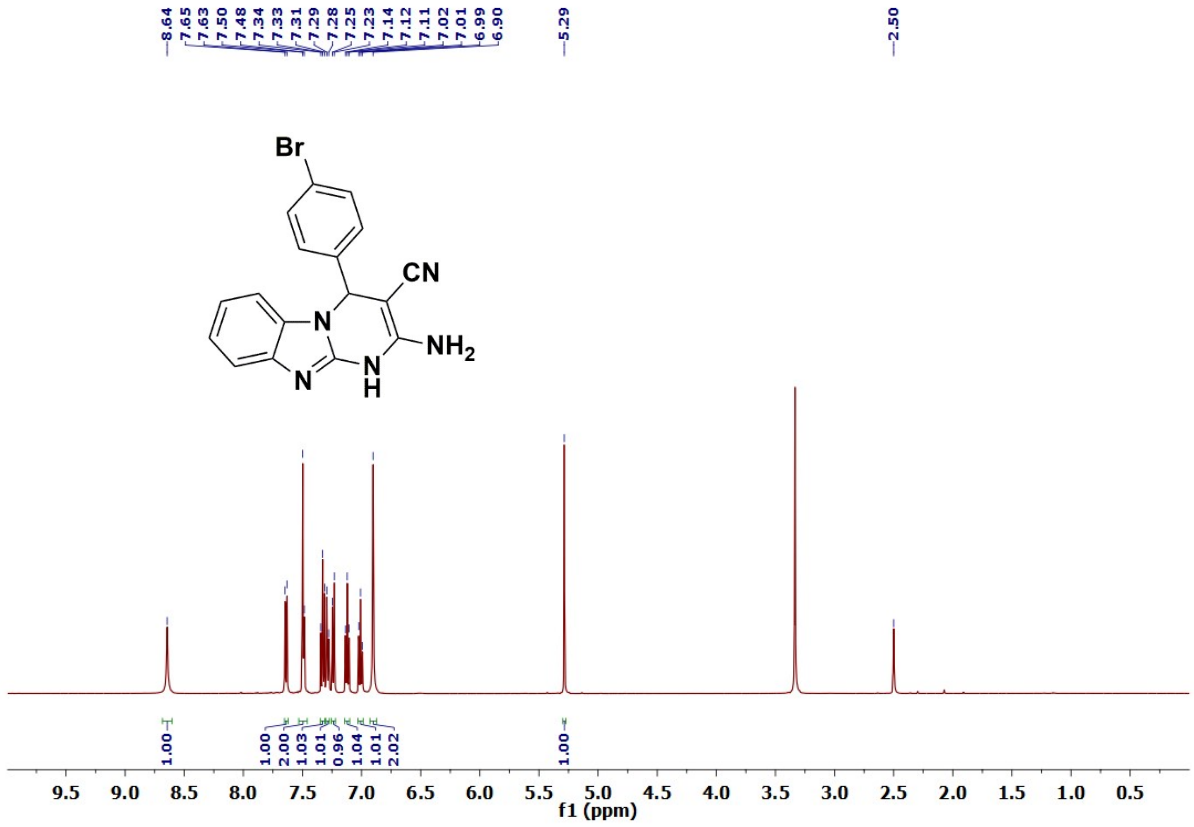

^{13}C {^{1}H} NMR of 3e in DMSO-d_6

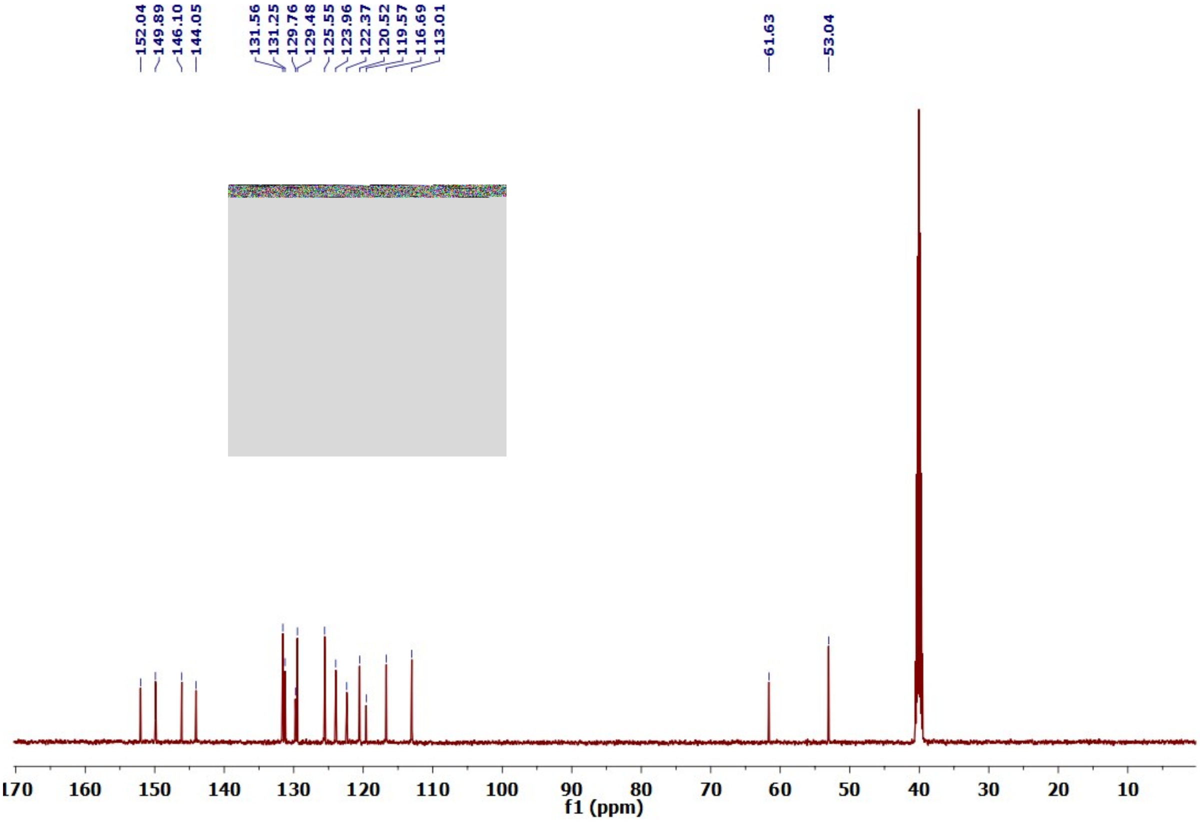

^{1}H-NMR of 3f in DMSO-*d*$_6$

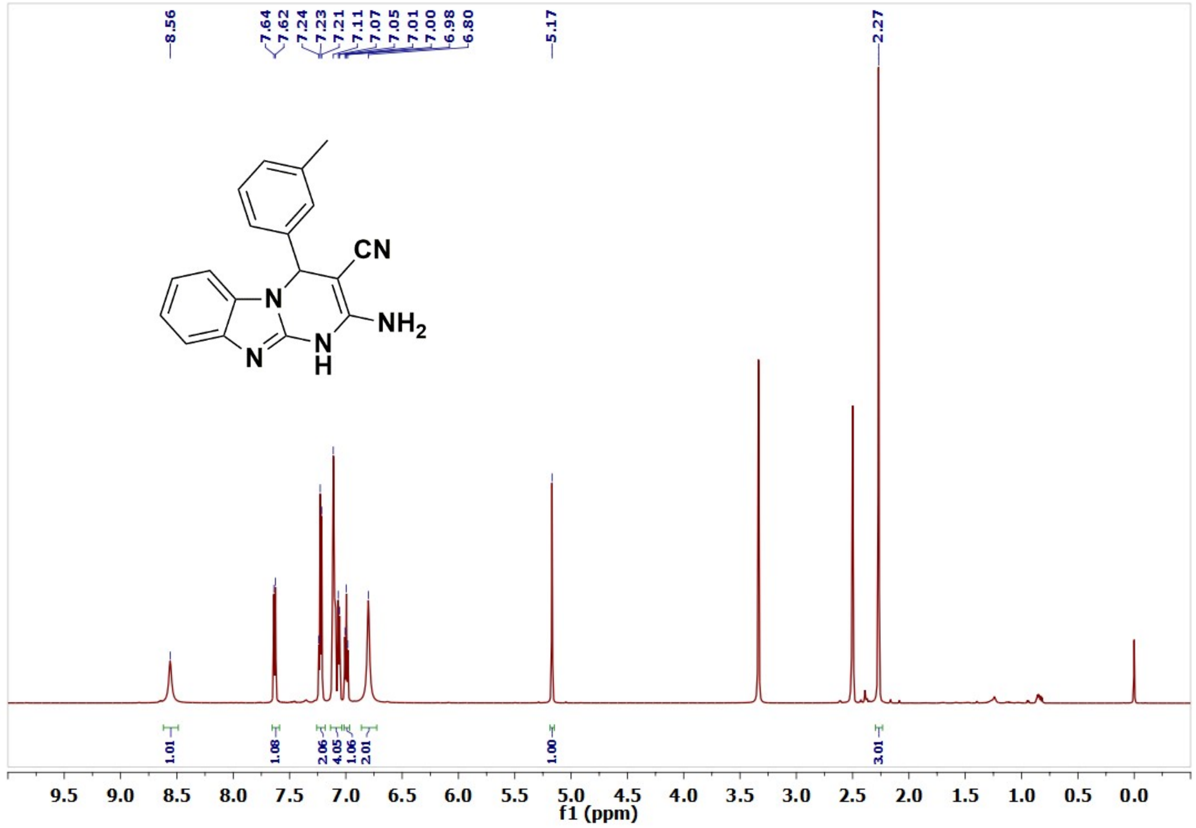

^{13}C {^{1}H} NMR of 3f in DMSO-*d*$_6$

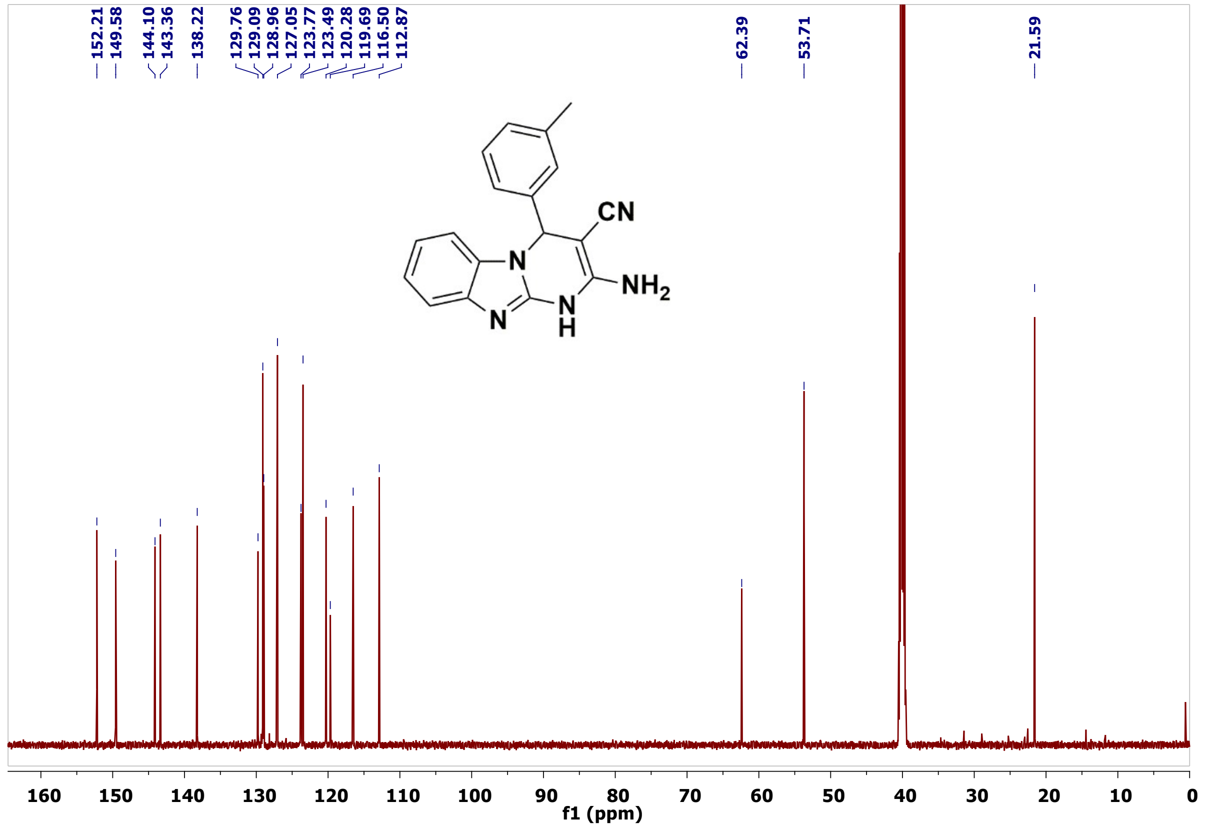

^{1}H-NMR of 3g in DMSO-d_6

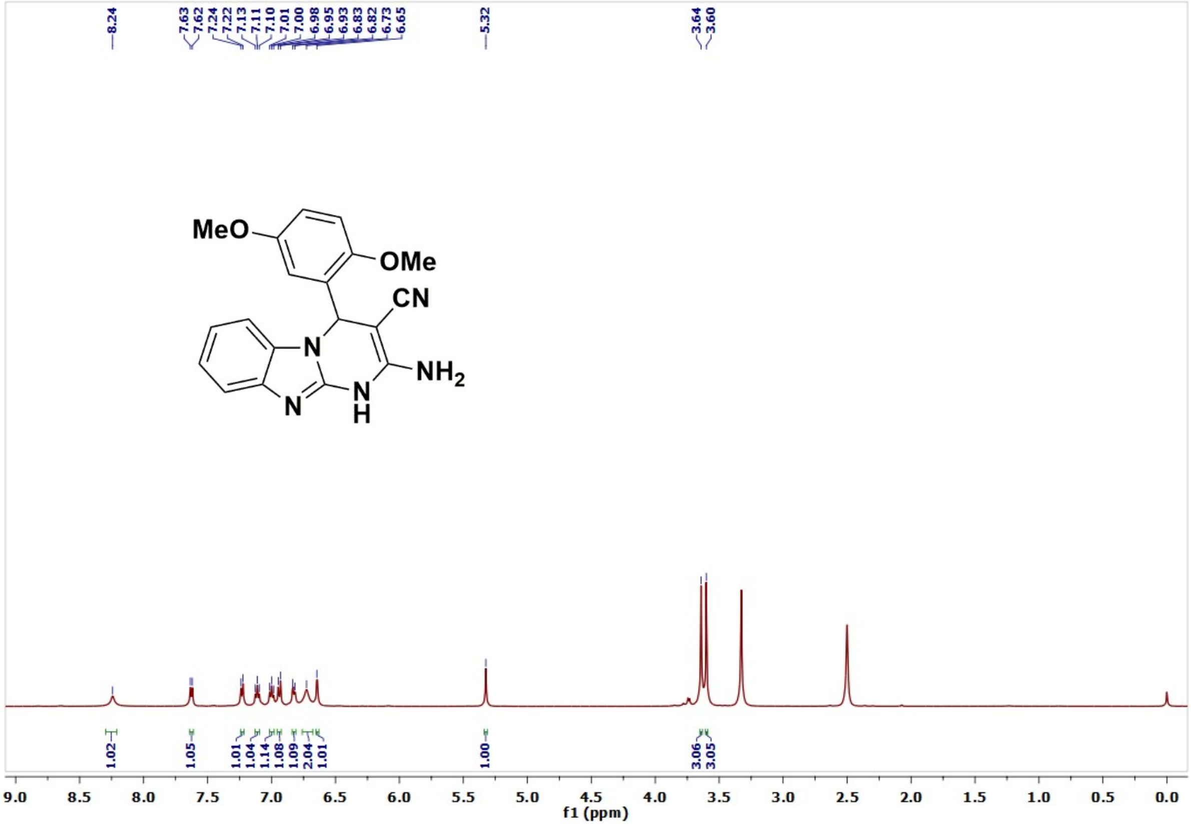

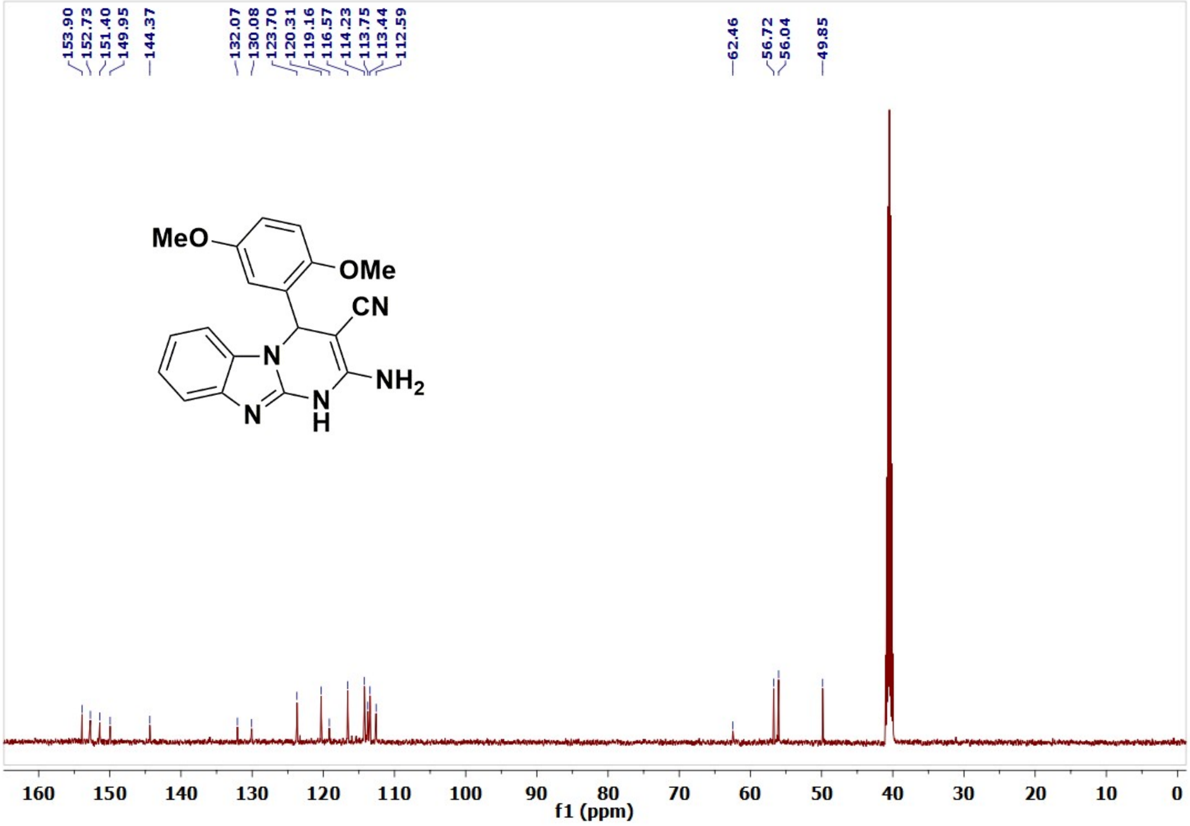

^{13}C {^{1}H} NMR of 3g in DMSO-d_6

^{1}H-NMR of 3h in DMSO-d_6

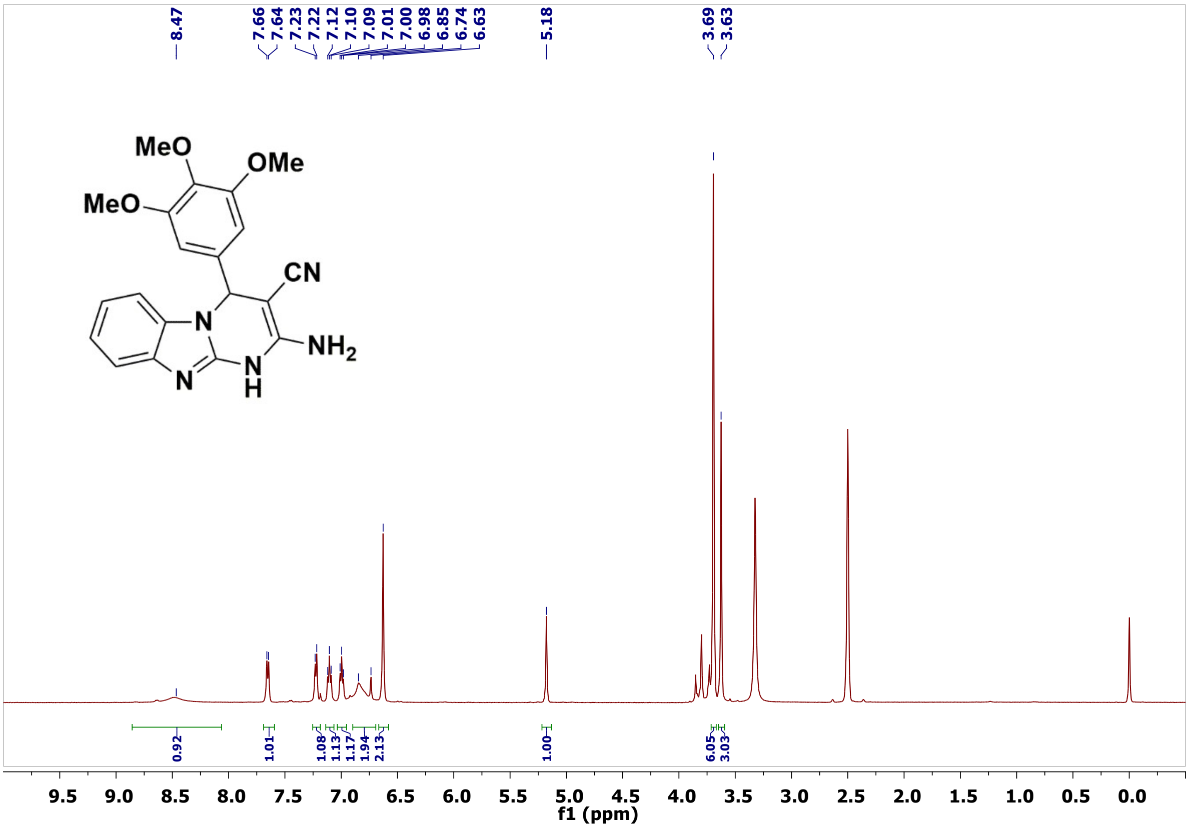

^{13}C {^{1}H} NMR of 3h in DMSO-d_6

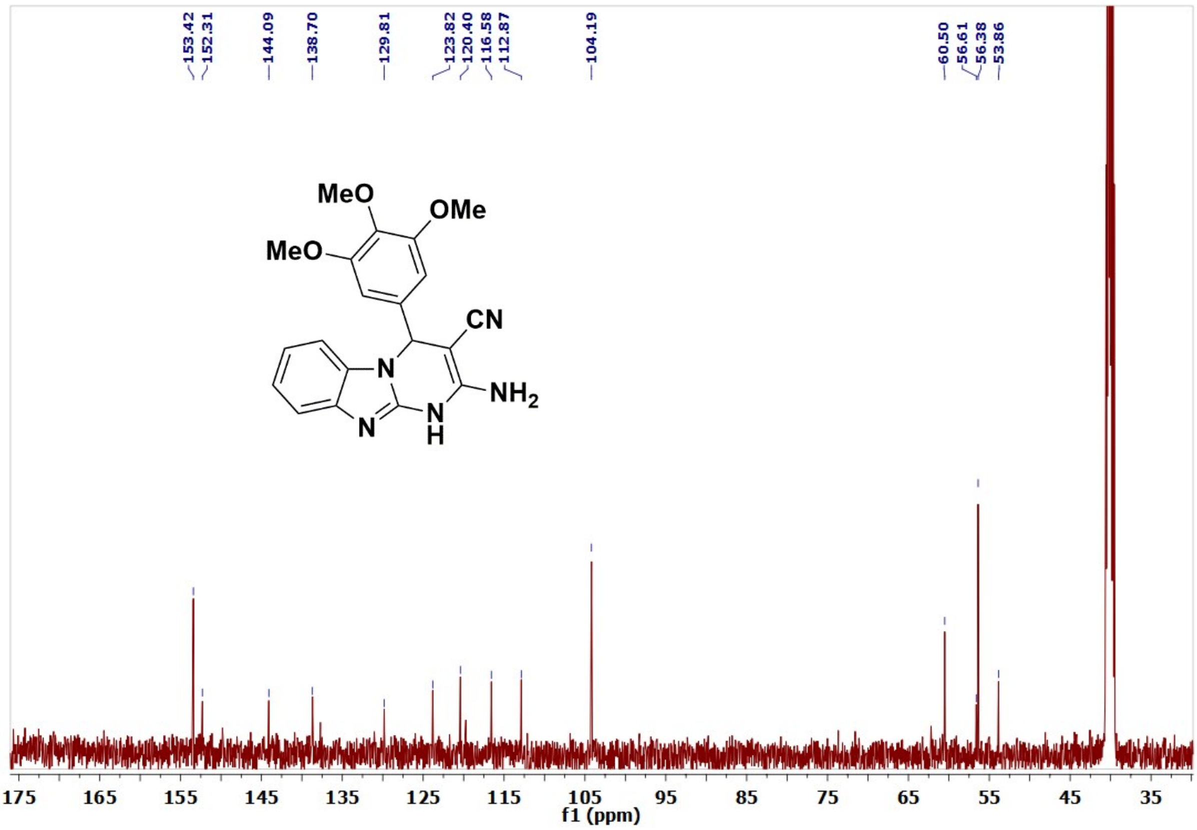

^{1}H-NMR of 3i in DMSO-d_6

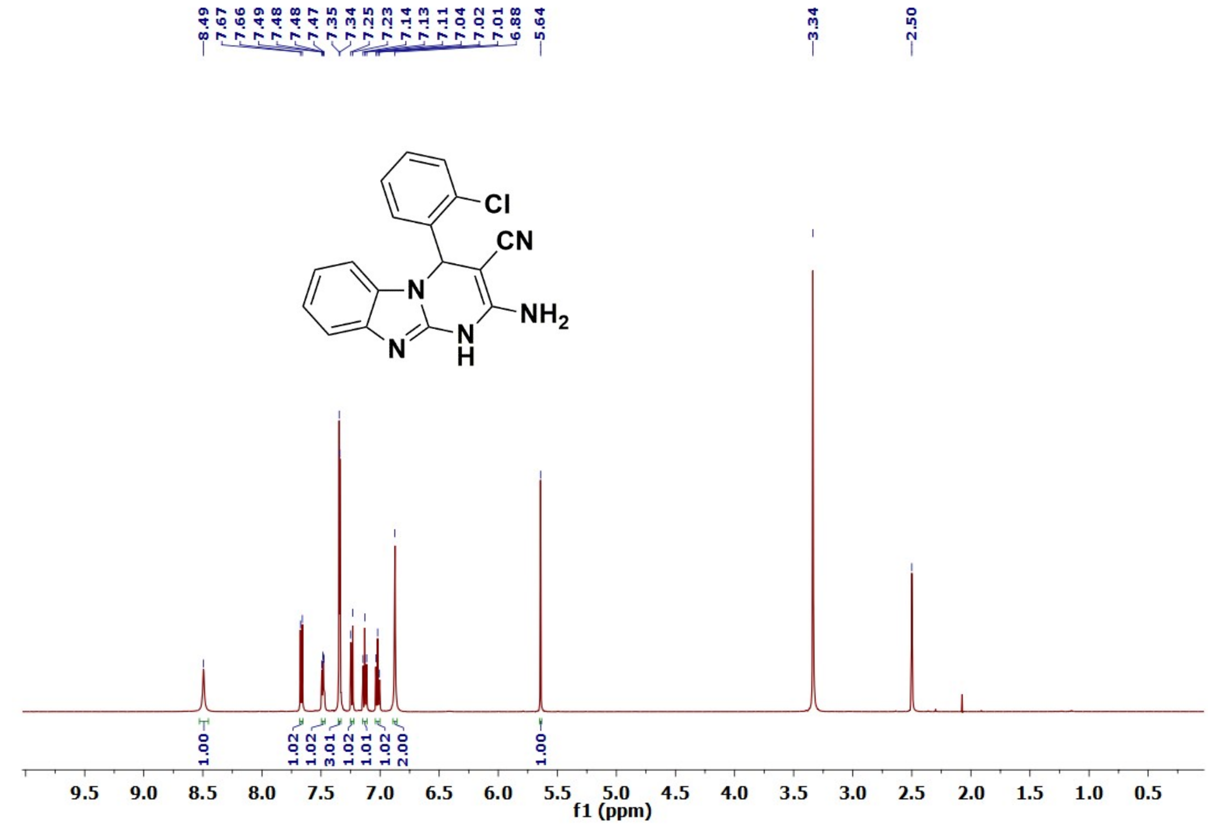

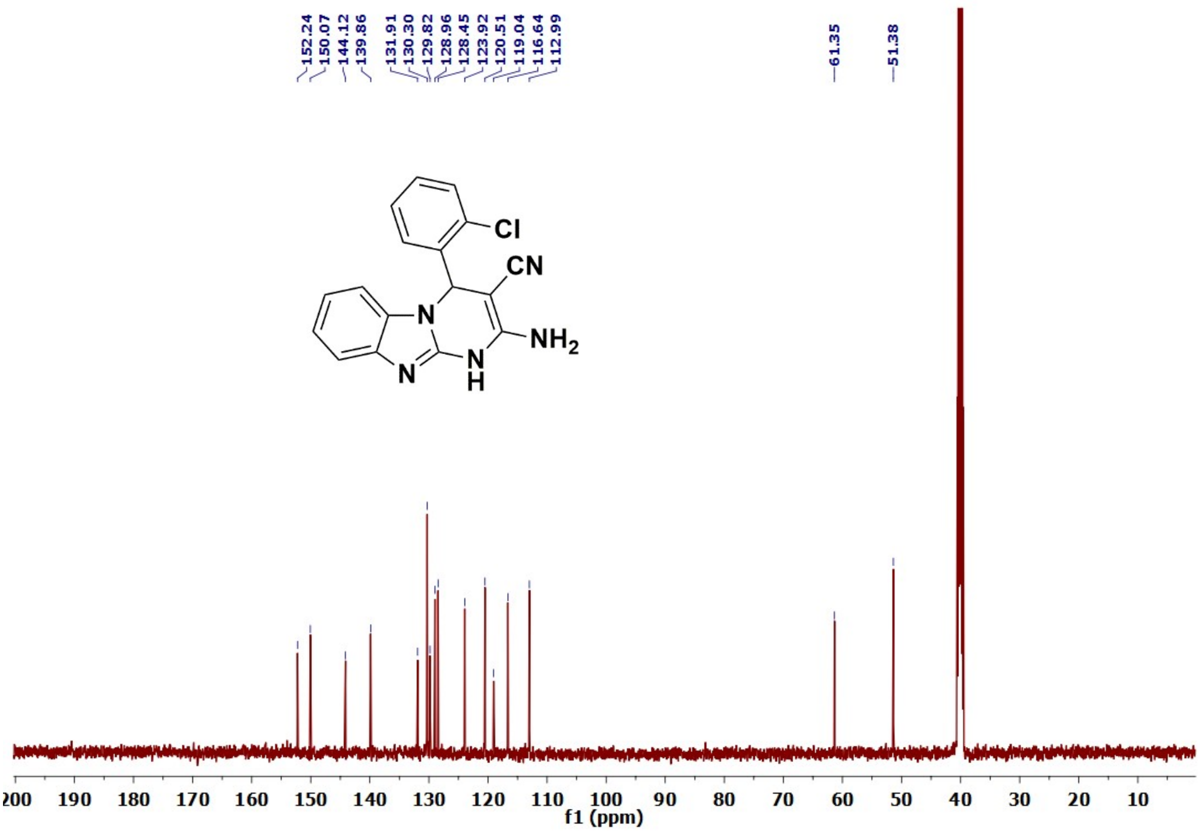

^{13}C {^{1}H} NMR of 3i in DMSO-d_6

^{1}H-NMR of 3j in DMSO-d_6

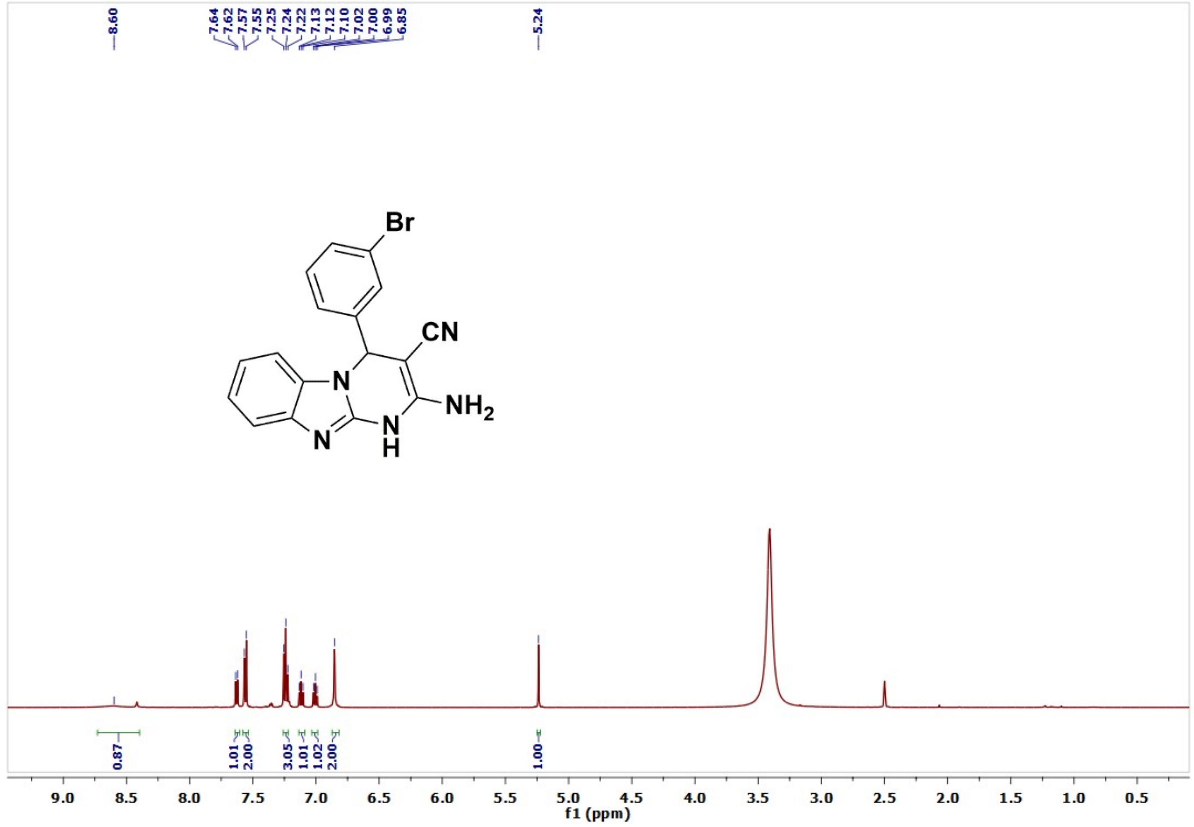

^{13}C {^{1}H} NMR of 3j in DMSO-d_6

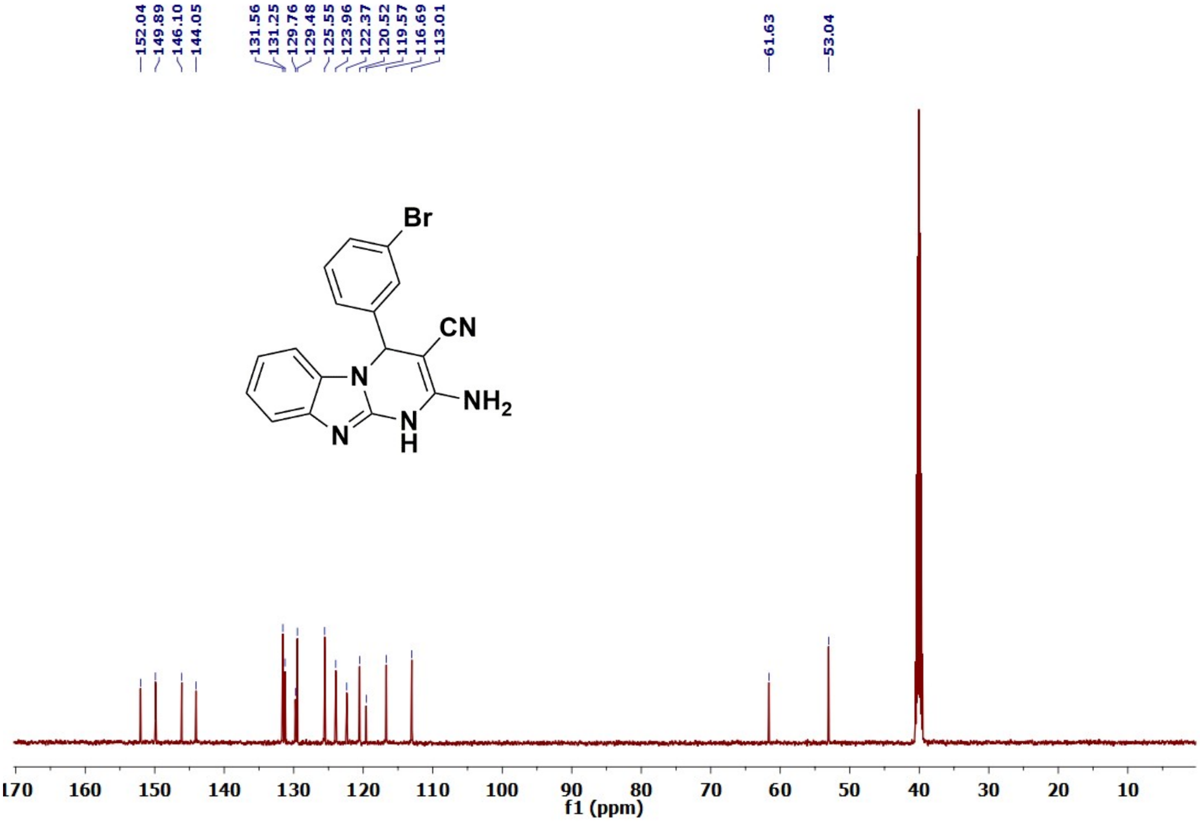

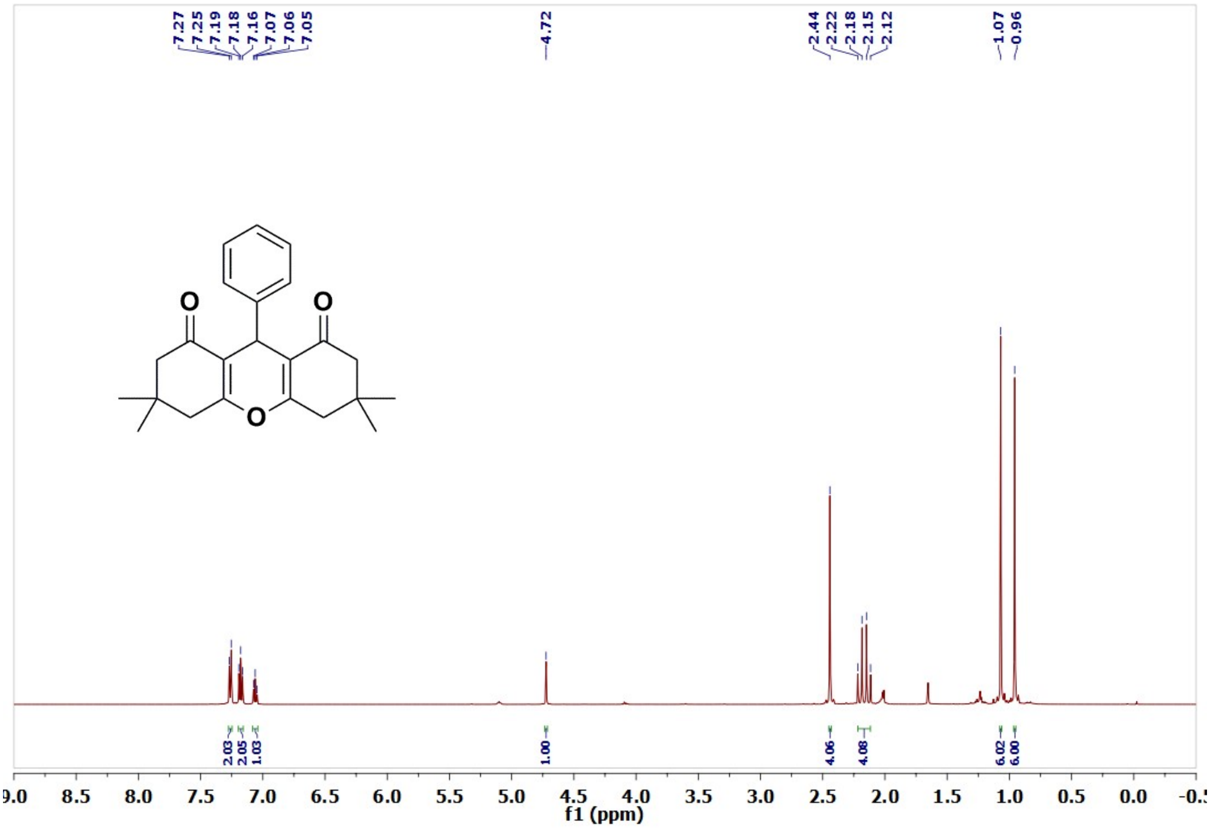

^{1}H-NMR of 4a in $CDCl_3$

^{13}C {^{1}H} NMR of 4a in $CDCl_3$

^{1}H-NMR of 4b in $CDCl_3$

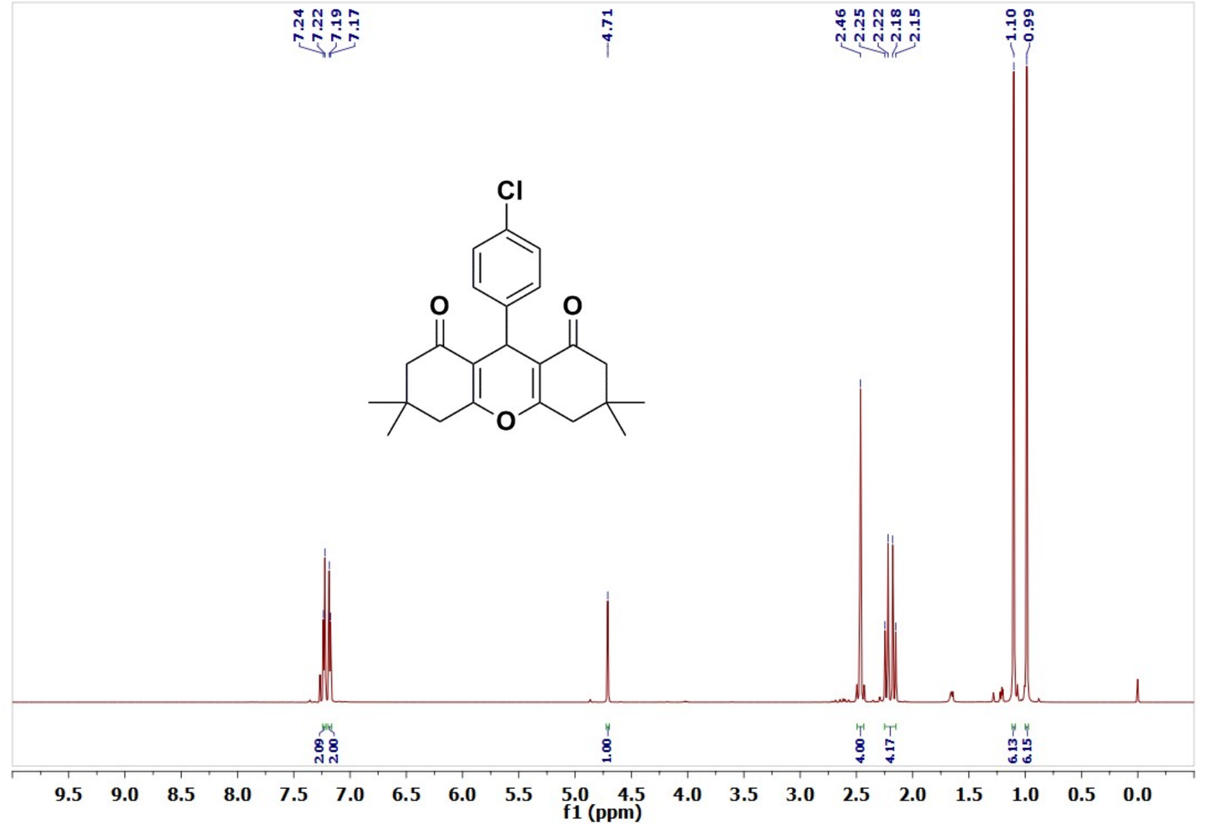

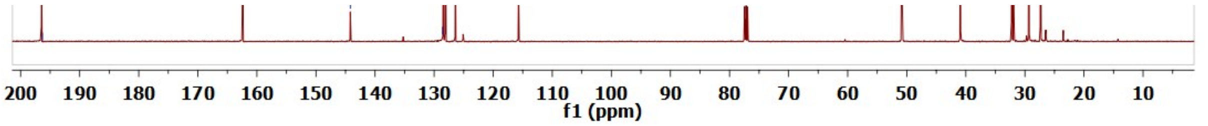

$^{13}C\ \{^{1}H\}$ NMR of 4b in $CDCl_3$

^{1}H-NMR of 4c in $CDCl_3$

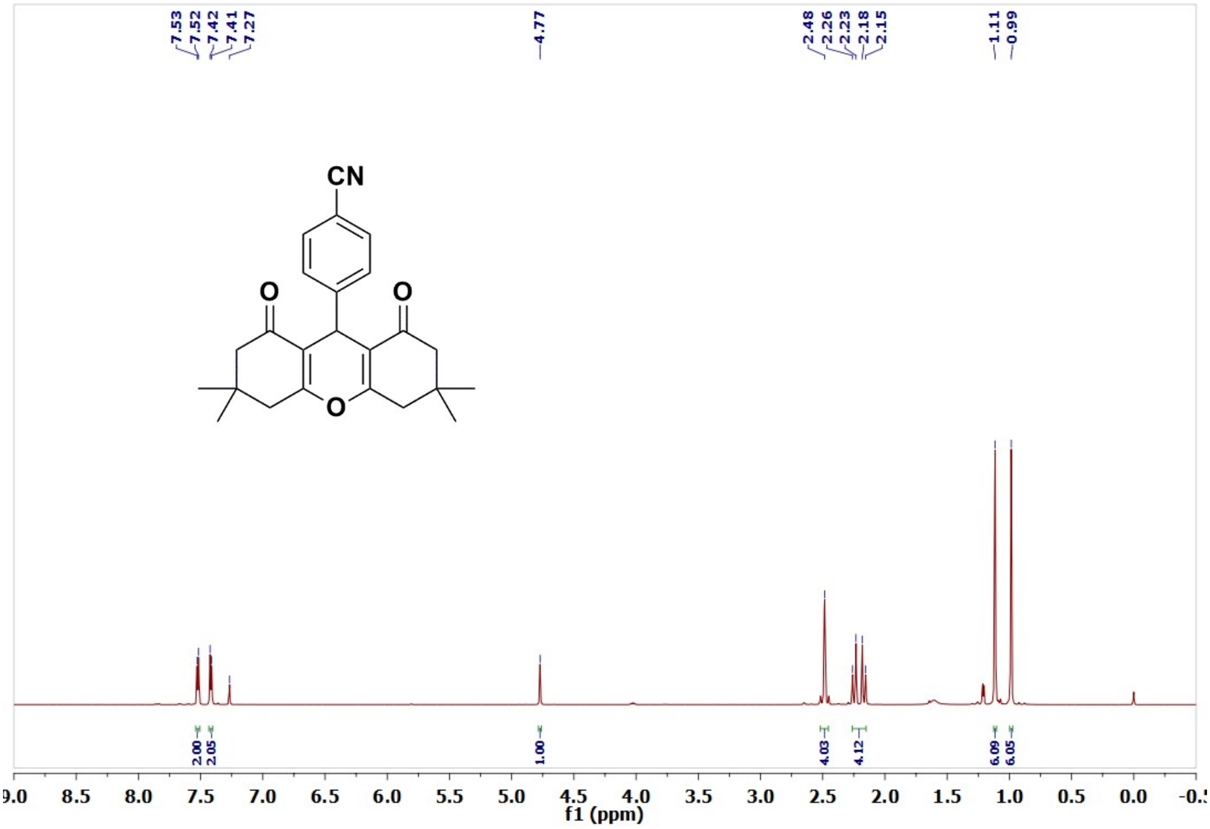

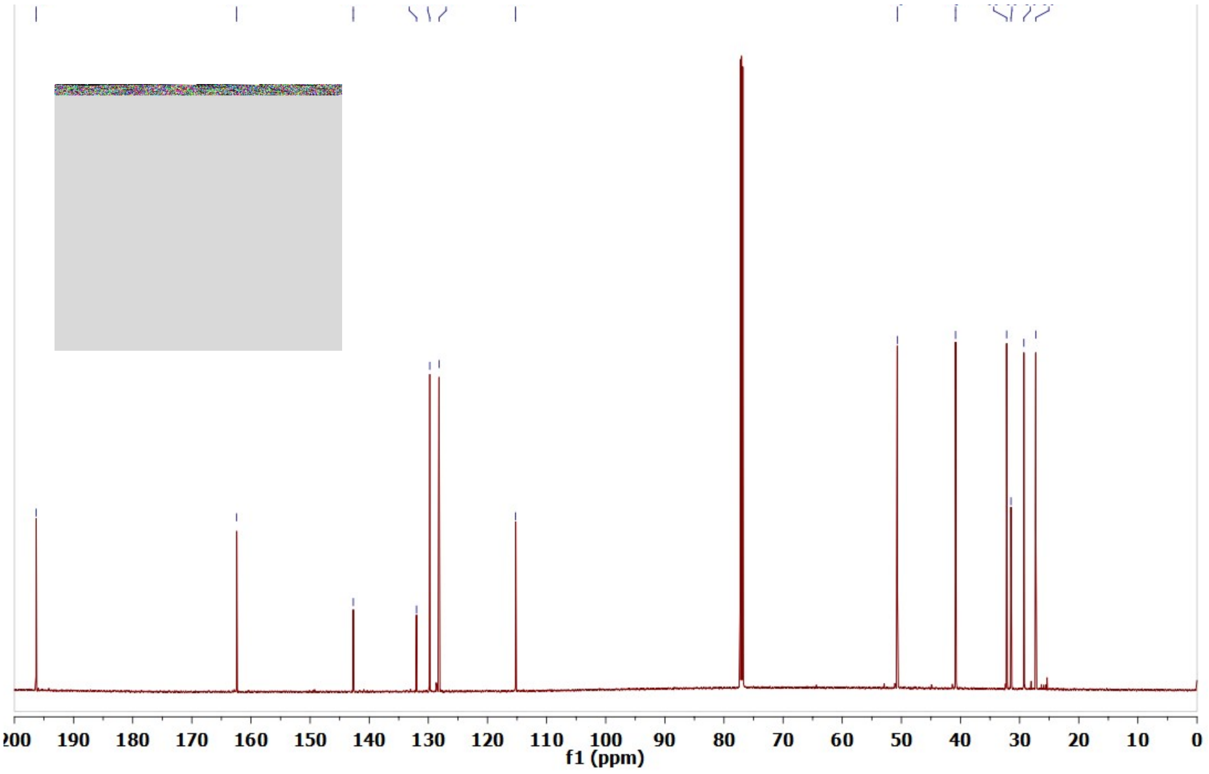

^{13}C {^{1}H} NMR of 4c in $CDCl_3$

^{1}H-NMR of 4d in $CDCl_3$

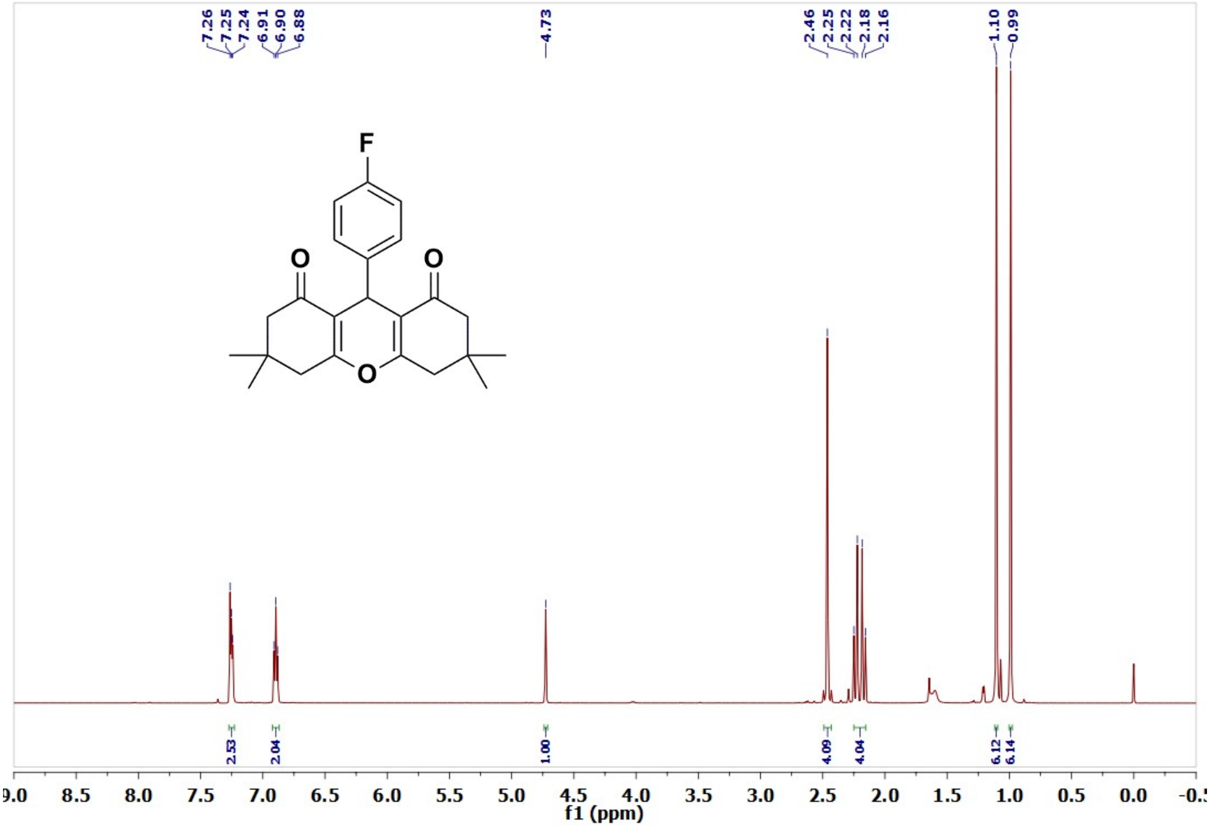

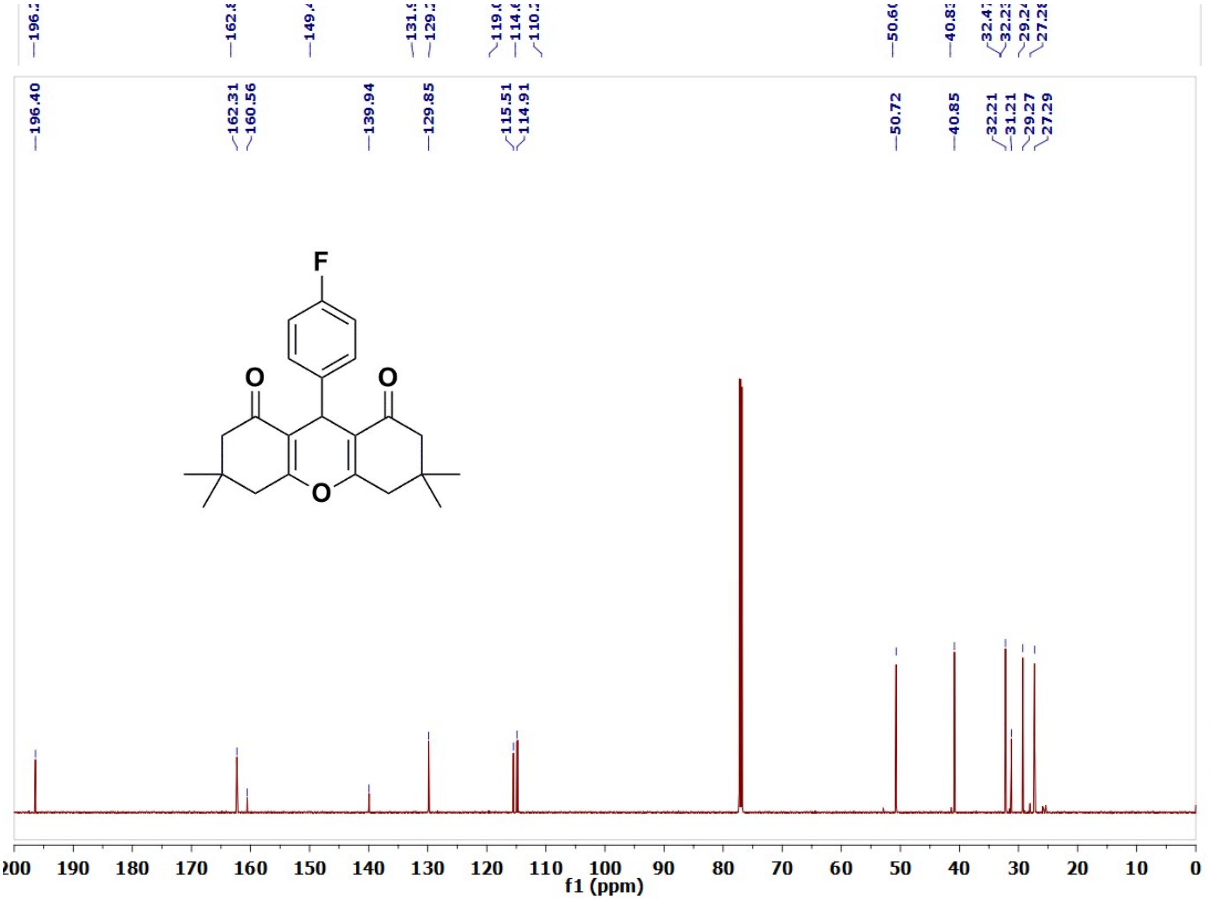

^{1}H-NMR of 4e in CDCl$_3$

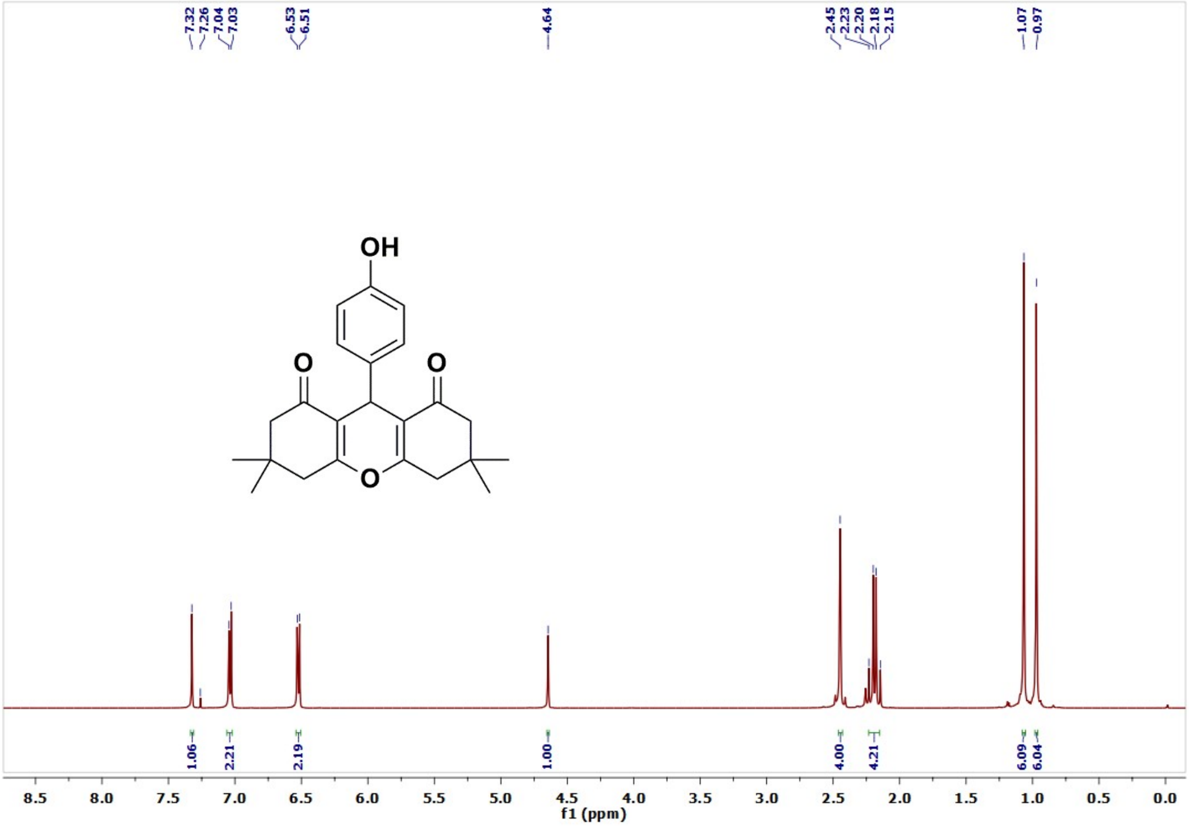

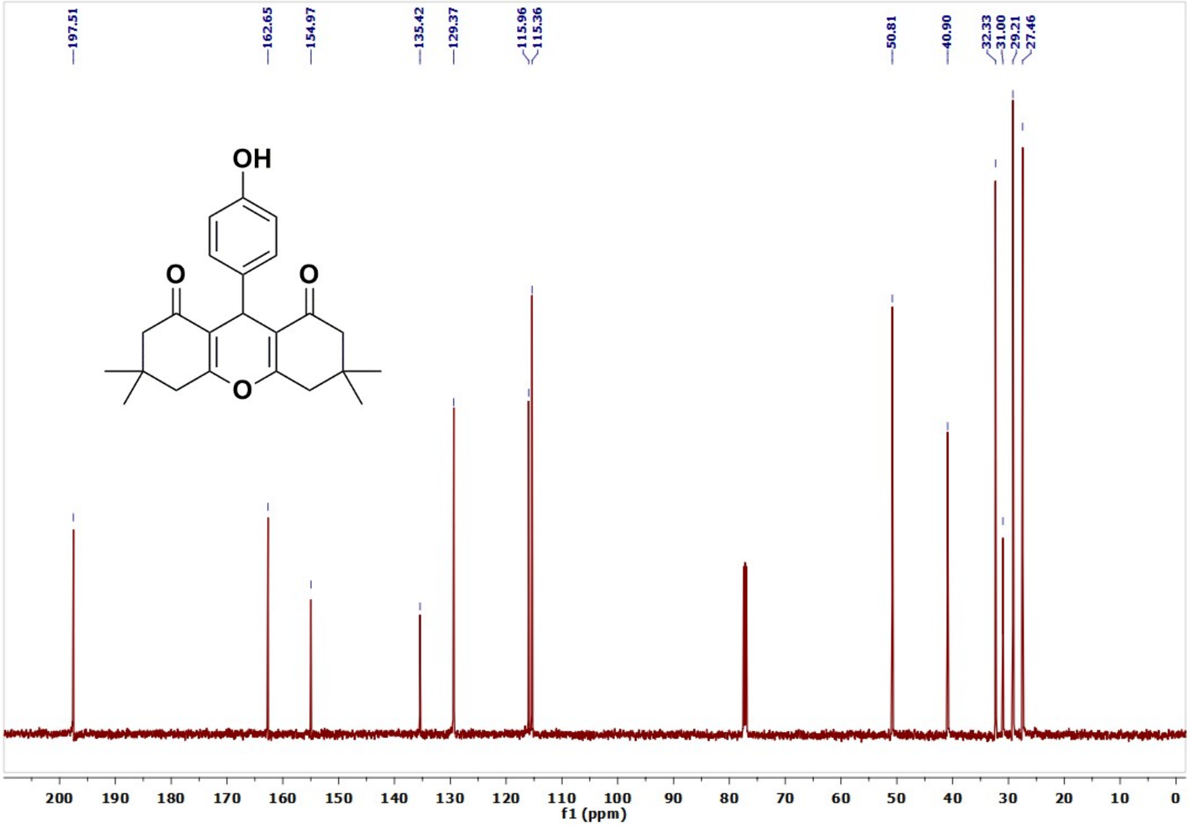

^{13}C {^{1}H} NMR of 4e in CDCl$_3$

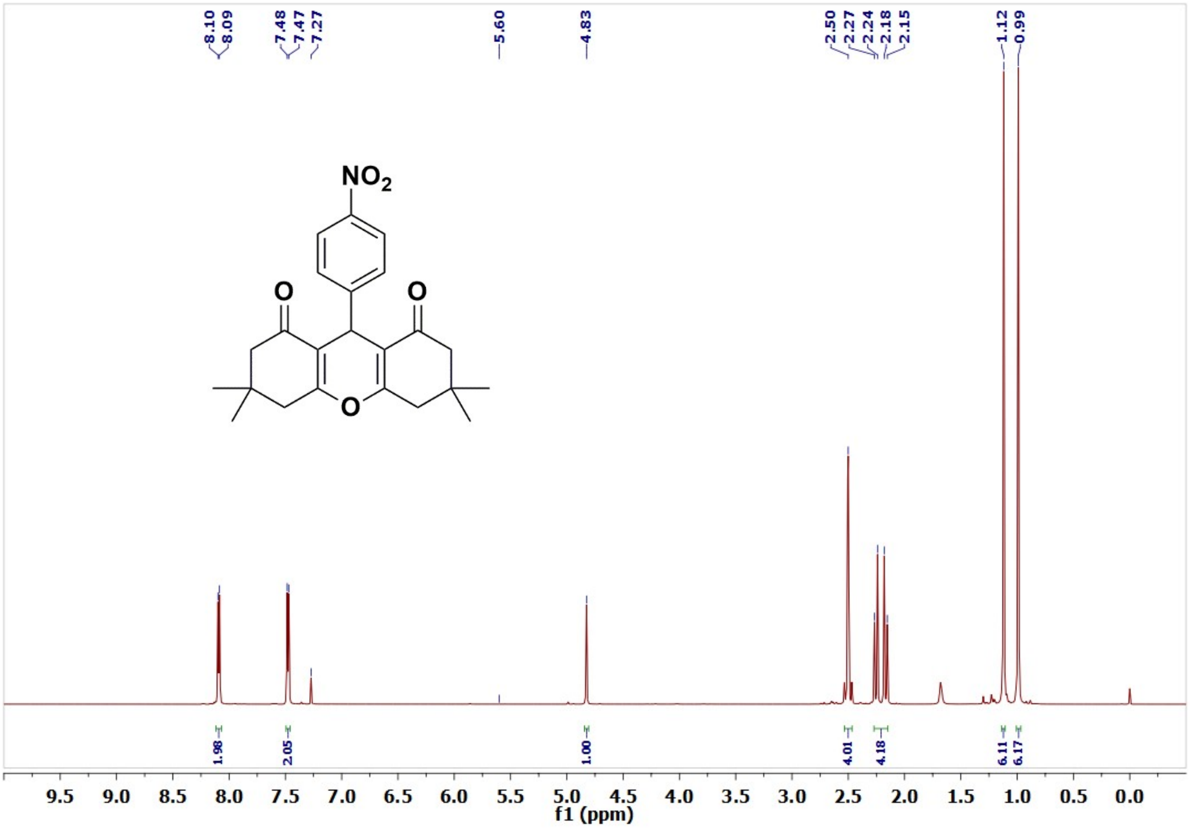

^{1}H-NMR of 4f in $CDCl_3$

^{13}C {^{1}H} NMR of 4f in $CDCl_3$

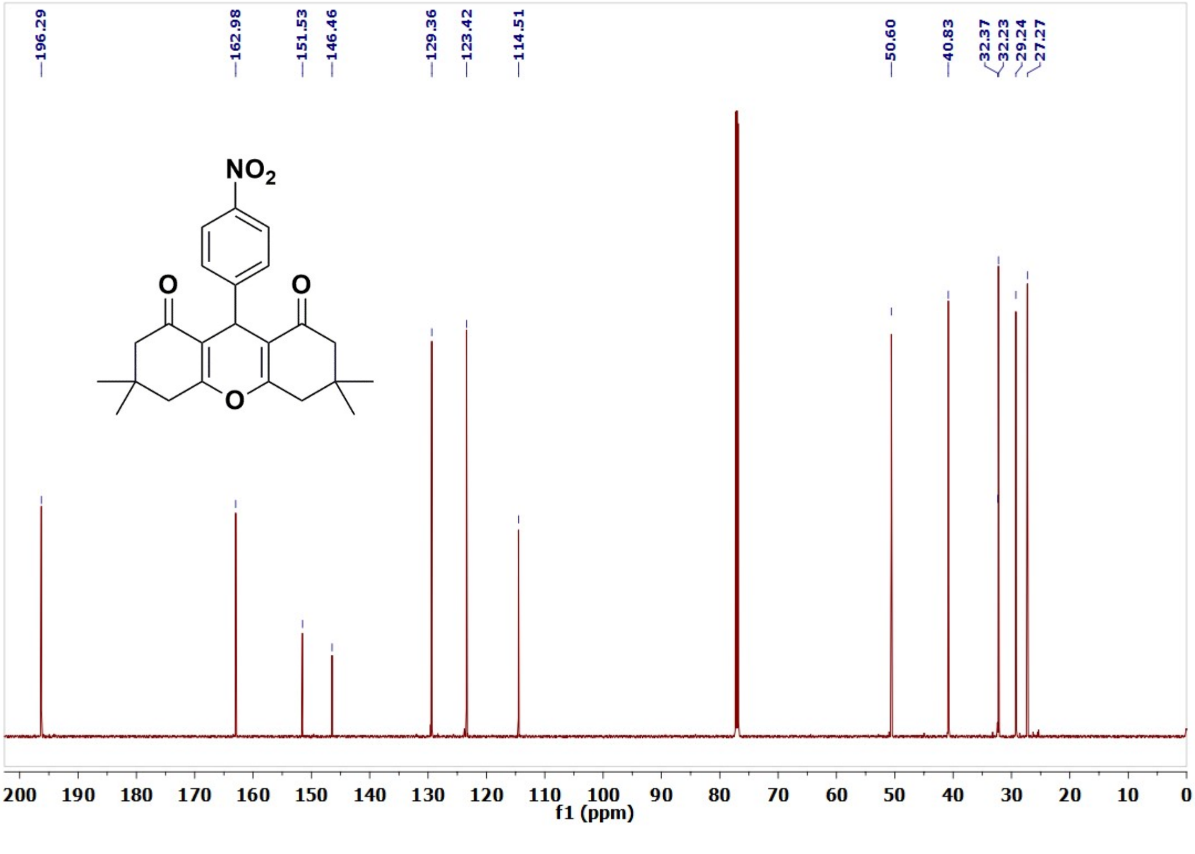

^{1}H-NMR of 4g in $CDCl_3$

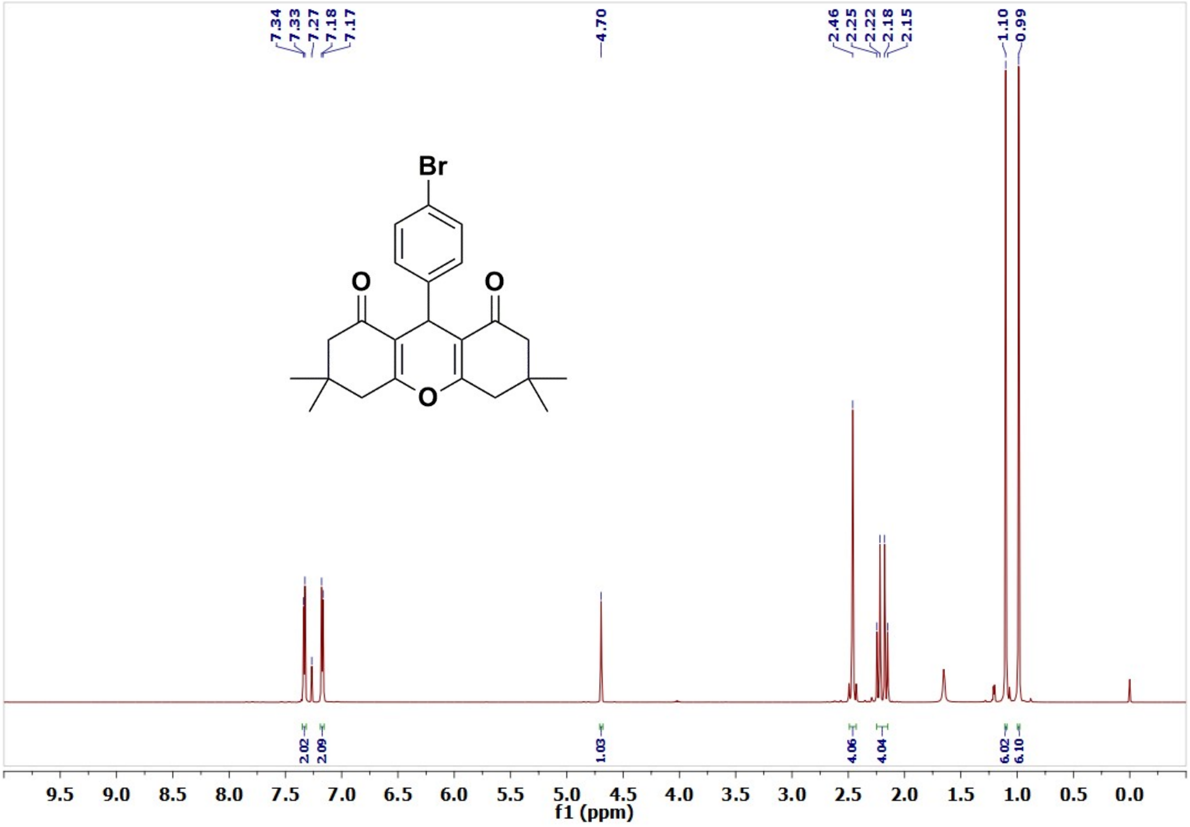

$^{13}C\ \{^1H\}$ NMR of 4g in $CDCl_3$

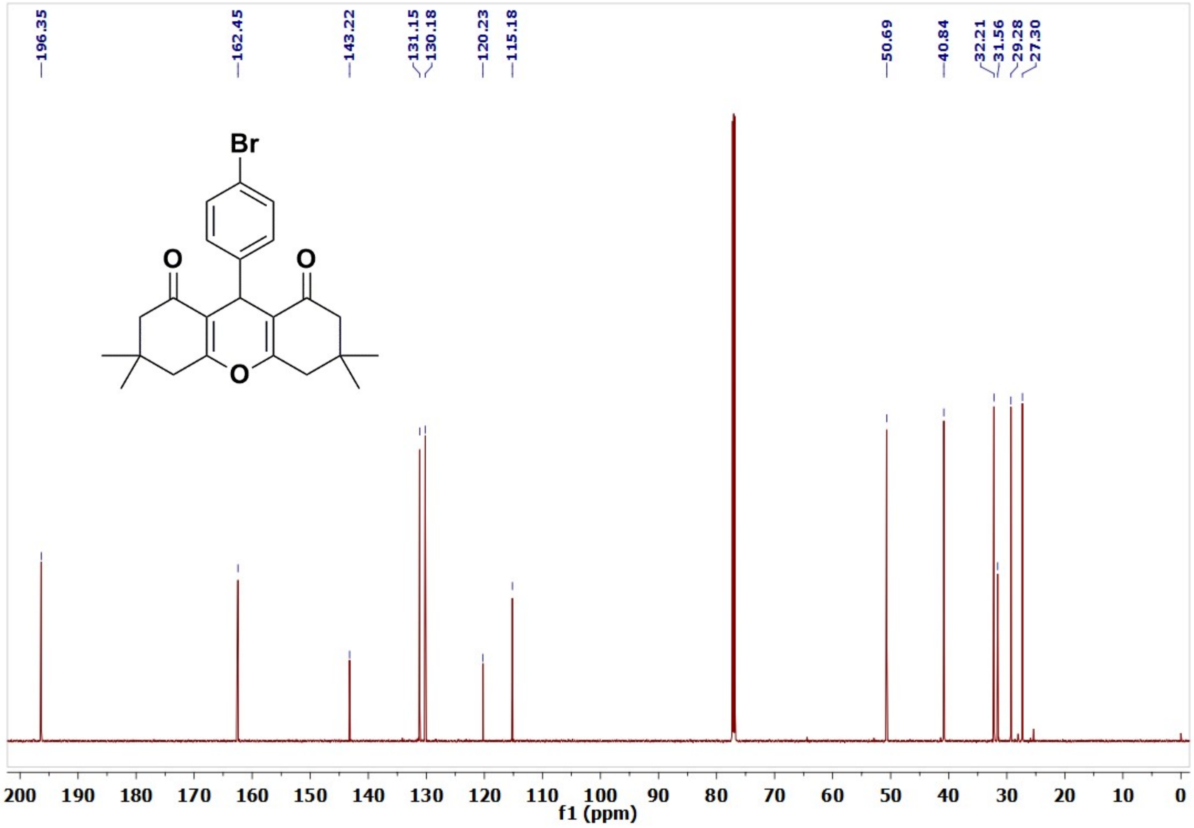

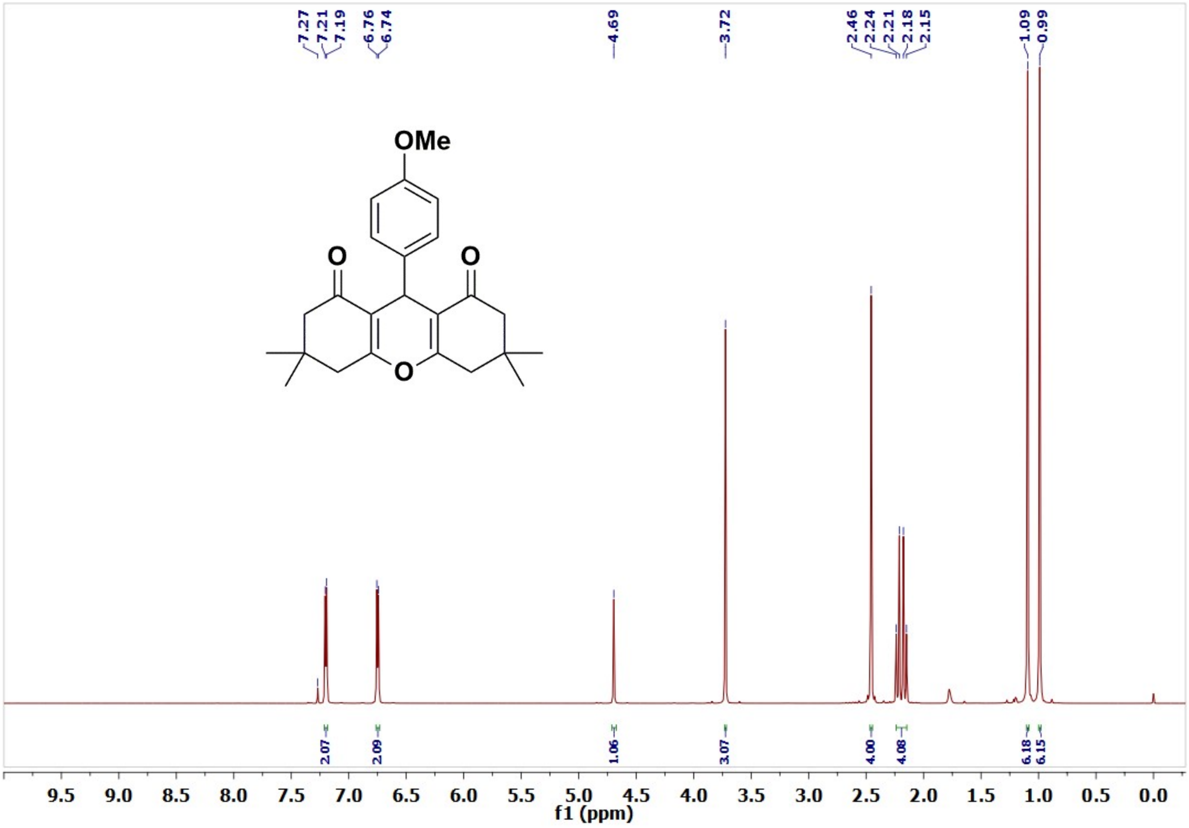

^{1}H-NMR of 4h in $CDCl_3$

^{13}C {^{1}H} NMR of 4h in $CDCl_3$

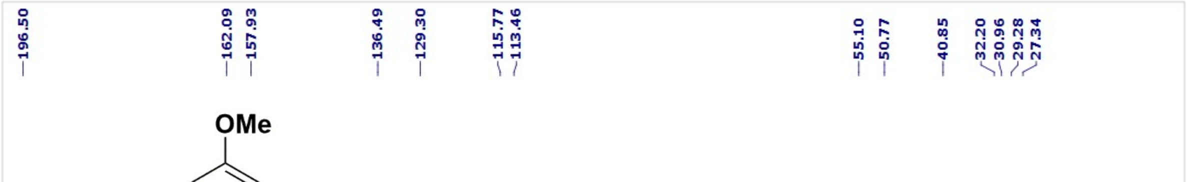

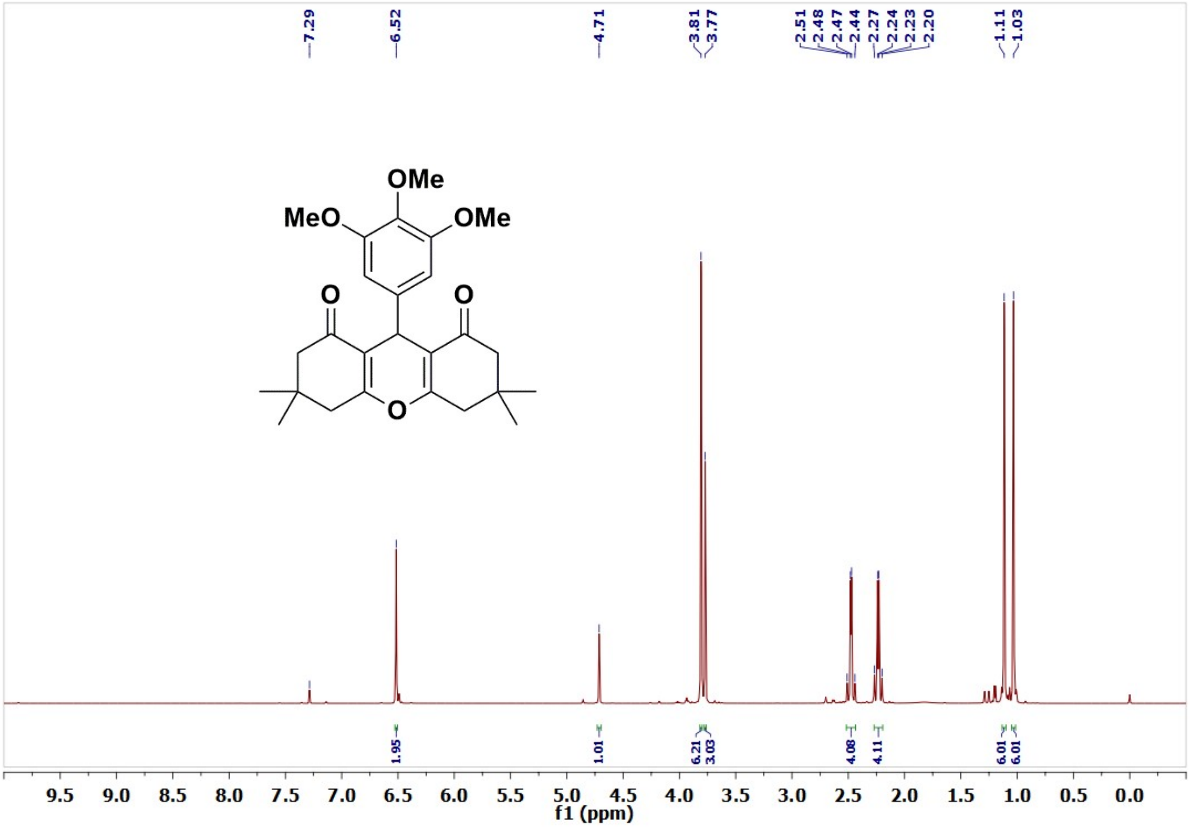

^{1}H-NMR of 4i in $CDCl_3$

^{13}C {^{1}H} NMR of 4i in $CDCl_3$

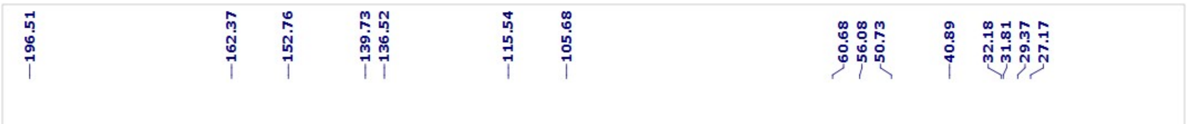

^{1}H-NMR of 4j in $CDCl_3$

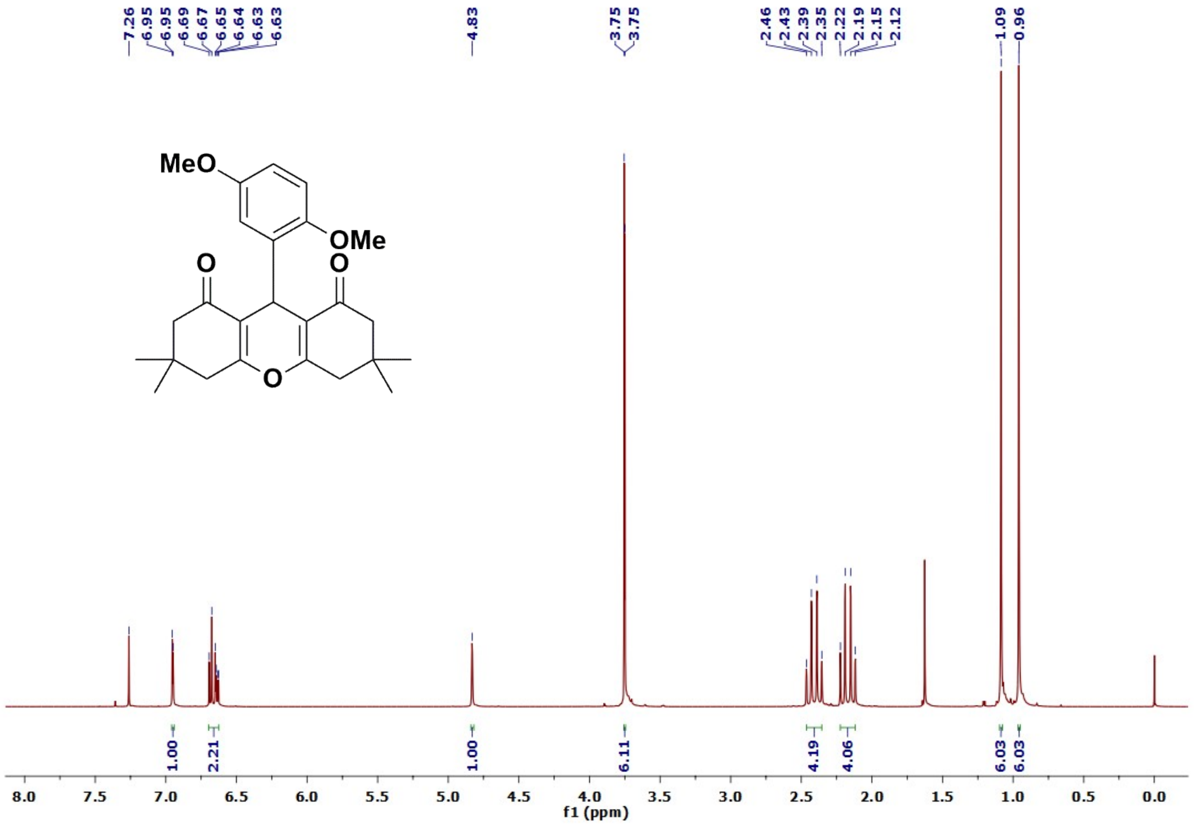

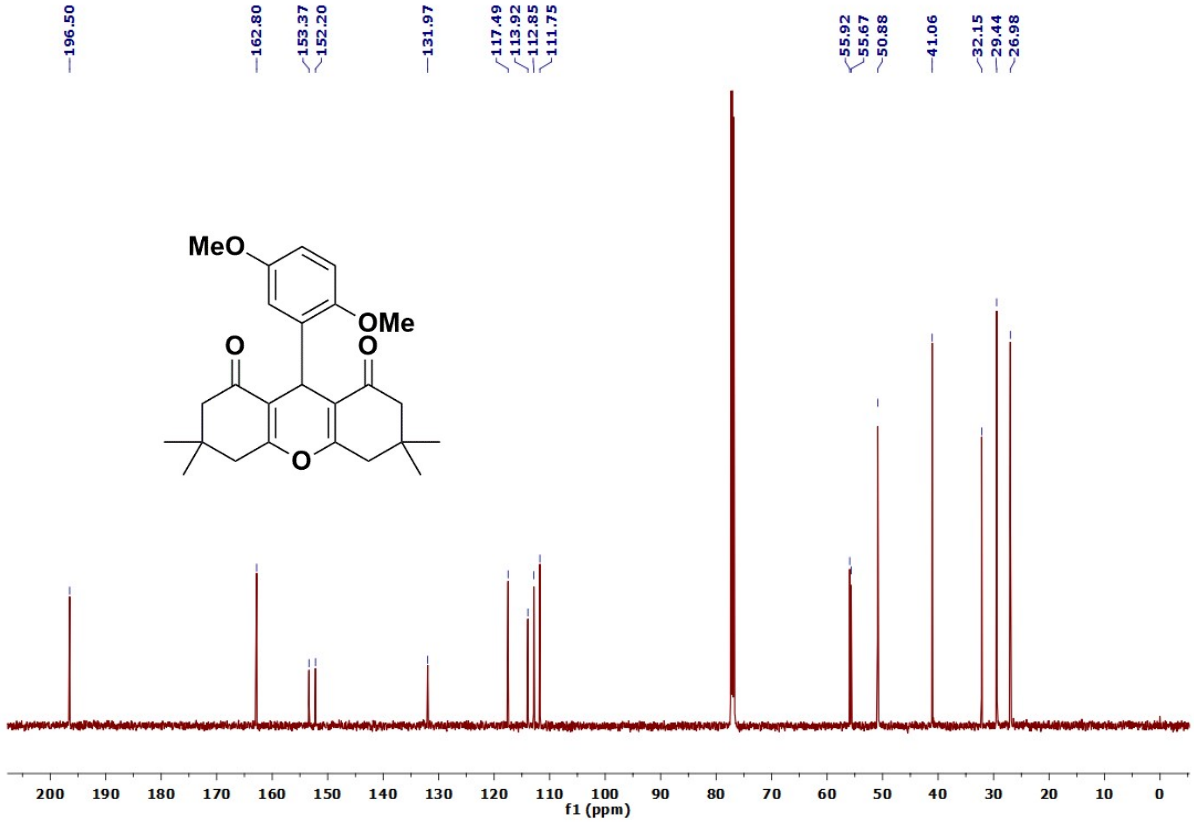

^{13}C {^{1}H} NMR of 4j in $CDCl_3$

HRMS data of compound 2d

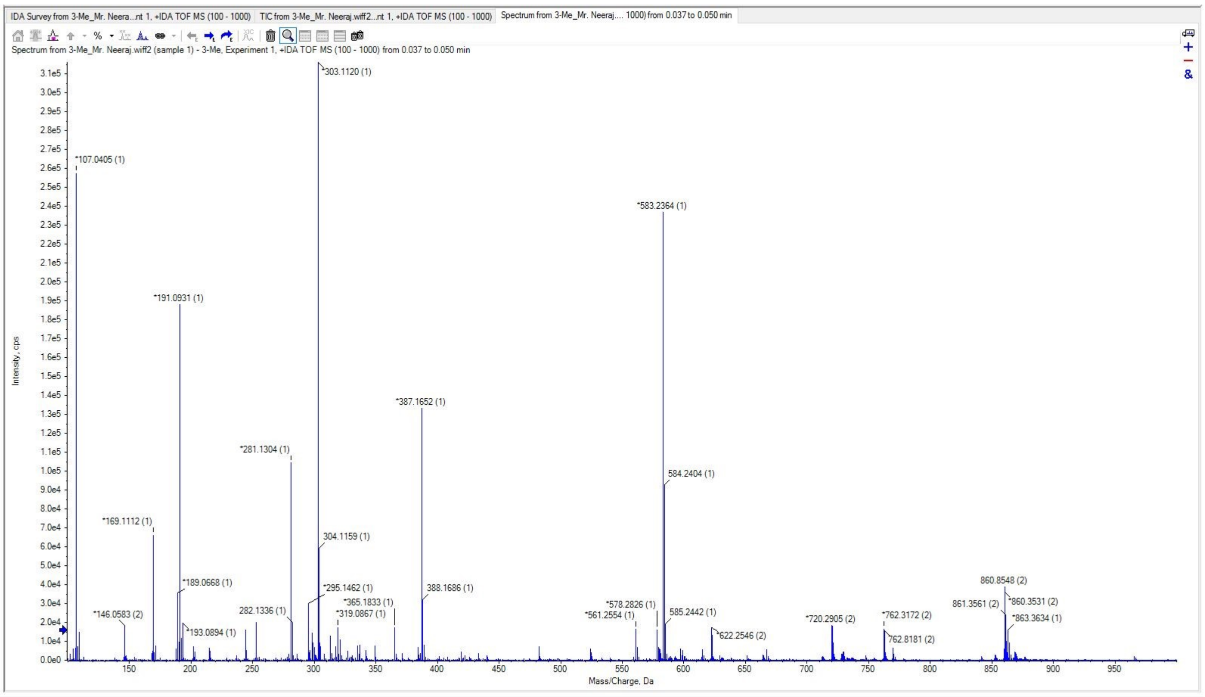

HRMS data of compound 2e

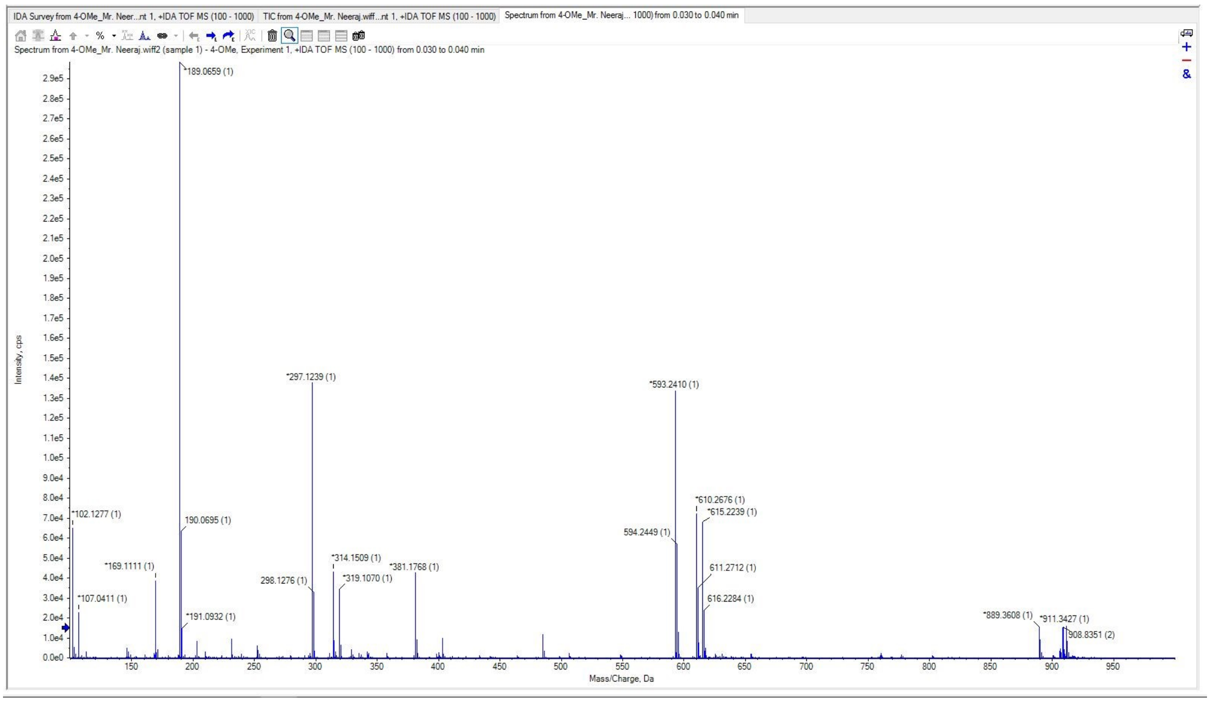

HRMS data of compound 2g

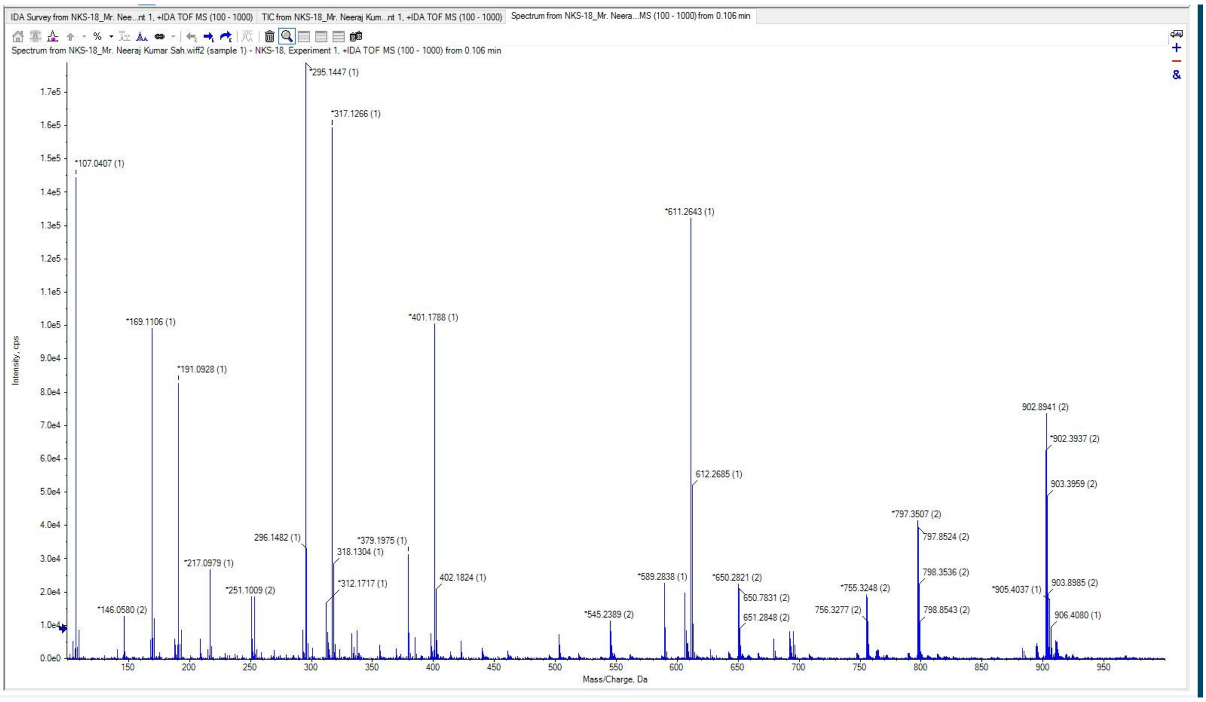

HRMS data of compound 3b

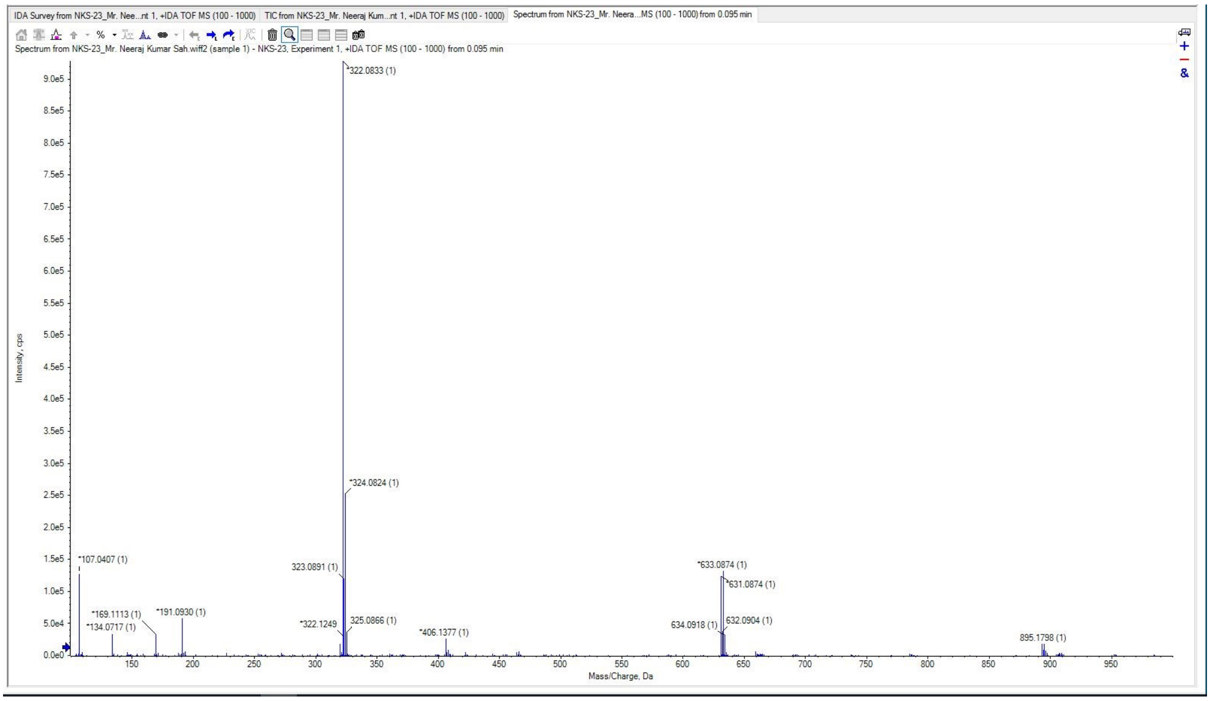

HRMS data of compound 3d

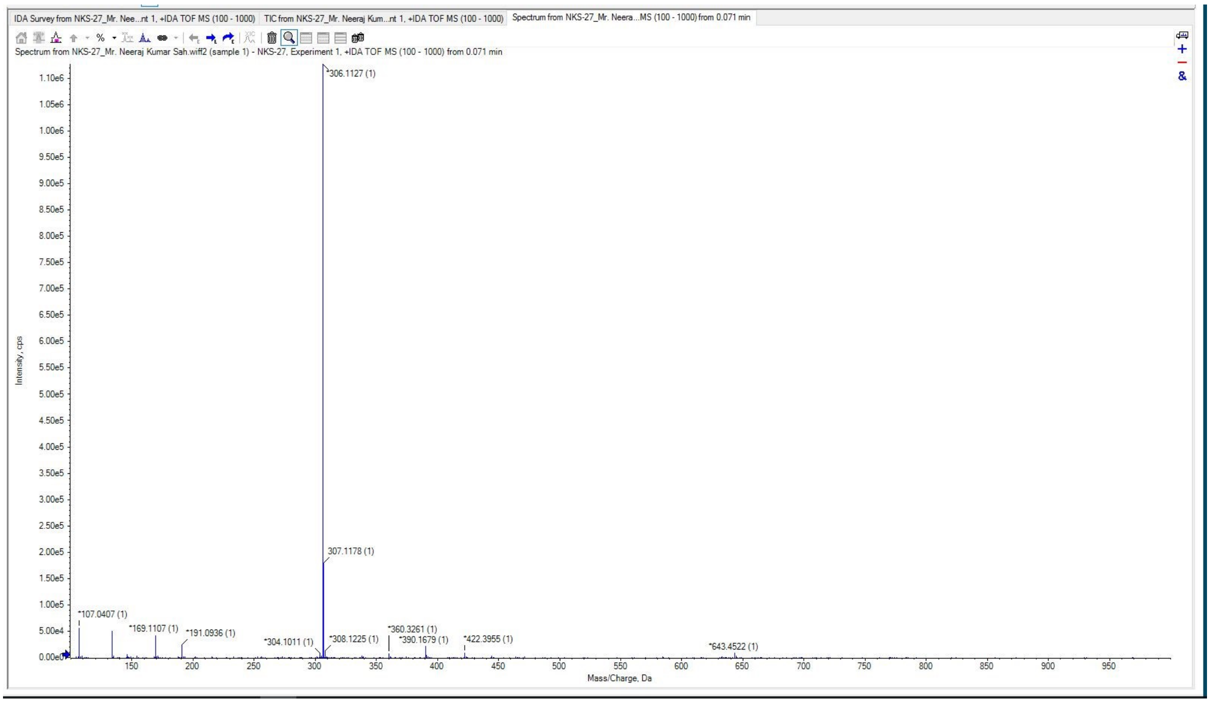

HRMS data of compound 3e

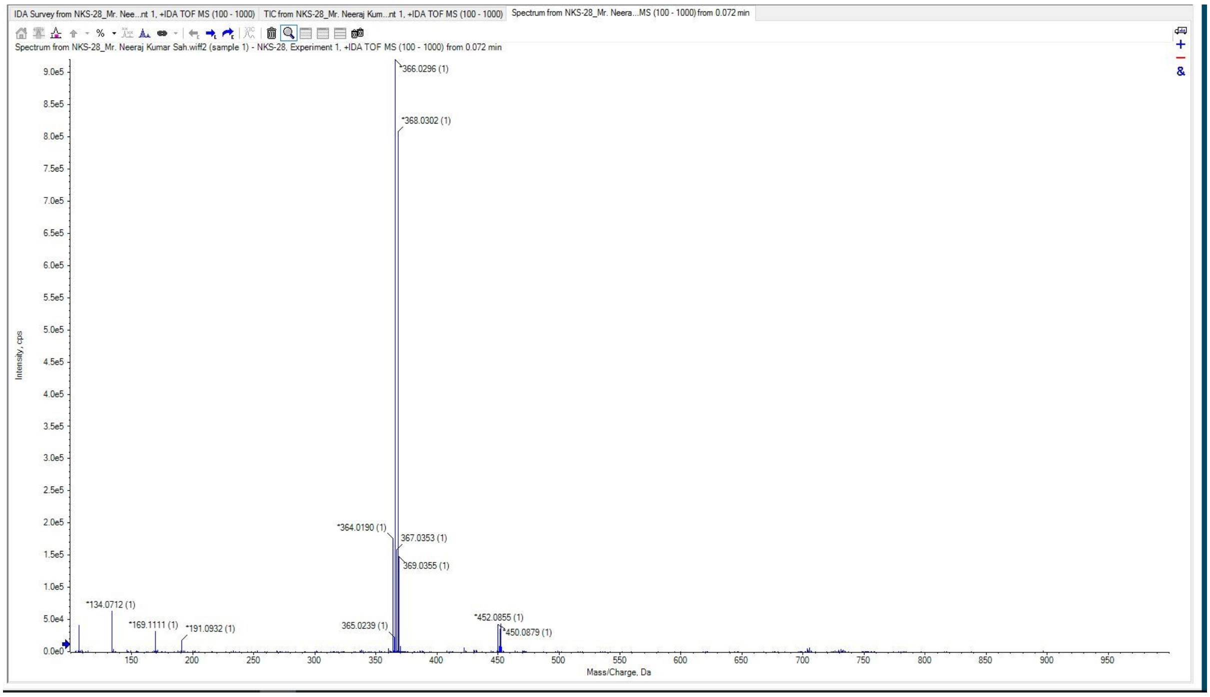

HRMS data of compound 3f

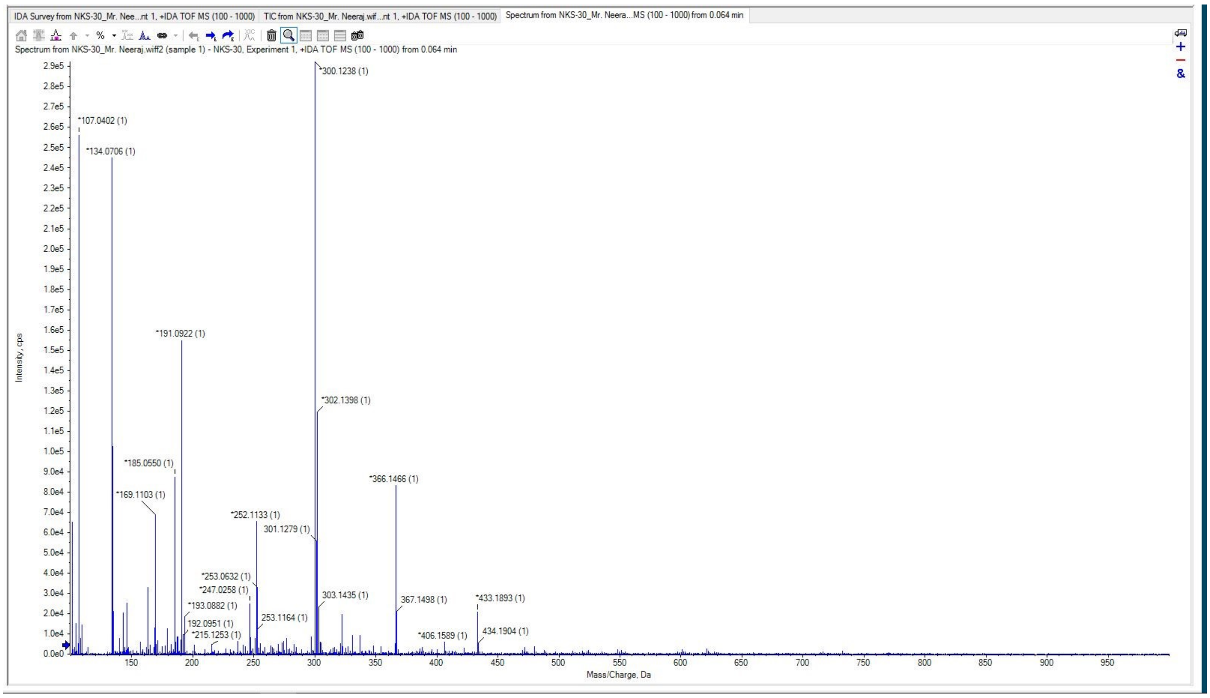

HRMS data of compound 3g

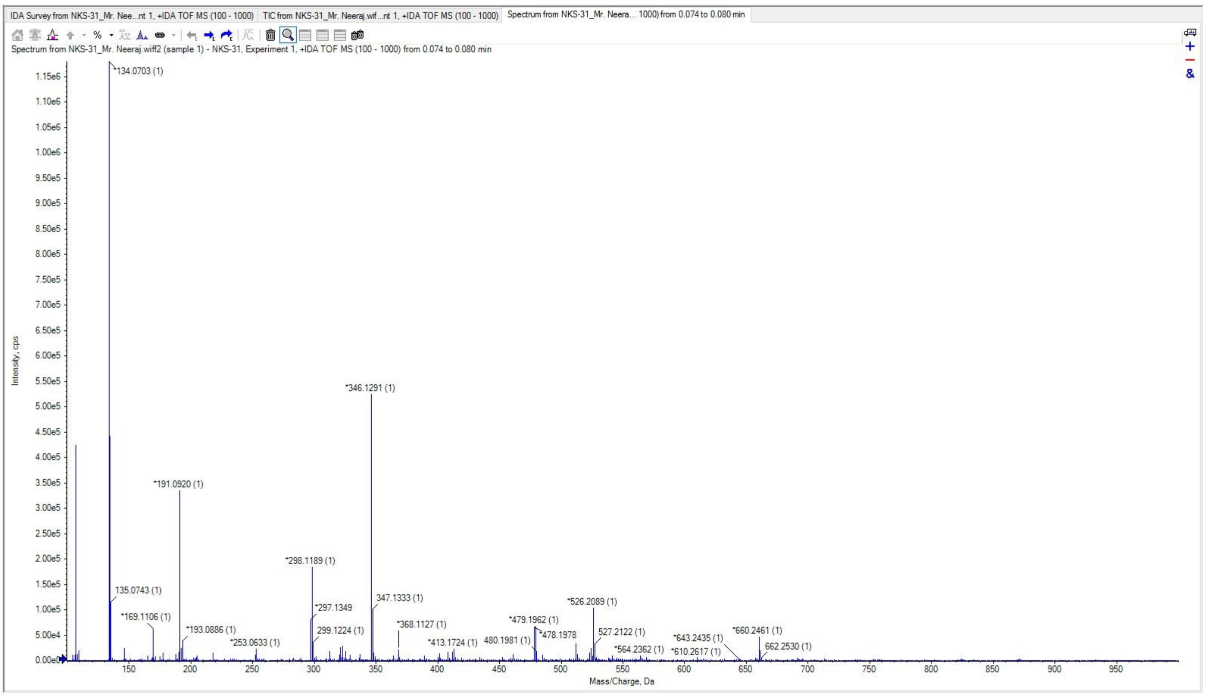

HRMS data of compound 3h

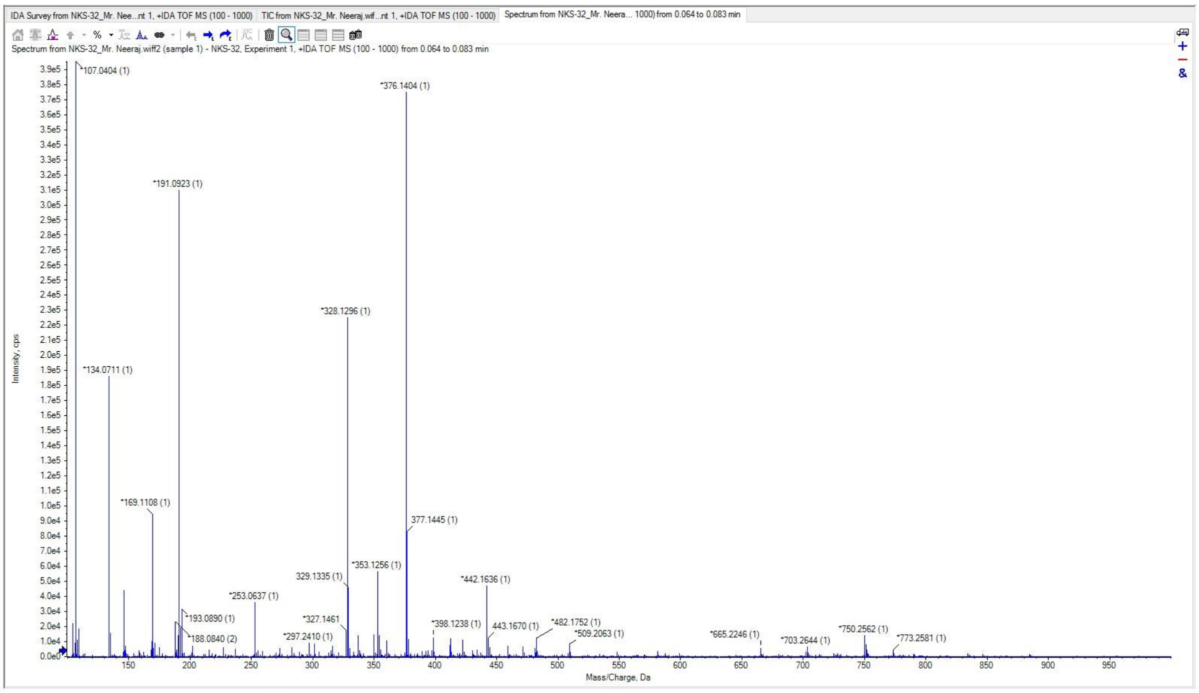

HRMS data of compound 4a

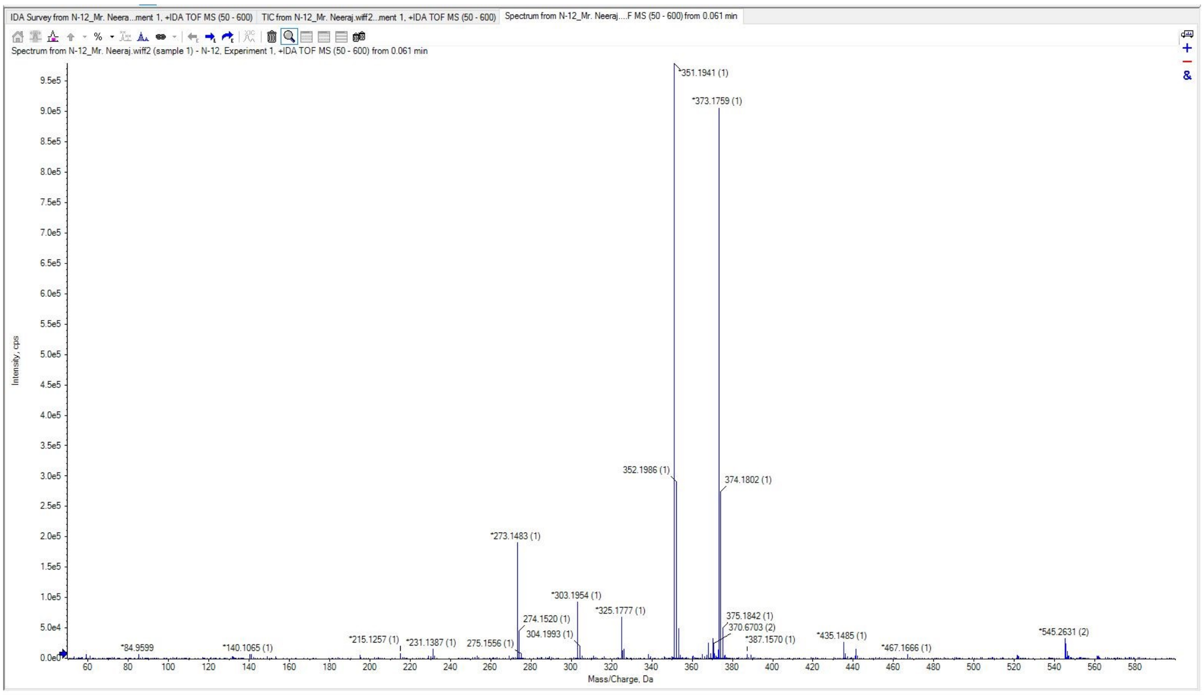

HRMS data of compound 4b

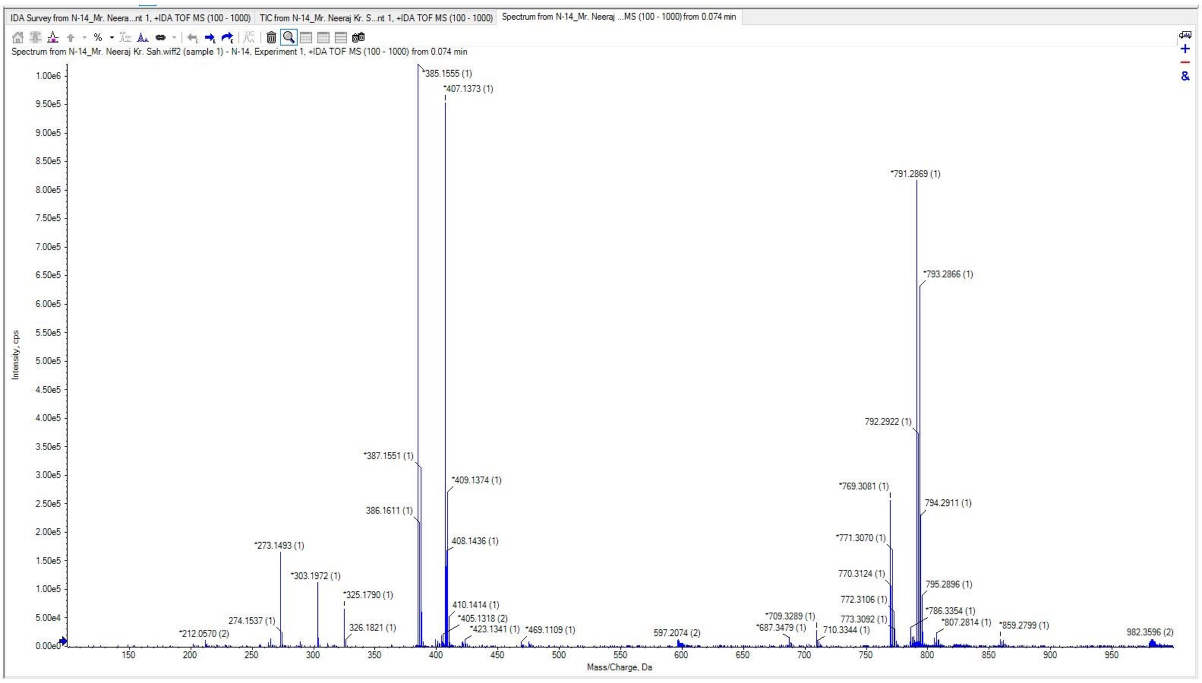

HRMS data of compound 4c

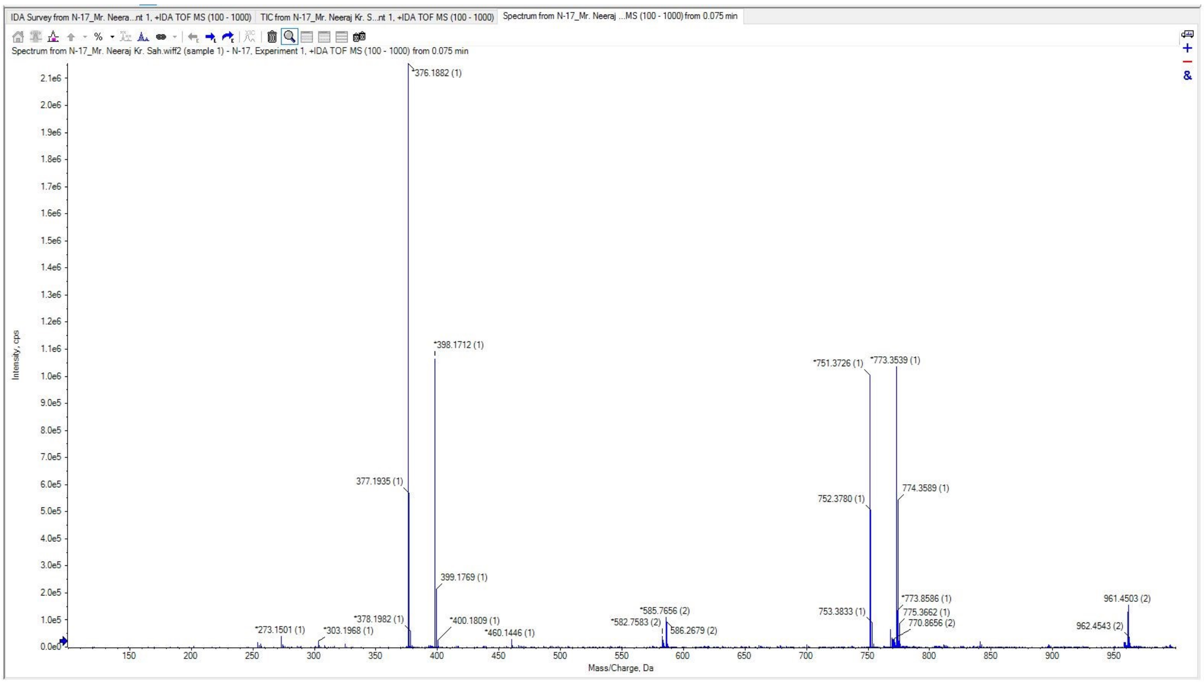

HRMS data of compound 4d

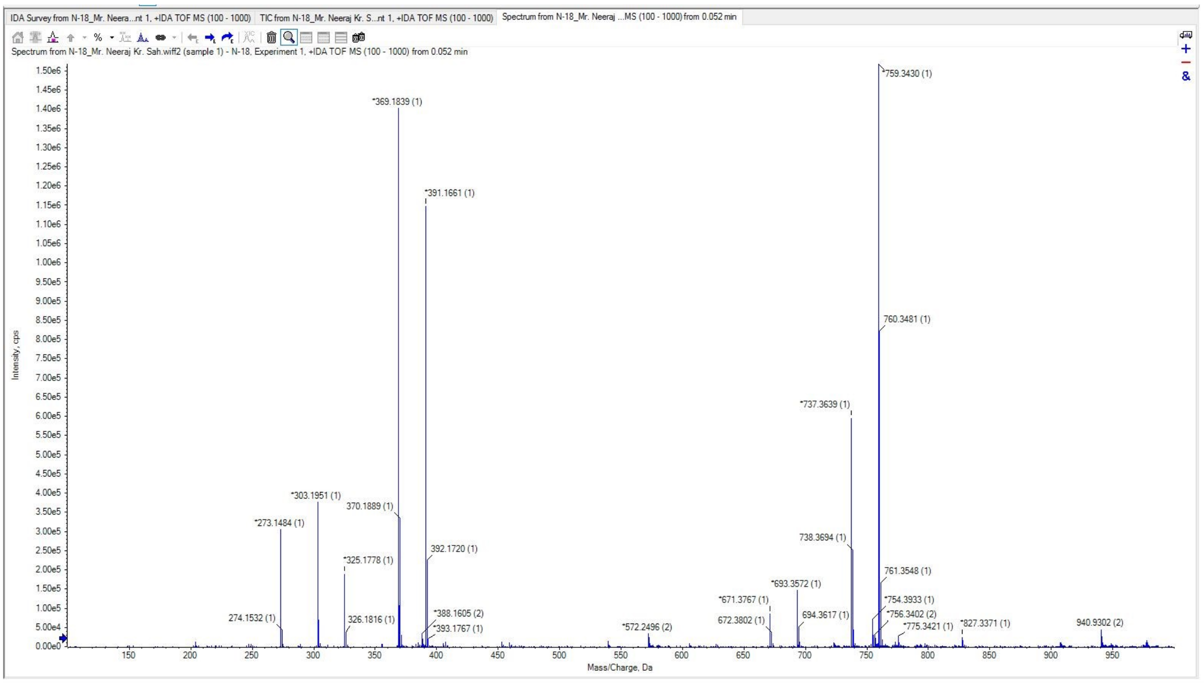

HRMS data of compound 4f

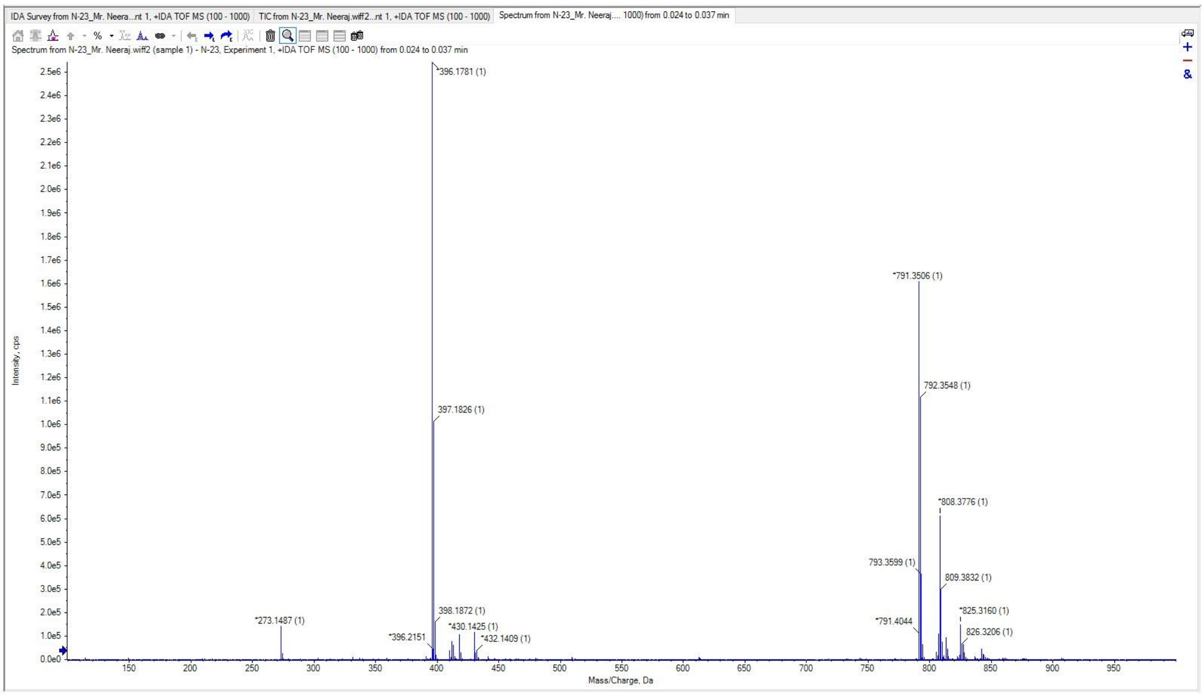

HRMS data of compound 4g

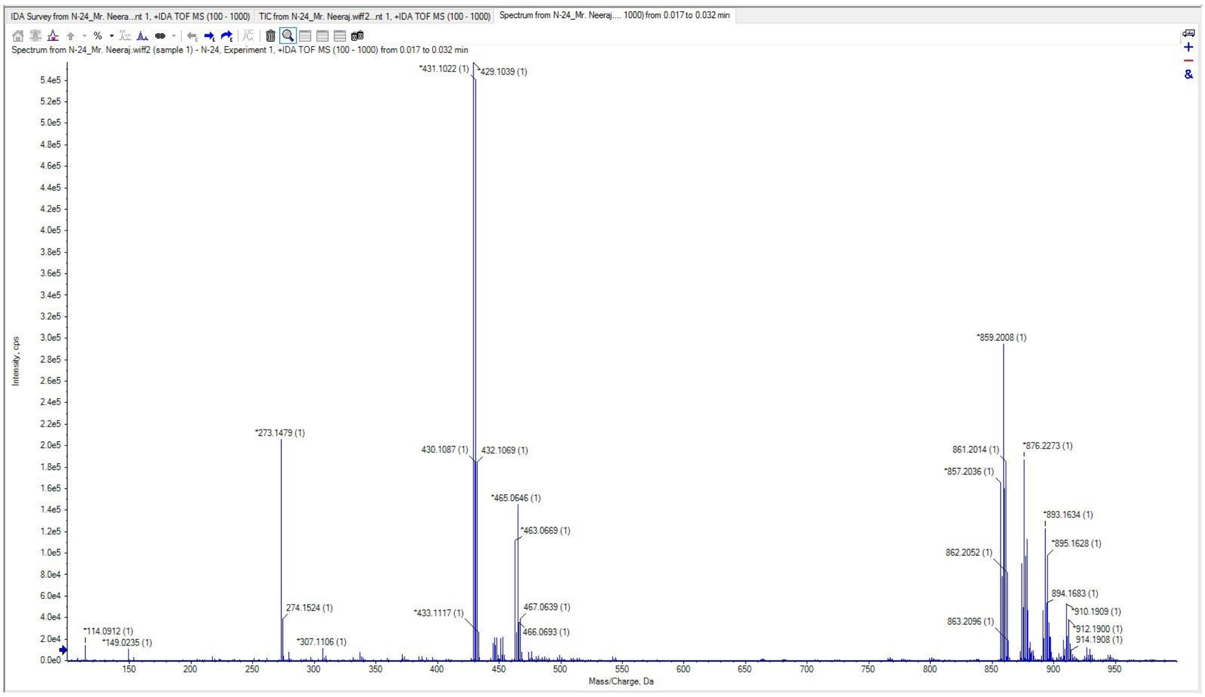

HRMS data of compound 4h

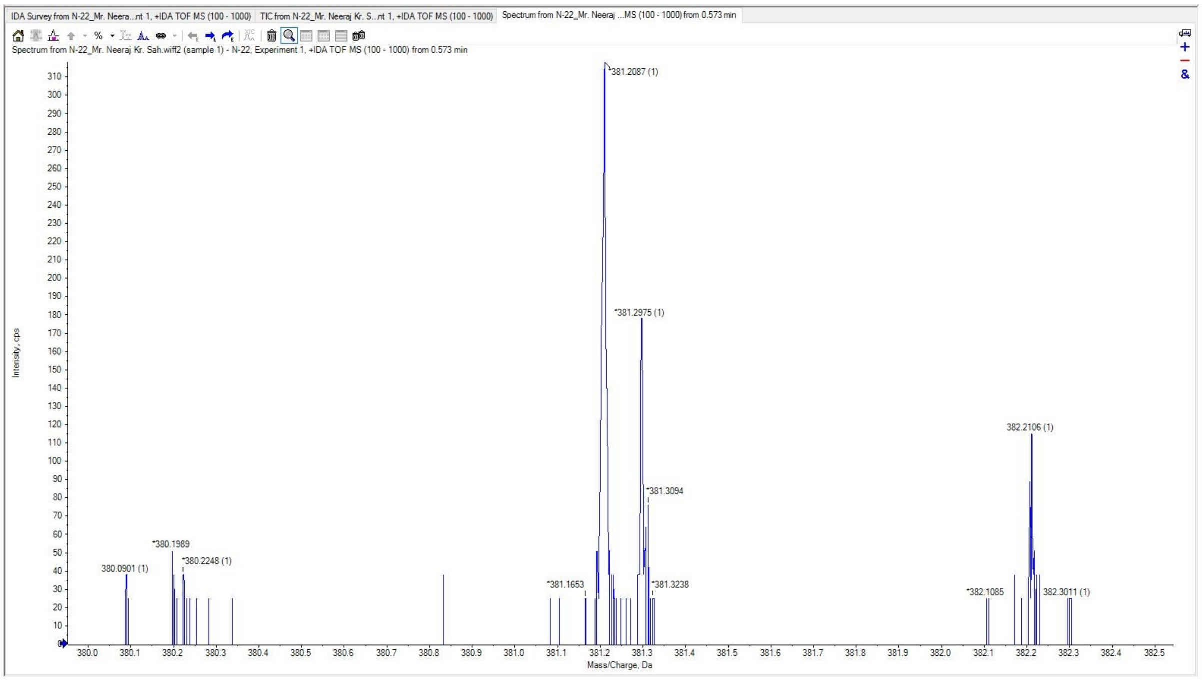

HRMS data of compound 4i

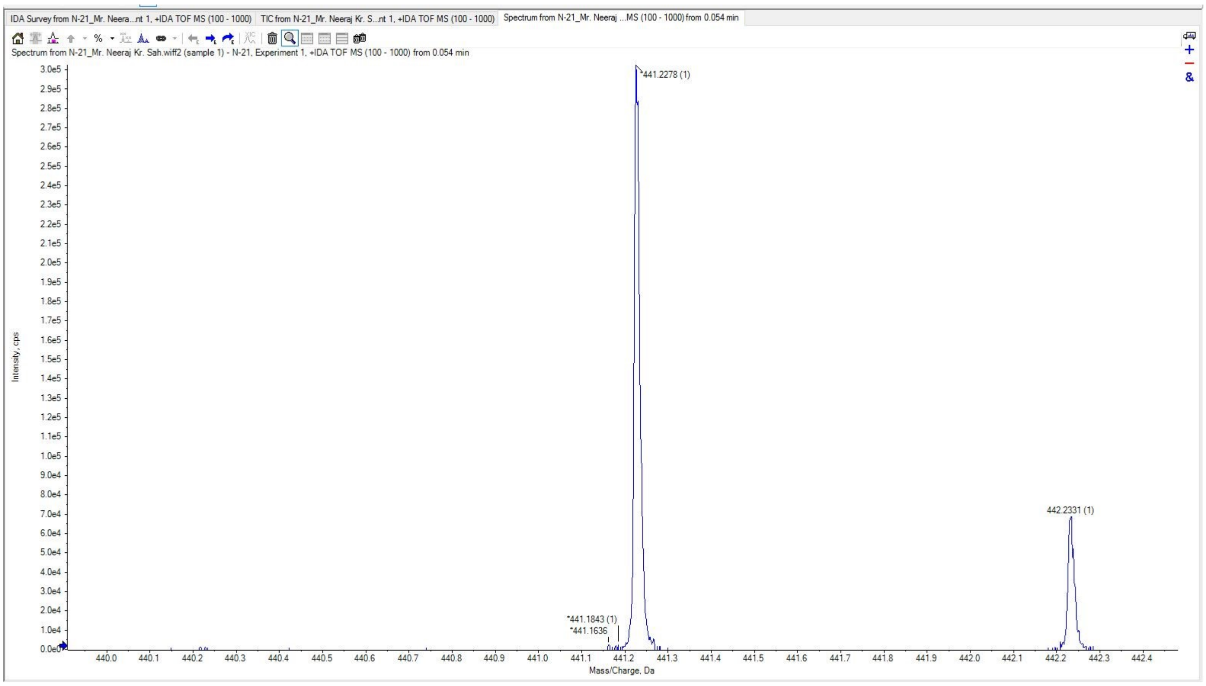

HRMS data of compound 4j

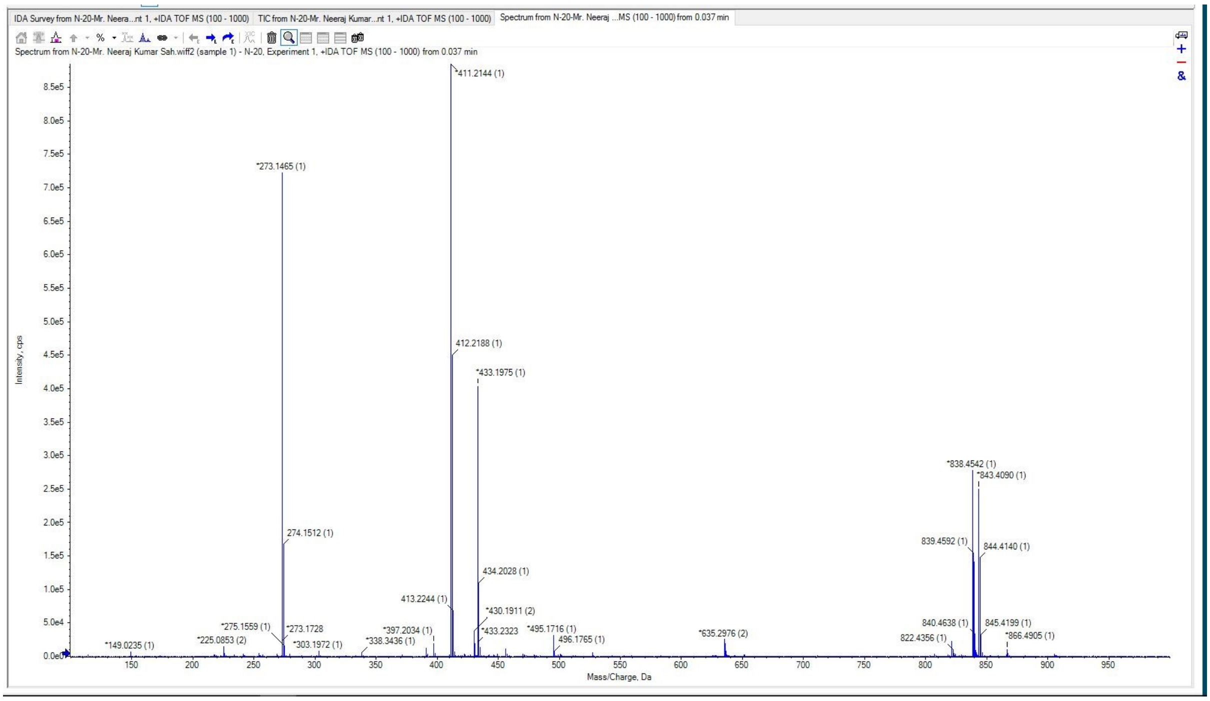

Single crystal X-ray diffraction

Single crystal of compound 2d and 4j were obtained by slow evaporation of ethyl acetate and acetonitrile solution (9:1) for 2d and IPA for 4d. Crystallographic data measurements were obtained on Rigaku XtaLAB Synergy-i dualflex X-ray diffractometer using graphite monochromated Cu-Kα radiation (λ = 1.54184 Å) based diffraction at 298.76 K. The Structures were solved by direct methods using SHELXL-2019 and Olex2 1.5 software and refined with full-matrix least squares on F2 using SHELXL-2019/1.2 Structural illustrations have been drawn with ORTEP-3 for Windows.3 The detailed data collection and structure refinement for 2d and 4j are summarized in Table S1 and S2, respectively. CCDC 2386630 and 2386631 contains the supplementary crystallographic data for compound 2d and 4j, respectively.

**Table S1. Crystal data and structure refinement for 2d.**	
Empirical formula	$C_{17}H_{16}N_2O_2$
Formula weight	280.32
Temperature/K	293
Crystal system	monoclinic
Space group	C2/c
a/Å	20.6773(3)
b/Å	8.90513(11)
c/Å	16.45657(18)
α/°	90
β/°	99.3758(12)
γ/°	90
Volume/Å^3	2989.74(6)
Z	8
ρ_{calc}g/cm^3	1.246
μ/mm^{-1}	0.667
F(000)	1184.0
Crystal size/mm^3	0.03 × 0.027 × 0.025
Radiation	Cu Kα (λ = 1.54184)
2Θ range for data collection/°	8.668 to 144.08
Index ranges	$-25 \le h \le 25, -10 \le k \le 10, -20 \le l \le 18$
Reflections collected	16352
Independent reflections	2930 [R_{int} = 0.0221, R_{sigma} = 0.0140]
Data/restraints/parameters	2930/0/201
Goodness-of-fit on F^2	1.057
Final R indexes [I>=2σ (I)]	R_1 = 0.0432, wR_2 = 0.1228
Final R indexes [all data]	R_1 = 0.0490, wR_2 = 0.1285
Largest diff. peak/hole / e Å^{-3}	0.23/-0.17

Figure S1. ORTEP diagram of compound **2d** with thermal ellipsoid of 50% probability

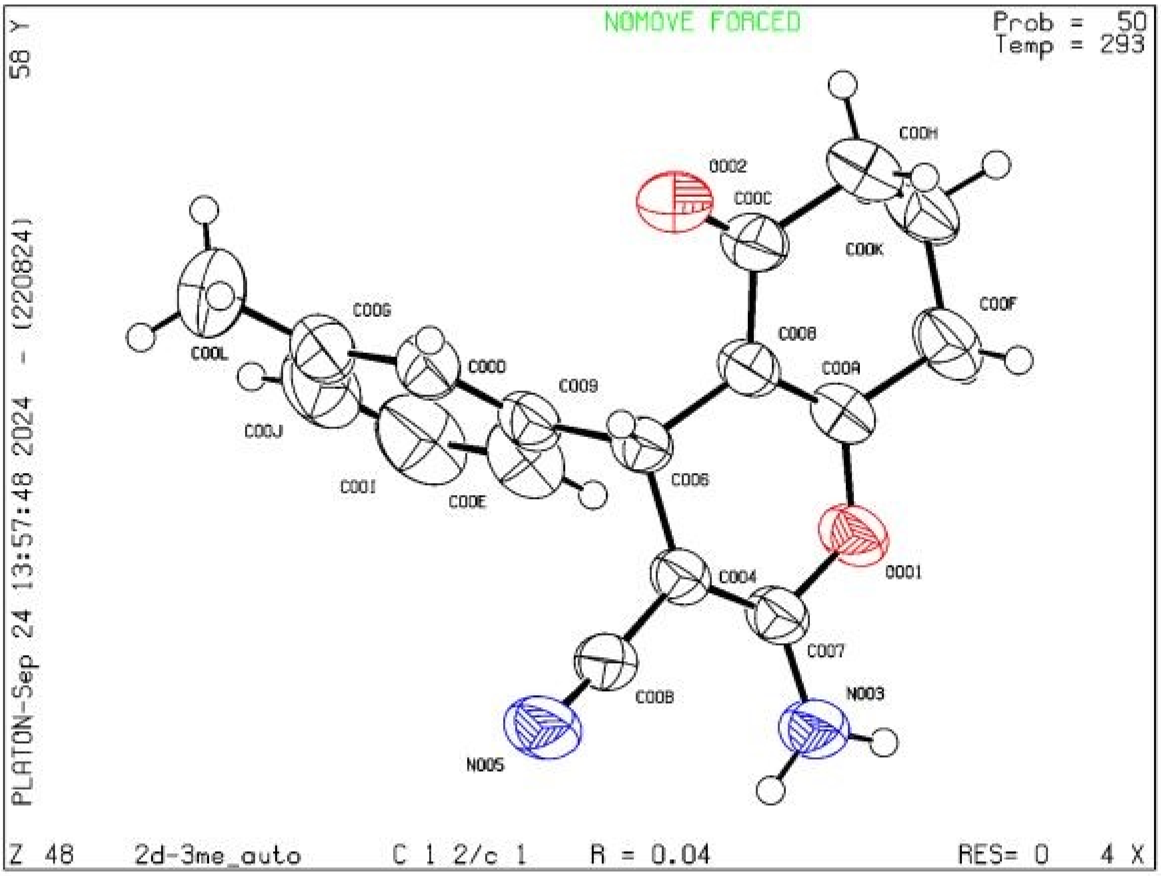

**Table S2 Crystal data and structure refinement for 4j.**	
Identification code	N-20_auto
Empirical formula	$C_{25}H_{30}O_5$
Formula weight	410.49
Temperature/K	293(2)
Crystal system	orthorhombic
Space group	Pbca
a/Å	20.5603(3)
b/Å	10.1414(2)
c/Å	21.5355(3)
α/°	90
β/°	90
γ/°	90
Volume/Å^3	4490.37(13)
Z	8
ρ_{calc}g/cm^3	1.214
μ/mm^{-1}	0.675
F(000)	1760.0
Crystal size/mm^3	0.2 × 0.16 × 0.13
Radiation	Cu Kα (λ = 1.54184)
2Θ range for data collection/°	8.212 to 144.238
Index ranges	-17 ≤ h ≤ 25, -12 ≤ k ≤ 12, -26 ≤ l ≤ 26

Reflections collected	25374
Independent reflections	4409 [R_{int} = 0.0677, R_{sigma} = 0.0414]
Data/restraints/parameters	4409/0/278
Goodness-of-fit on F^2	1.102
Final R indexes [I>=2σ (I)]	R_1 = 0.0567, wR_2 = 0.1749
Final R indexes [all data]	R_1 = 0.0639, wR_2 = 0.1832
Largest diff. peak/hole / e Å^{-3}	0.26/-0.20

Figure S2. ORTEP diagram of compound **4j** with thermal ellipsoid of 50% probability

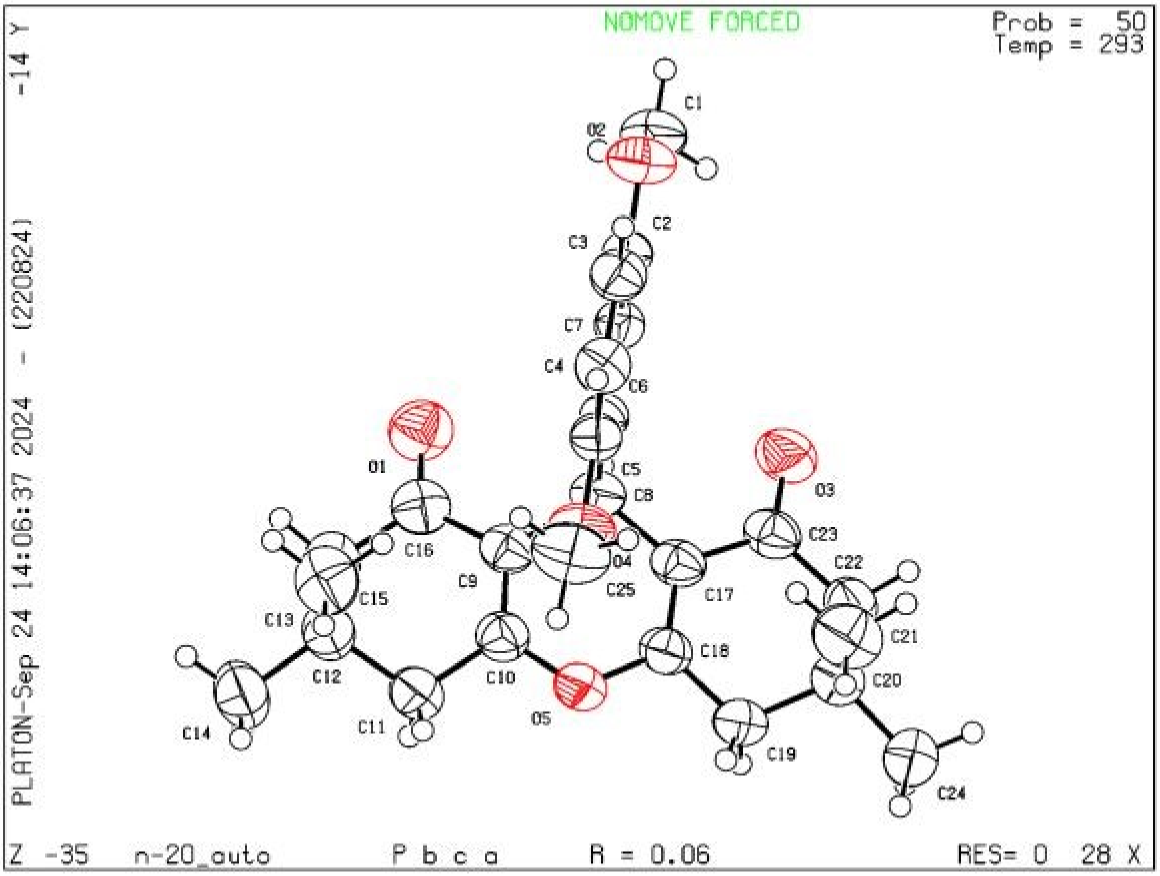

References:

1. G. M. Sheldrick, SHELXS-2014, Program for the crystal structure solution; University of Göttingen: Göttingen, Germany, 2014.

2. L. J. Farrugia, XRDIFF: simulation of X-ray diffraction patterns, J. Appl. Crystallogr., 1997, 30, 565.

Table S1 Cartesian coordinates for the optimized structures shown in Fig. 5

Pre reactant mixture (I^{CW}):

C	-5.21304900	5.03081900	-0.93036700
C	-4.87827600	3.69811900	-0.67691400
C	-4.50472800	3.23466800	0.52249700
N	-4.44565700	2.78355000	1.63829600
H	-3.69553800	2.53453000	2.29557900
N	-5.50641500	6.13261100	-1.16616600
H	-4.86617900	2.99989000	-1.53618500
C	-5.72536300	-0.66557800	1.17709000
H	-4.86729400	-0.27882500	1.77012800
O	-6.79862100	-0.07535000	1.18492700
C	-5.51677900	-1.92360400	0.42295600
C	-4.30114600	-2.61474400	0.54530600
C	-6.52911600	-2.43203400	-0.41393300
C	-4.11302600	-3.82264500	-0.14354000
H	-3.48418700	-2.21108000	1.17406600
C	-6.33044100	-3.62144100	-1.11031300
H	-7.46564500	-1.88147400	-0.51070700
C	-5.11895200	-4.31802500	-0.97351500
H	-3.16070600	-4.36123600	-0.03640500
H	-7.10874100	-4.01125100	-1.76325200
H	-4.96305200	-5.24709700	-1.52305000
O	-1.31567100	-3.04782400	0.21152100
O	-2.24625900	2.20269400	-0.49356300
O	-3.21175400	0.55637100	3.79850600
O	-0.42161600	0.24720300	3.87678100
O	2.07496300	-0.56123500	5.16494400
O	1.83296500	1.48692300	3.03852600
O	0.02377800	-0.53674200	1.46733900

O	1.16424400	-3.44082100	0.95862000
O	3.01689100	-0.80623100	2.52440800
O	3.22496600	3.90169300	2.61963100
O	3.69356500	1.35466400	1.18119000
O	5.75449000	-0.55021700	1.38353000
O	4.42401200	-0.19737200	-1.05222100
O	3.74246100	-2.42917900	0.46384800
O	4.24474300	-0.44928400	-3.96115800
O	2.91750600	-4.80688000	-0.77039100
O	-0.76117800	0.64808800	-5.18461400
O	-2.18943100	0.80399900	-2.77253600
O	-1.96957200	-4.05571700	-2.52081200
O	-1.94611600	4.71059700	0.79857000
O	0.55386200	-3.23460700	-1.60301200
O	3.00567200	-2.26768700	-2.20502700
O	-2.92802300	0.11170900	0.91058900
O	-1.04340500	-4.43203800	2.64848500
O	-1.59851500	2.04745100	2.04170200
O	0.72944600	-2.21196700	3.32545200
O	2.85098800	1.59245600	-2.31212800
O	0.58710300	3.61232900	1.68860000
O	2.00868900	-0.74626100	-0.34240100
O	0.78716700	1.63101900	0.04895300
O	-1.91058100	-1.74688100	2.63577500
O	1.47164600	-0.31285600	-3.49268600
O	-4.69022500	0.68031900	-1.26436300
O	-0.52151800	-0.38081000	-1.17941100
O	2.22136800	4.28720100	-2.82809500
O	2.61349400	3.42495800	-0.18492000
O	-0.00720200	3.82889400	-1.00643900

O	-2.84923300	-1.45841100	-1.42086600
O	-0.91968700	-1.60771300	-3.37215600
O	0.33874900	2.00737200	-2.97853200
Si	0.56964300	-0.00673900	-0.00260000
Mo	3.16551100	-0.41648300	-2.65739100
Mo	-1.93385300	0.28209400	2.72098100
Mo	1.70699500	3.02474000	-1.82581600
Mo	4.16004300	-0.45467800	0.82509400
Mo	-1.04406000	3.30720100	0.50313700
Mo	2.32415300	-3.23998000	-0.53487700
Mo	-3.04663300	0.37303100	-0.97355600
Mo	-0.47995100	0.38572200	-3.53685700
Mo	1.49010700	-0.46930500	3.57923800
Mo	-0.57628200	-2.96496400	1.94789400
Mo	-1.24897400	-2.69127100	-1.82754000
Mo	2.31753500	2.78069500	1.73524500

Intermediate complex 2 (III^{CW})

C	-6.38861300	2.42244000	0.05960800
C	-5.24722900	1.58726900	-0.28961000
C	-5.25637200	1.26790200	-1.71361400
N	-5.29241300	1.01918700	-2.84268000
H	-4.27050600	0.65258700	2.33928200
N	-7.28088300	3.09756000	0.35271900
H	-3.98080519	2.46303184	-0.02897301
C	-5.16015200	0.28420900	0.58050100
H	-4.15371500	-0.19236000	0.37111400
O	-5.20946600	0.68403800	1.95433700
C	-6.31039400	-0.66606100	0.34384500
C	-6.08891300	-1.82895100	-0.40801400

C	-7.58088400	-0.41322000	0.88011300
C	-7.14067100	-2.72014000	-0.63857900
H	-5.08794600	-2.04840500	-0.80775100
C	-8.62846900	-1.30881100	0.64976900
H	-7.74259000	0.47747400	1.48743200
C	-8.41085900	-2.46057700	-0.11365400
H	-6.96612500	-3.62179300	-1.22577500
H	-9.61513000	-1.10937900	1.06616600
H	-9.22861000	-3.15697500	-0.29539800
O	-0.57823800	-1.26824700	3.19132400
O	3.68690700	-2.19667100	-0.19069700
O	0.18579100	-5.34991000	-0.36077000
O	-1.19497400	-3.17842600	-1.45587700
O	-3.60507100	-1.99734000	-2.59226900
O	-0.87893000	-1.04179800	-3.06964800
O	-0.37636000	-1.22981000	0.00907500
O	-2.34367300	0.24226300	1.94364500
O	-2.52340000	0.45210100	-1.66235600
O	0.40909200	-0.04804500	-5.38130100
O	-0.61614700	1.58083200	-3.09477600
O	-2.85624000	3.26767200	-2.67439200
O	-0.42765000	3.54830000	-1.16284900
O	-2.42442700	2.37055800	0.14295200
O	1.37777100	5.31245800	0.36030600
O	-2.86919200	2.84910500	2.87198600
O	4.99233700	2.11605300	2.42444300
O	4.24027700	-0.51340100	1.97453100
O	0.82862900	0.01315000	5.51737200
O	4.31237800	-3.05534100	-2.81243200
O	-0.35292400	1.35146400	3.30343200

O	-0.41161500	3.59792400	1.62940900
O	1.66251600	-3.27551500	1.12758900
O	-3.15228300	-2.41079800	2.91523100
O	1.62541100	-3.19765800	-1.49523600
O	-2.88708000	-1.69205800	0.11967100
O	2.19506100	3.00953100	-1.26087600
O	1.87562300	-1.42878600	-3.44913300
O	0.00143700	1.40298700	0.51635200
O	1.29369200	0.25947800	-1.50411700
O	-1.02758700	-3.33410100	1.32643800
O	2.34178100	2.87592100	1.38055800
O	4.09572800	-3.33303500	2.53226800
O	1.96652600	-0.31339600	1.00137100
O	4.52422200	2.53655900	-2.77565200
O	2.14013600	1.34631700	-3.57903900
O	3.91445200	-0.28045300	-2.17107500
O	2.04597800	-1.58971500	3.32838700
O	2.39065600	1.05633700	3.35000600
O	3.98777200	1.42927000	-0.11216000
Si	0.70938400	0.01818200	0.00519100
Mo	1.00845400	3.66295500	0.31365200
Mo	0.21484100	-3.66166400	-0.26796100
Mo	3.26817900	1.66895400	-2.04895800
Mo	-1.74374900	2.32461600	-1.80497800
Mo	3.21461500	-2.05105400	-1.99615100
Mo	-1.71357600	2.07438400	1.90997200
Mo	3.06791400	-2.19128000	1.82238100
Mo	3.67293000	1.30465600	1.74674900
Mo	-2.28121100	-1.45286700	-1.68944400
Mo	-1.93526300	-1.76528300	1.93449700

Mo	0.87143700	0.01256700	3.82032700
Mo	0.63086600	-0.01576000	-3.70403700

Intermediate complex 1 (TS^{CW})

C	0.00000000	0.00000000	0.00000000
C	0.00000000	0.00000000	1.57145223
C	1.32190093	0.00000000	2.16774795
N	2.33449834	-0.01481311	2.73166638
H	-2.95353451	-0.57035890	0.93835678
N	-1.11909206	-0.05213775	-0.60660005
H	-0.52684224	0.96631409	1.83635432
C	-0.82870959	-1.16965037	2.18575284
H	-0.64187459	-1.20812442	3.28966604
O	-2.21546407	-0.87387504	2.15577100
C	-0.51150999	-2.51720255	1.56122972
C	-1.21894715	-3.00280445	0.45498243
C	0.52364223	-3.28822269	2.11514035
C	-0.87844724	-4.23830699	-0.10632001
H	-2.03557833	-2.40972915	-0.00019049
C	0.84442465	-4.53266101	1.56728372
H	1.09881079	-2.90276984	2.95302095
C	0.14571621	-5.00852546	0.45162466
H	-1.39825335	-4.57056335	-1.00612580
H	1.65462031	-5.11592130	1.99009505
H	0.41608614	-5.95789250	0.00237442
O	-4.23181370	3.22231259	-2.77025616
O	-2.05726182	3.99325364	-7.63446365
O	-2.06789624	7.62398545	-4.45832175
O	-0.09918146	5.55038010	-3.40672597
O	1.83424660	5.17066958	-1.40966752

O	1.89899172	3.83856114	-4.02681679
O	-1.06169793	2.99577534	-3.13561445
O	-2.68915613	1.90226040	-1.09083433
O	1.41117298	2.32591379	-1.94045050
O	3.99884320	3.00328344	-5.74745288
O	2.34399198	1.26552294	-4.16676650
O	3.40920681	-0.02371608	-2.02884116
O	0.81223663	-0.81799103	-3.27581308
O	1.18076484	0.03850731	-0.51688052
O	-0.41573467	-3.06317253	-4.68439535
O	-3.66122333	-1.17136973	-0.63534956
O	-4.31903593	-1.06286821	-7.82034438
O	-3.83336994	1.87655211	-7.52239856
O	-6.55513919	1.84626229	-3.56626086
O	0.15632232	5.37902705	-8.78214042
O	-4.05976464	0.59740862	-2.86292801
O	-1.97278777	-1.29603486	-2.92362190
O	-3.14810984	5.23268212	-5.58922152
O	-3.40842284	4.41488681	-0.18846486
O	-0.58031526	5.57096866	-6.07186431
O	-0.75416793	4.17701487	-1.06713520
O	0.17185655	-0.80933373	-6.15423934
O	1.73183194	4.06449210	-6.89301975
O	-1.01252847	0.66332908	-4.33069296
O	-0.16205879	2.79051557	-5.67245886
O	-2.61695726	5.29357589	-2.87851673
O	-2.44034033	-1.17129273	-5.66928189
O	-4.81626612	4.46608660	-8.00097382
O	-2.78391941	2.47815891	-5.12352379
O	0.83632242	-0.16175517	-9.08679268

O	1.82898043	1.27363131	-6.77674274
O	0.01119937	2.43707355	-8.32854415
O	-5.17485178	3.26281188	-5.49530048
O	-4.65360527	0.49395746	-5.44209481
O	-1.65849055	0.31614439	-7.68987665
Si	-1.24556845	2.20904404	-4.57549608
Mo	-0.67643831	-1.38546562	-4.61400966
Mo	-1.82489478	5.93316768	-4.47740920
Mo	0.24441348	0.67205623	-7.72355055
Mo	1.87418850	0.54266461	-2.51549044
Mo	-0.12961329	4.35145594	-7.46287730
Mo	-2.75394584	-0.14759903	-1.69503766
Mo	-3.81600373	3.66954793	-6.88531687
Mo	-3.44184596	-0.01734528	-6.79727093
Mo	0.76866261	4.23144464	-2.32945851
Mo	-2.63533027	3.71414335	-1.52376767
Mo	-4.93016650	2.01018396	-4.02425878
Mo	2.33013127	2.78682260	-5.53658078

Combined Products (IV^{CW})

C	6.77509000	0.47434400	-0.66646700
O	2.16905000	-2.24782800	-4.16066600
H	6.23919600	0.80962300	-1.58684000
C	6.15707300	-0.66728800	0.00164300
C	5.58329000	-1.67505100	-0.80399900
C	6.06361400	-0.75997900	1.40107400
C	4.94290900	-2.76353500	-0.21186200
H	5.63342800	-1.59970900	-1.89496200
C	5.41097000	-1.84895600	1.98621300
H	6.46257100	0.03214900	2.03487000

C	4.84106000	-2.84485400	1.18411400
H	4.49056600	-3.53853200	-0.84241500
H	5.32144700	-1.90662100	3.07551300
H	4.29409700	-3.67559600	1.64300700
C	8.40848400	2.23628200	-1.01798700
N	8.81385000	3.14196700	-1.61444200
C	7.91002500	1.11648100	-0.28023100
C	8.70940600	0.73880200	0.84162000
H	2.21174400	-2.47870200	-3.20092400
H	1.49036500	-1.53036700	-4.17364200
N	9.37314800	0.43390700	1.74027300
O	2.13553700	2.10280300	-0.88828800
O	0.63448300	-1.05513200	3.18827900
O	0.26967600	3.55139000	4.02150400
O	-1.72922100	3.34106200	2.05922400
O	-3.81406000	4.43728800	0.35112300
O	-3.67121800	1.62695700	1.28334300
O	-0.67269000	1.65317300	0.42512000
O	0.07656700	2.53910800	-2.47750900
O	-3.28187400	2.19071900	-1.25661500
O	-5.70145700	-0.18668700	1.99040200
O	-4.22810400	-0.15978900	-0.57343600
O	-5.01100900	0.68163800	-3.14087500
O	-3.08578000	-1.34033100	-2.85666500
O	-2.16998500	1.19653800	-3.51897600
O	-1.54884500	-3.62213900	-3.84383800
O	0.12386200	1.52051700	-5.10416700
O	2.26821400	-4.46686800	-0.33361600
O	2.22782100	-2.27389600	1.43916200
O	4.25941600	0.51181800	-2.12847900

O	-1.30478000	-1.31023100	5.21389500
O	1.54393500	0.37269500	-2.77074100
O	-0.50410600	-1.01247000	-3.82396100
O	1.54597400	1.32449500	2.54069400
O	1.49509000	4.83033400	-1.24732500
O	-0.90637300	1.05815000	3.47919700
O	-1.20993900	3.96963600	-0.59069000
O	-2.18243900	-3.07613200	-1.00116000
O	-3.14610600	-0.41042100	3.14132500
O	-1.24047700	-0.13166100	-1.52170400
O	-1.66282200	-0.77809800	1.06551800
O	0.89365100	3.55428500	1.17157900
O	0.29872800	-2.85432600	-1.87322200
O	3.50377100	-0.44995500	3.42077400
O	0.85471500	-0.55012200	0.12212800
O	-2.74370500	-4.81672400	1.14668400
O	-3.79406200	-2.21159800	1.13067700
O	-1.61418800	-2.69538000	2.78604400
O	3.26221400	0.20882100	0.64300800
O	2.62616900	-1.70886900	-1.30736900
O	-0.16315300	-3.46742800	0.64127900
Si	-0.67877700	0.04842400	0.02377700
Mo	-1.32035500	-2.37133700	-2.72545400
Mo	-0.08950300	2.57342200	2.68690300
Mo	-2.19431900	-3.23507400	0.89675900
Mo	-3.58738700	0.41303600	-2.26537000
Mo	-1.22244900	-0.98435200	3.55444800
Mo	-0.26310300	1.00670400	-3.53788900
Mo	2.32495000	-0.37146900	2.20903000
Mo	1.48281300	-2.97542900	-0.16663300

Mo	-2.71229400	3.15448500	0.27400600
Mo	0.75563000	3.39937100	-0.72679100
Mo	2.78870900	0.20648500	-1.34095600
Mo	-4.08604500	-0.21159200	1.48465600
